# Supplementary material for: Aerobic Biostabilization of the Organic Fraction of Municipal Solid Waste—Monitoring Hot and Cold Spots in the Reactor as a Novel Tool for Process Optimization
Source: Materials (Basel). 2022 May 4;15(9):3300. doi: 10.3390/ma15093300 (PMC9104568; doi:10.3390/ma15093300)
Supplement: Supplementary file 1 [file materials-15-03300-s001.zip › materials-1693158-supplementary.pdf]

materials

Article

Aerobic Biostabilization of the Organic Fraction of Municipal Solid Waste—Monitoring Hot and Cold Spots in the Reactor as a Novel Tool for Process Optimization

Sylwia Stegenta-Dąbrowska [1], Peter F. Randerson [2] and Andrzej Białowiec [1,*]

[1] Department of Applied Bioeconomy, Wrocław University of Environmental and Life Sciences, 37a, Chełmońskiego Str., 51-630 Wrocław, Poland; sylwia.stegenta@upwr.edu.pl
[2] School of Biosciences, Cardiff University, Sir Martin Evans Building, Museum Avenue, Cardiff CF10 3AX, UK; randerson@cardiff.ac.uk
* Correspondence: andrzej.bialowiec@upwr.edu.pl

Abstract: The process of aerobic biostabilization (AB) has been adopted for treatment of the organic fraction of municipal solid waste (OFMSW). However, thermal gradients and some side effects in the bioreactors present difficulties in optimization of AB. Forced aeration is more effective than natural ventilation of waste piles, but "hot and cold spots" exist due to inhomogeneous distribution of air and heat. This study identified the occurrence of hot and cold spots during the OFMSW biostabilization process at full technical scale. It was shown that the number of hot and cold spots depended on the size of the pile and aeration rate. When the mass of stabilized waste was significantly lower and the aeration rate was two-fold higher the number of anaerobic hot spots decreased, while cold spots increased. In addition, the results indicated that pile construction with sidewalls decreased the number of hot spots. However, channelizing the airflow under similar conditions increased the number of cold spots. Knowledge of the spatial and temporal distribution of process gases can enable optimization and adoption of the OFMSW flow aeration regime. Temperature monitoring within the waste pile enables the operator to eliminate undesirable "hot spots" by modifying the aeration regime and hence improve the overall treatment efficiency.

Keywords: air flow rate; composting; municipal solid waste; monitoring; optimization; spatial distributions

Citation: Stegenta-Dąbrowska, S.; Randerson, P.F.; Białowiec, A. Aerobic Biostabilization of the Organic Fraction of Municipal Solid Waste—Monitoring Hot and Cold Spots in the Reactor as a Novel Tool for Process Optimization. *Materials* **2022**, *15*, 3300. https://doi.org/10.3390/ma15093300

Academic Editor(s): Rossana Bellopede; Lorena Zichella

Received: 6 April 2022
Accepted: 2 May 2022
Published: 4 May 2022

Publisher's Note: MDPI stays neutral with regard to jurisdictional claims in published maps and institutional affiliations.

Copyright: © 2022 by the authors. Submitted for possible open access publication under the terms and conditions of the Creative Commons Attribution (CC BY) license (https://creativecommons.org/licenses/by/4.0/).

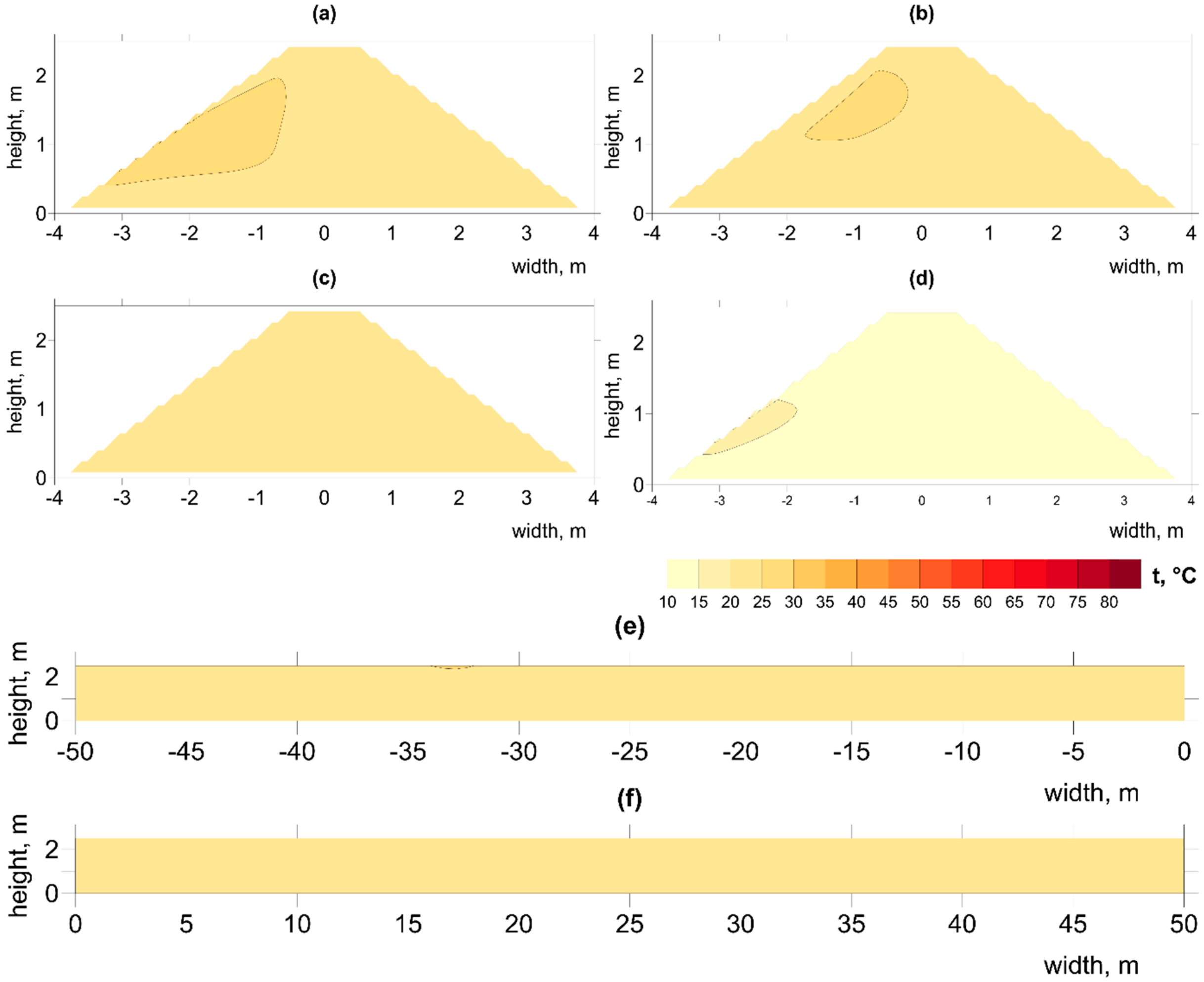

Figure S1. Temperature changes in the pile A1, day 1.

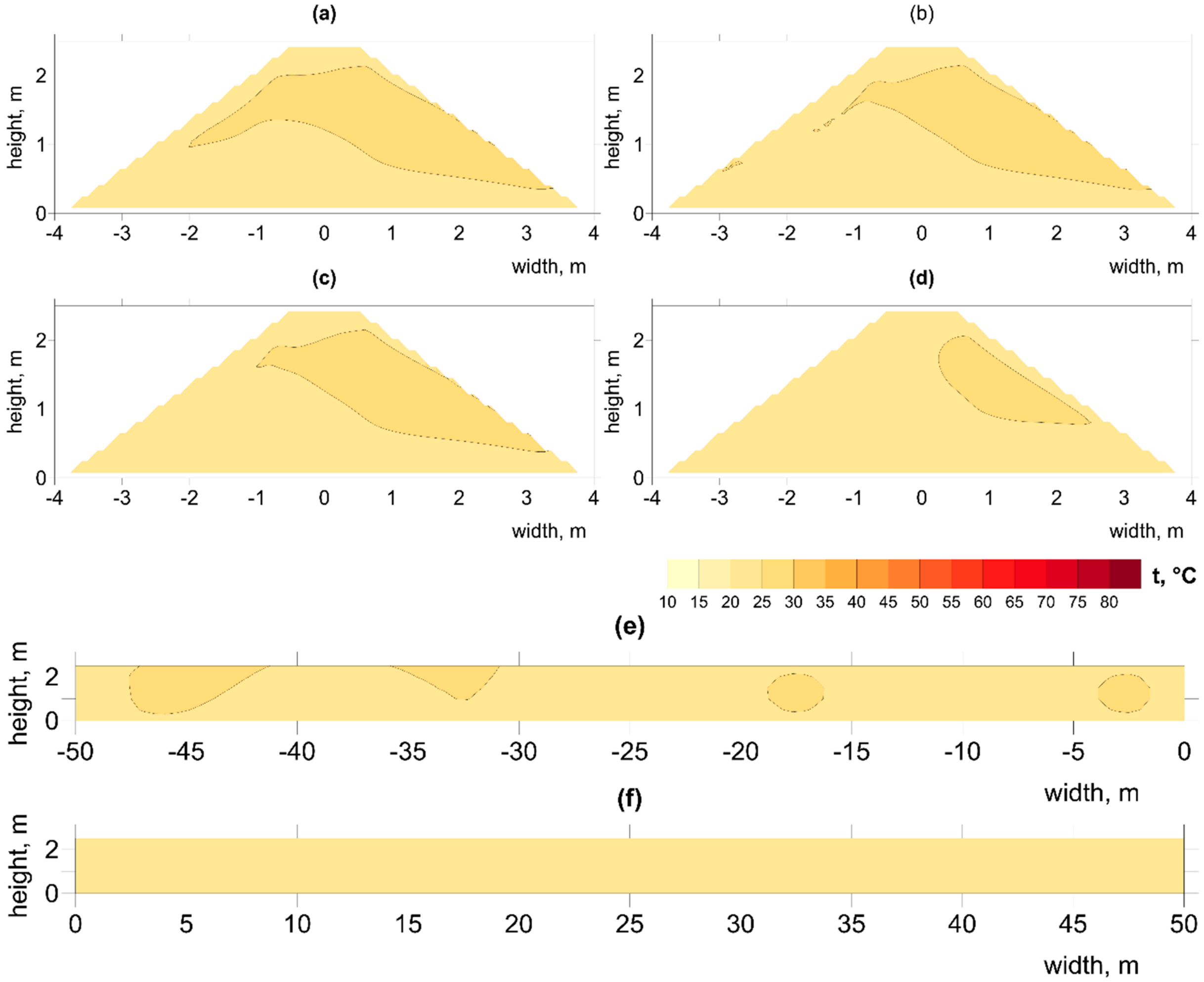

Figure S2. Temperature changes in the pile A1, day 4.

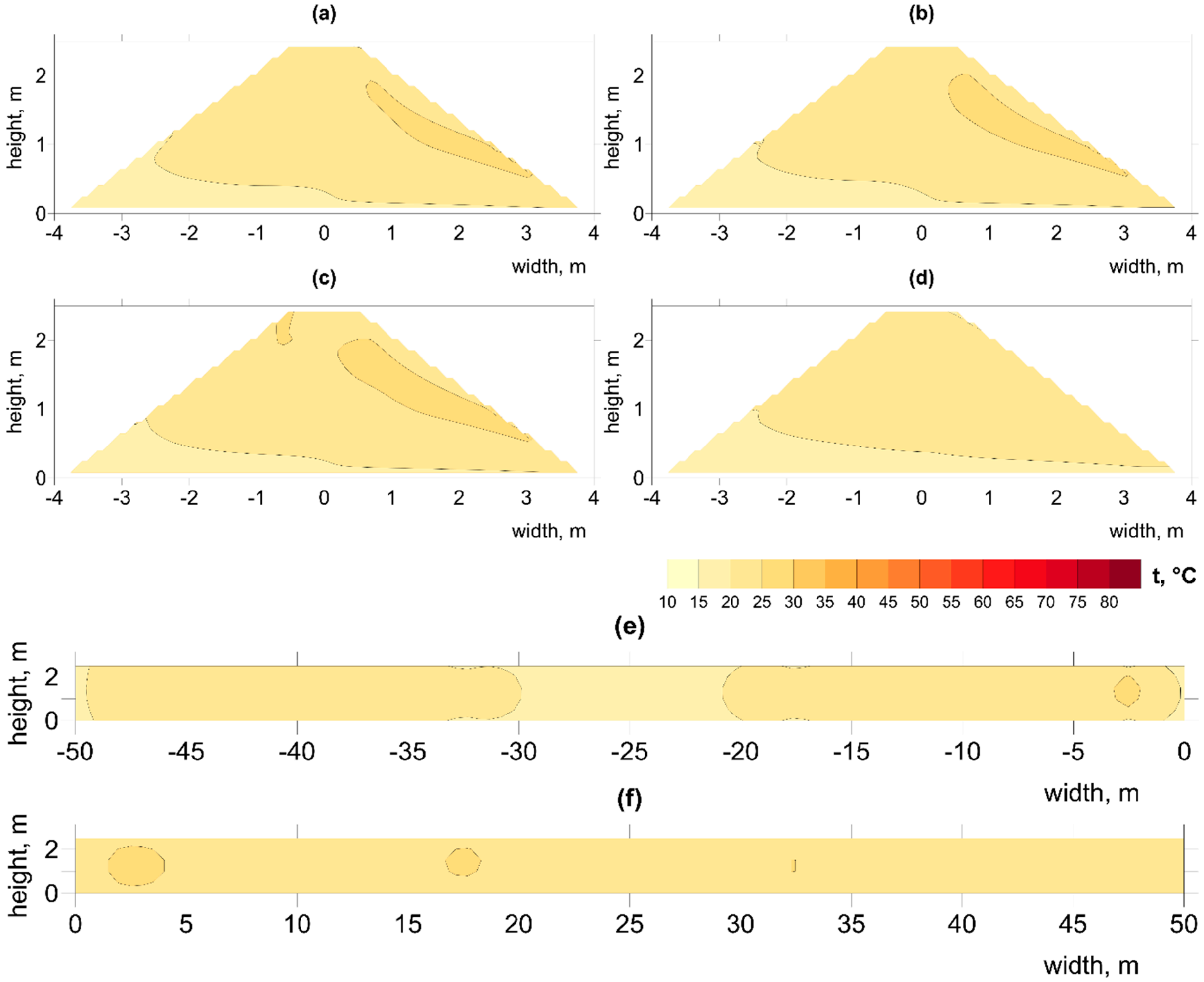

Figure S3. Temperature changes in the pile A1, day 14.

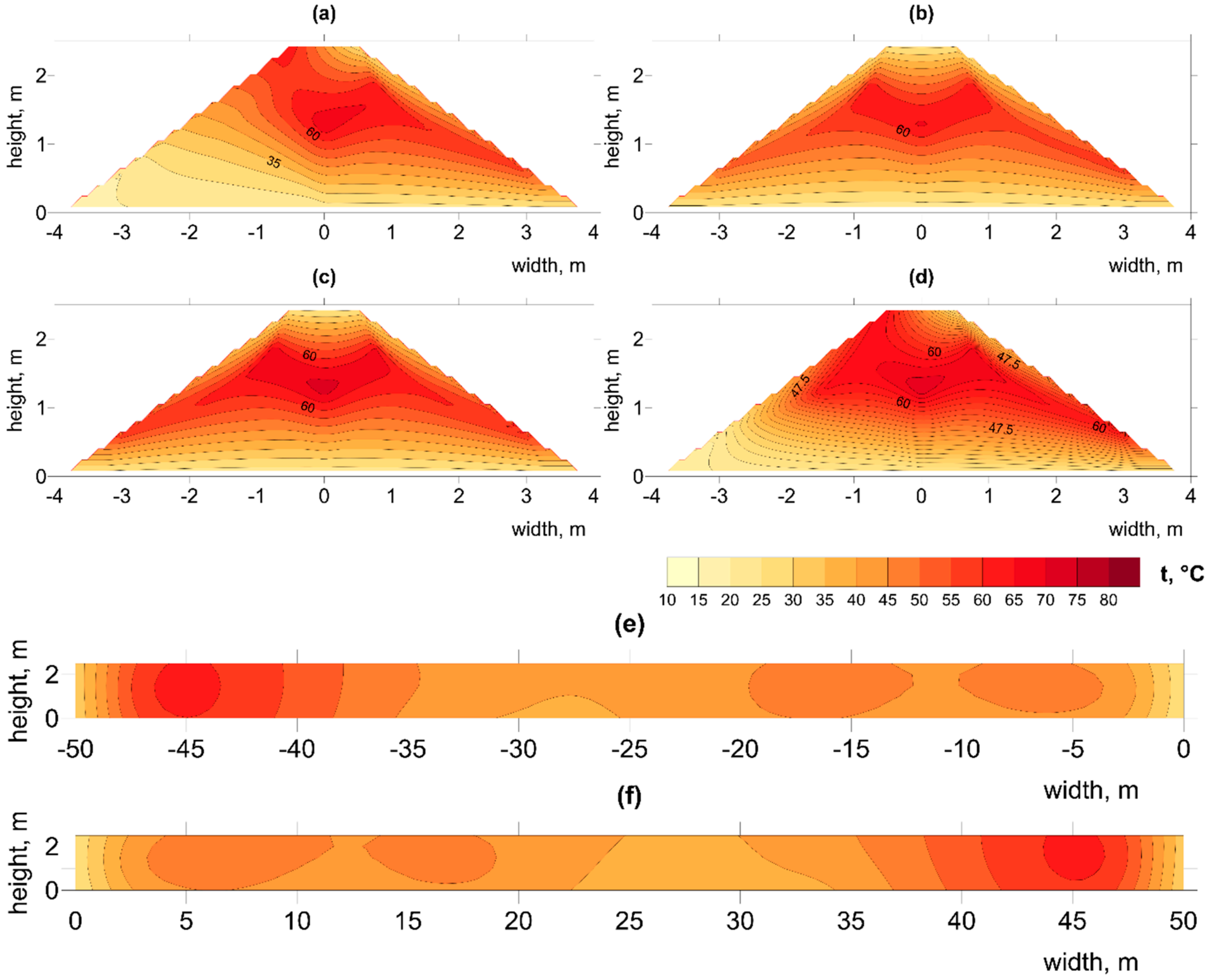

Figure S4. Temperature changes in the pile A1, day 20.

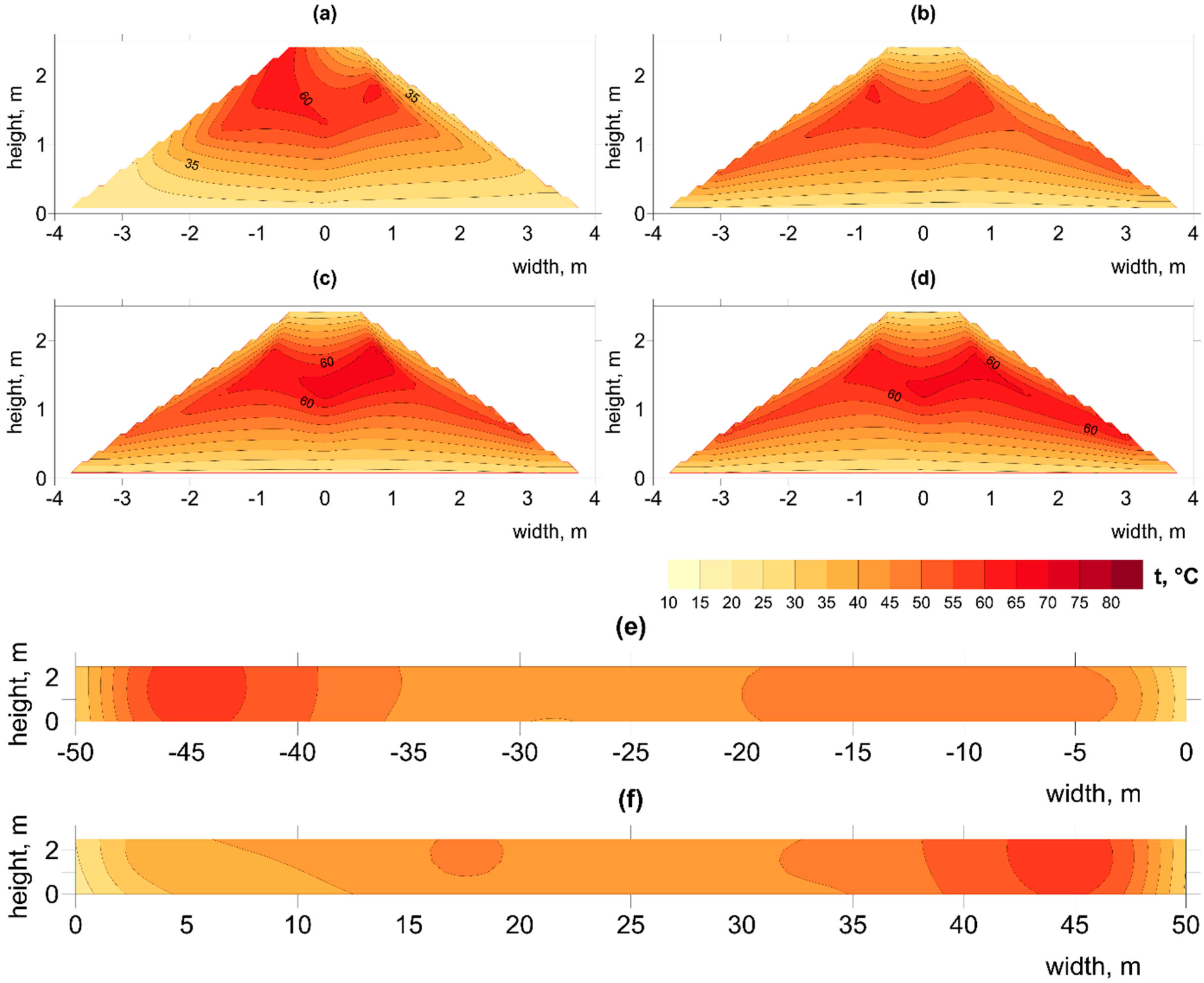

Figure S5. Temperature changes in the pile A1, day 28.

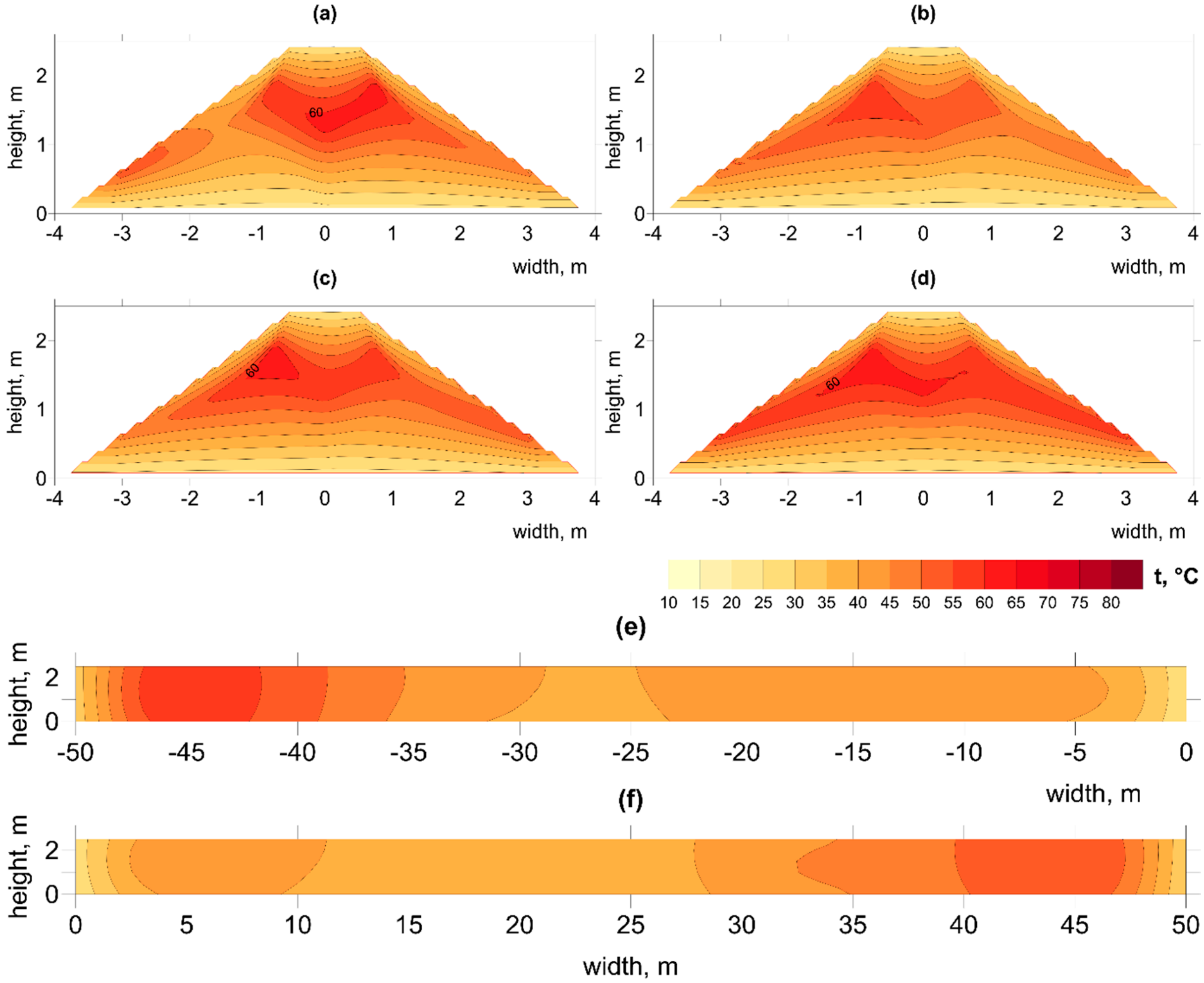

Figure S6. Temperature changes in the pile A1, day 35.

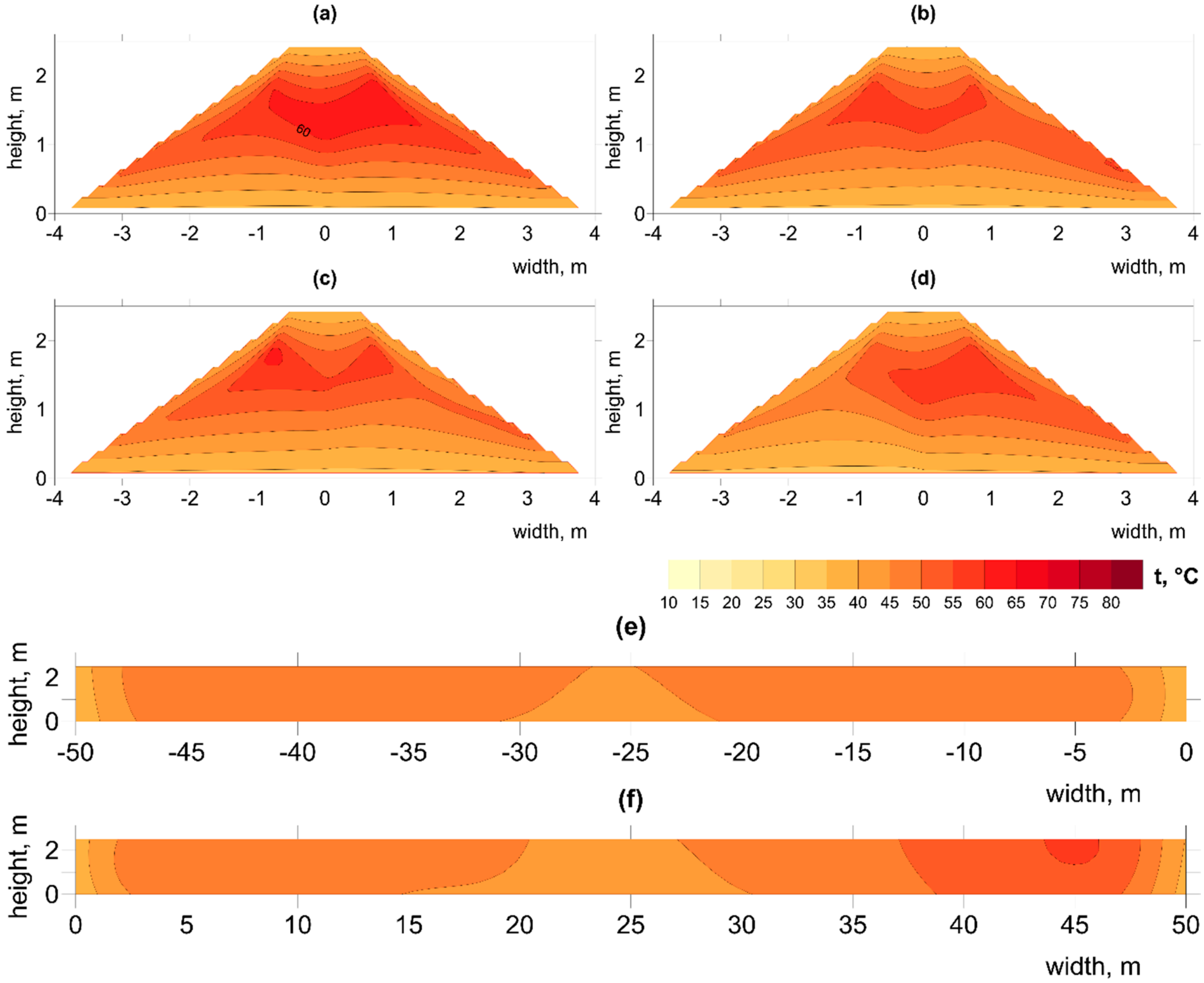

Figure S7. Temperature changes in the pile A1, day 41.

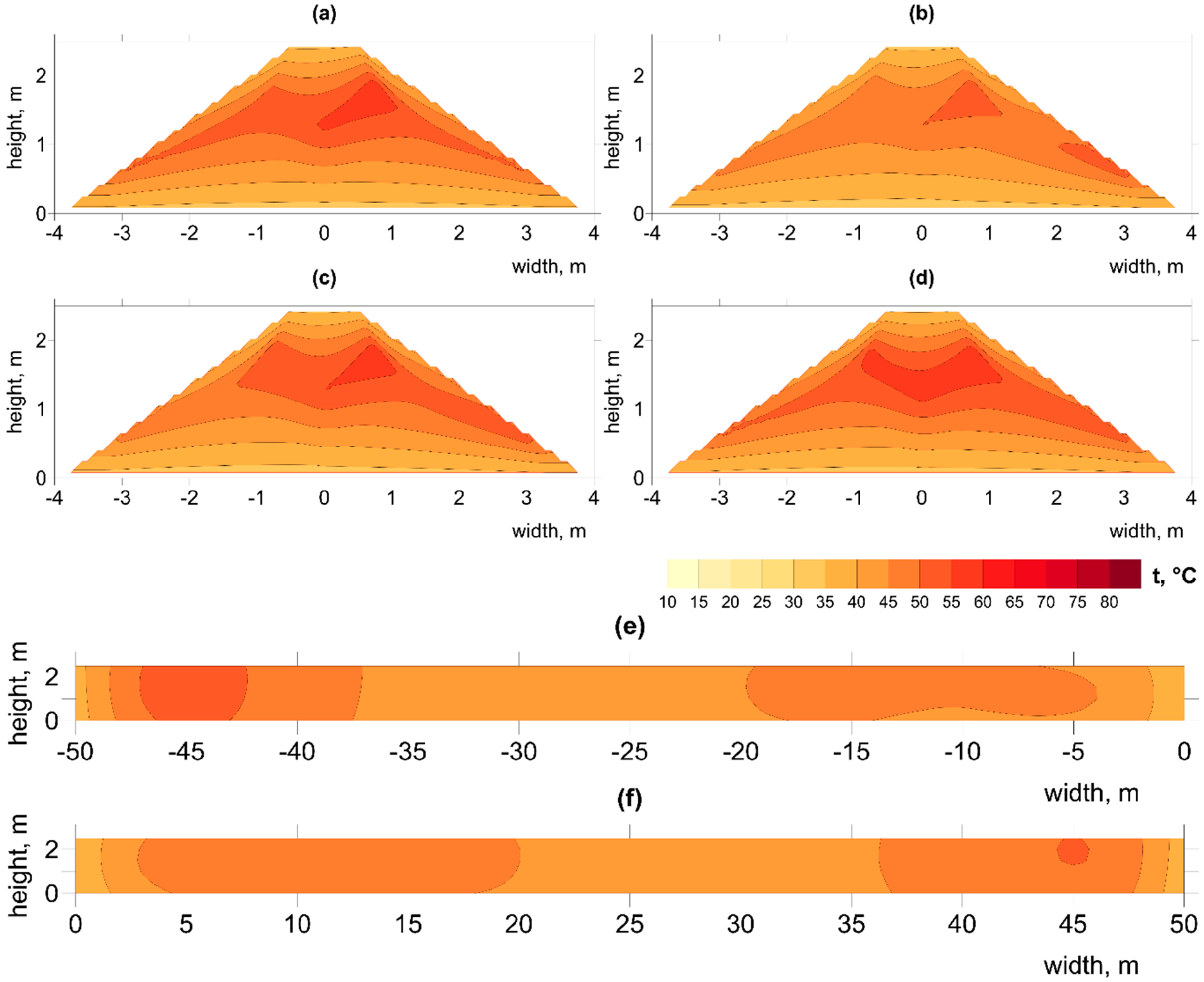

Figure S8. Temperature changes in the pile A1, day 50.

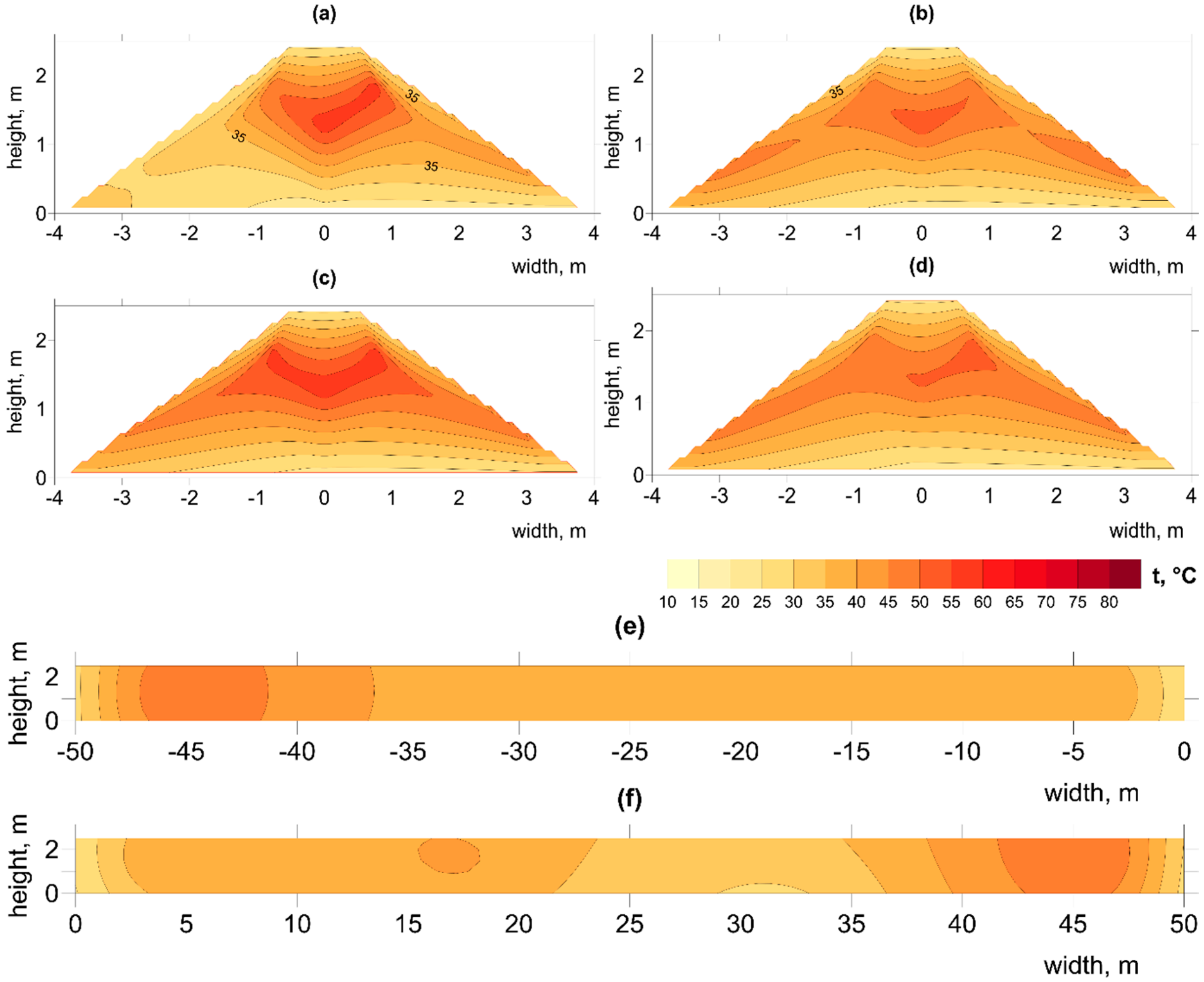

Figure S9. Temperature changes in the pile A1, day 60.

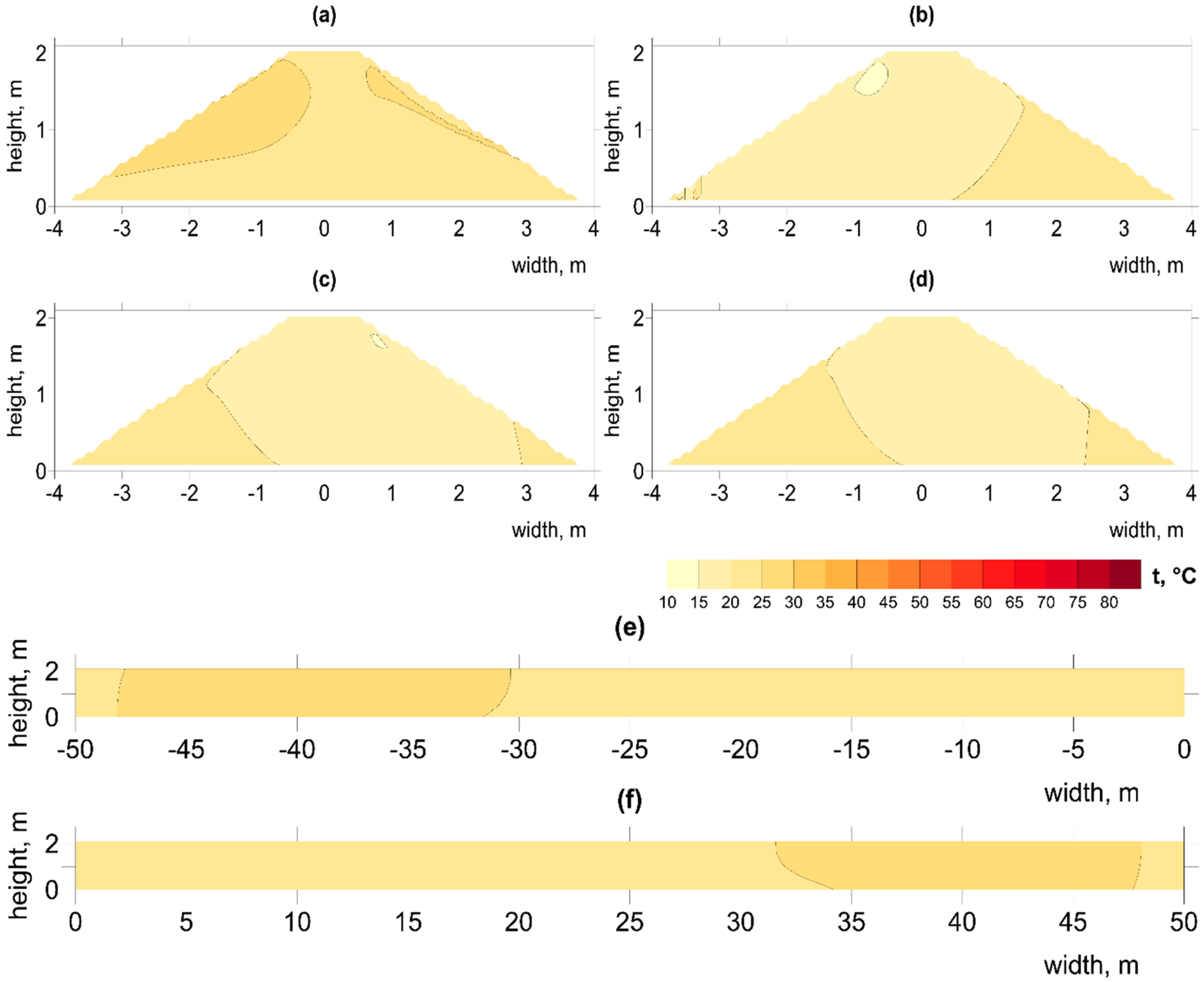

Figure S10. Temperature changes in the pile A2, day 1.

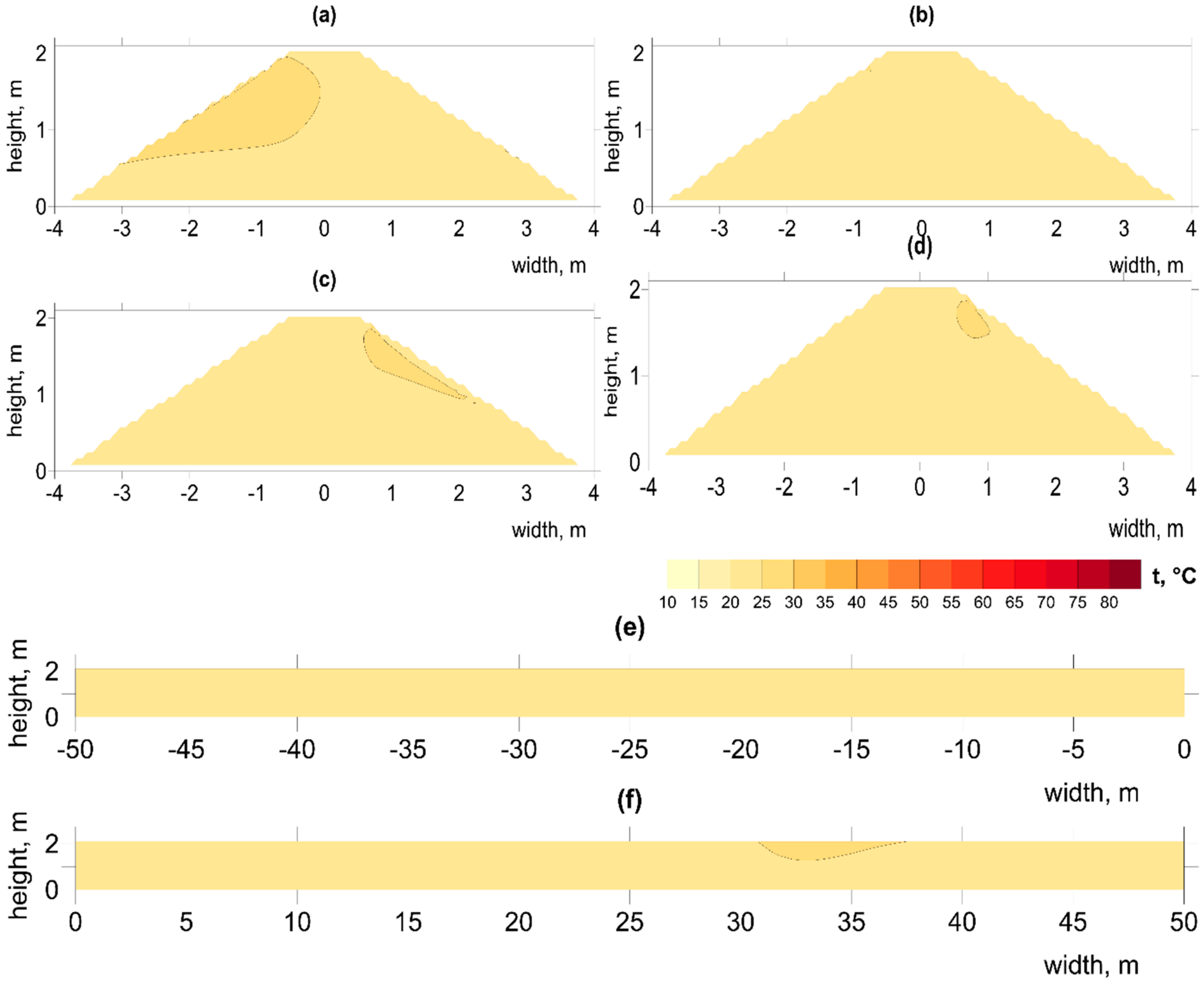

Figure S11. Temperature changes in the pile A2, day 11.

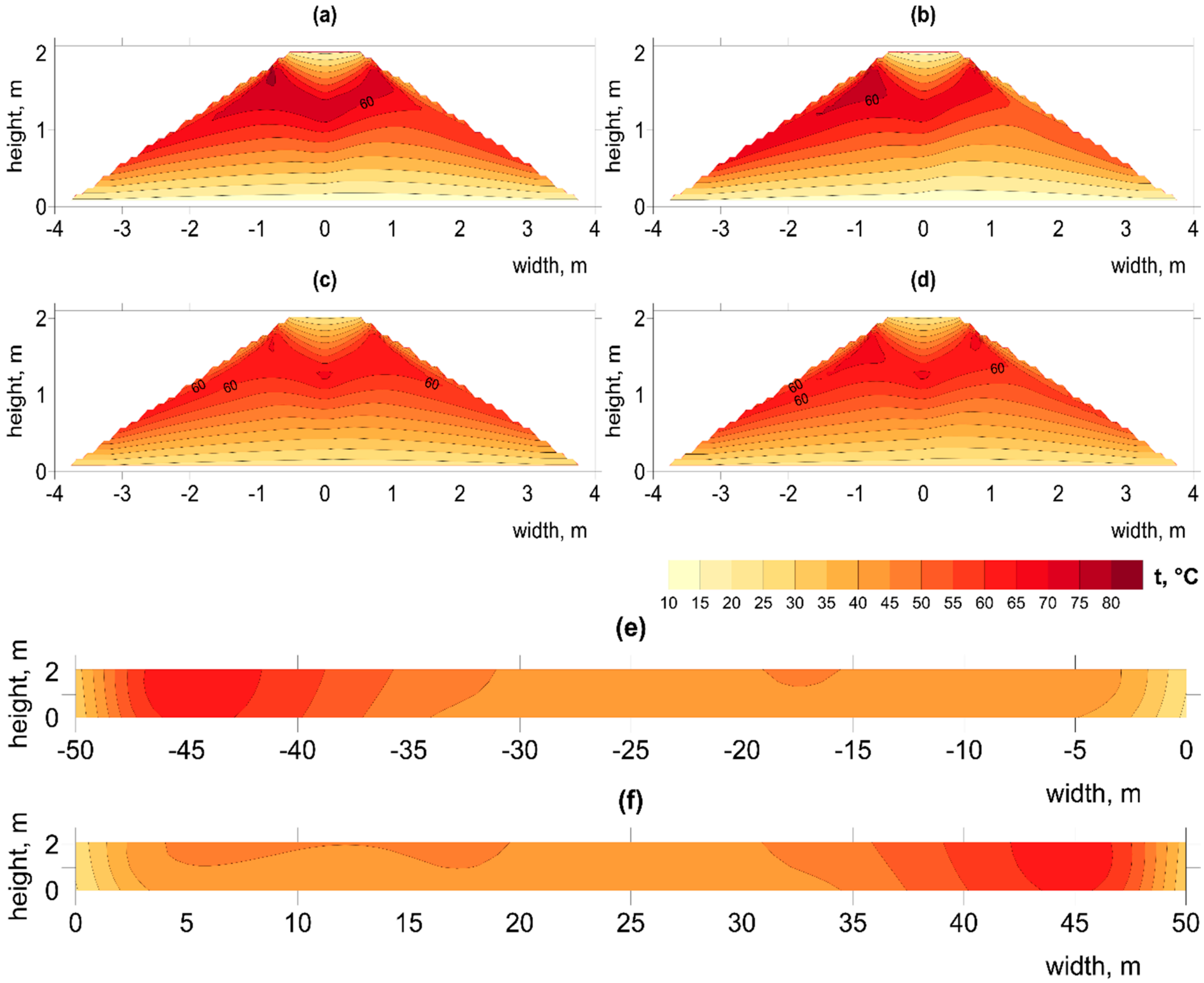

Figure S12. Temperature changes in the pile A2, day 17.

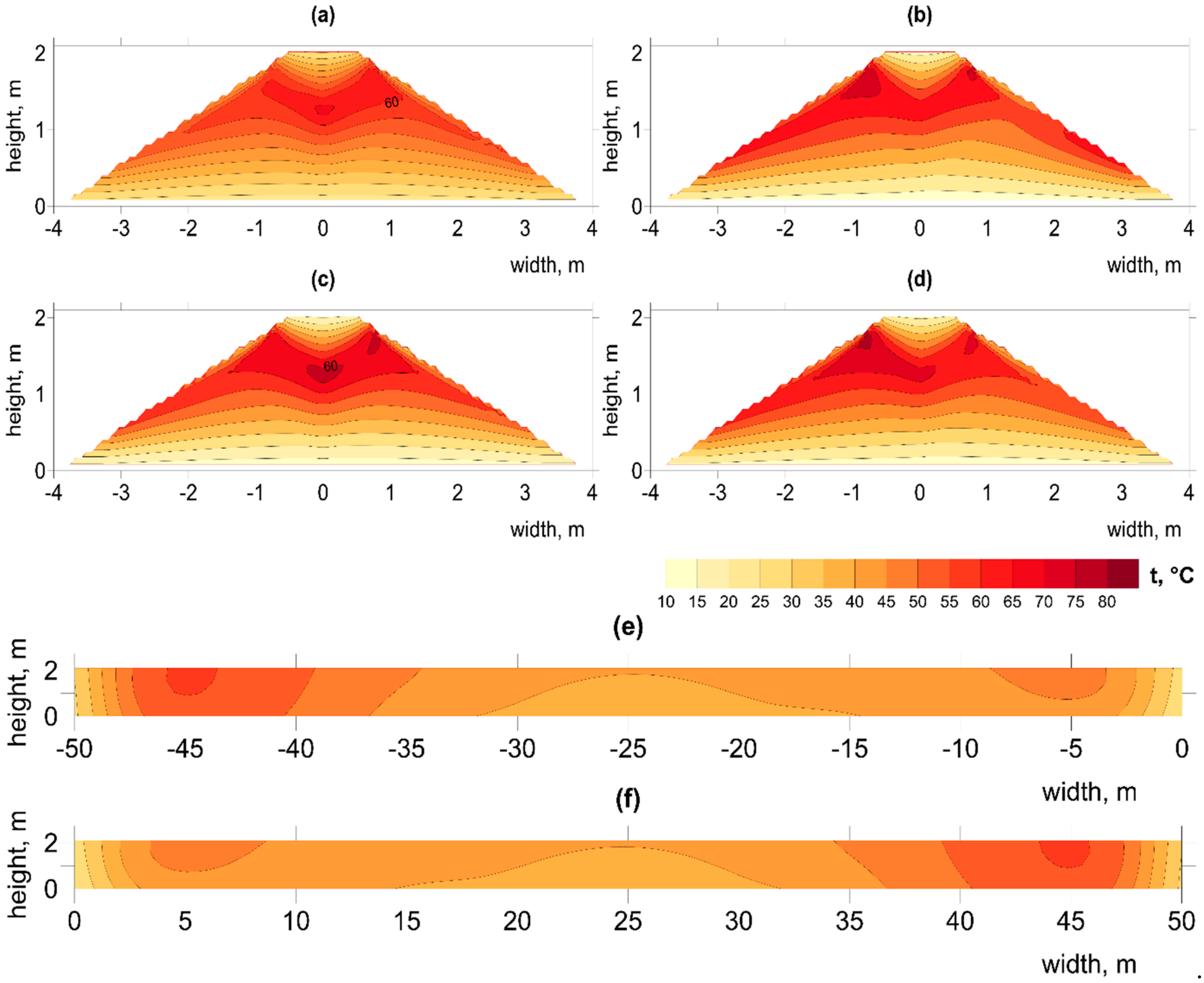

Figure S13. Temperature changes in the pile A2, day 25.

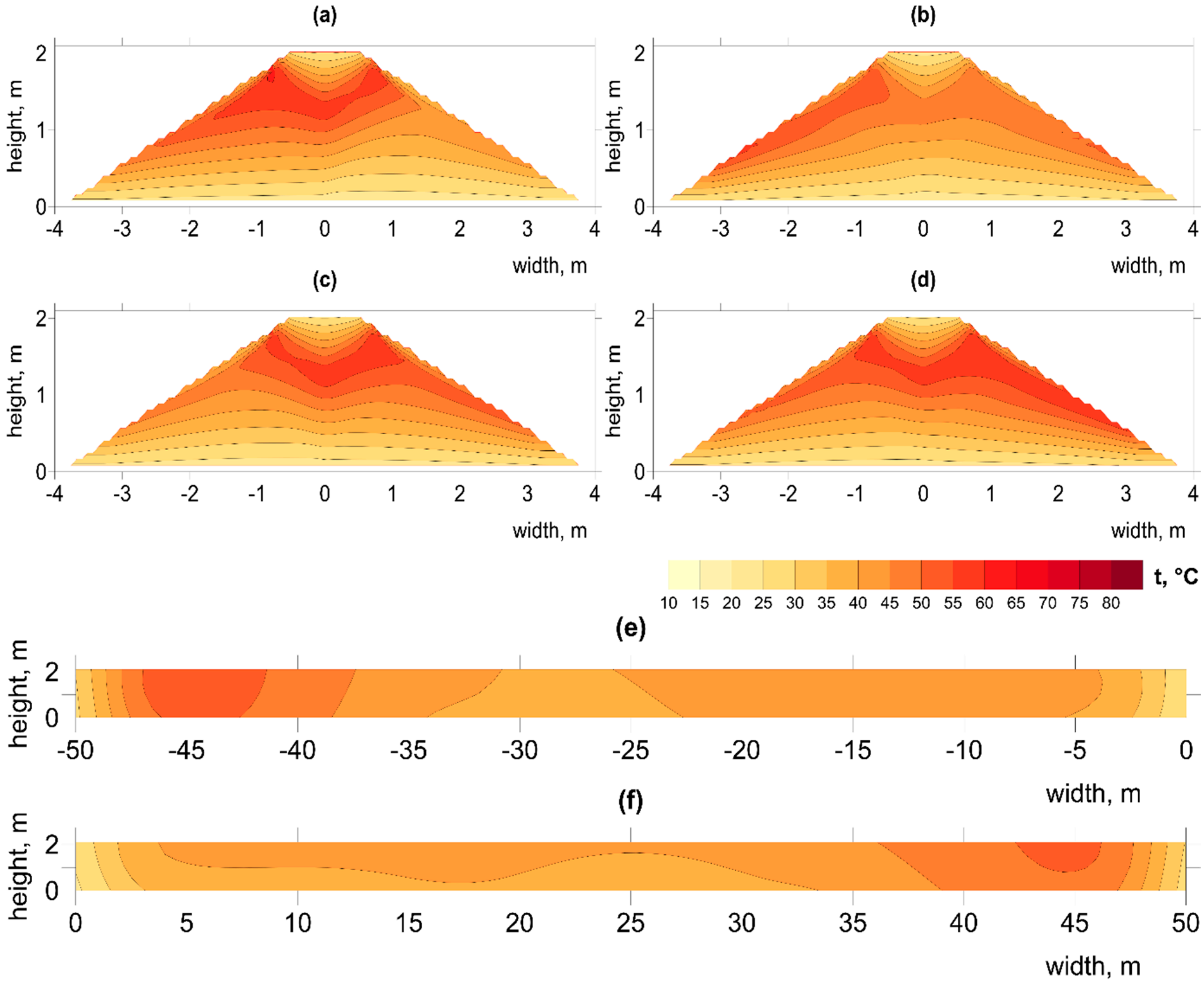

Figure S14. Temperature changes in the pile A2, day 32.

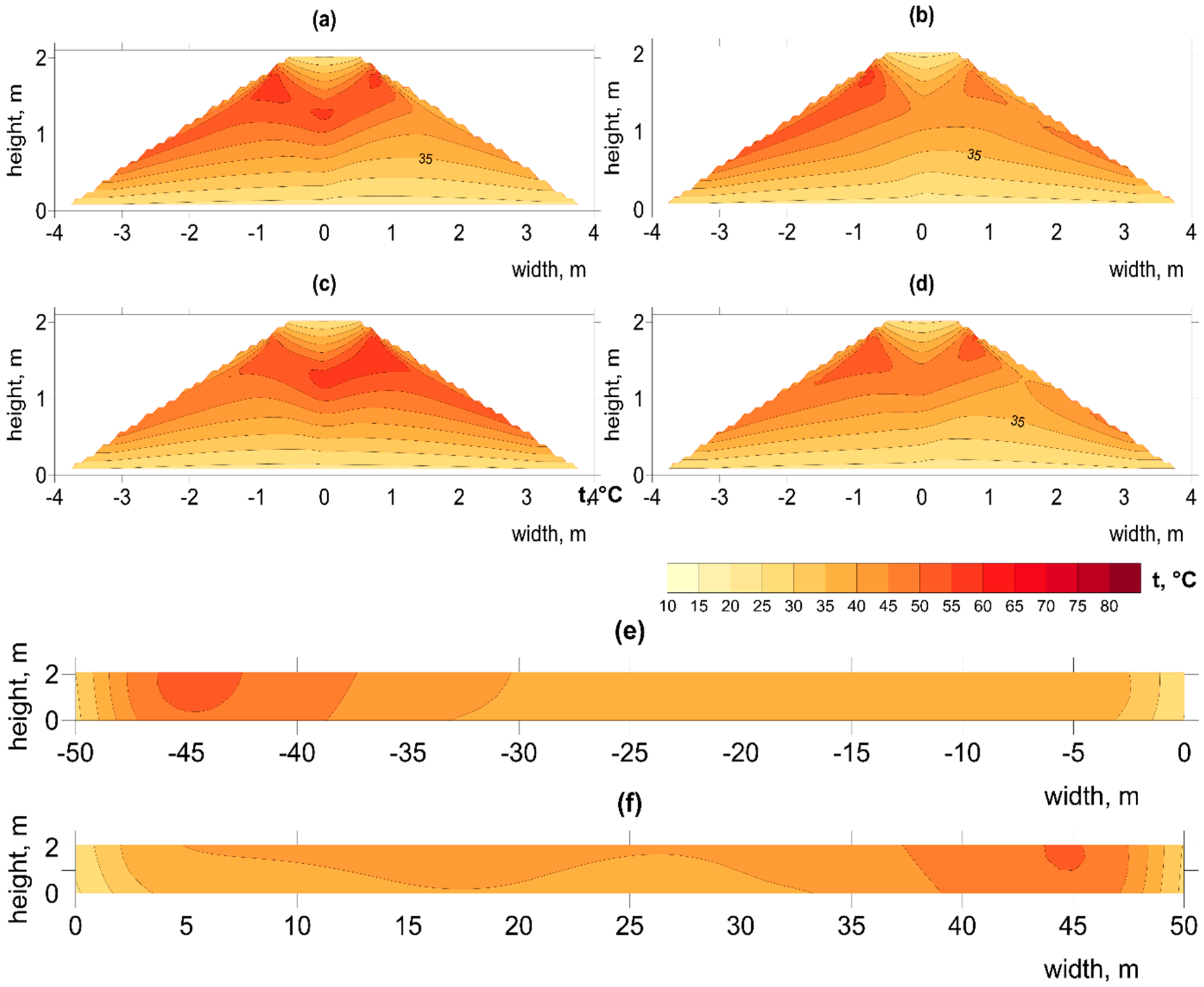

Figure S15. Temperature changes in the pile A2, day 38.

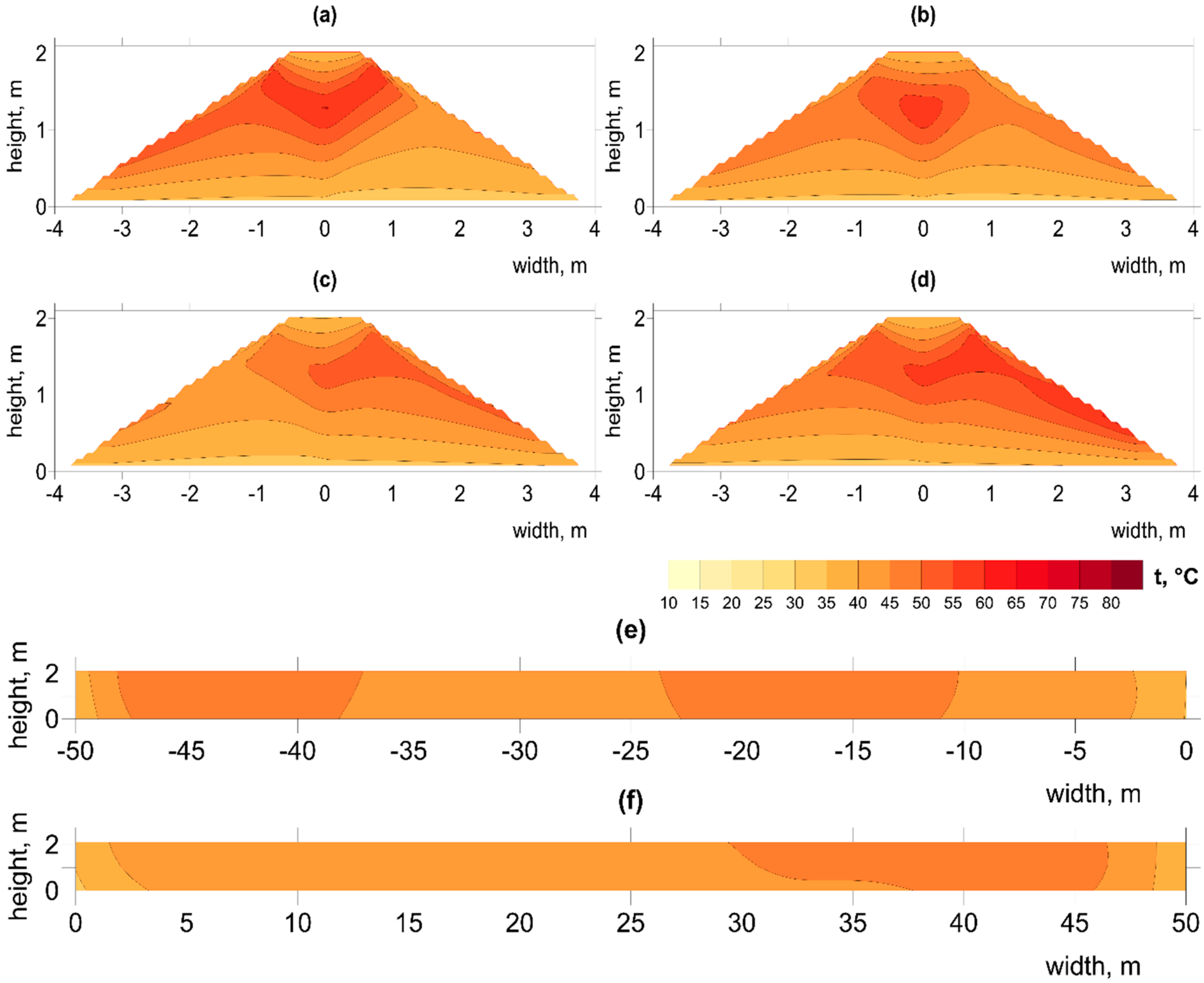

Figure S16. Temperature changes in the pile A2, day 47.

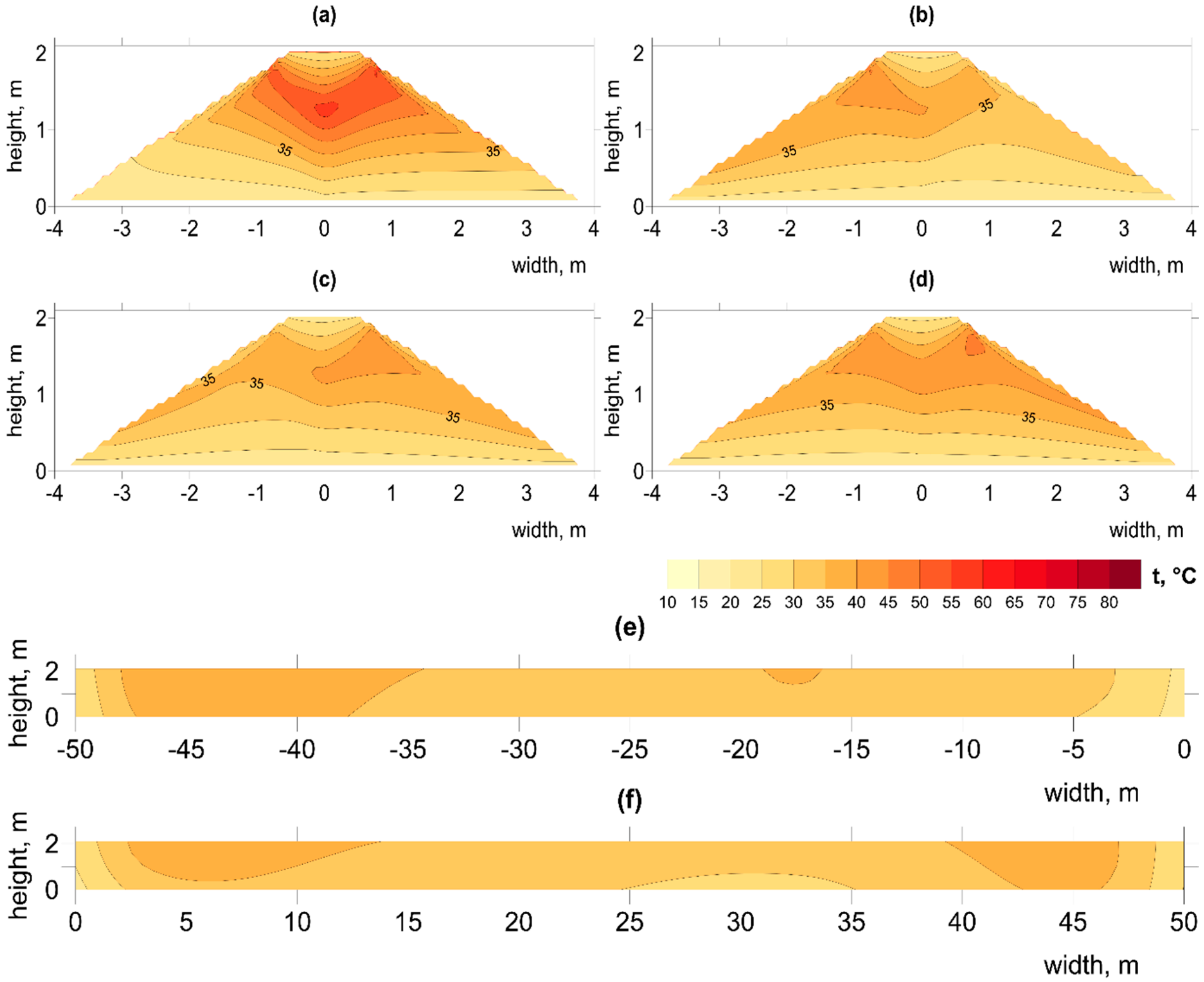

Figure S17. Temperature changes in the pile A2, day 57.

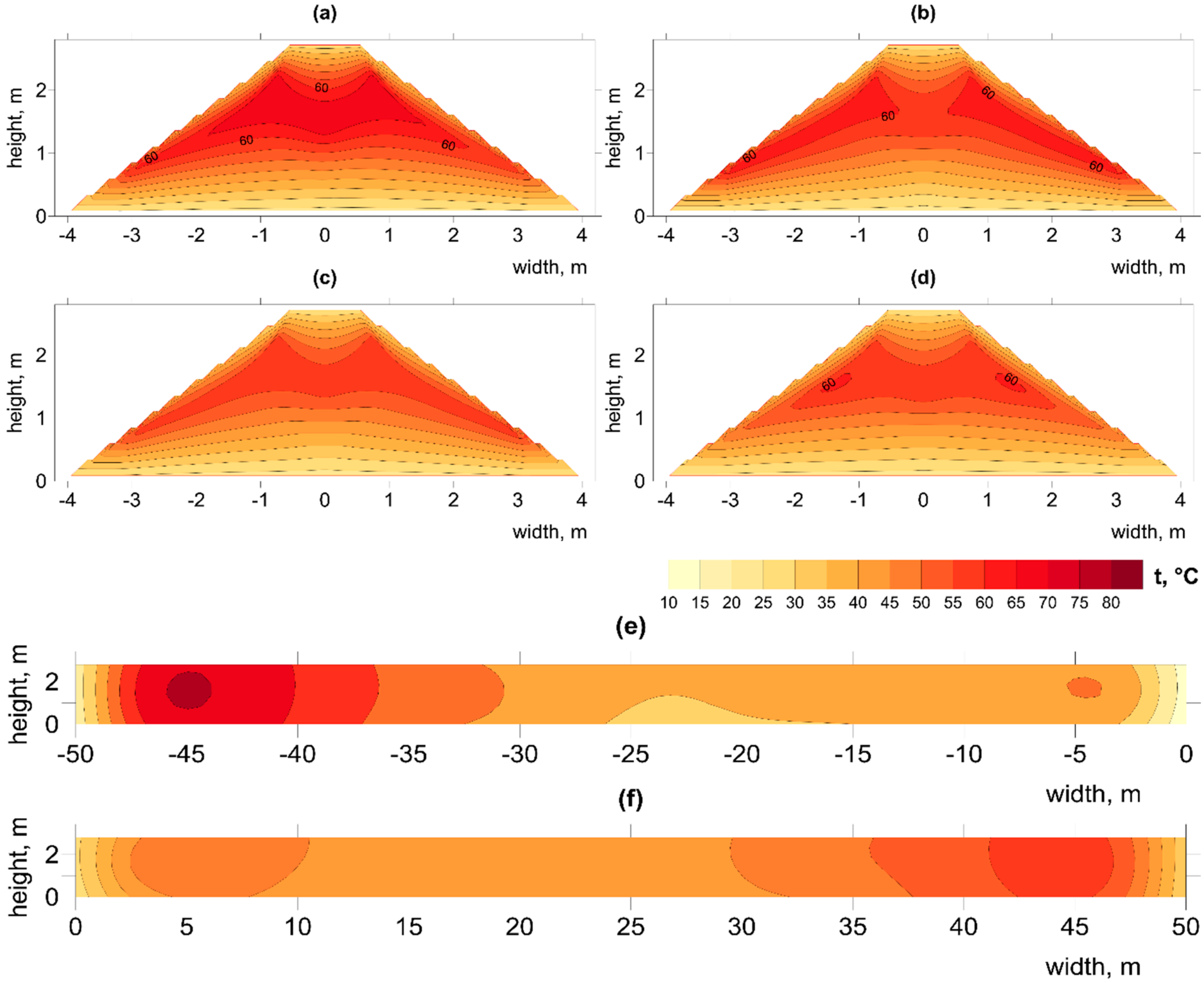

Figure S18. Temperature changes in the pile B1, day 9.

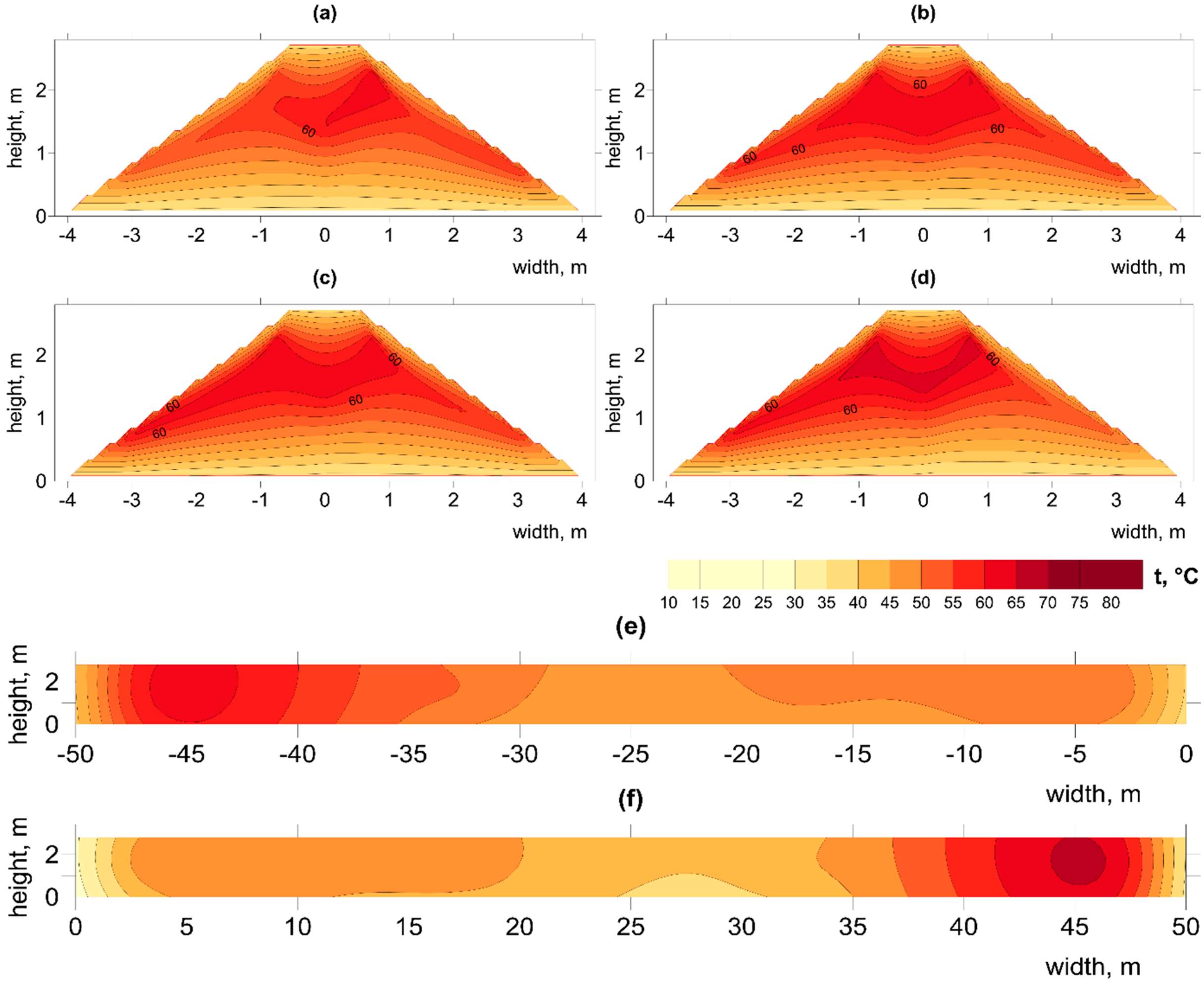

Figure S19. Temperature changes in the pile B1, day 16.

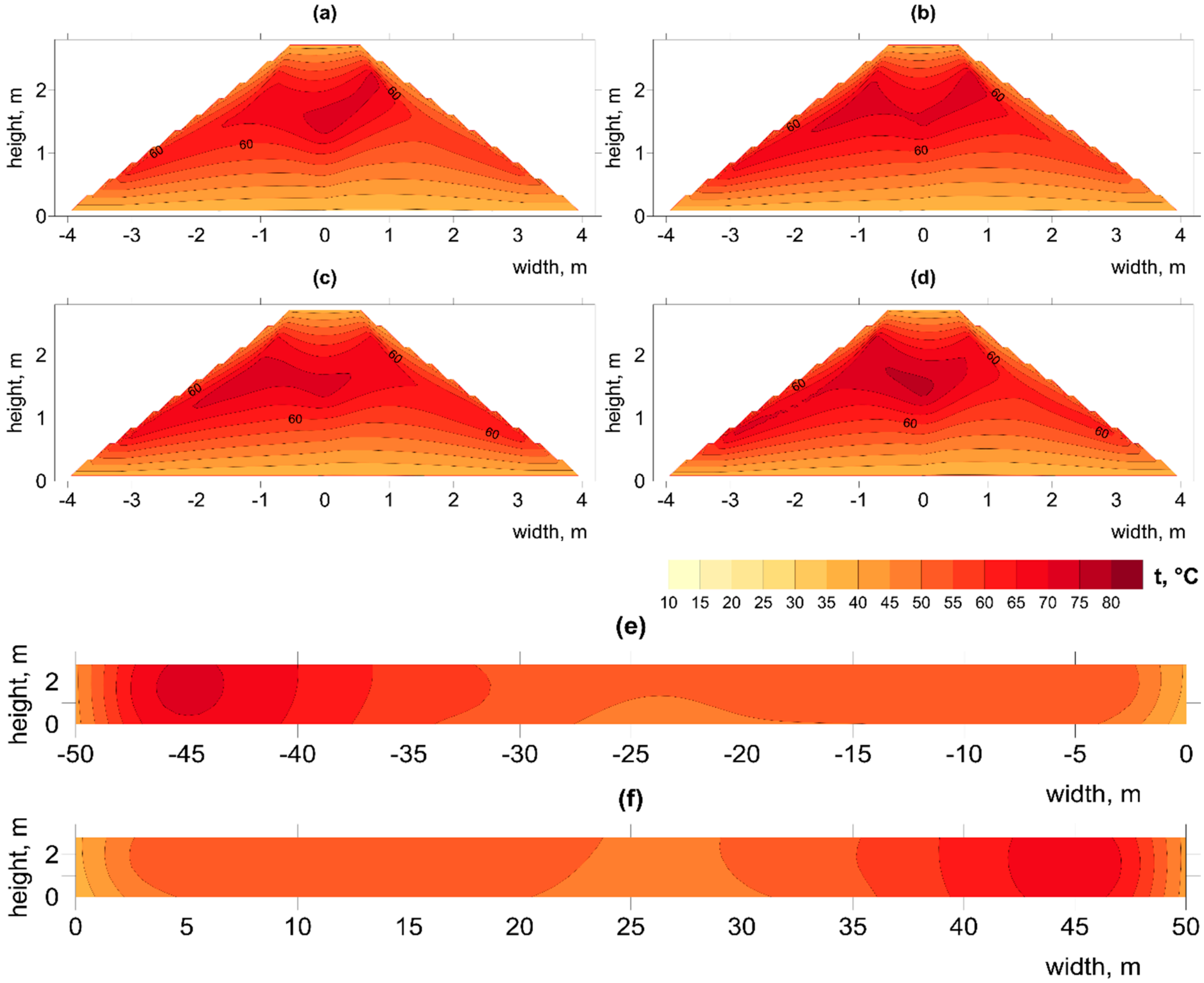

Figure S20. Temperature changes in the pile B1, day 22.

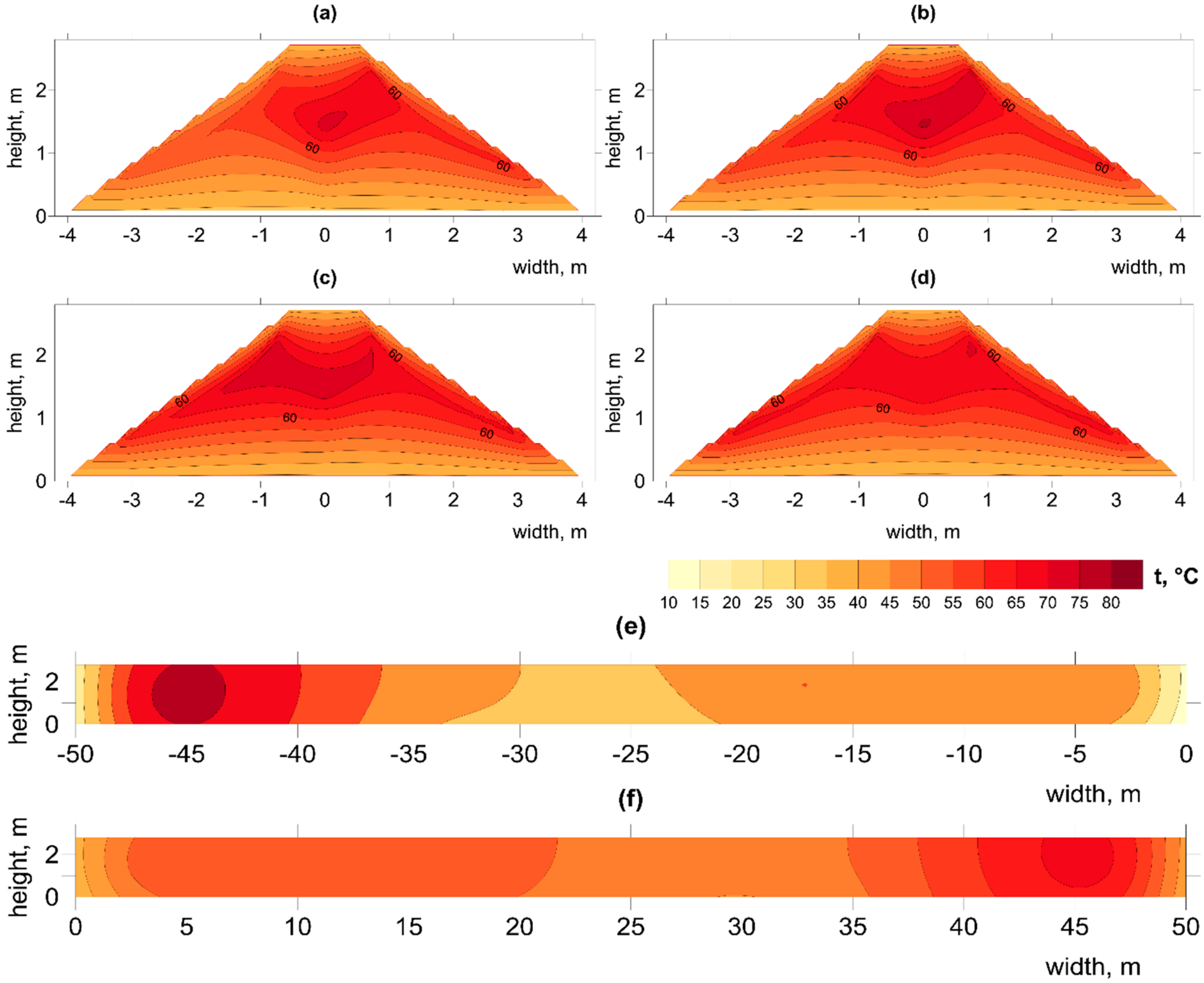

Figure S21. Temperature changes in the pile B1, day 31.

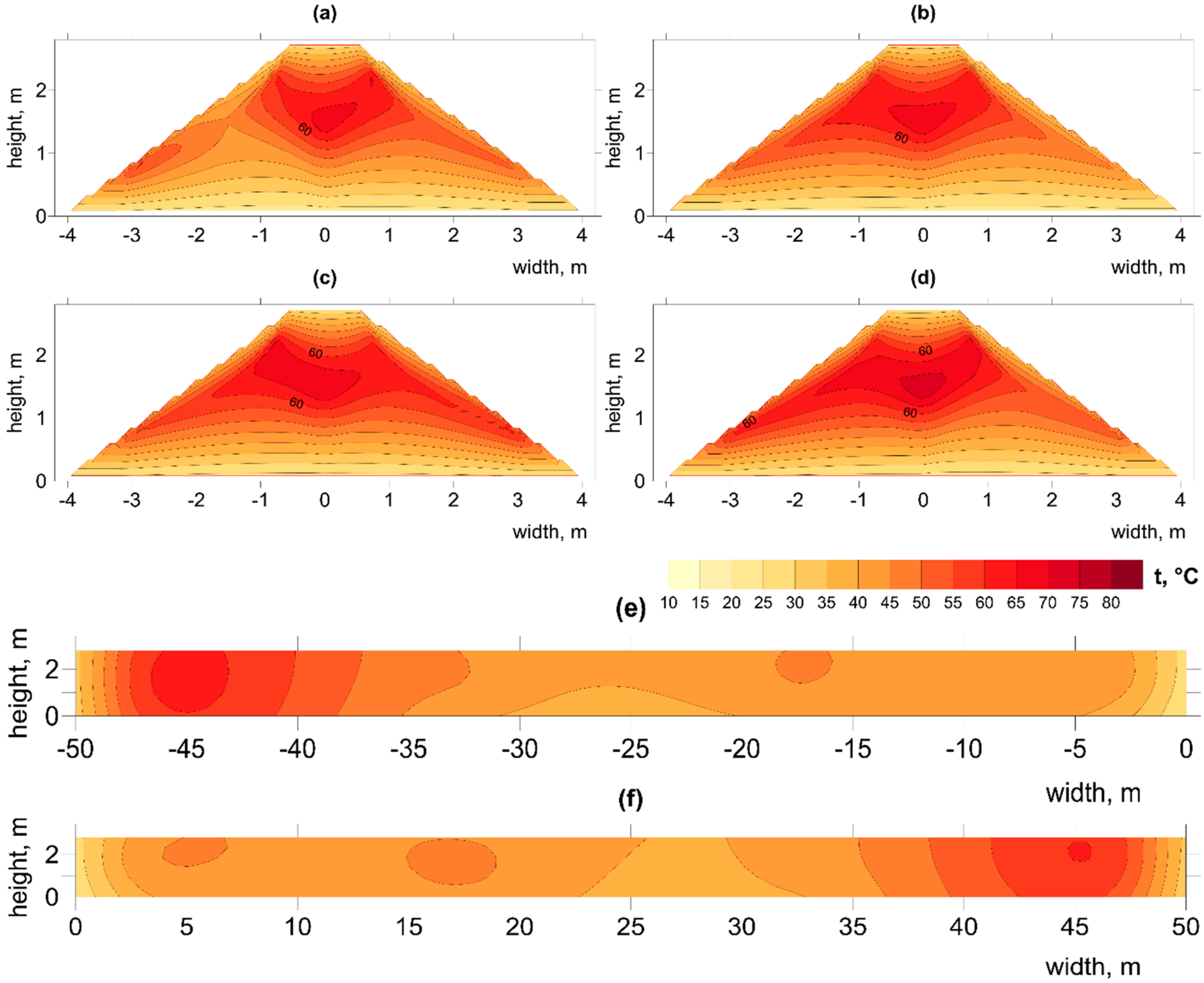

Figure S22. Temperature changes in the pile B1, day 41.

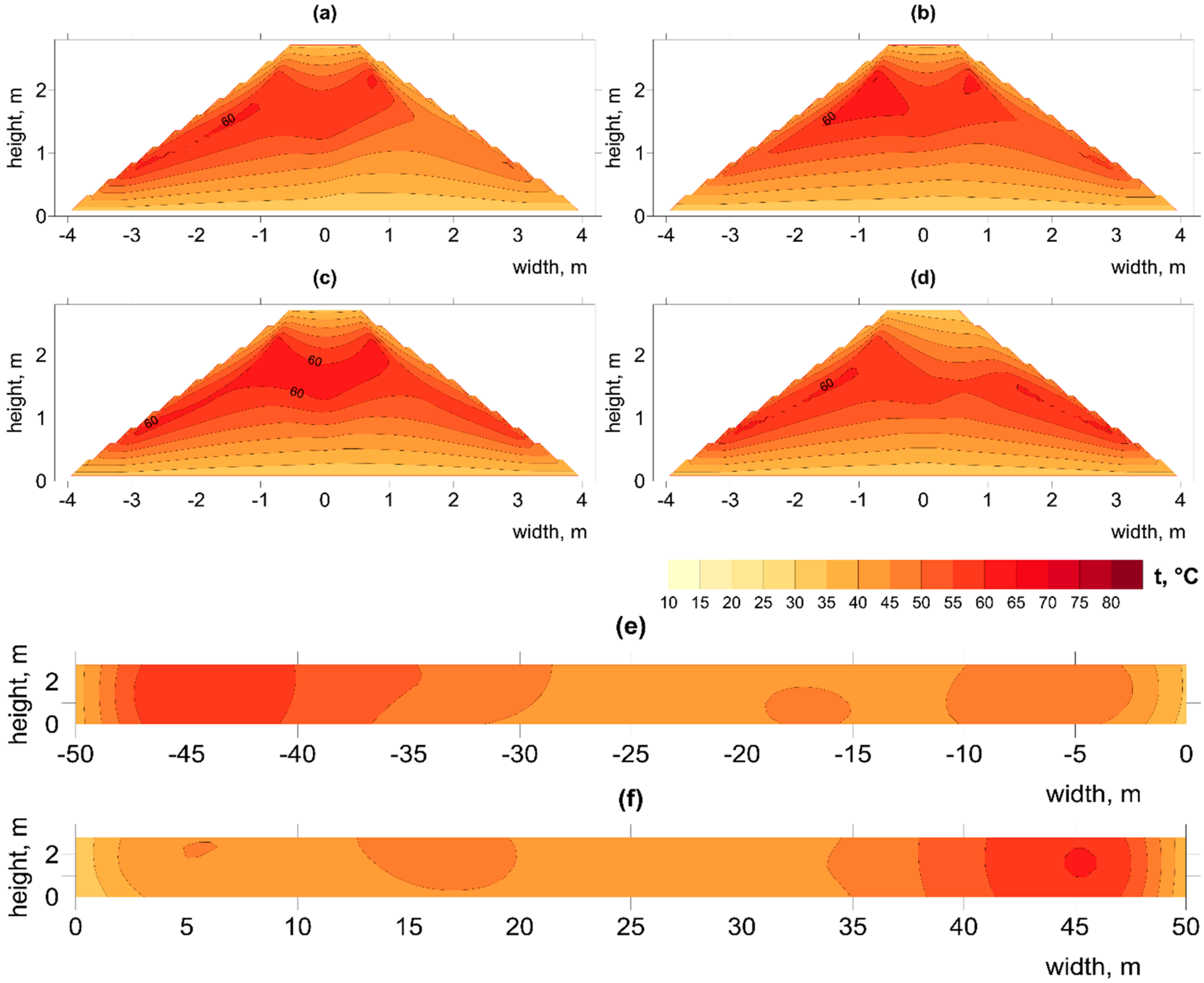

Figure S23. Temperature changes in the pile B1, day 57.

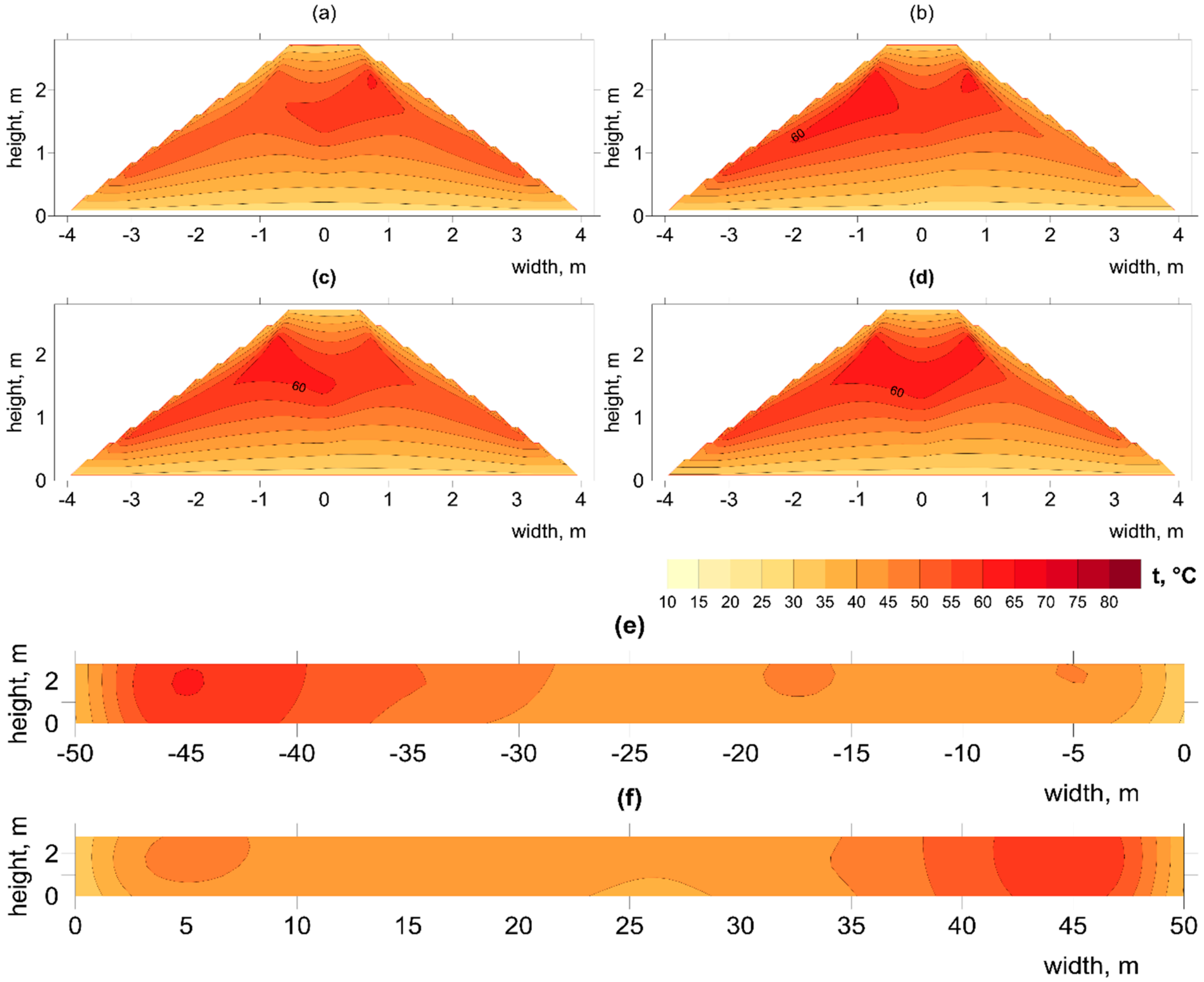

Figure S24. Temperature changes in the pile B1, day 64.

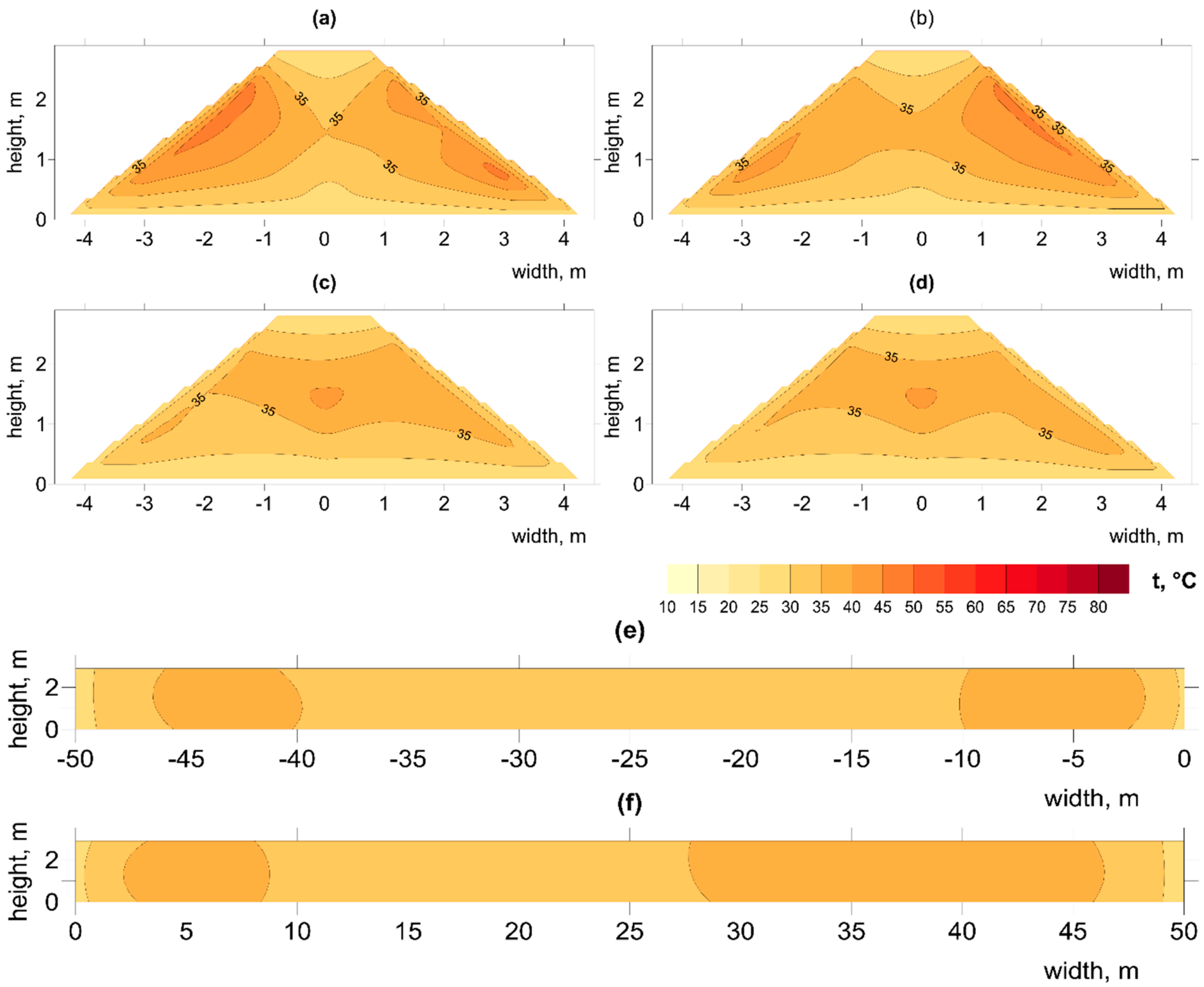

Figure S25. Temperature changes in the pile B2, day 1.

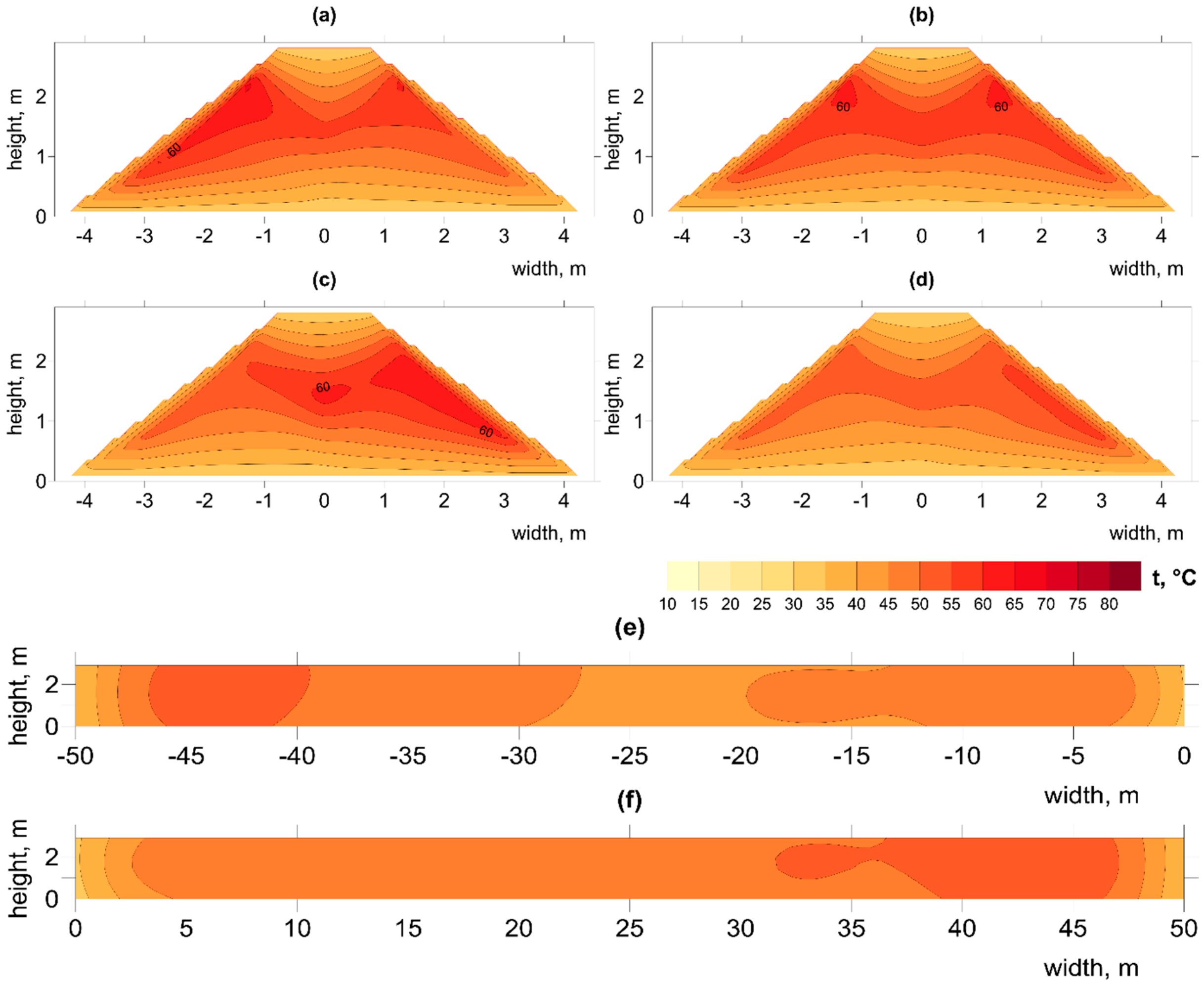

Figure S26. Temperature changes in the pile B2, day 8.

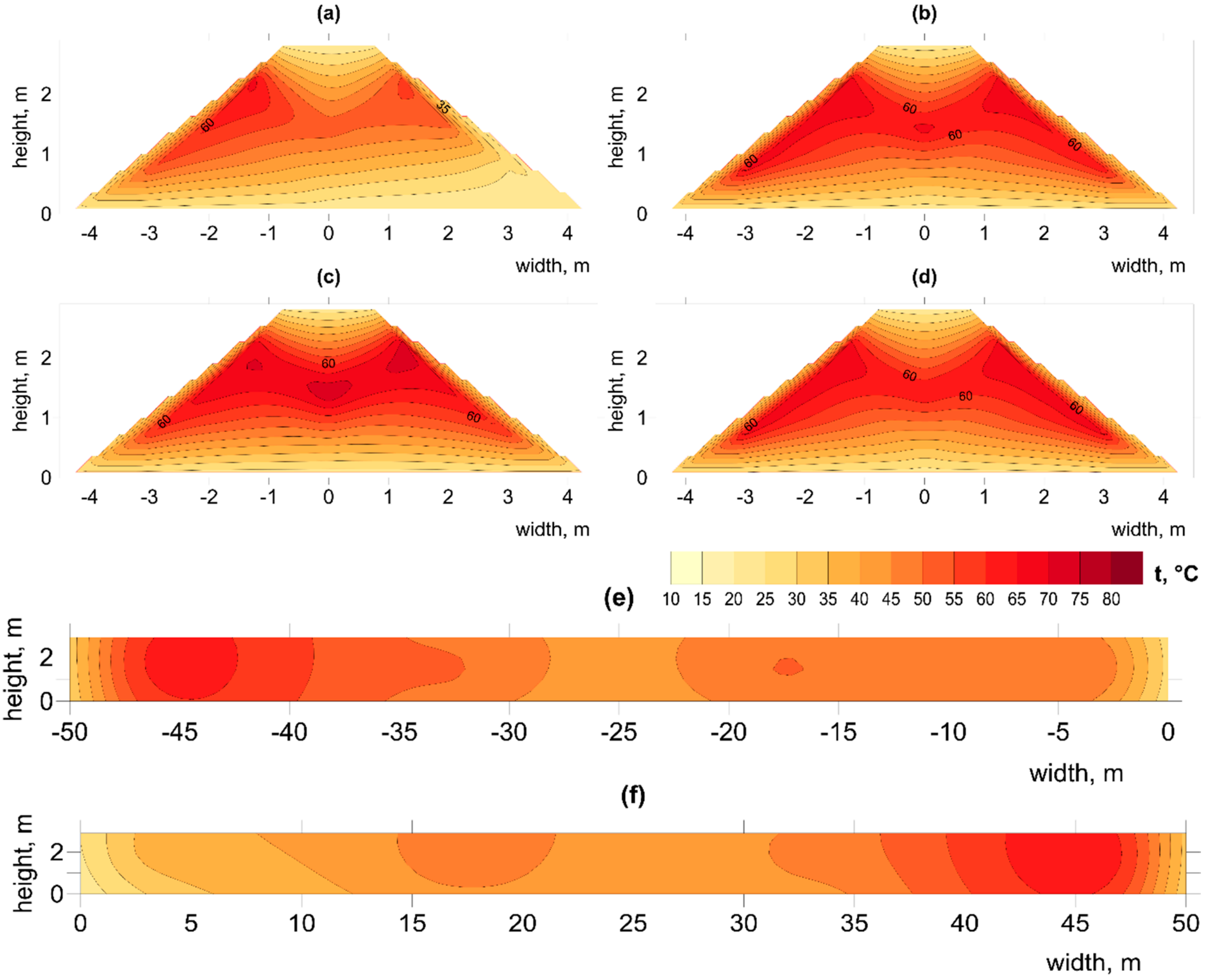

Figure S27. Temperature changes in the pile B2, day 16.

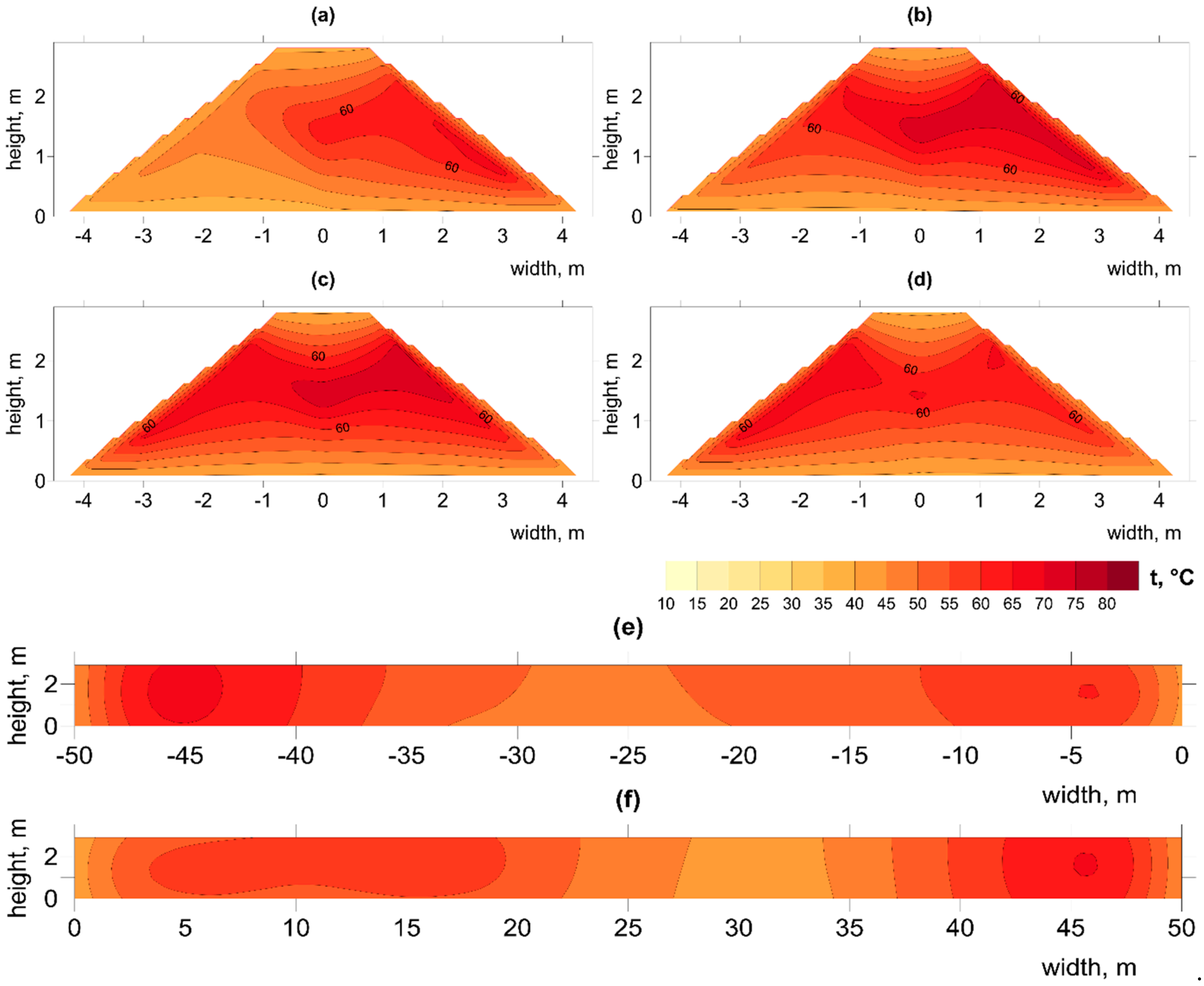

Figure S28. Temperature changes in the pile B2, day 24.

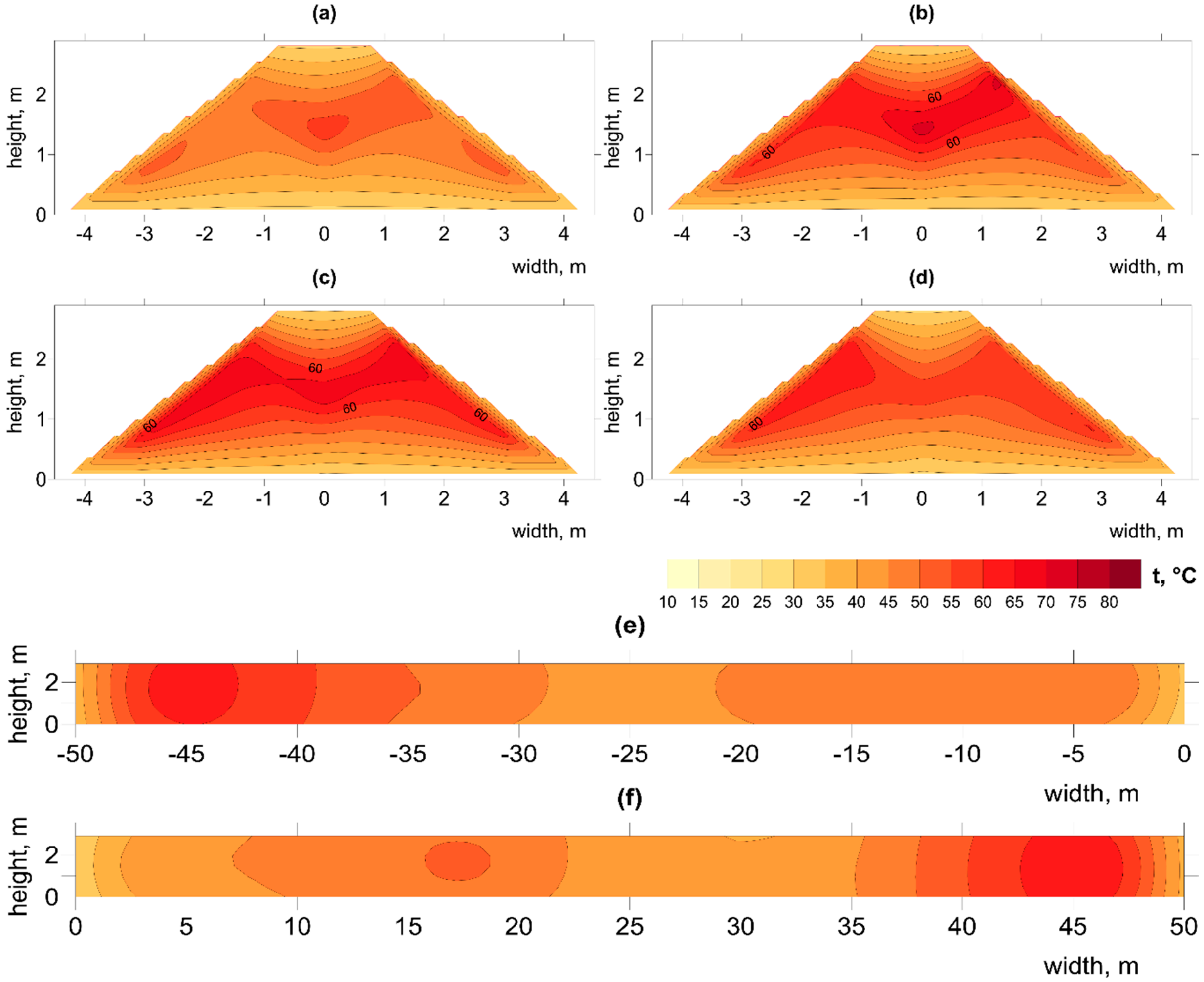

Figure S29. Temperature changes in the pile B2, day 36.

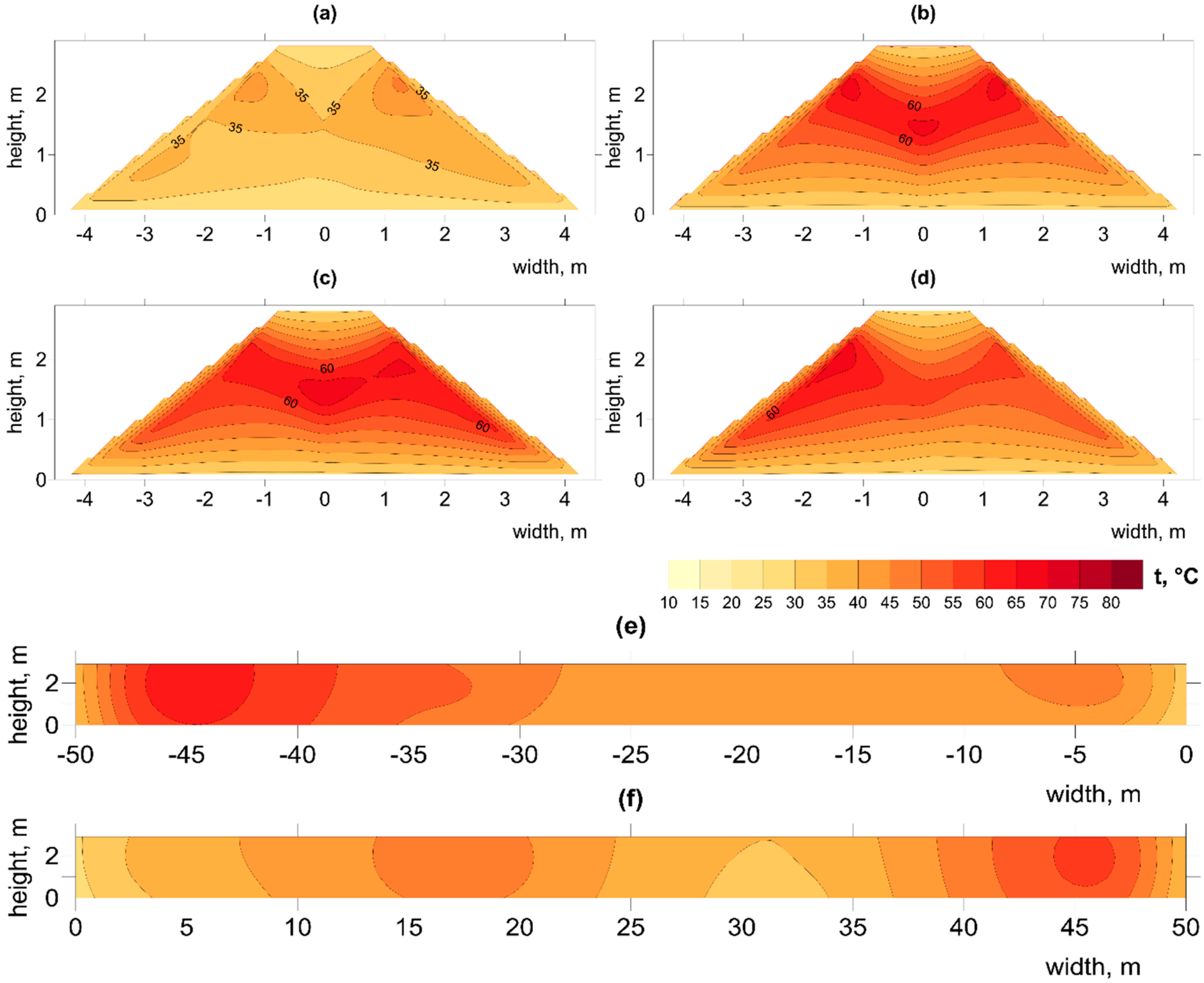

Figure S30. Temperature changes in the pile B2, day 43.

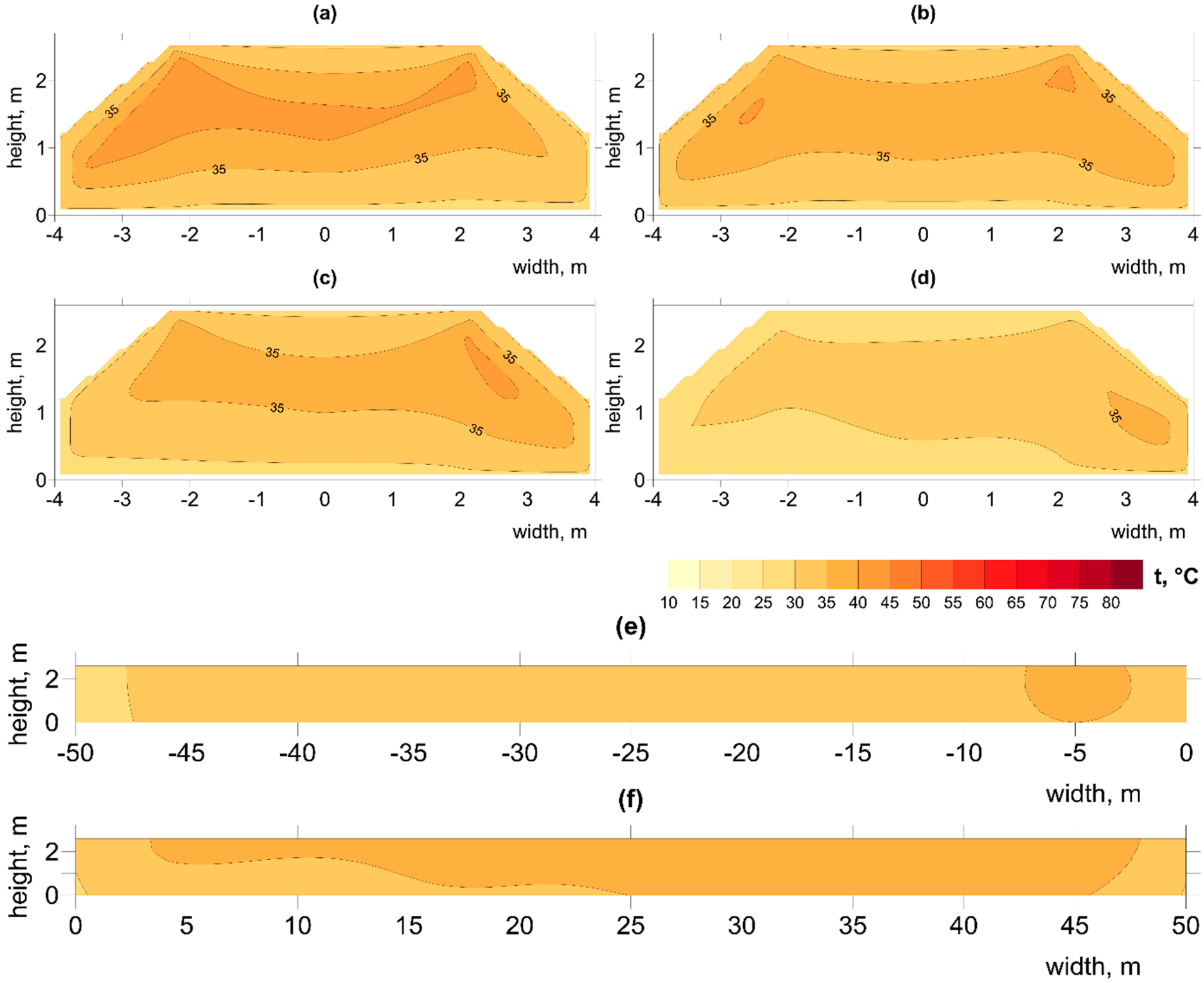

Figure S31. Temperature changes in the pile C1, day 1.

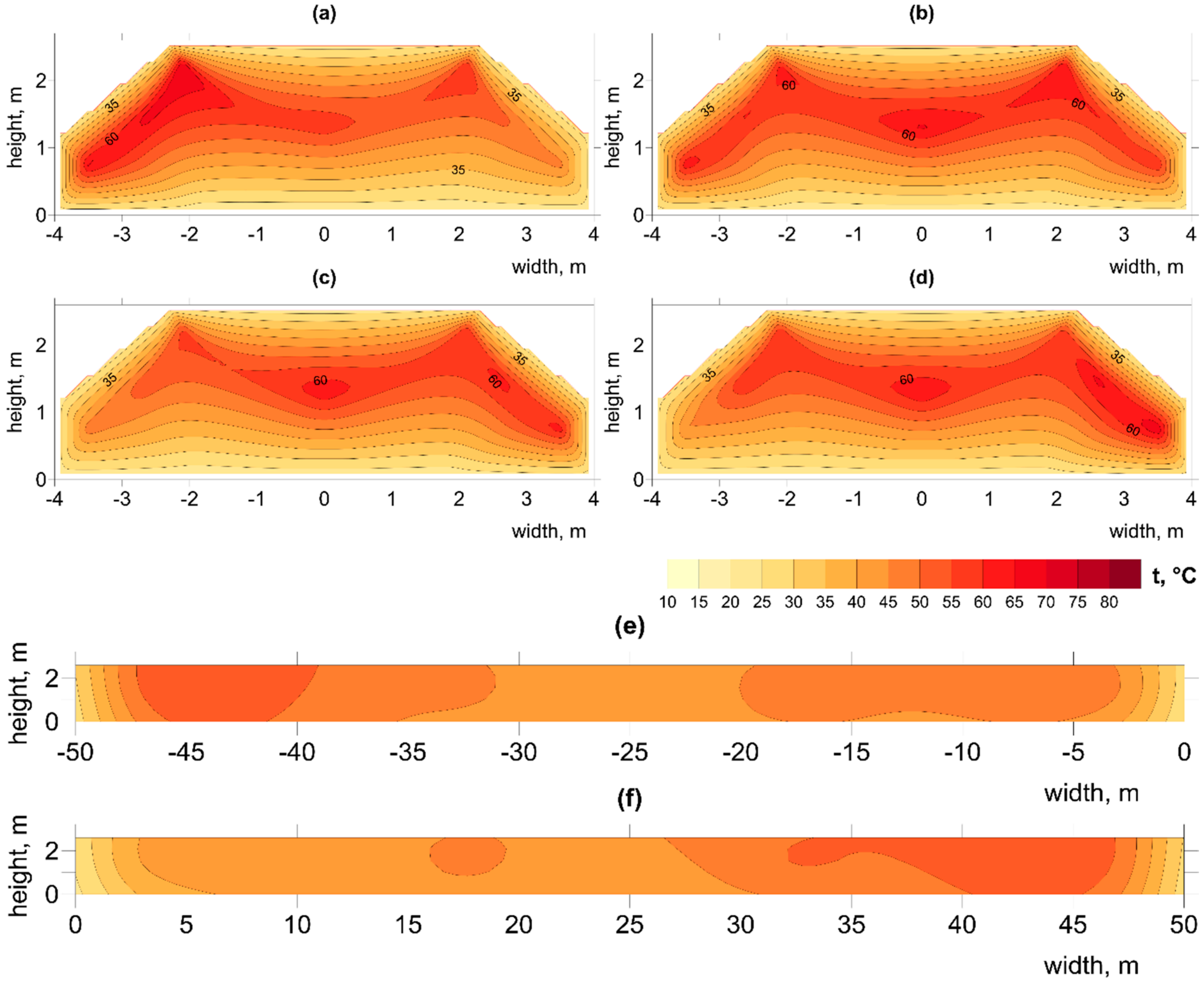

Figure S32. Temperature changes in the pile C1, day 9.

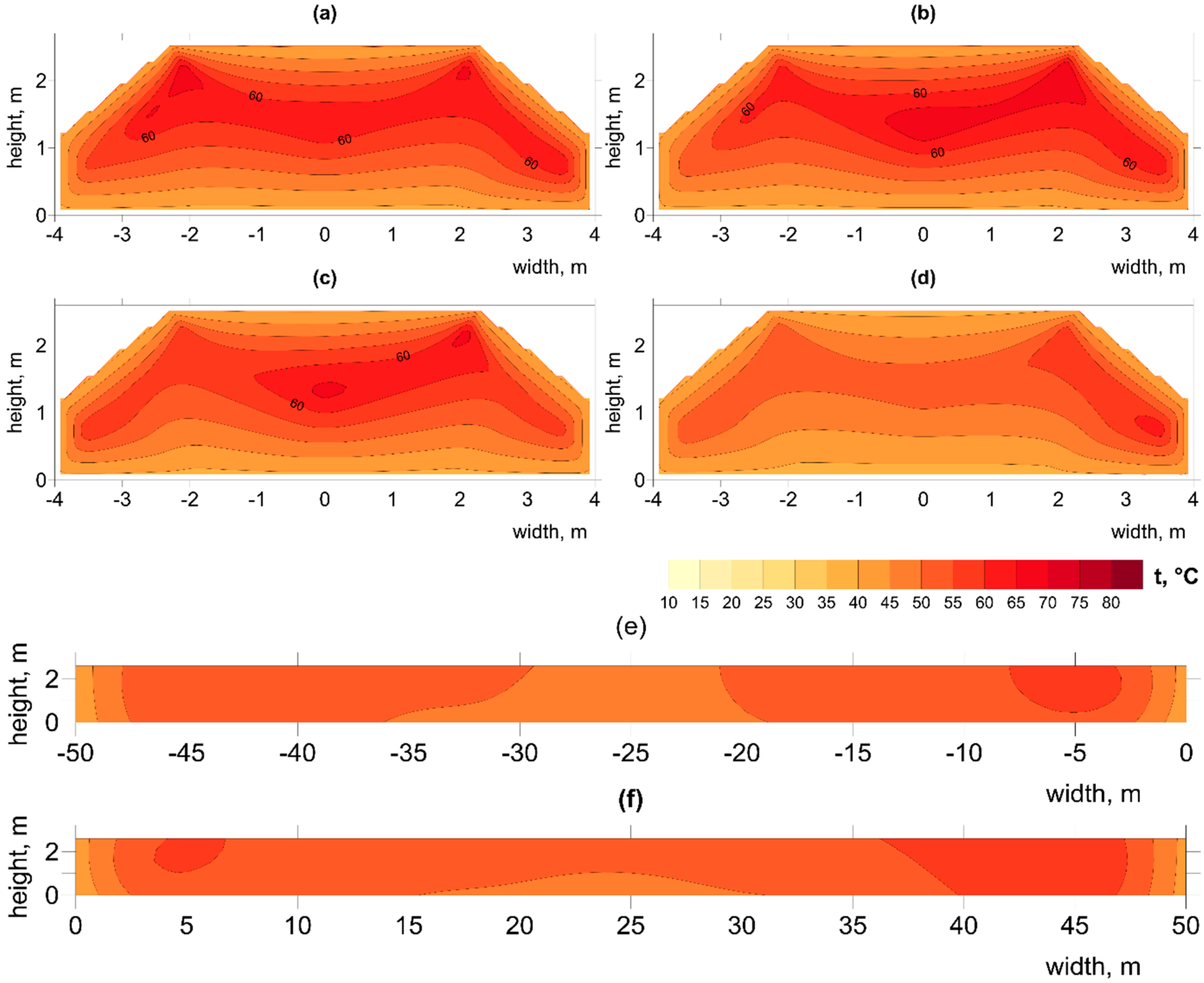

Figure S33. Temperature changes in the pile C1, day 16.

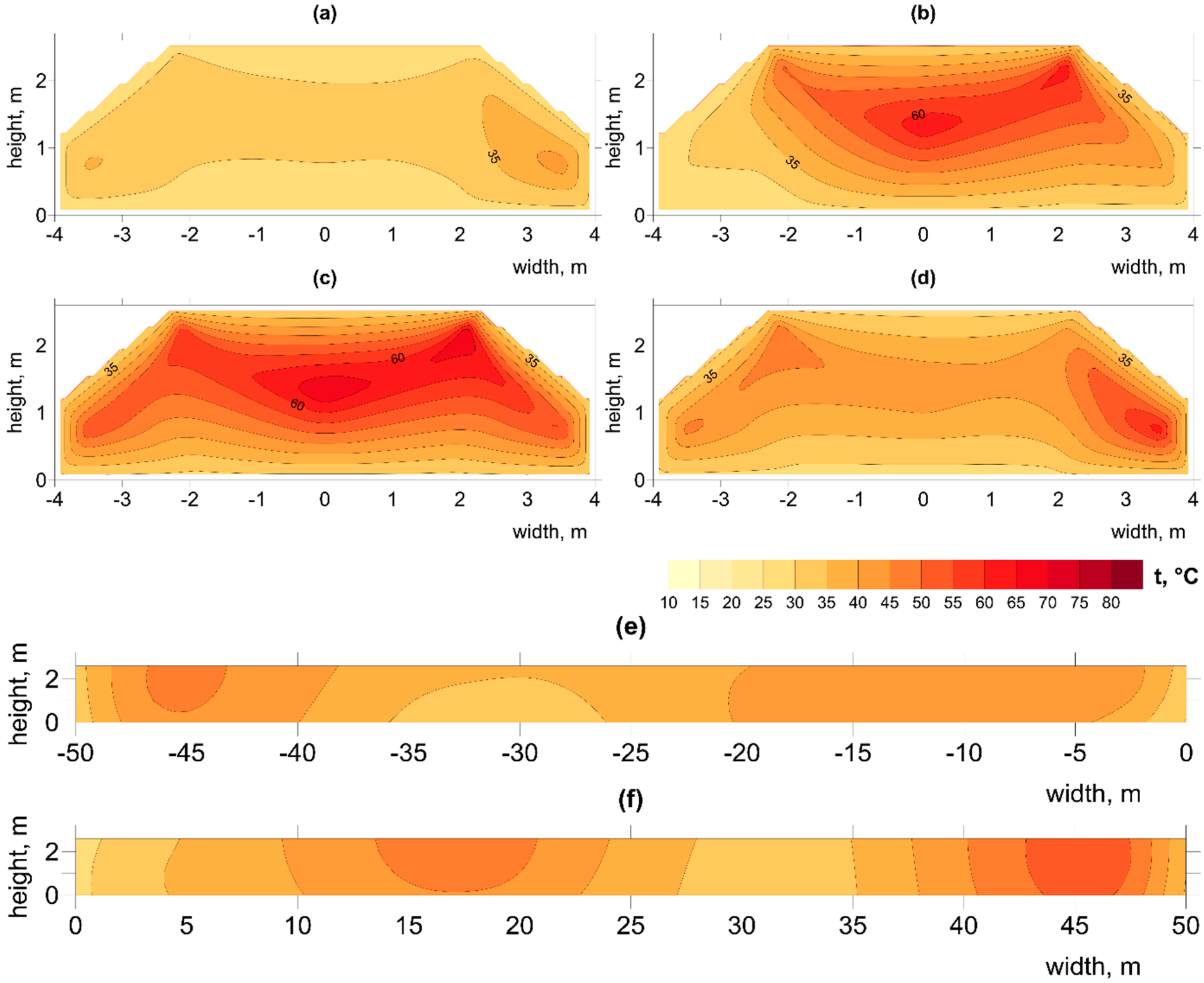

Figure S34. Temperature changes in the pile C1, day 28.

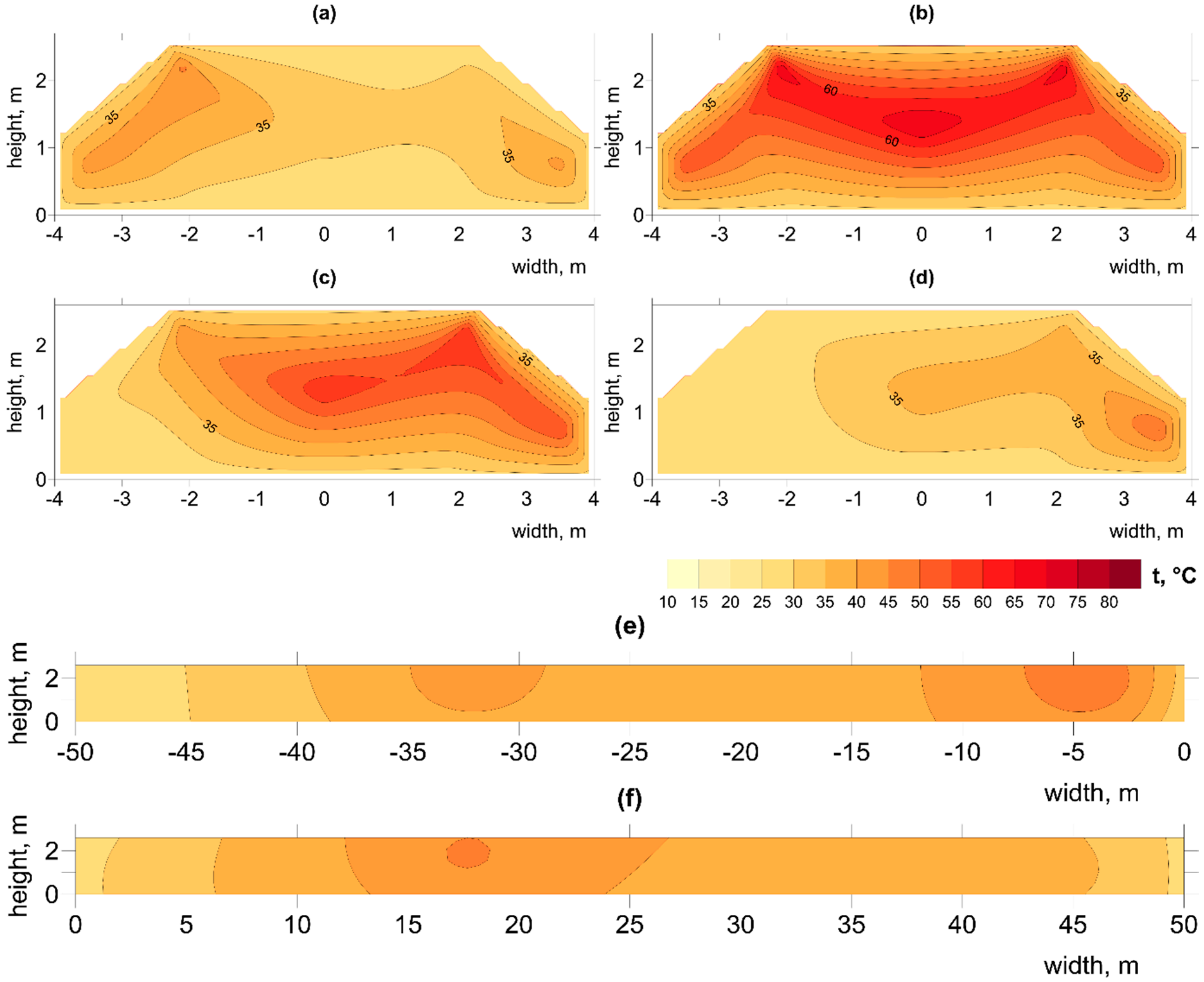

Figure S35. Temperature changes in the pile C1, day 35.

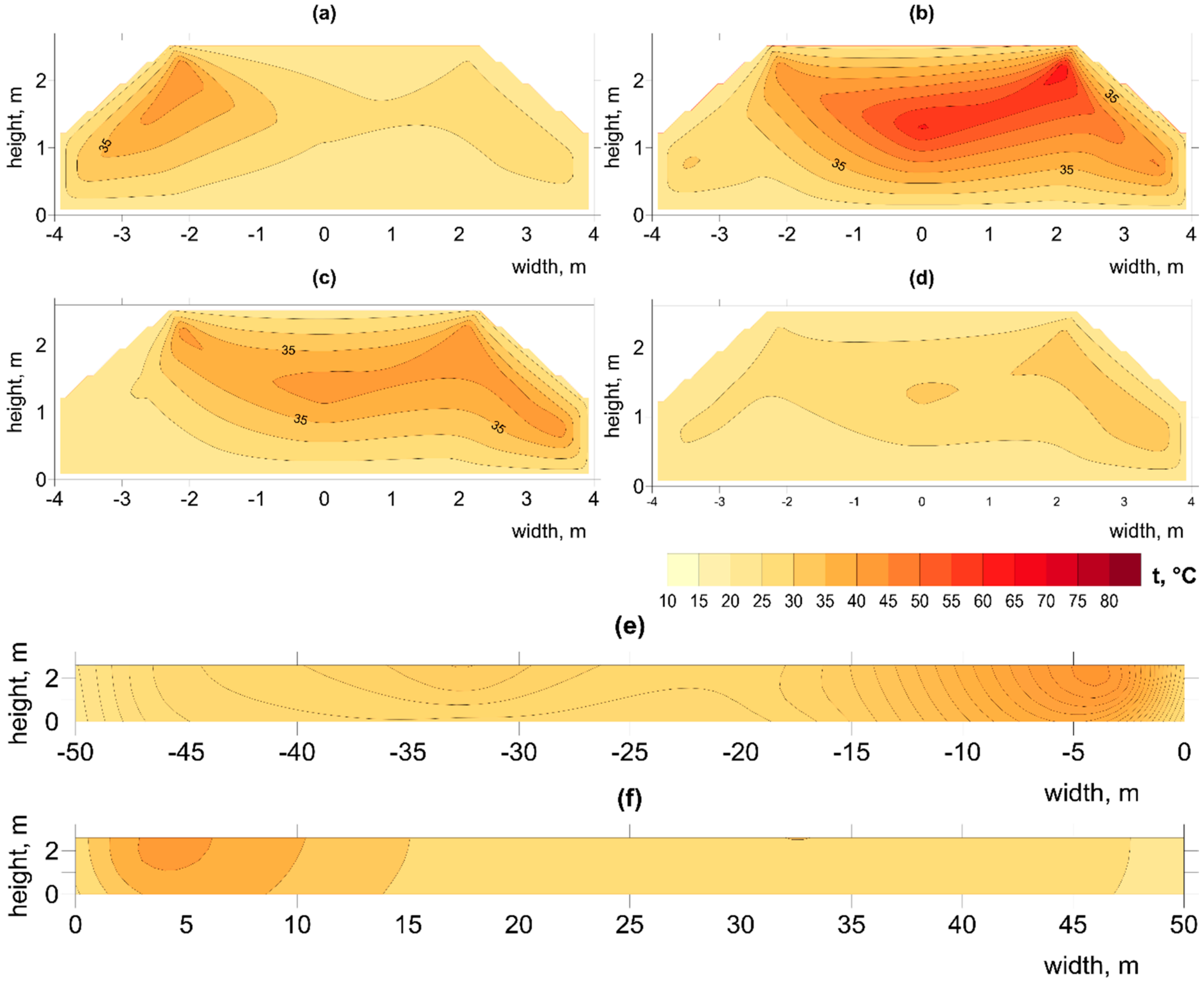

Figure S36. Temperature changes in the pile C1, day 42.

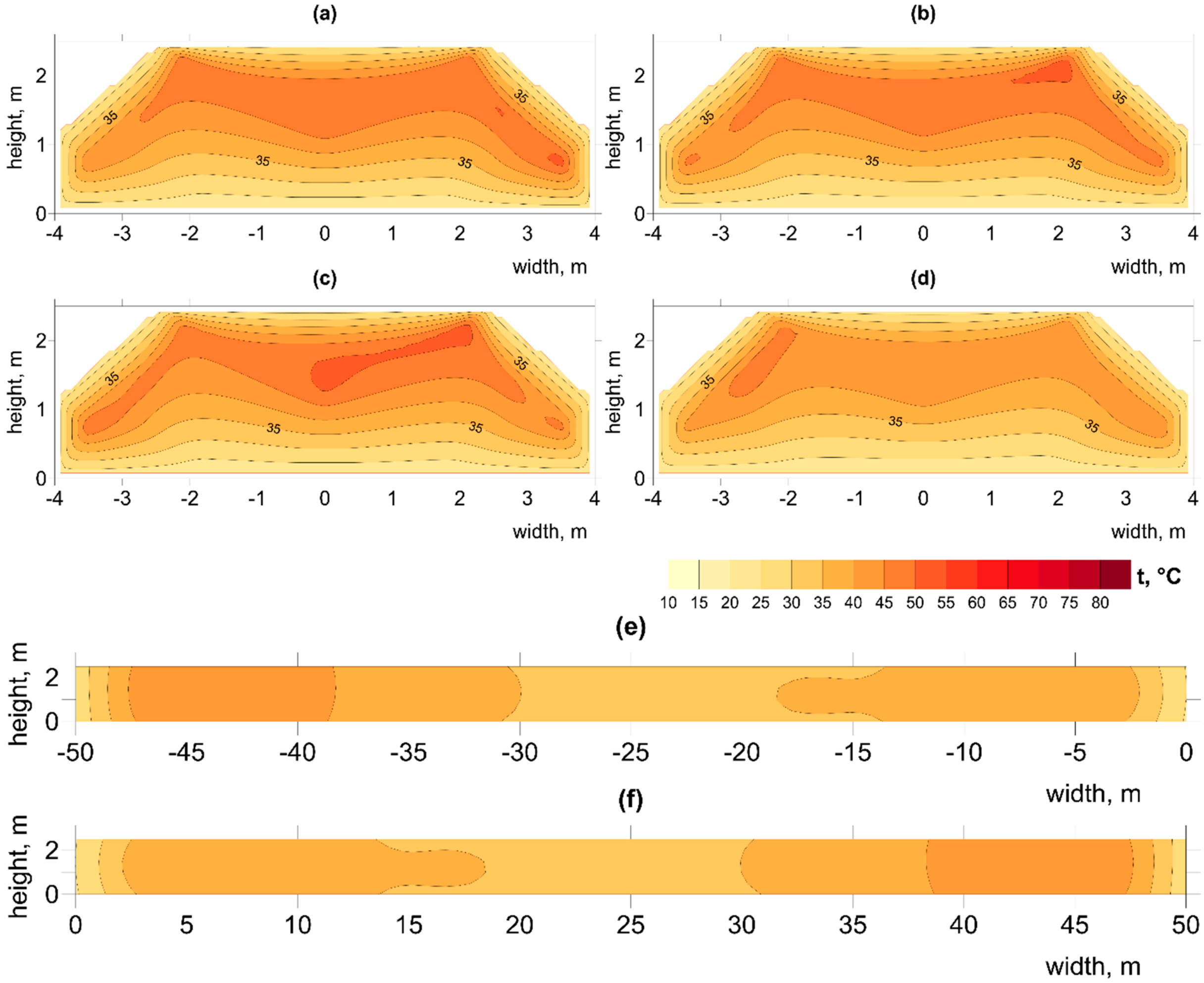

Figure S37. Temperature changes in the pile C2, day 1.

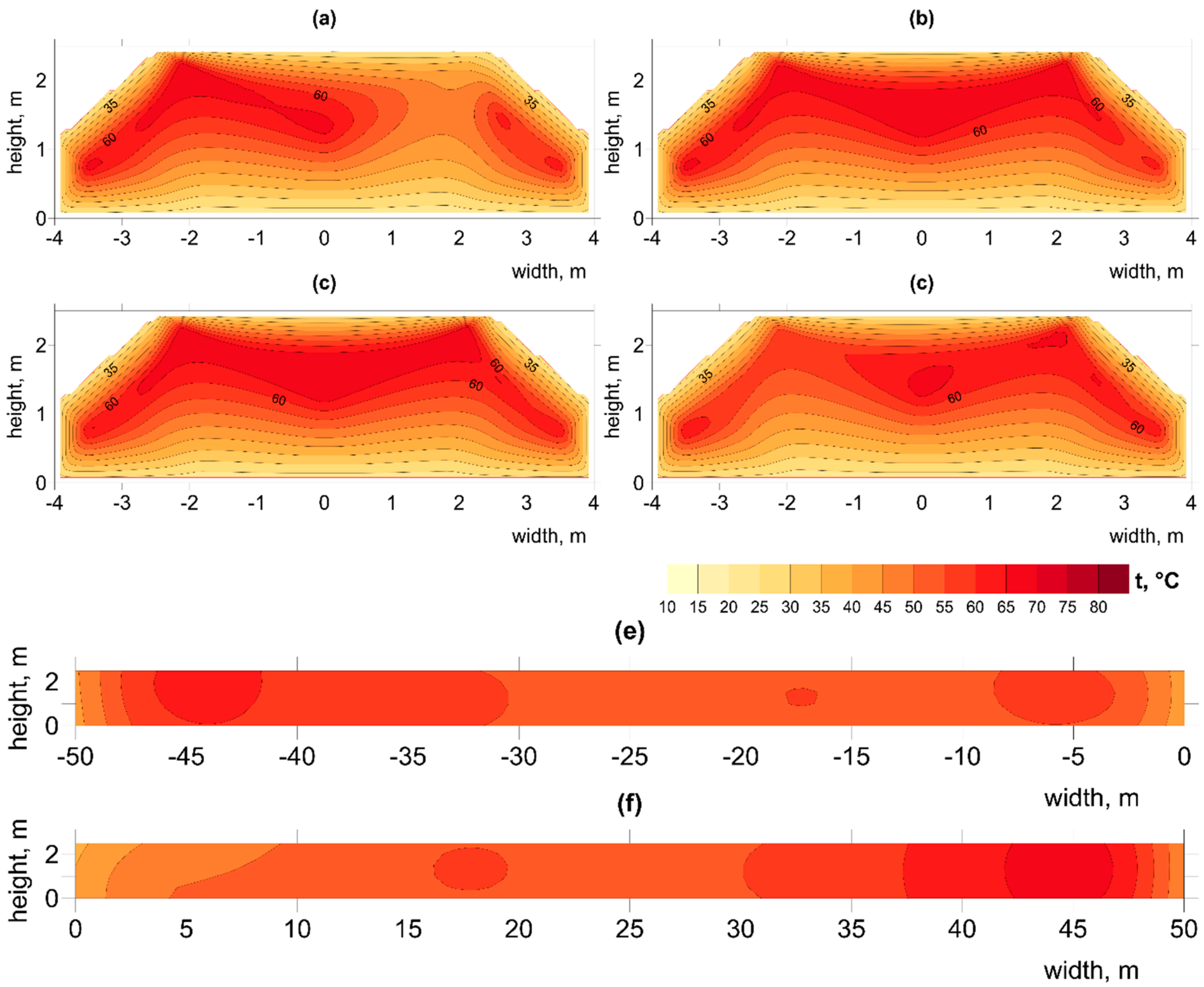

Figure S38. Temperature changes in the pile C2, day 9.

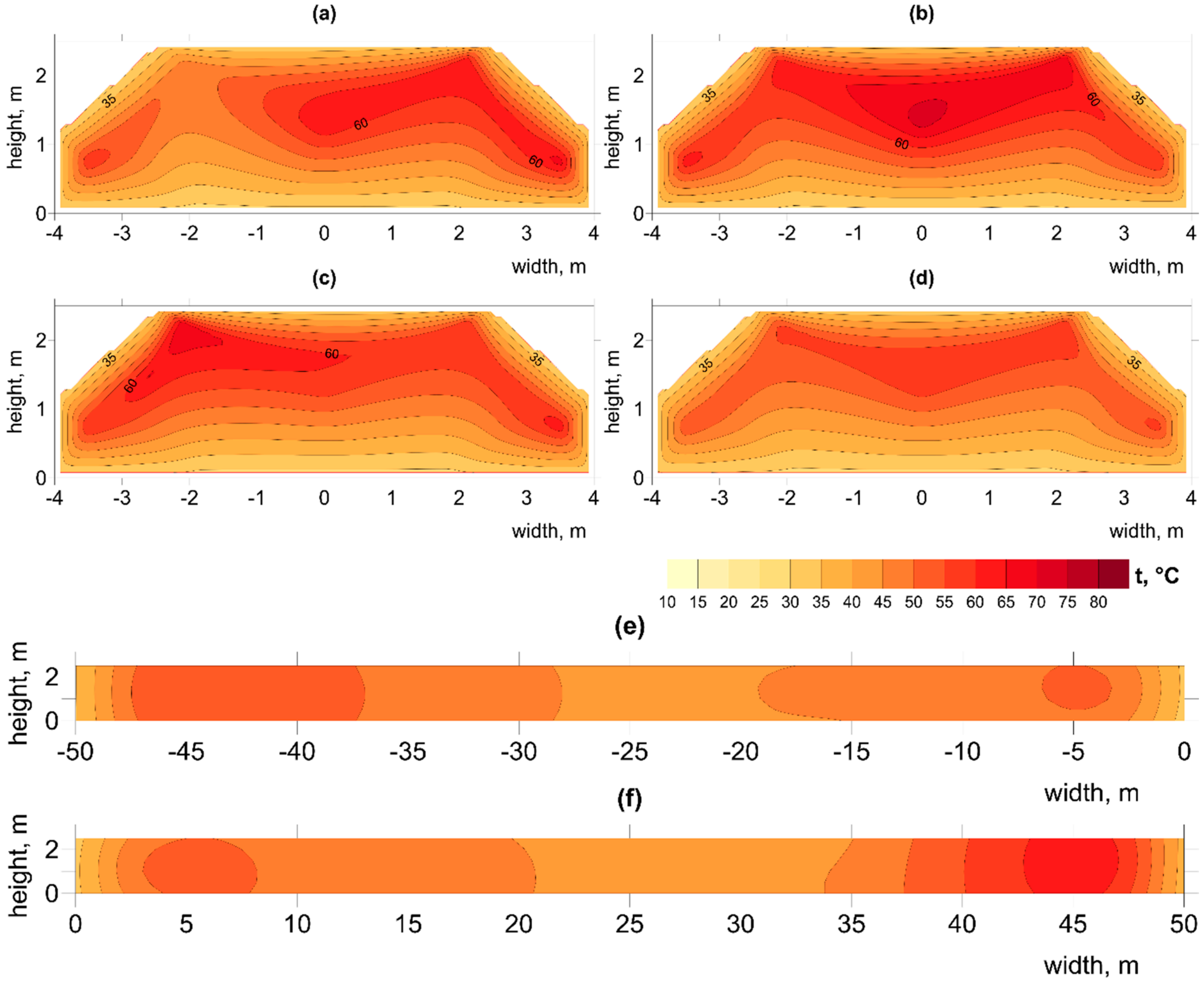

Figure S39. Temperature changes in the pile C2, day 21.

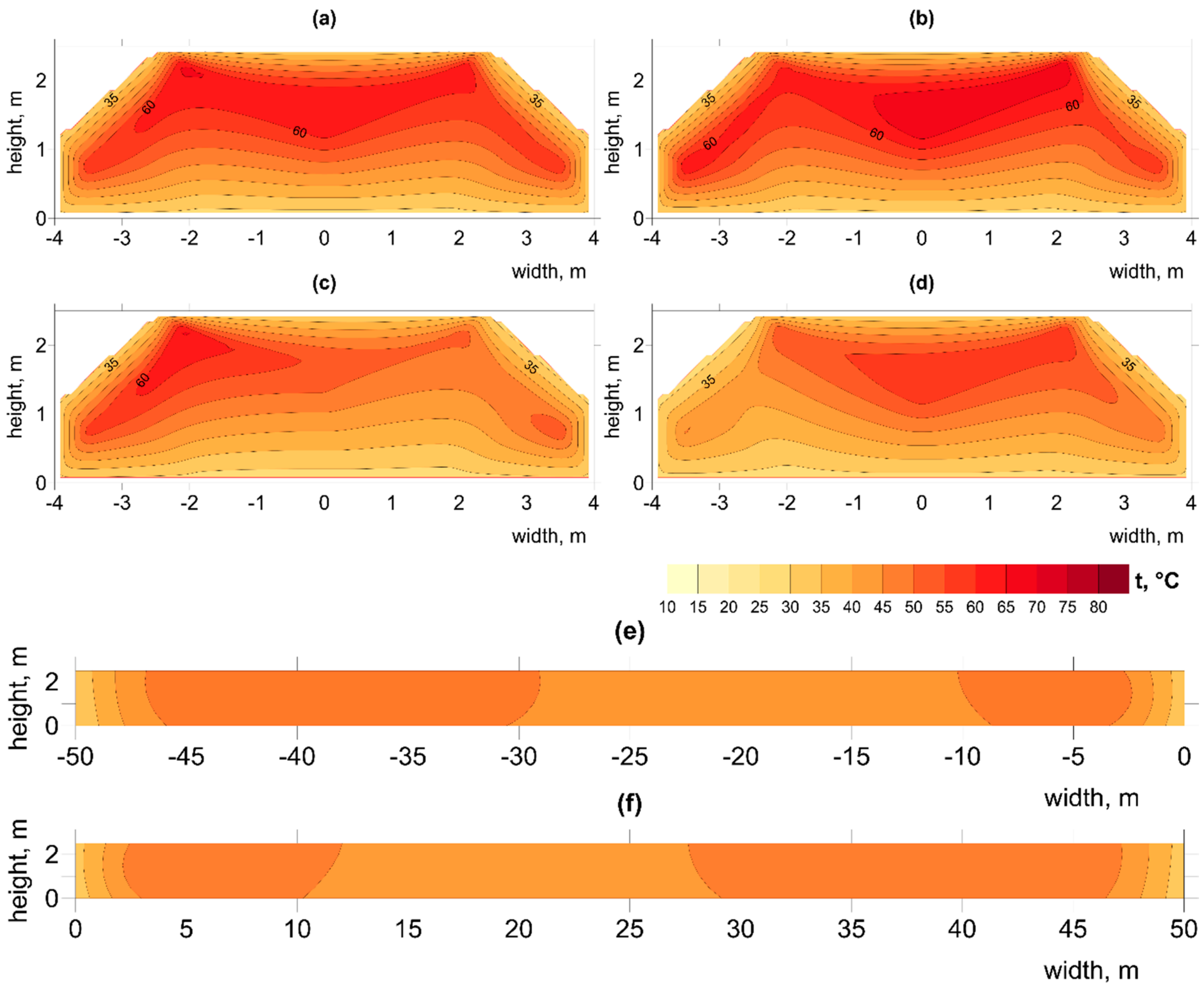

Figure S40. Temperature changes in the pile C2, day 28.

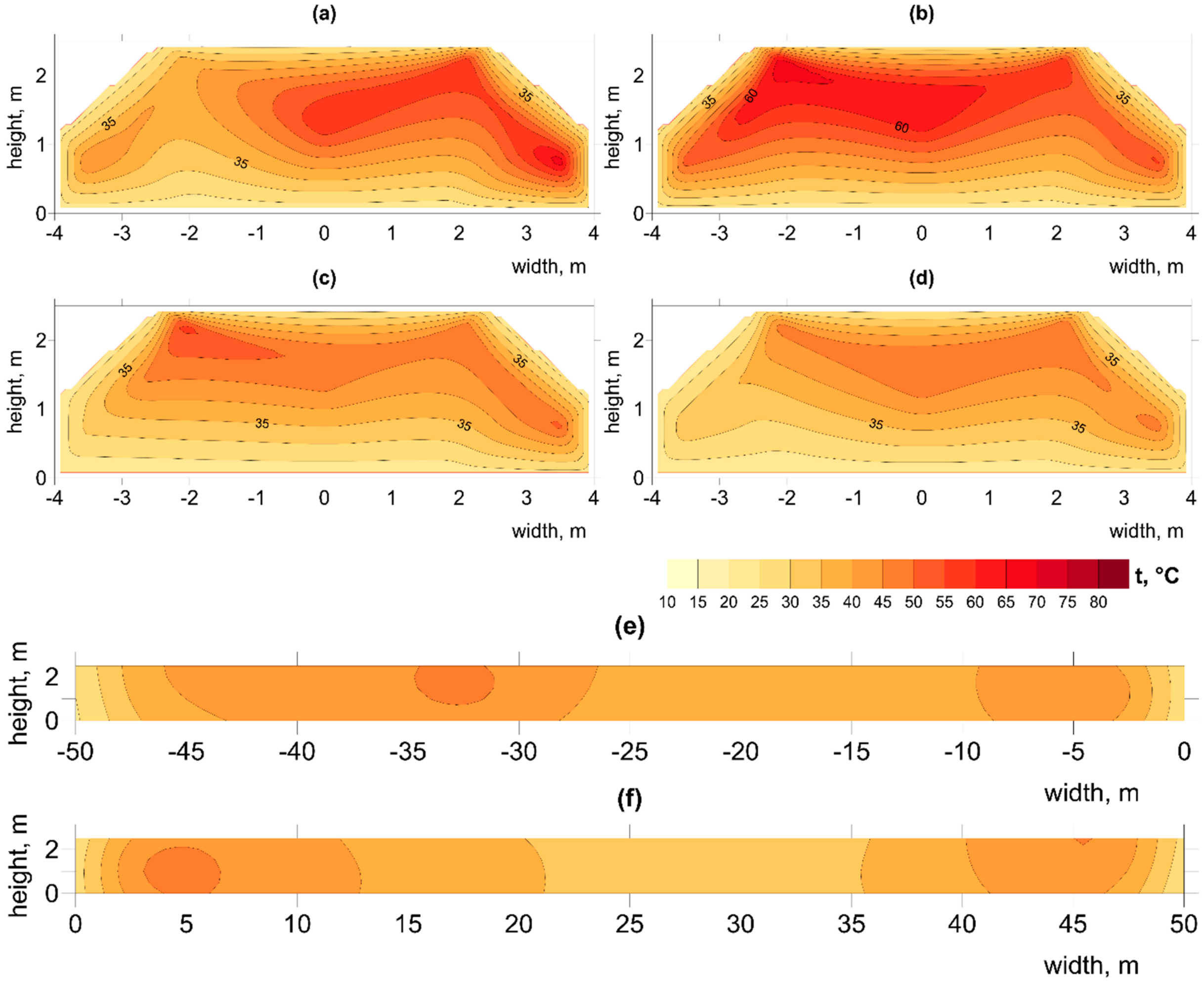

Figure S41. Temperature changes in the pile C2, day 35.

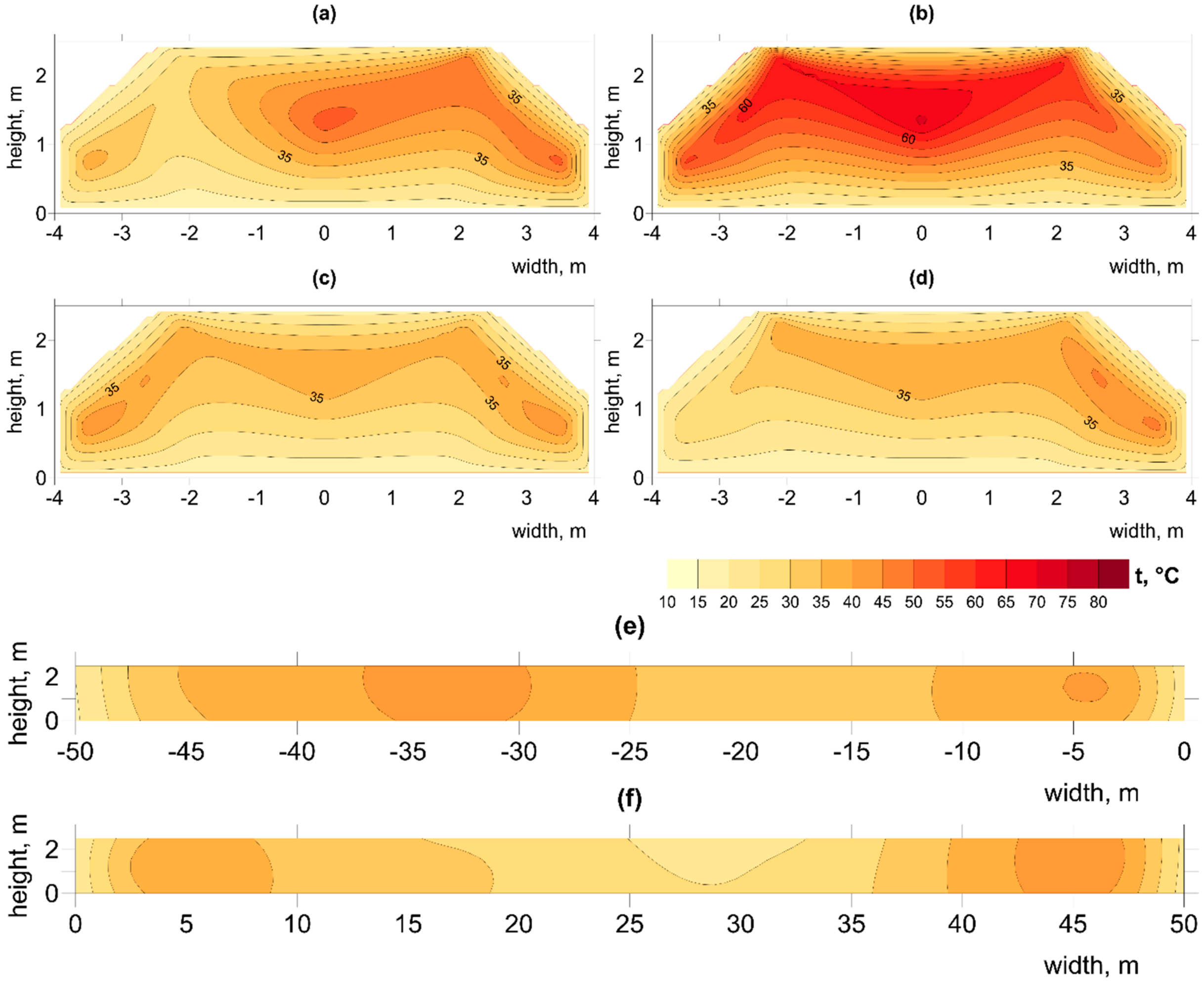

Figure S42. Temperature changes in the pile C2, day 42.

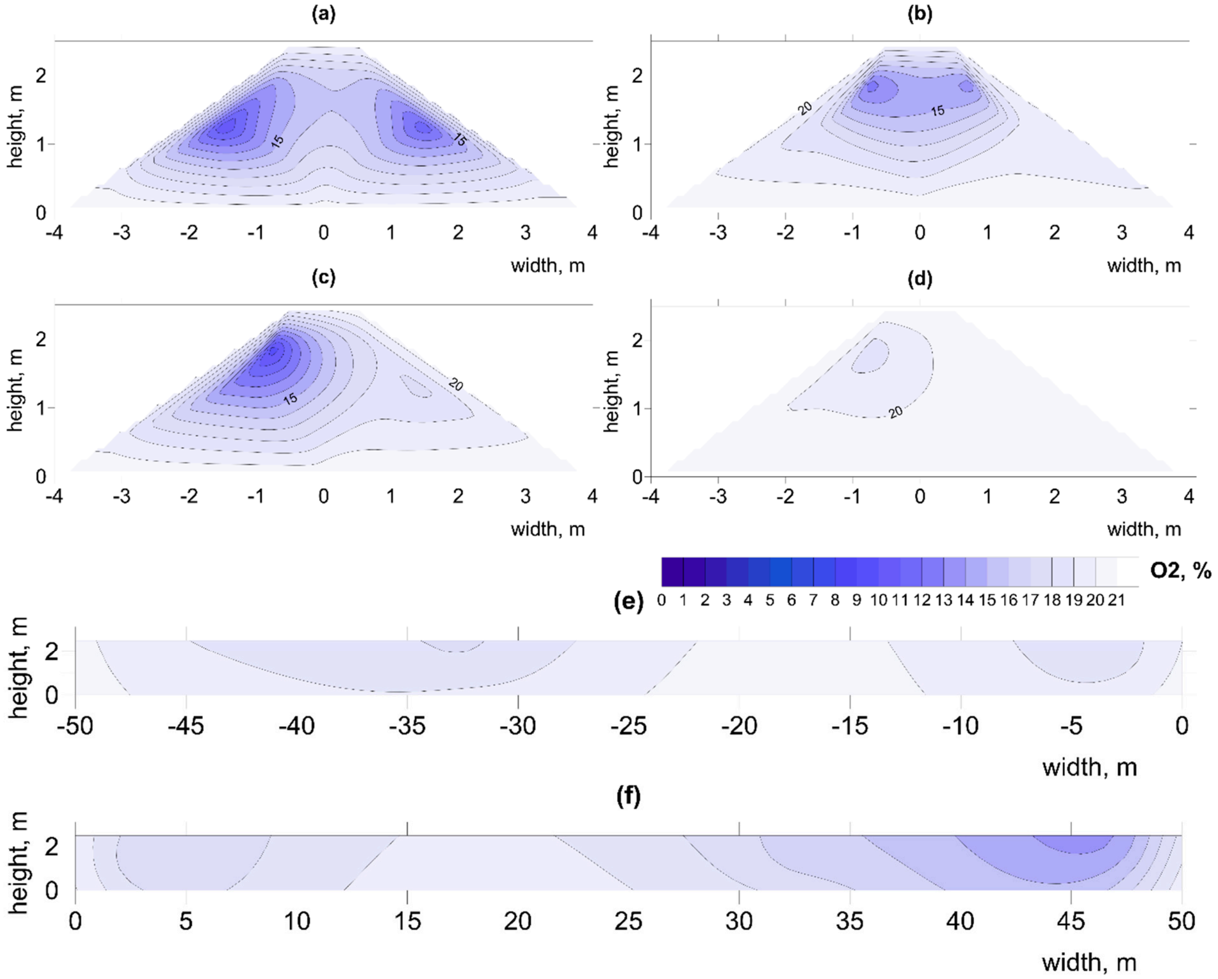

Figure S43. O2 distribution in the pile A1, day 1.

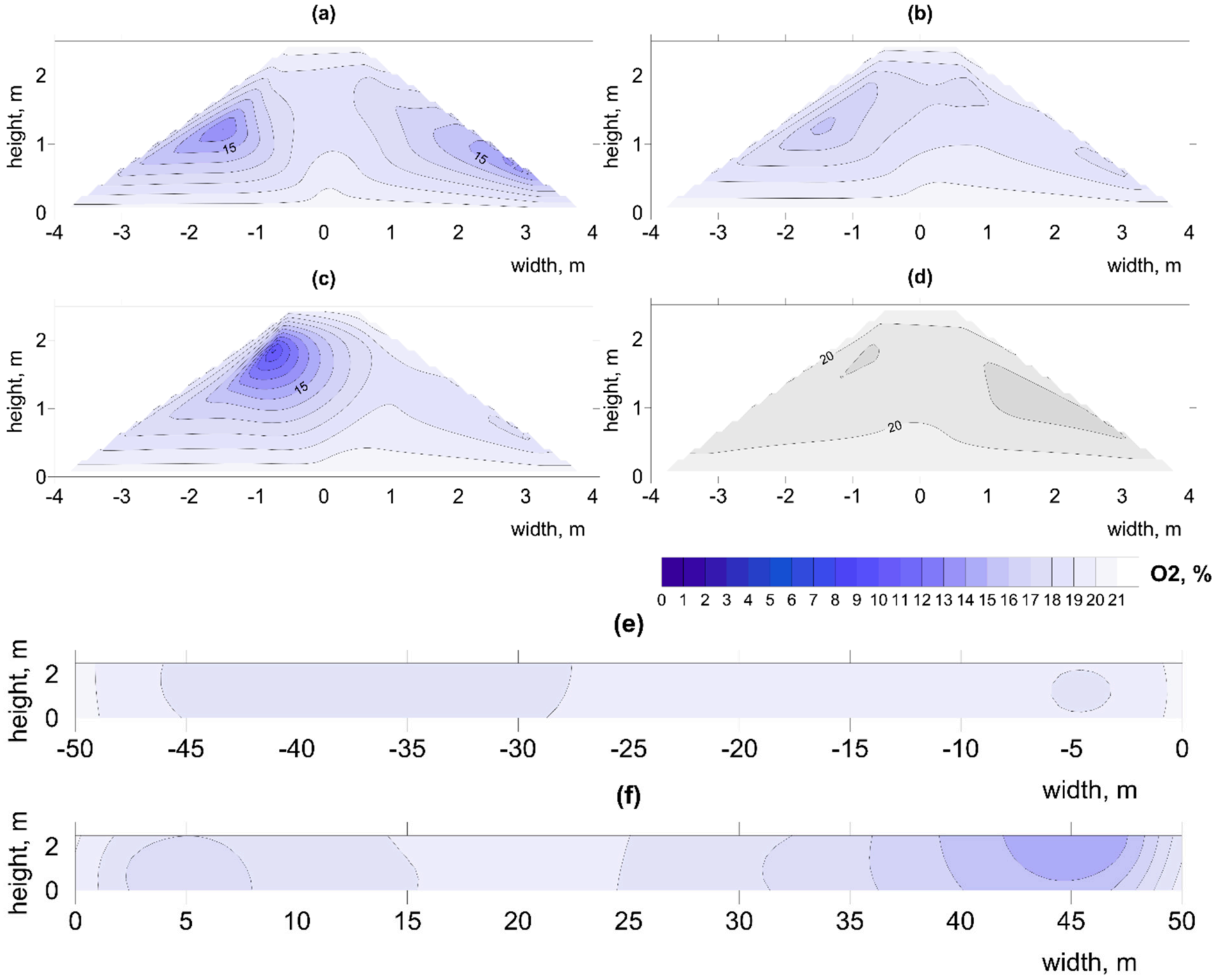

Figure S44. O2 distribution in the pile A1, day 4.

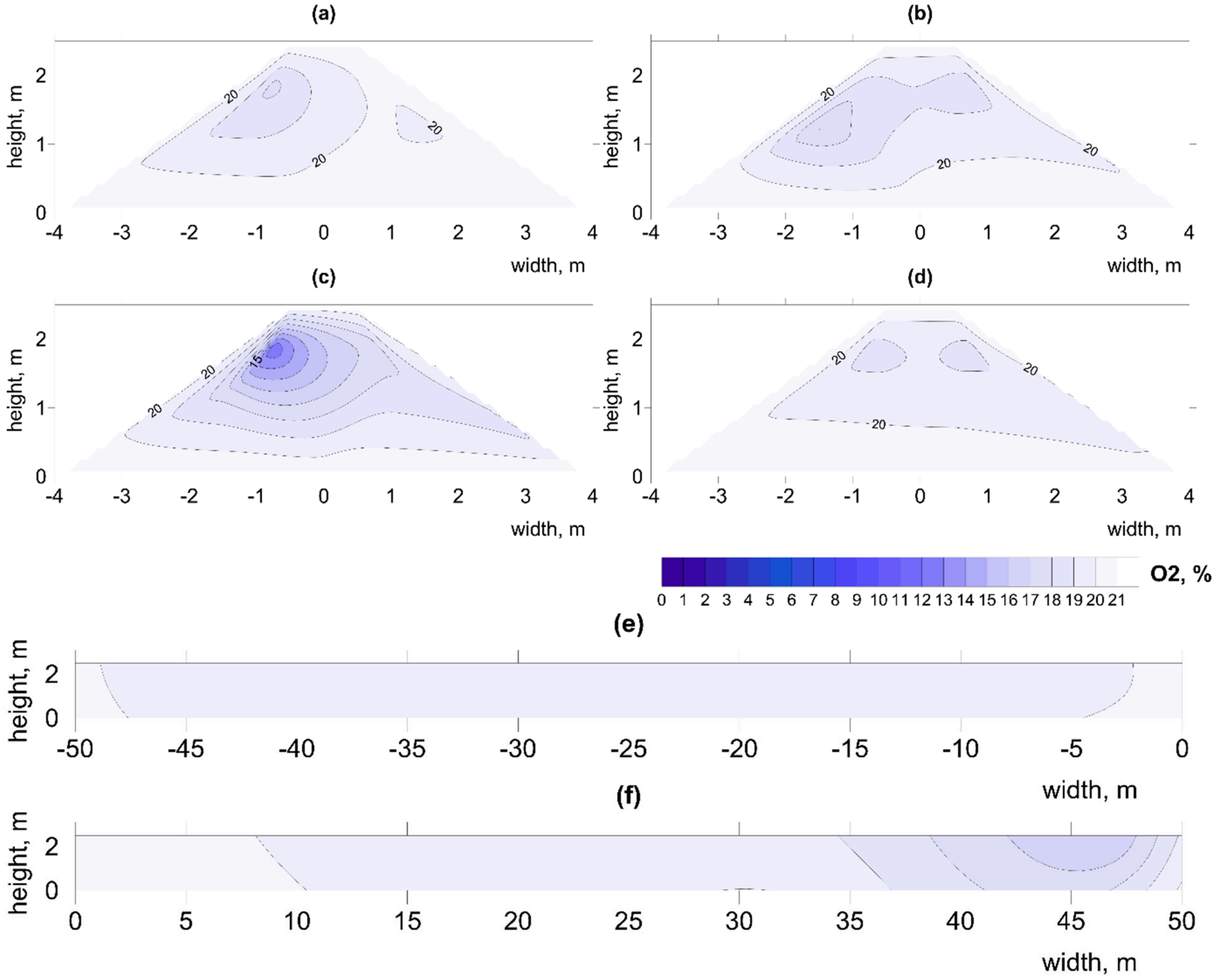

Figure S45. O2 distribution in the pile A1, day 14.

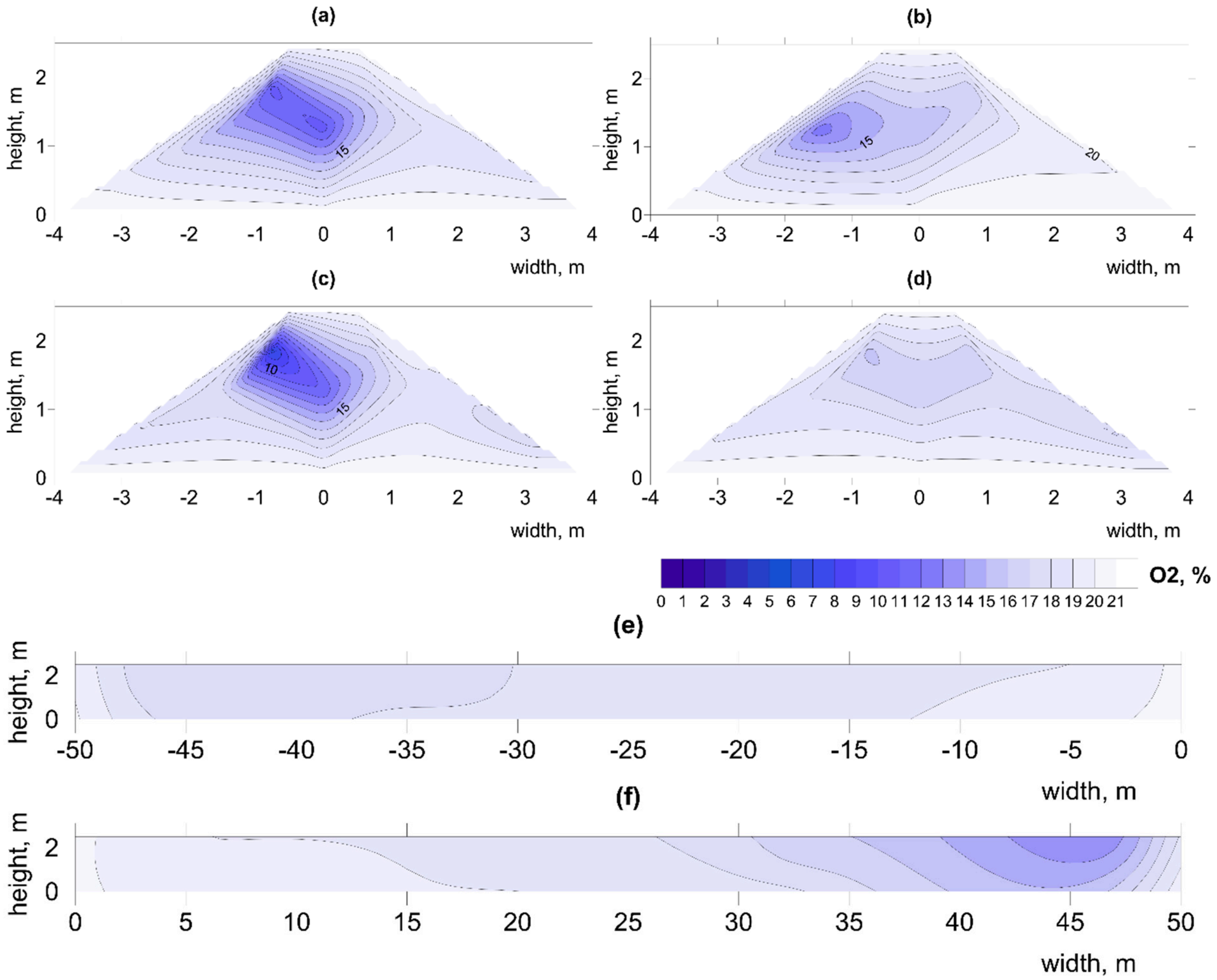

Figure S46. O2 distribution in the pile A1, day 20.

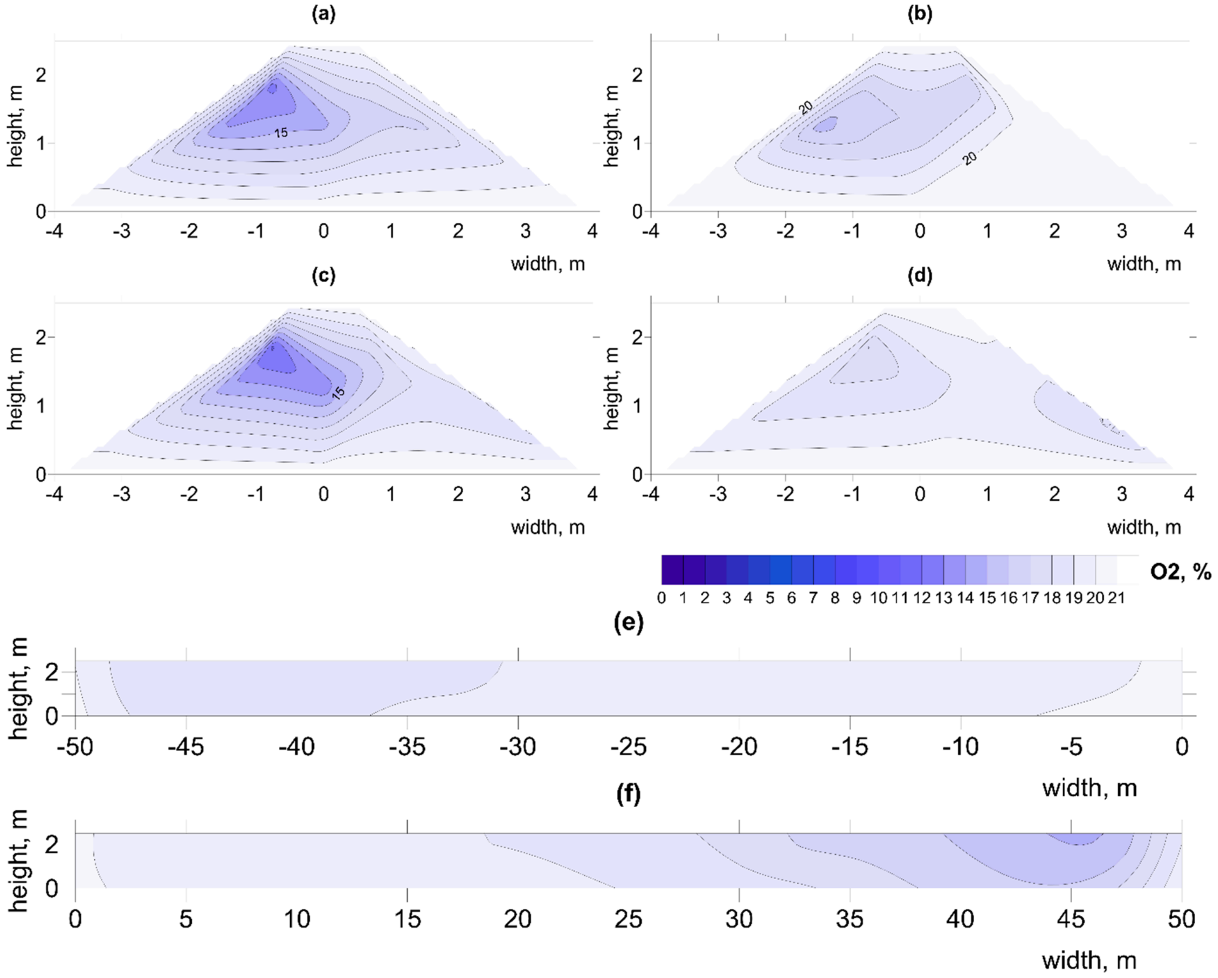

Figure S47. O2 distribution in the pile A1, day 28.

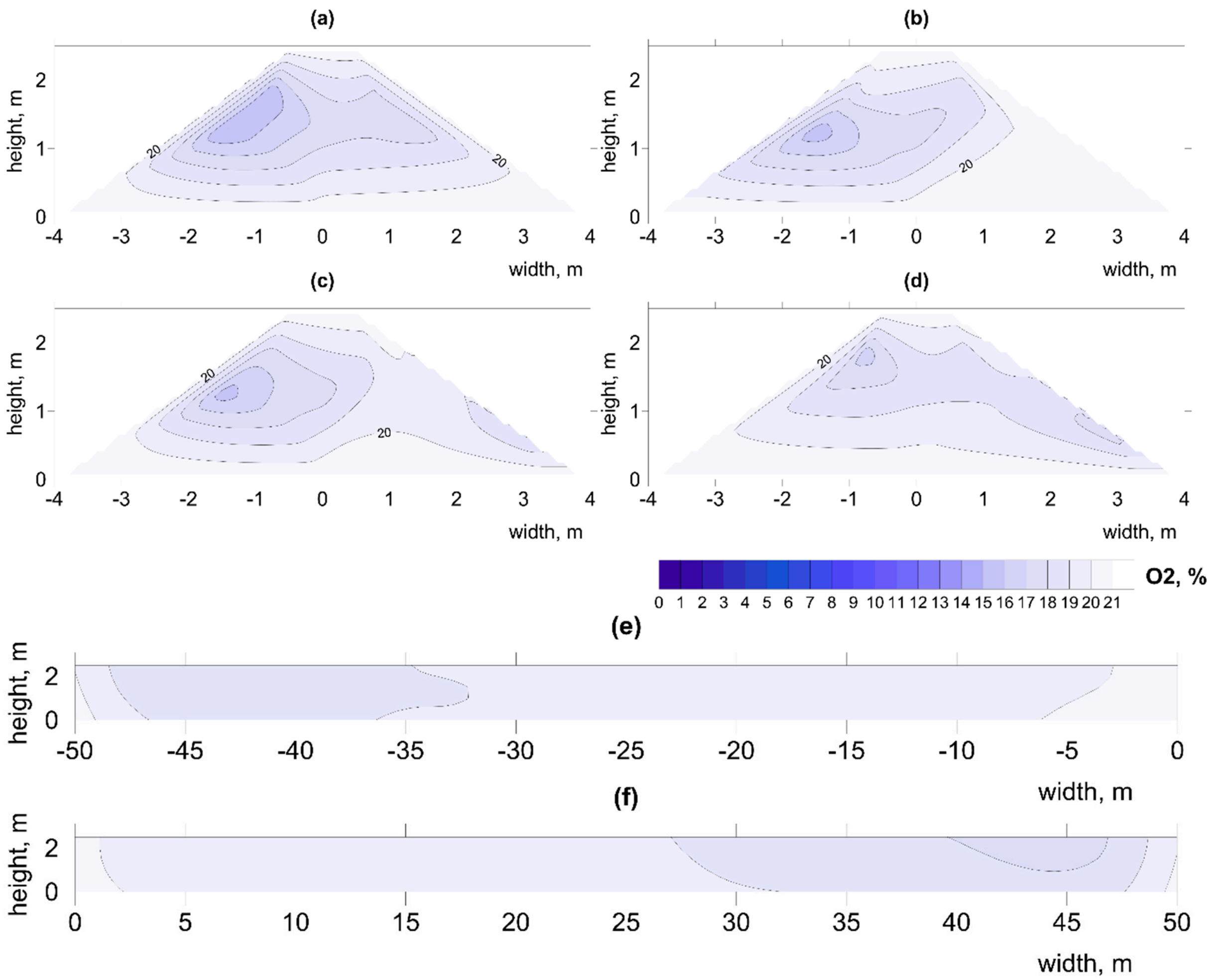

Figure S48. O2 distribution in the pile A1, day 35.

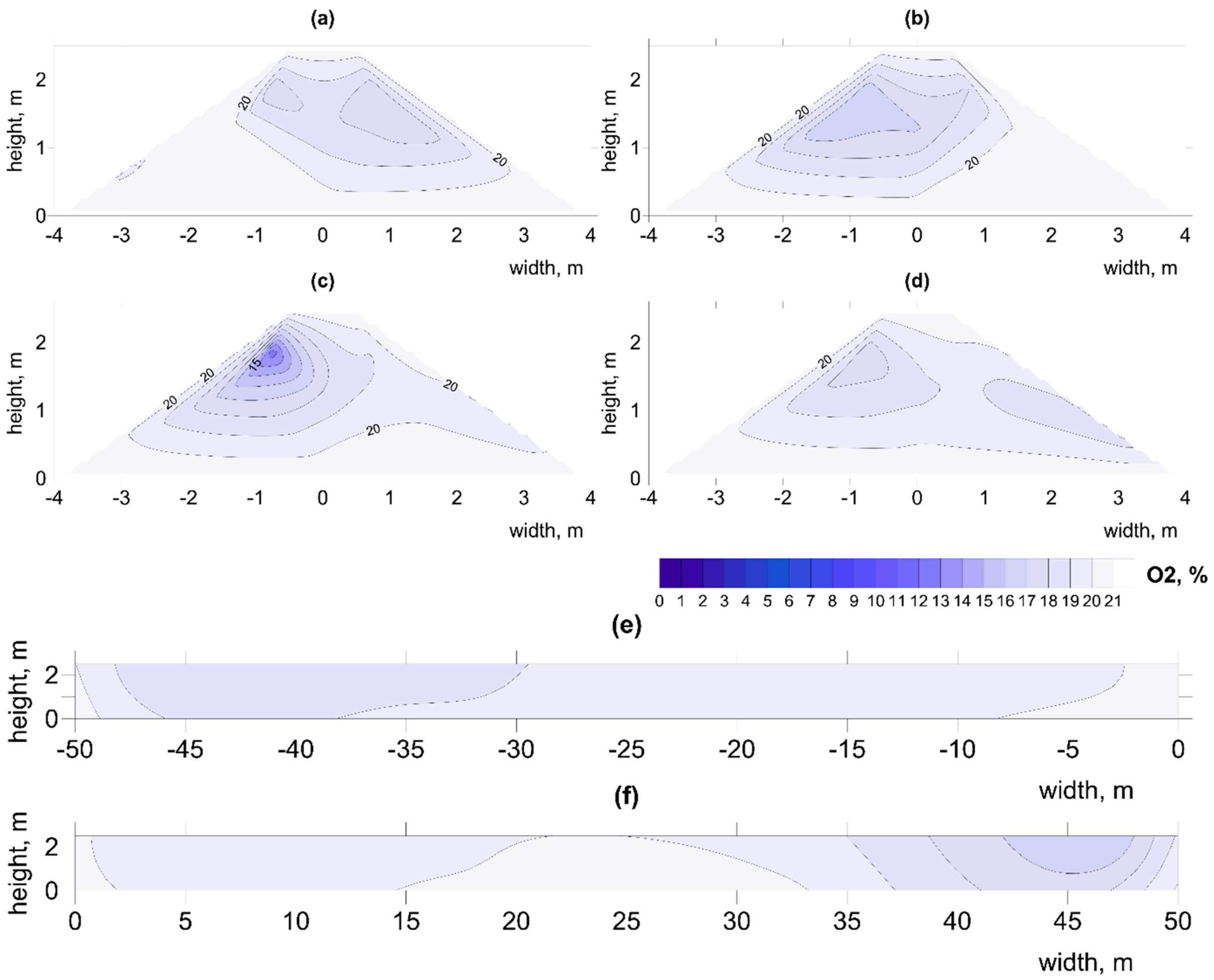

Figure S49. O2 distribution in the pile A1, day 41.

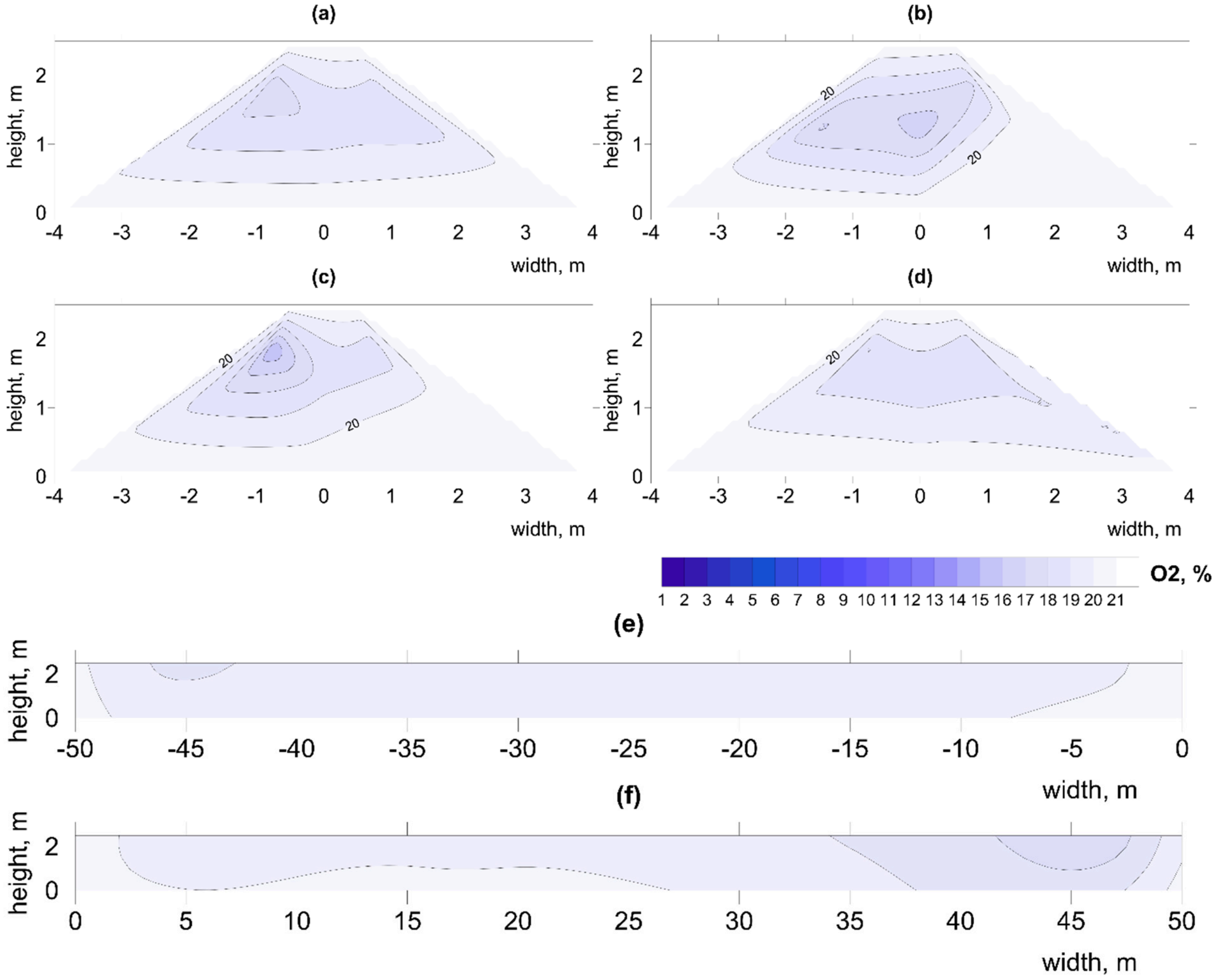

Figure S50. O2 distribution in the pile A1, day 50.

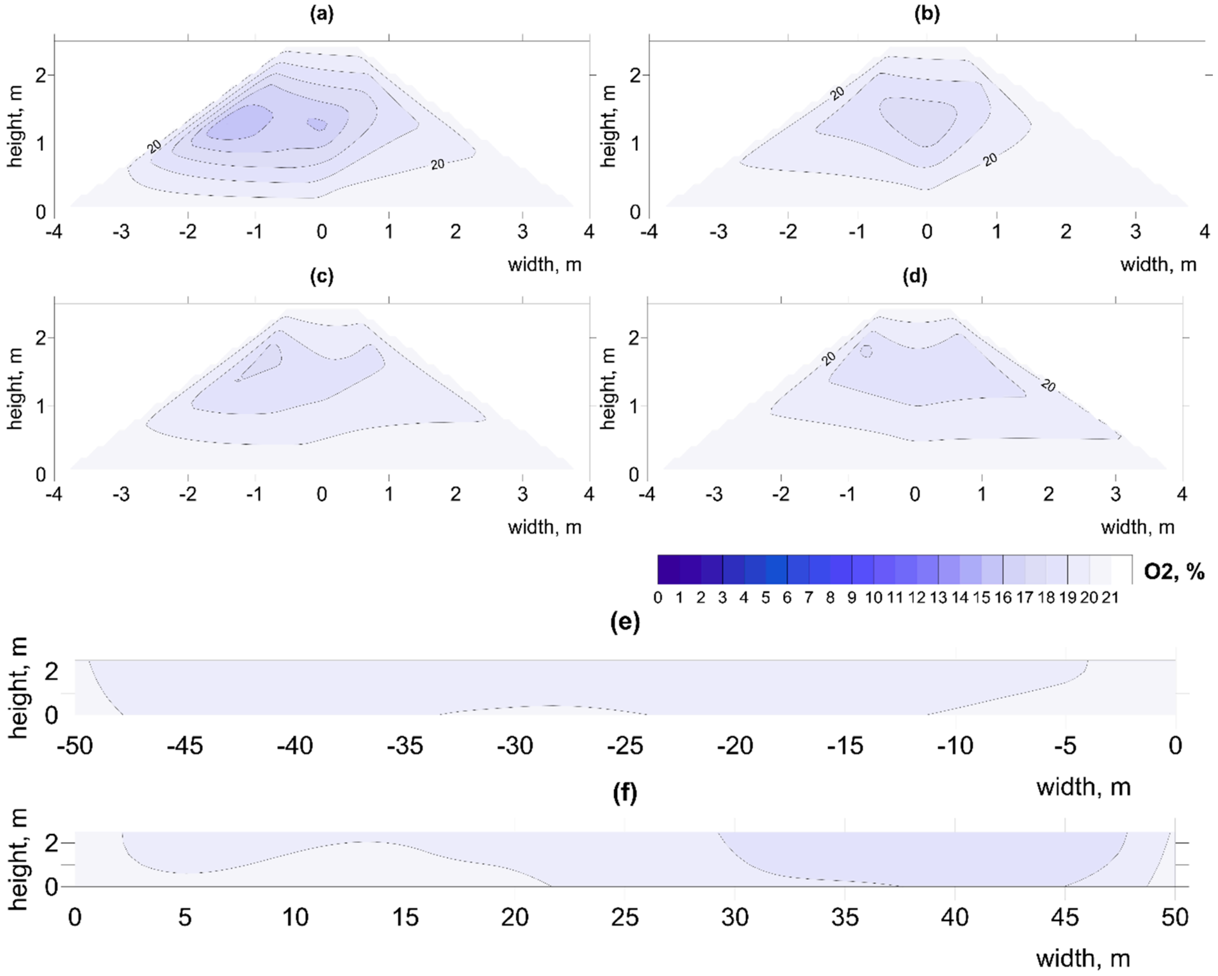

Figure S51. O2 distribution in the pile A1, day 60.

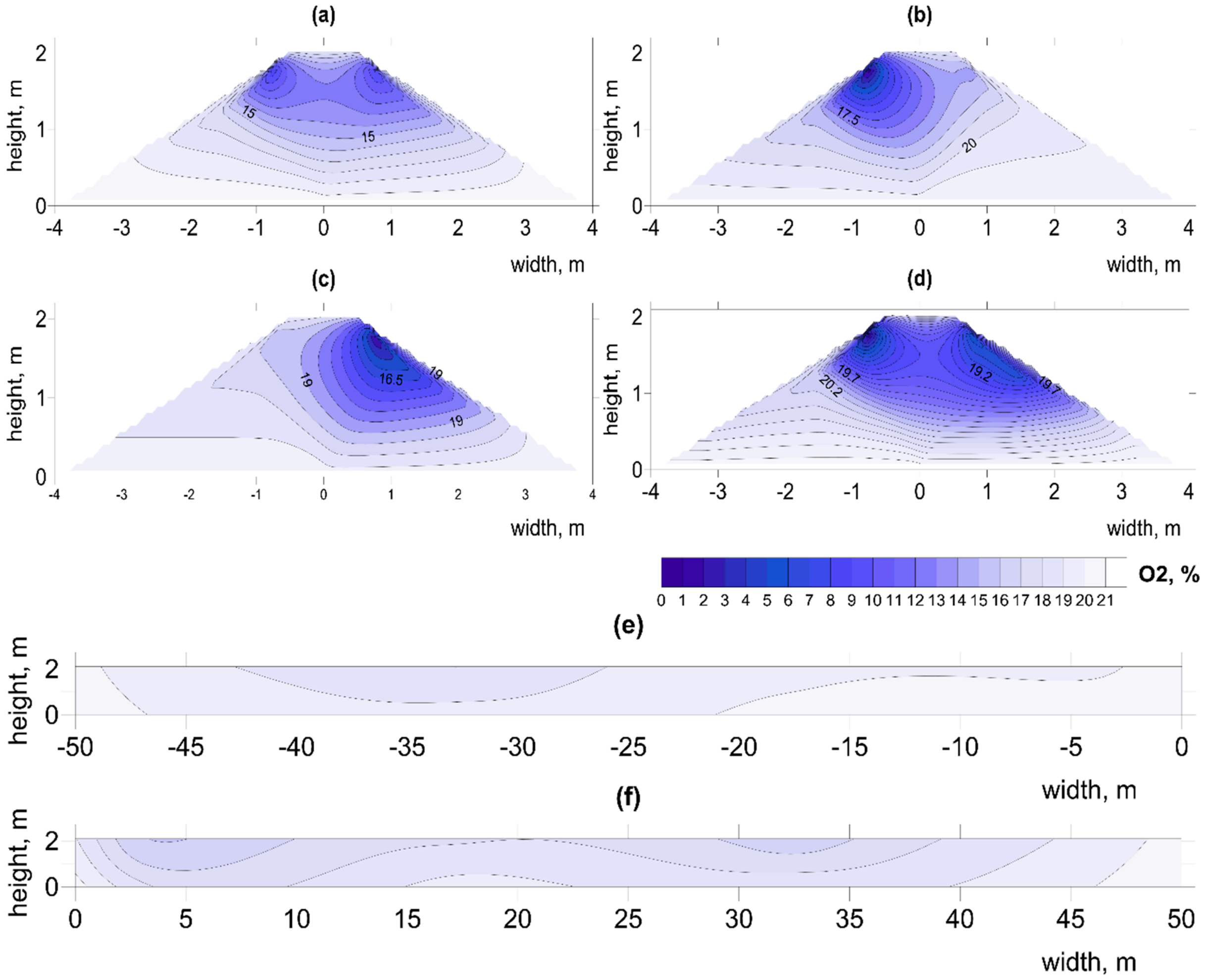

Figure S52. O2 distribution in the pile A2, day 1.

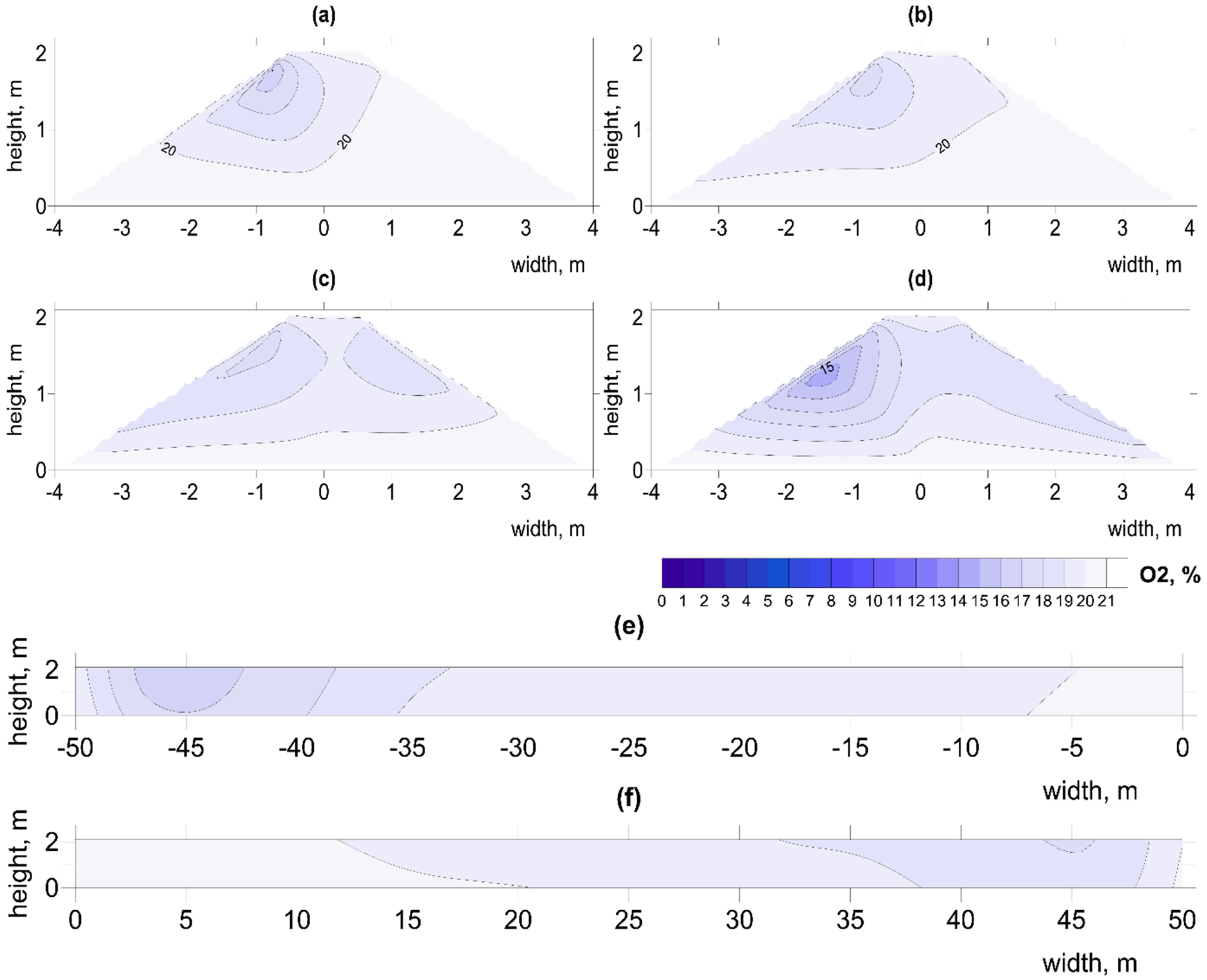

Figure S53. O2 distribution in the pile A2, day 11.

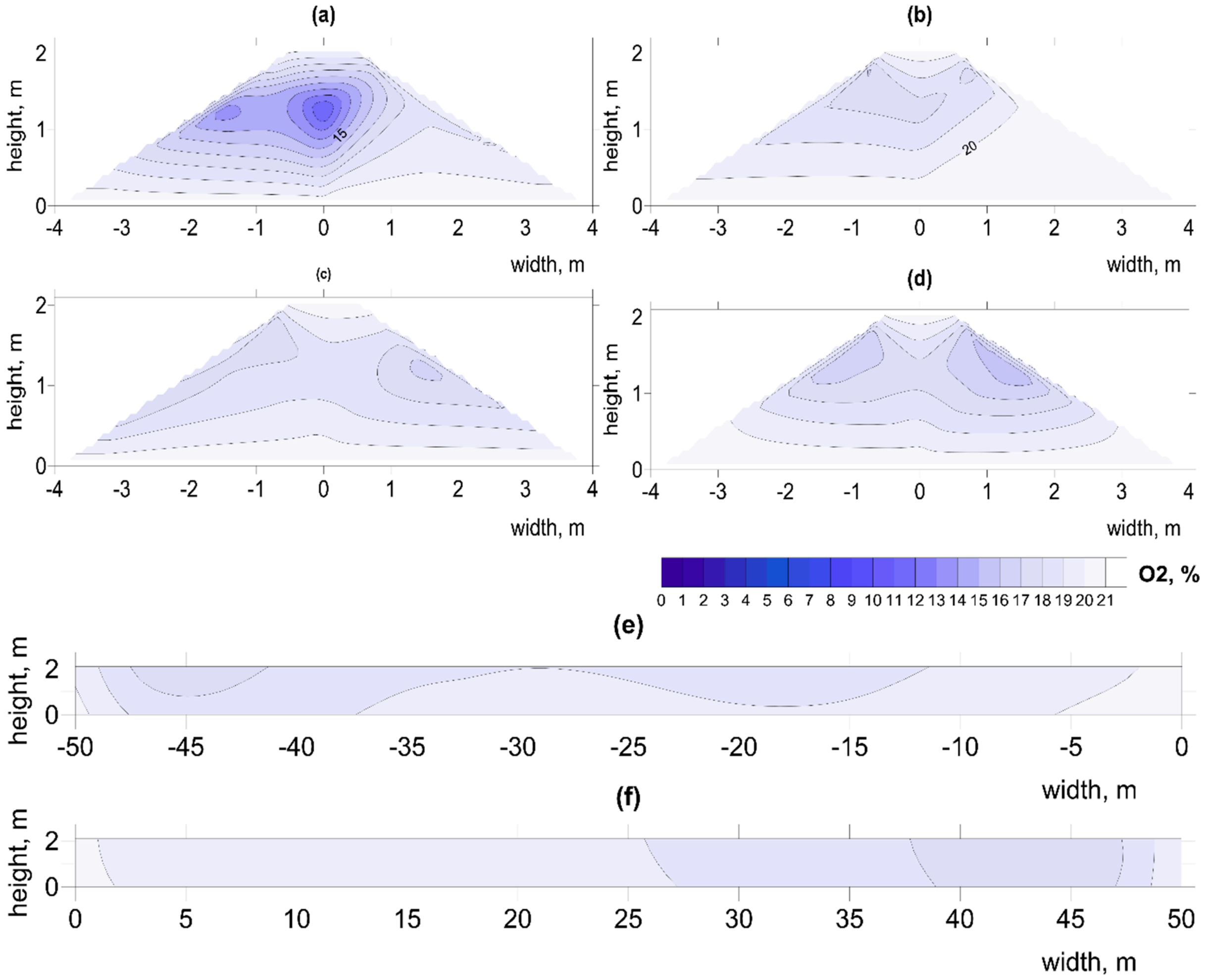

Figure S54. O2 distribution in the pile A2, day 17.

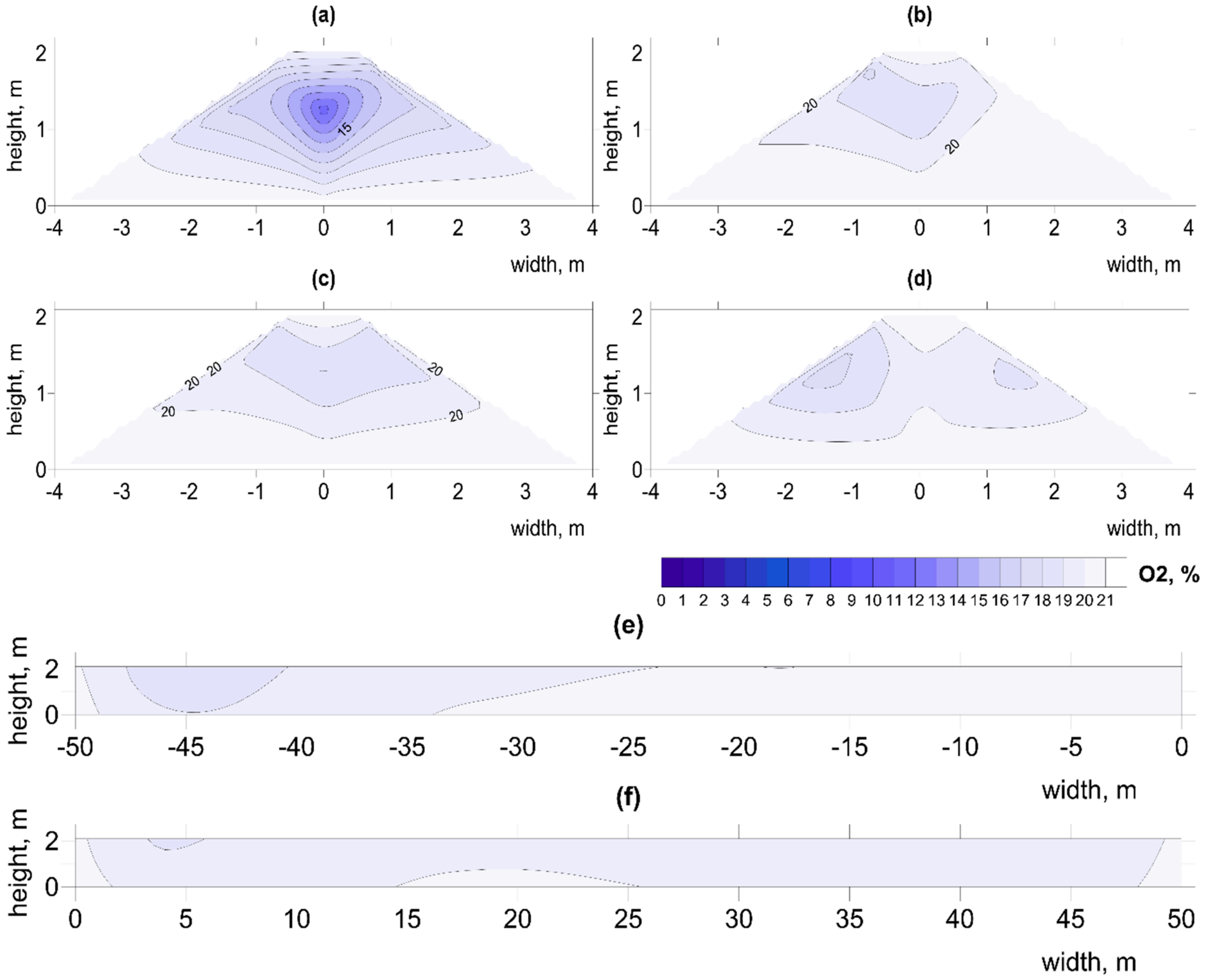

Figure S55. O2 distribution in the pile A2, day 25.

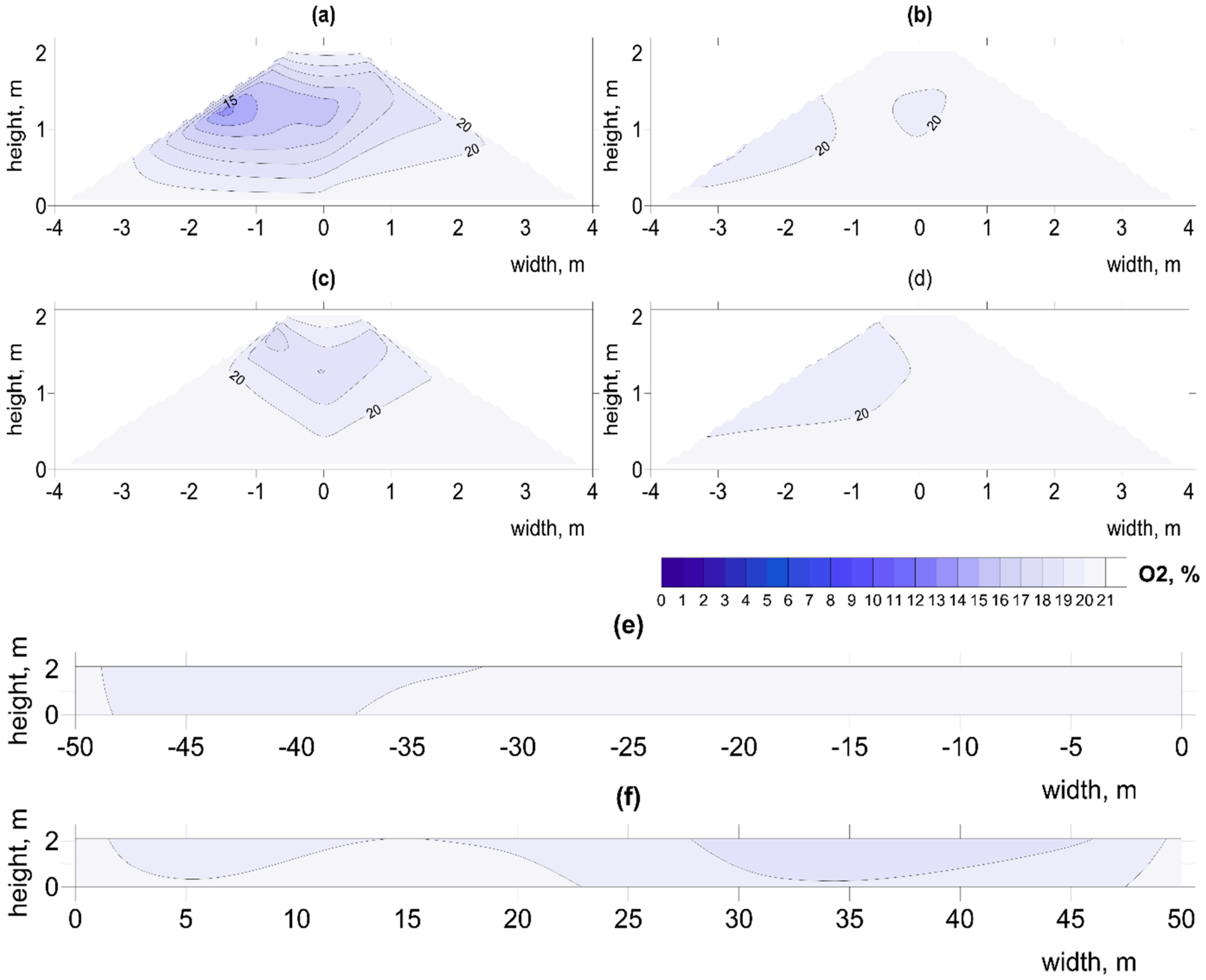

Figure S56. O2 distribution in the pile A2, day 32.

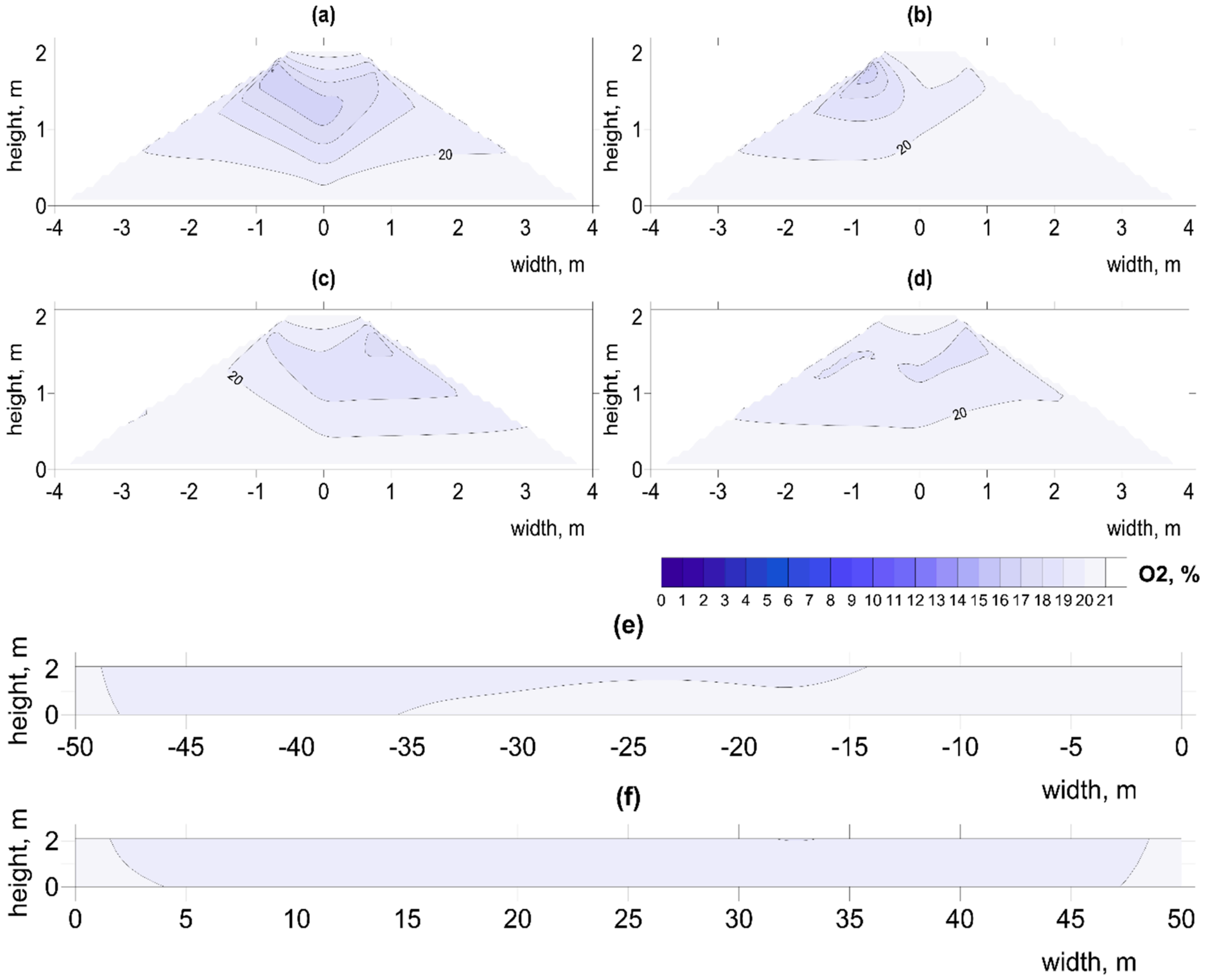

Figure S57. O2 distribution in the pile A2, day 38.

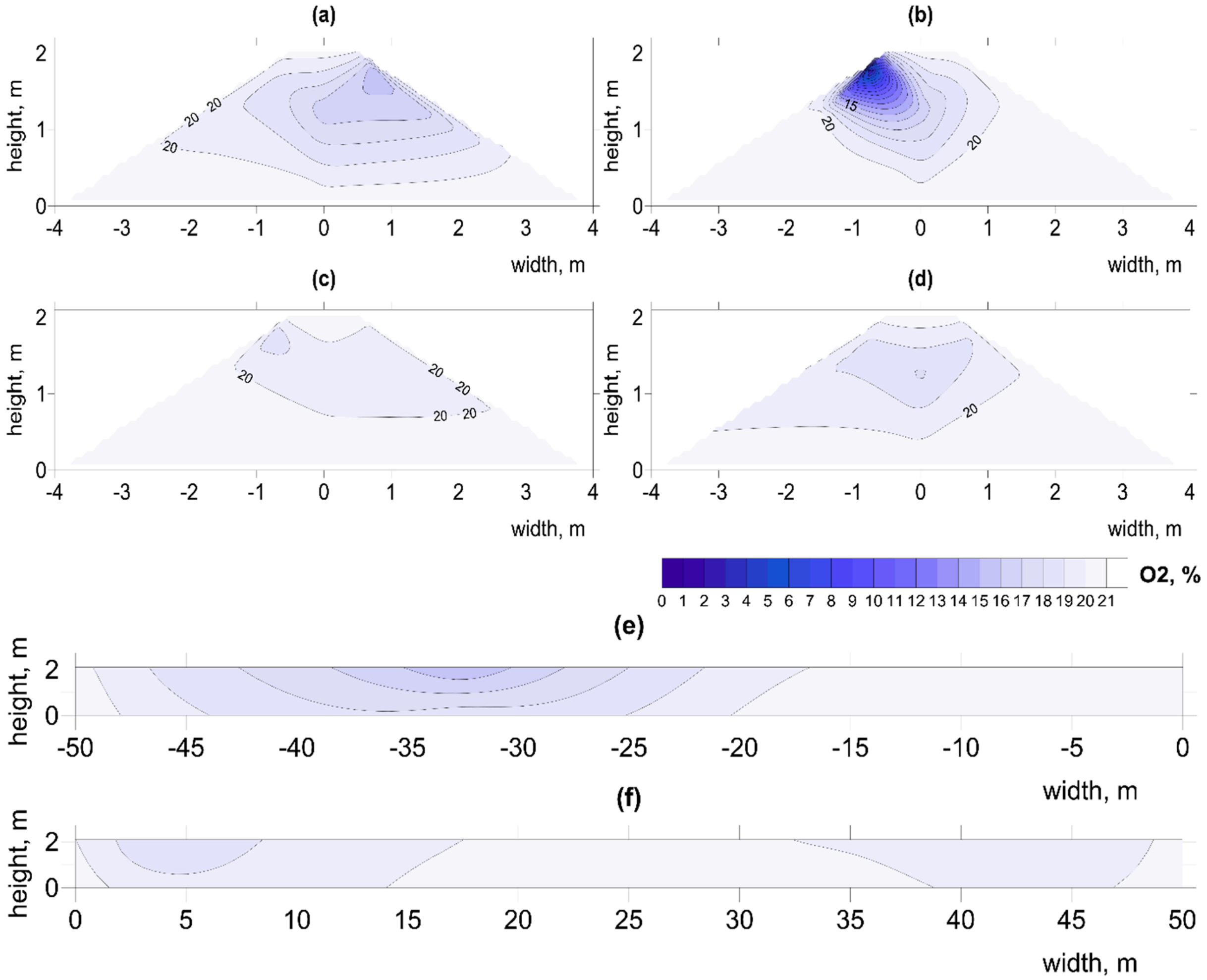

Figure S58. O2 distribution in the pile A2, day 47.

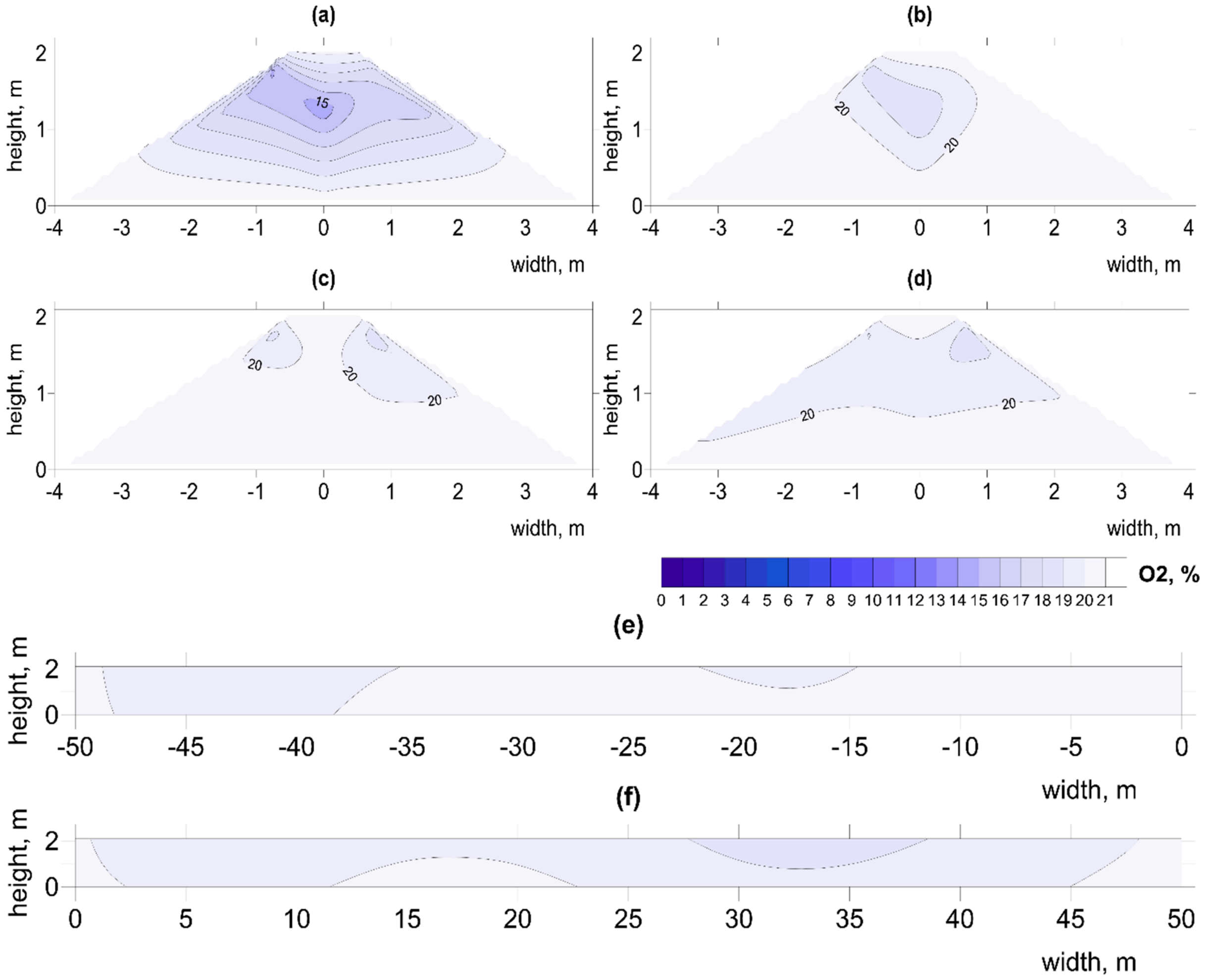

Figure S59. O2 distribution in the pile A2, day 57.

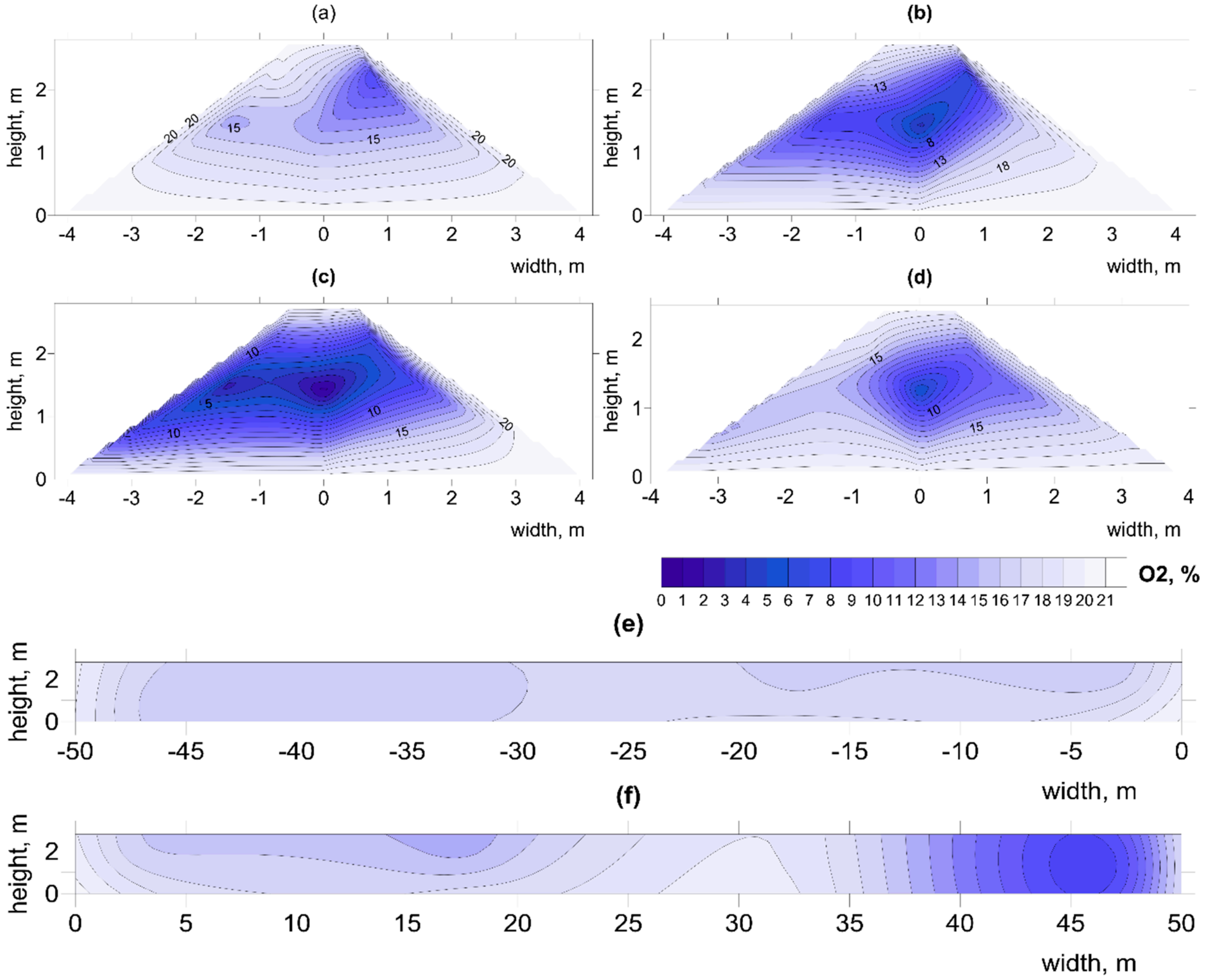

Figure S60. O2 distribution in the pile B1, day 1.

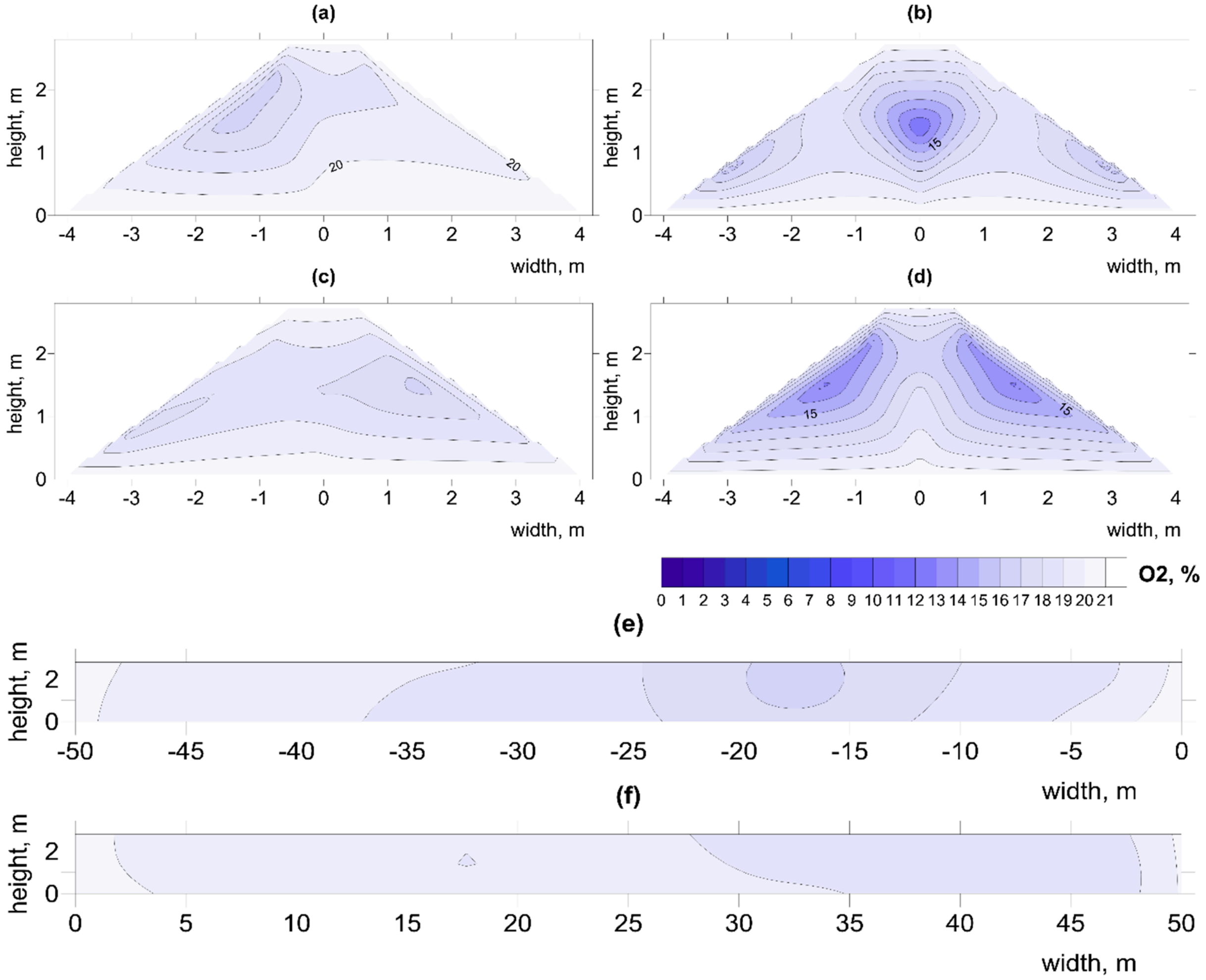

Figure S61. O2 distribution in the pile B1, day 9.

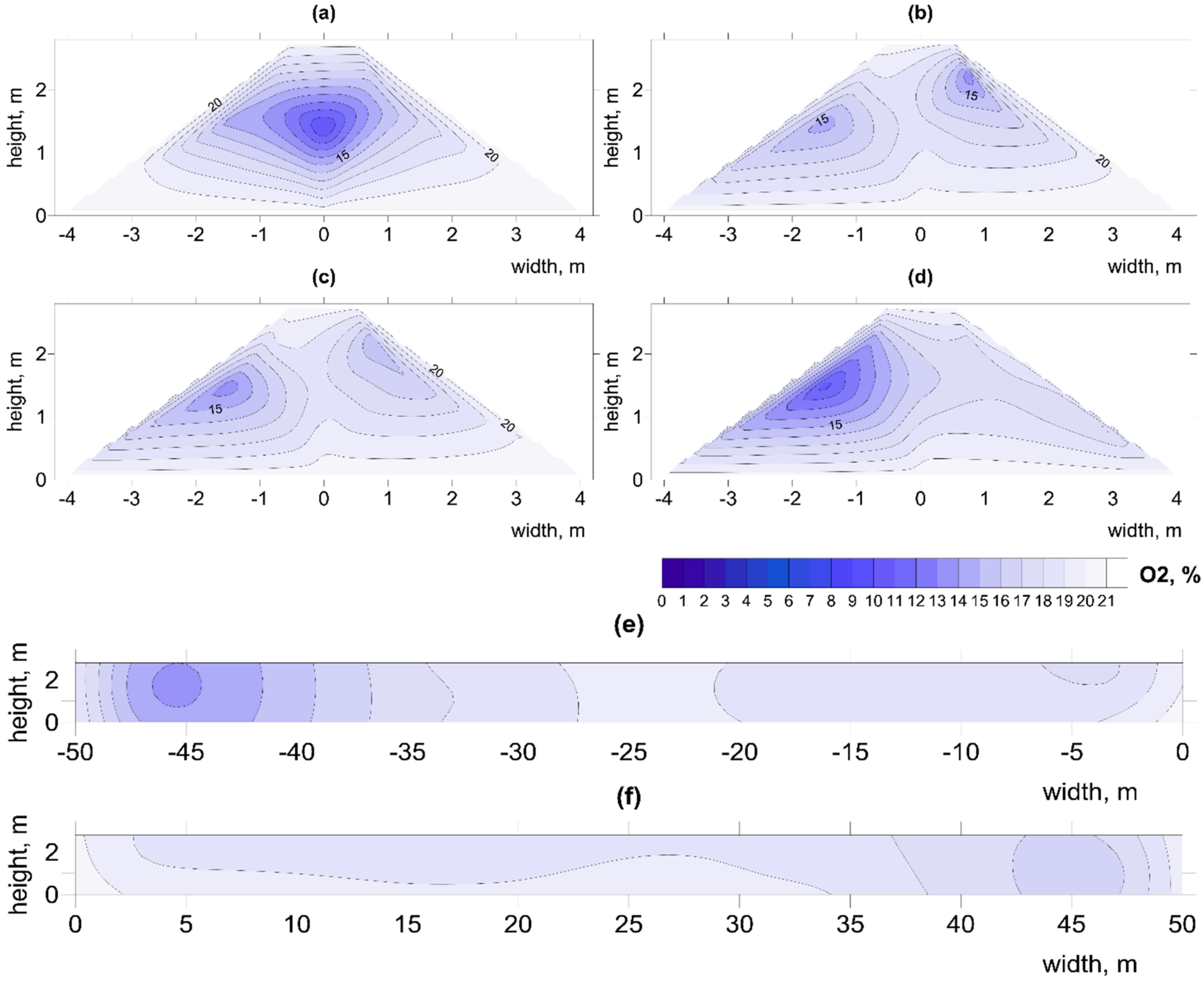

Figure S62. O2 distribution in the pile B1, day 16.

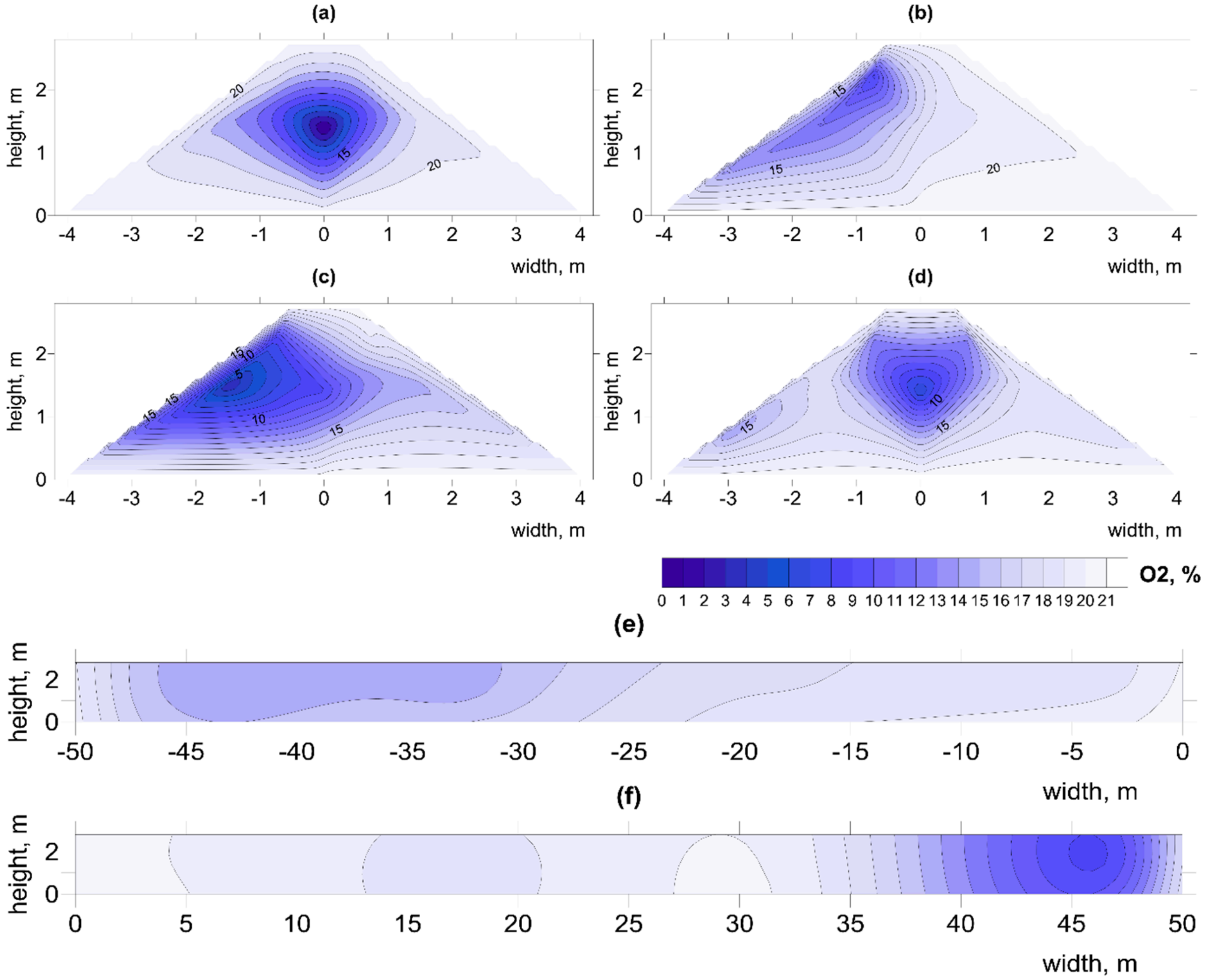

Figure S63. O2 distribution in the pile B1, day 31.

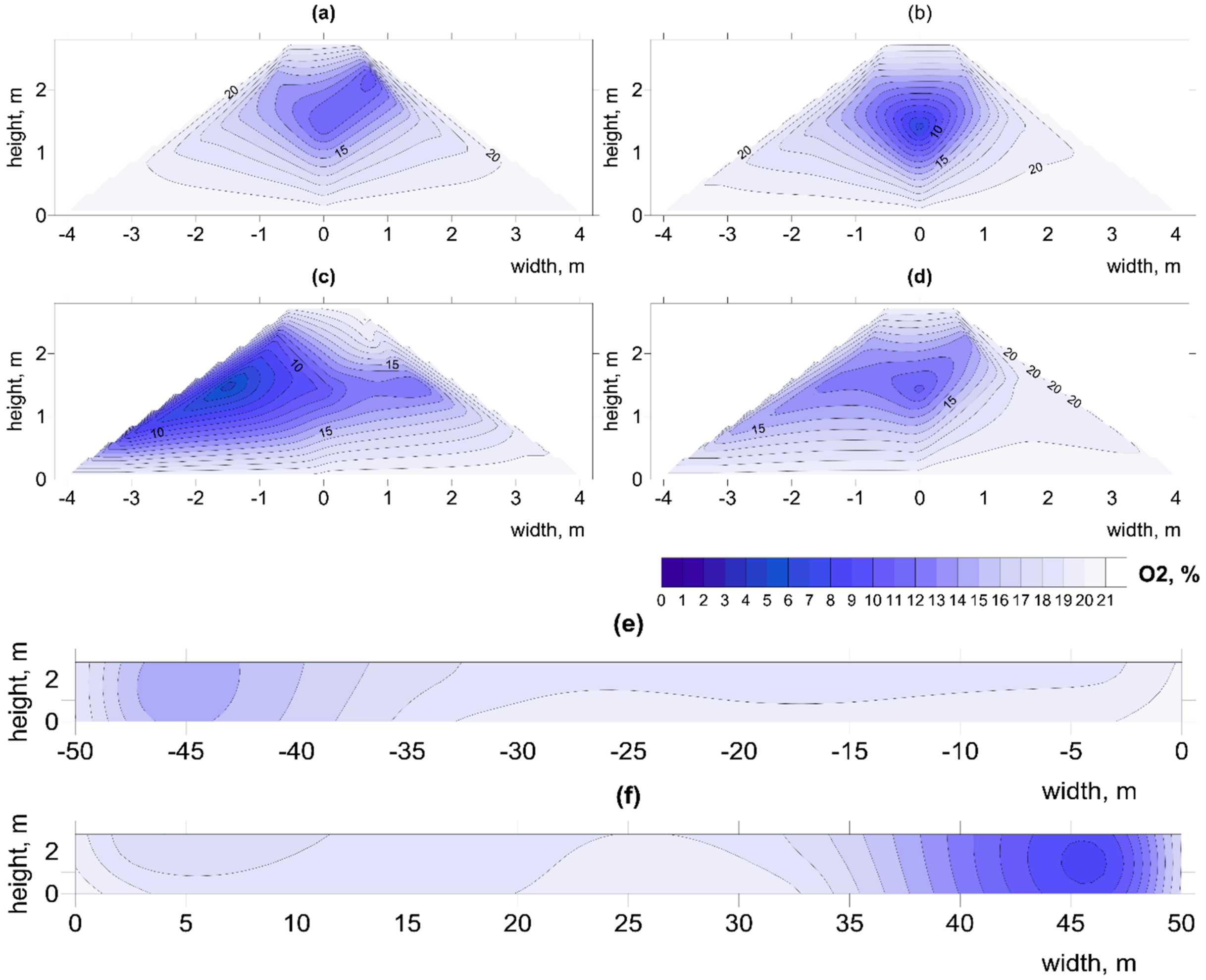

Figure S64. O2 distribution in the pile B1, day 41.

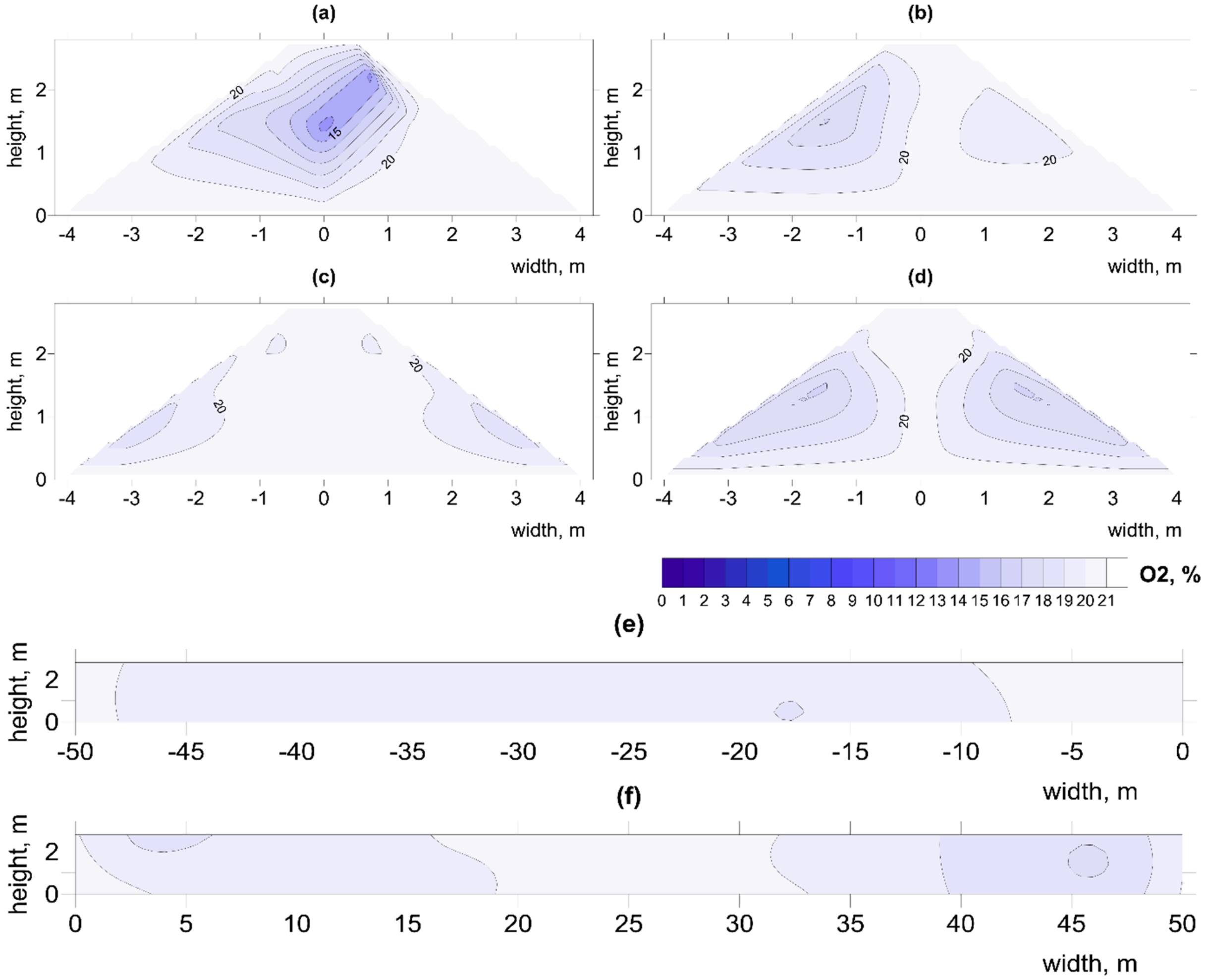

Figure S65. O2 distribution in the pile B1, day 50.

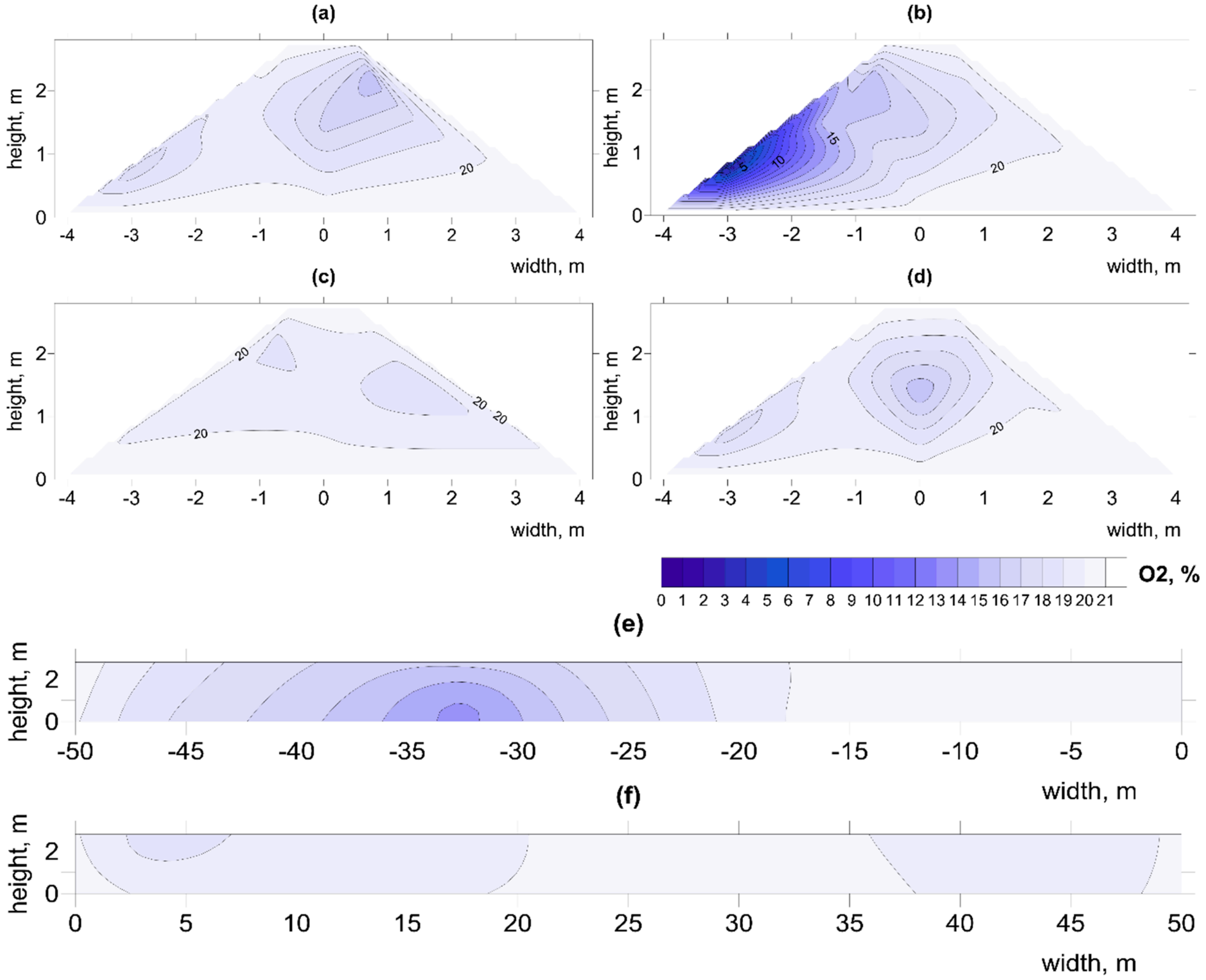

Figure S66. O2 distribution in the pile B1, day 57.

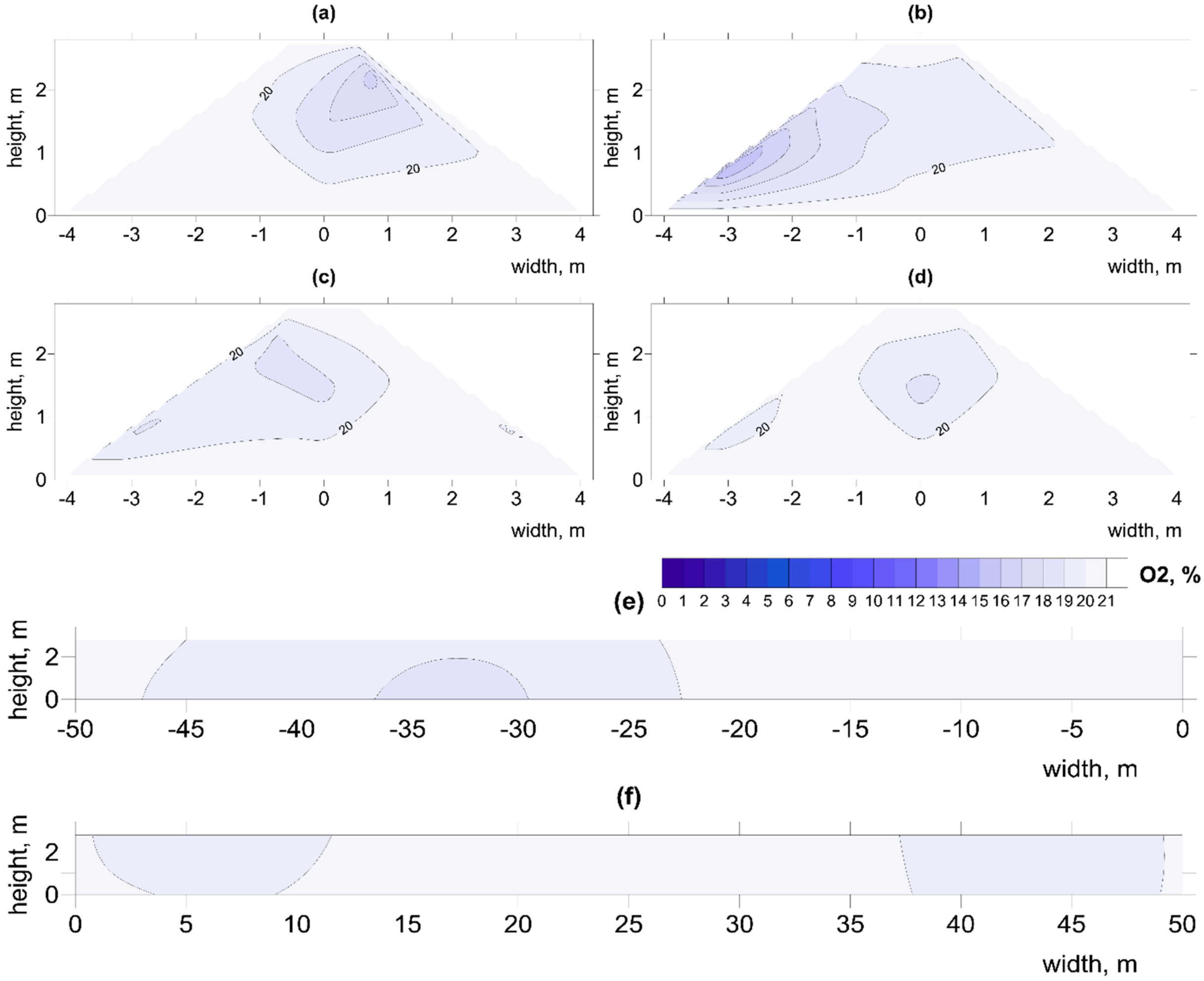

Figure S67. O2 distribution in the pile B1, day 64.

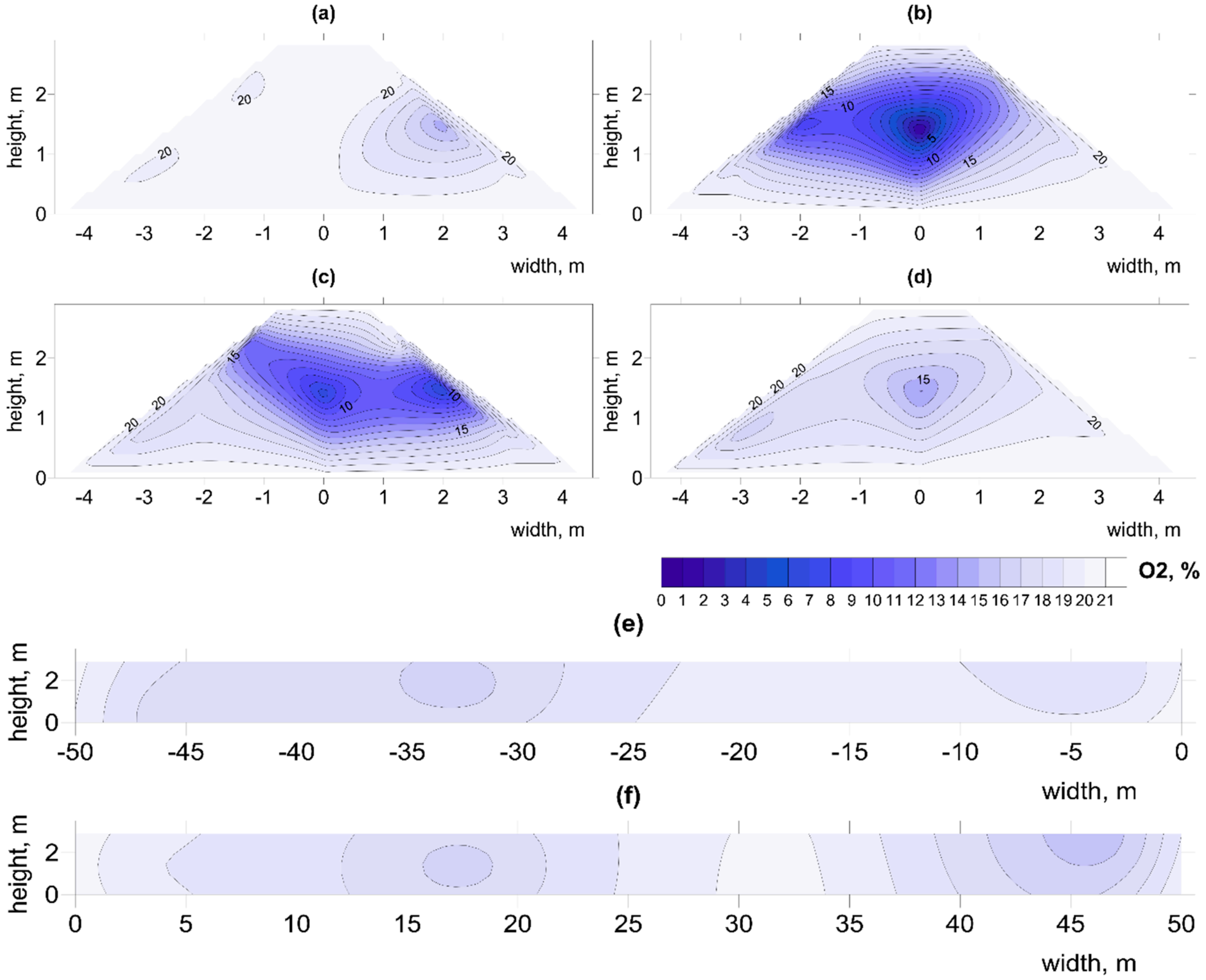

Figure S68. O2 distribution in the pile B2, day 1.

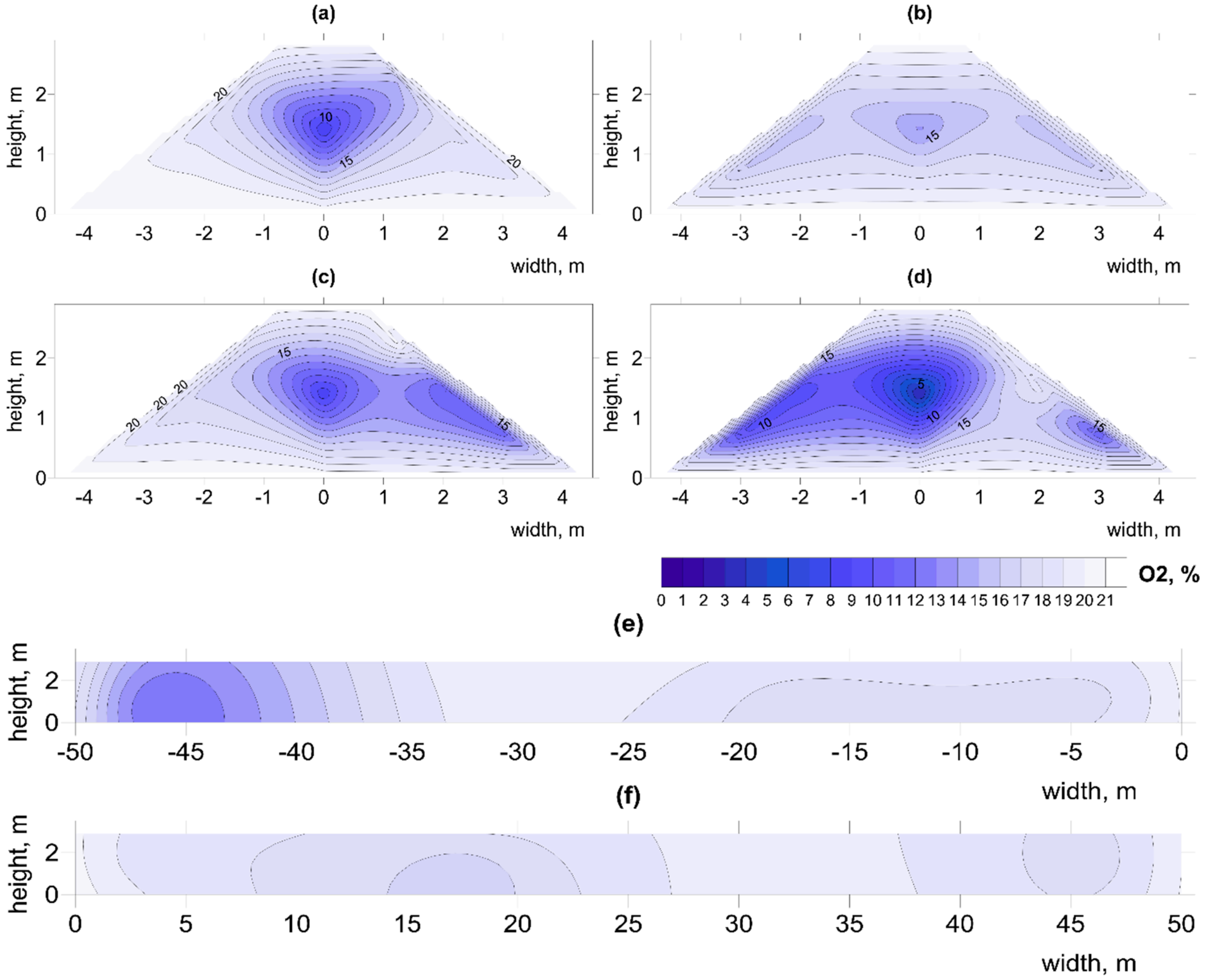

Figure S69. O2 distribution in the pile B2, day 8.

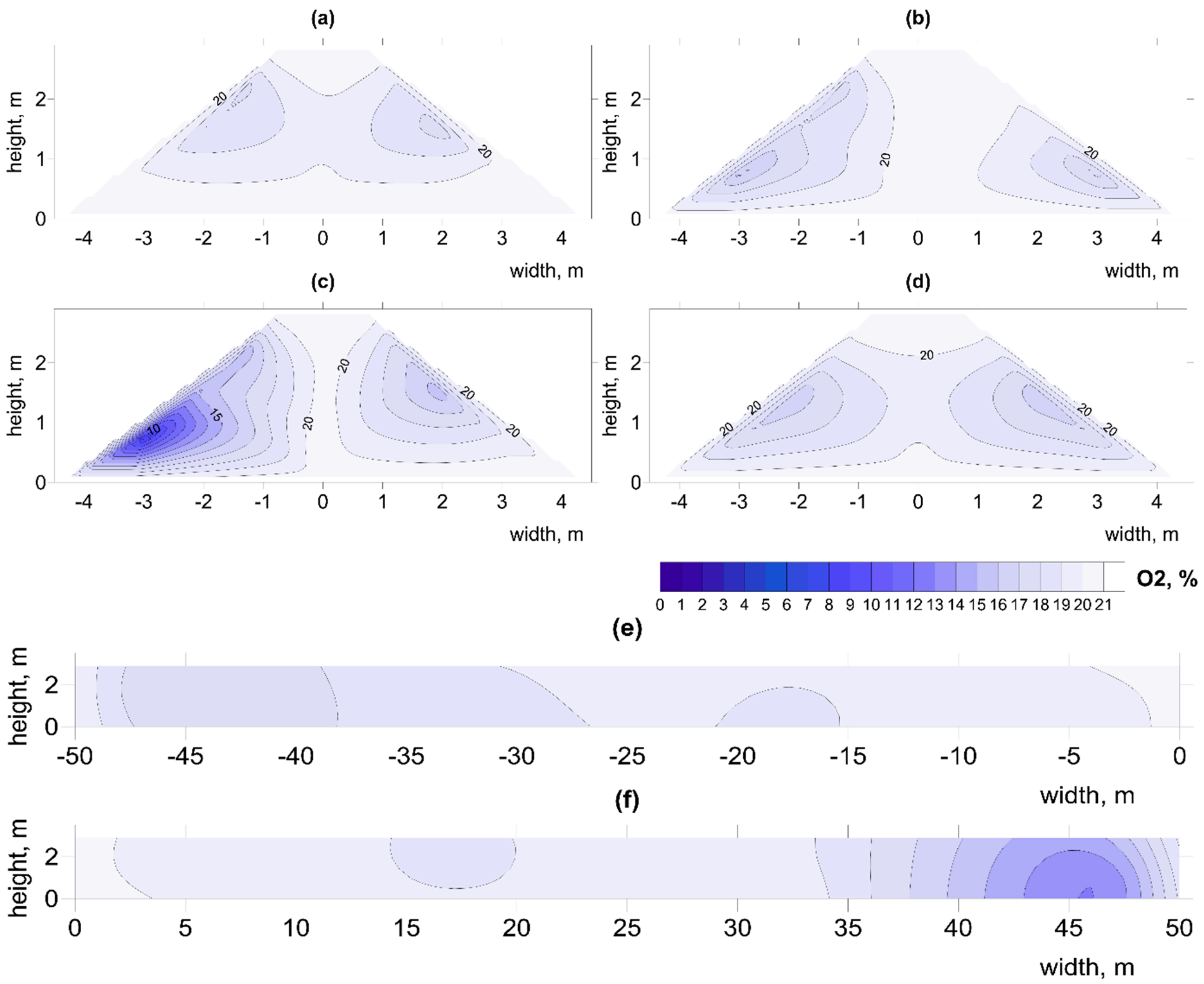

Figure S70. O2 distribution in the pile B2, day 16.

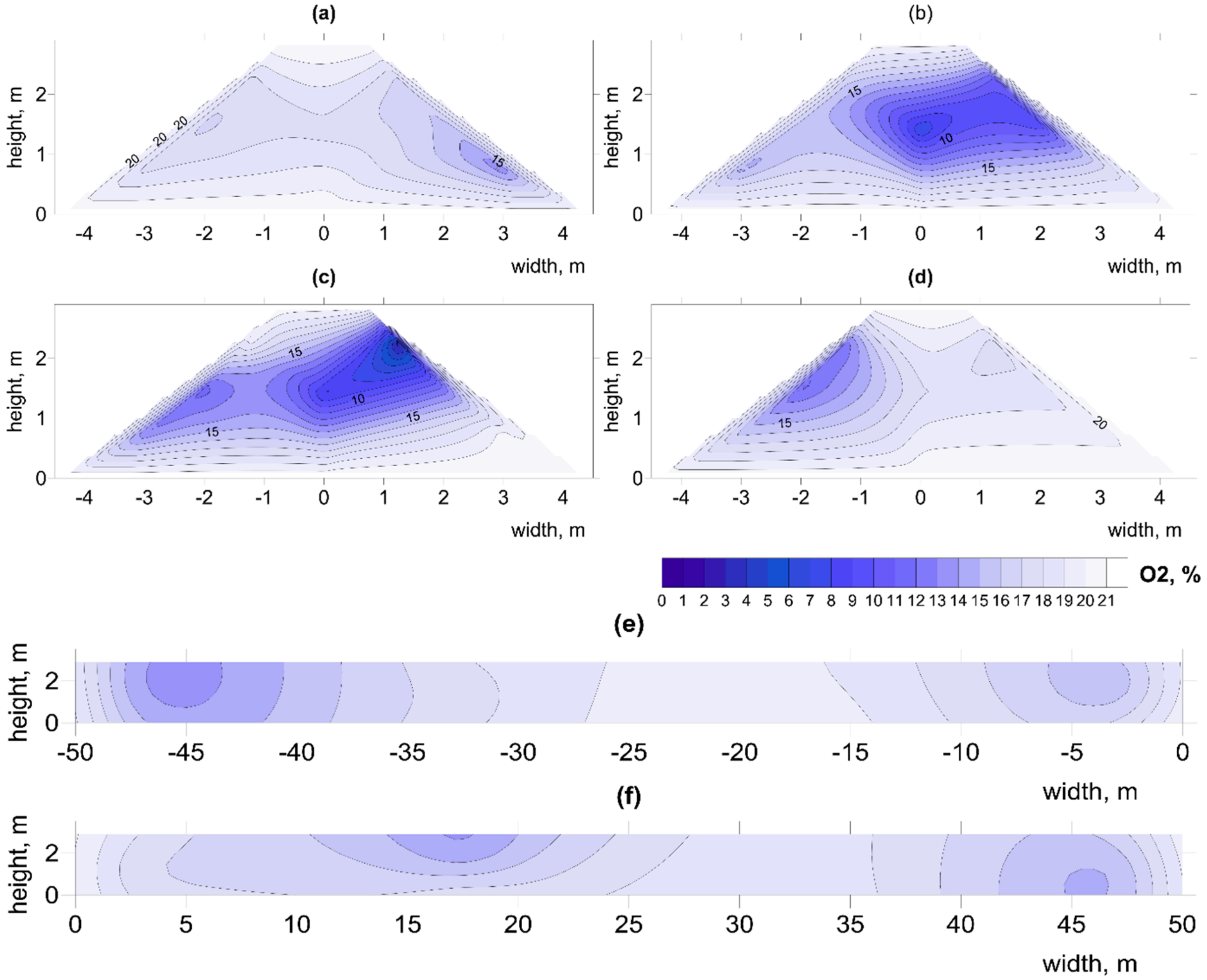

Figure S71. O2 distribution in the pile B2, day 24.

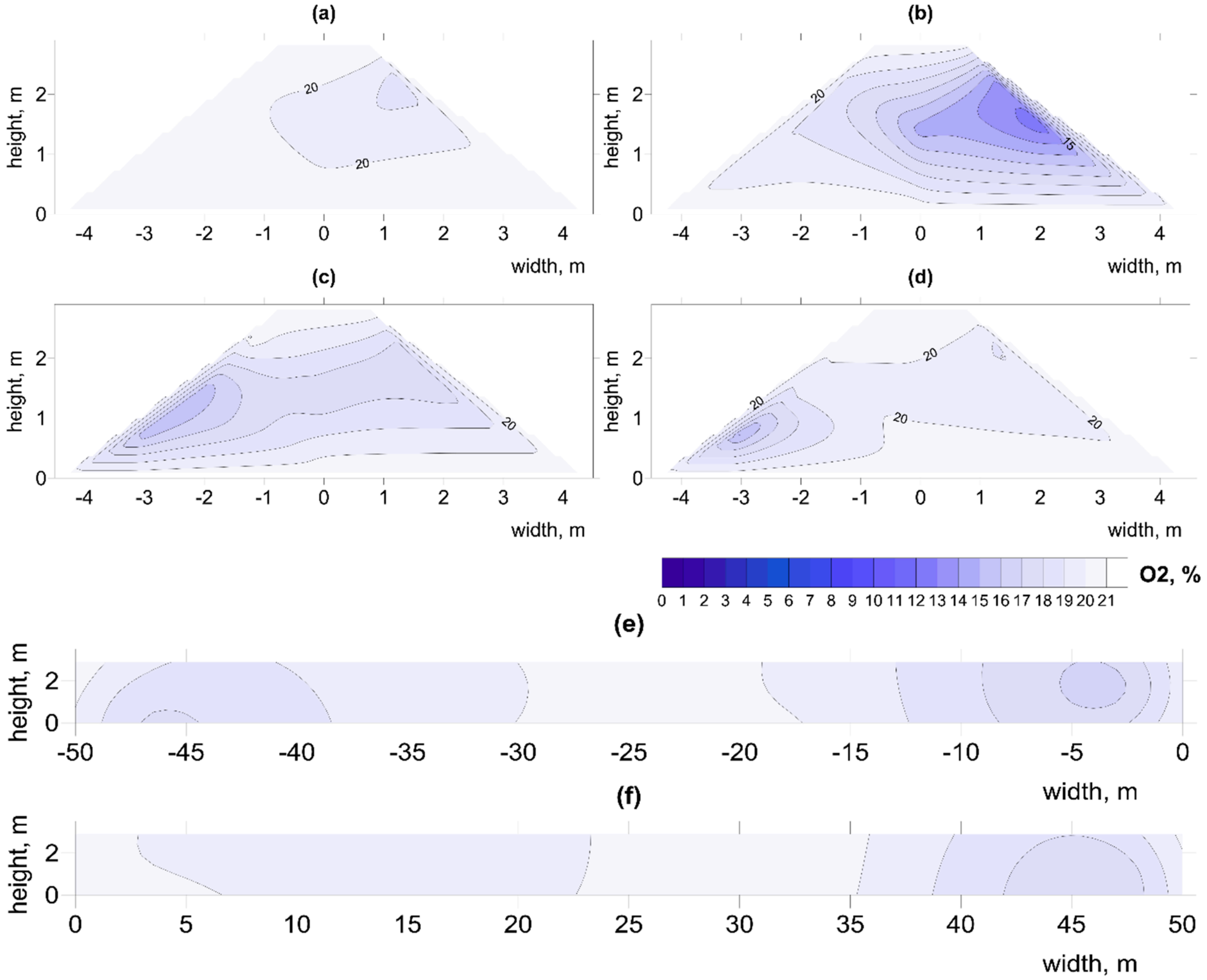

Figure S72. O2 distribution in the pile B2, day 36.

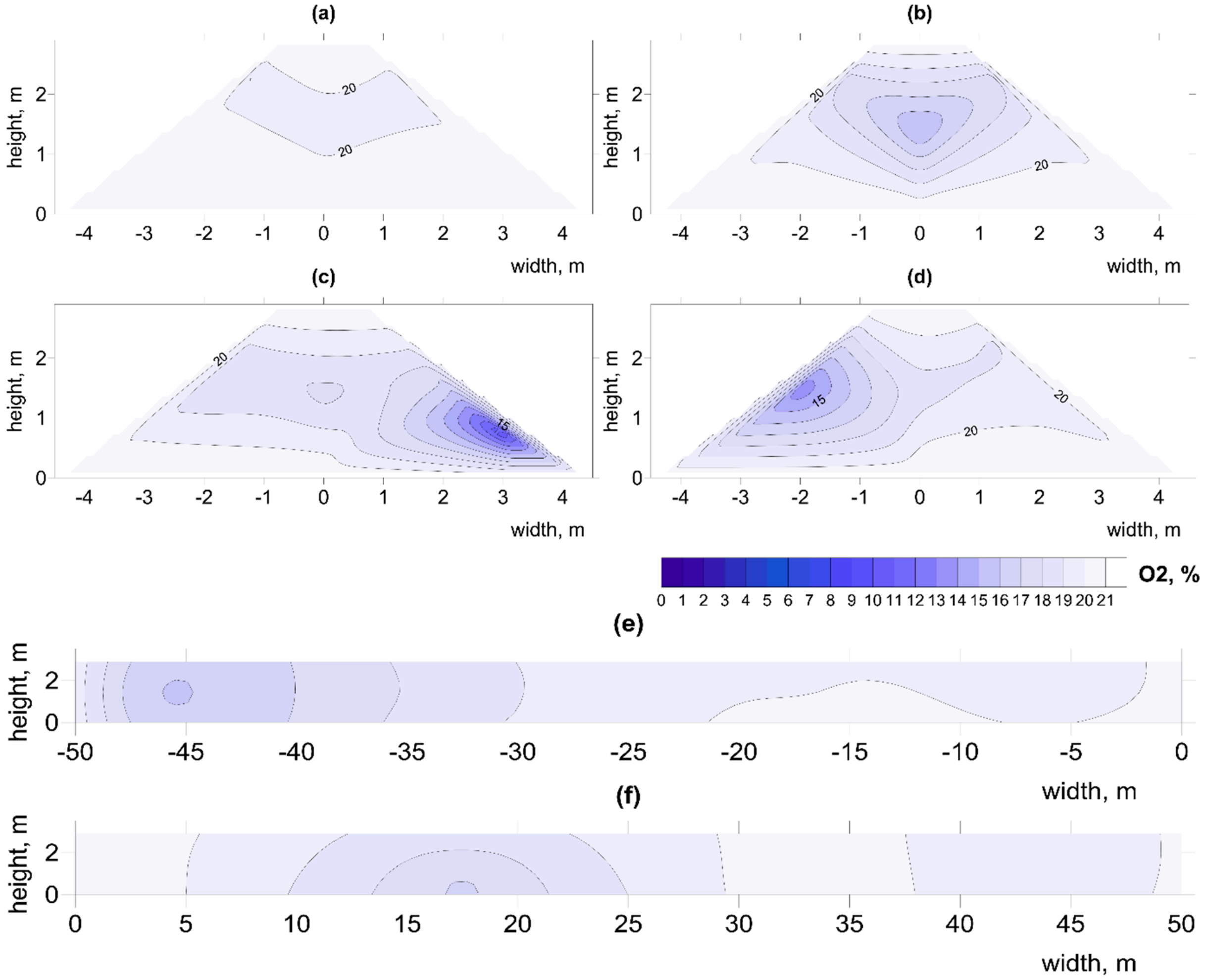

Figure S73. O2 distribution in the pile B2, day 43.

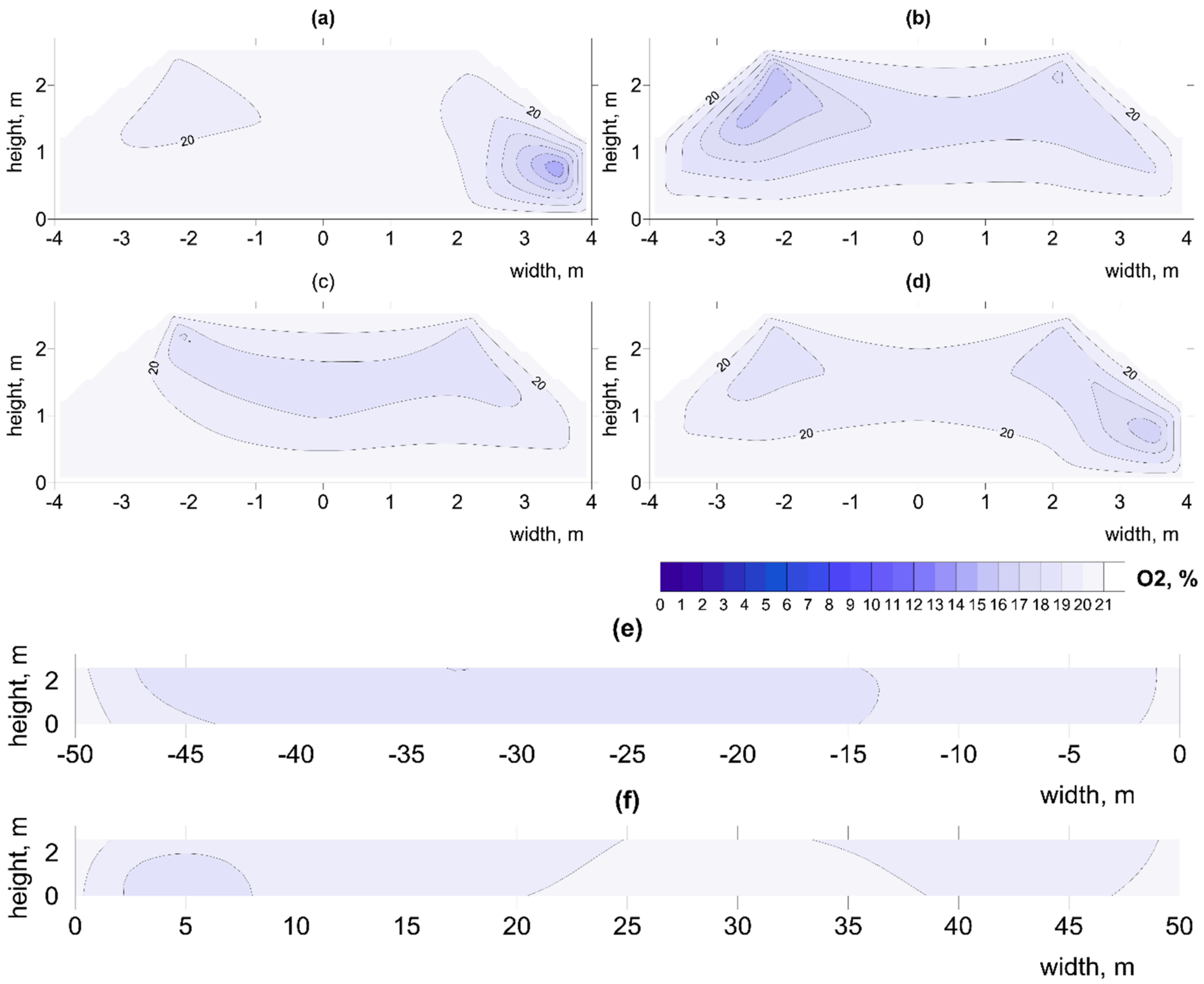

Figure S74. O2 distribution in the pile C1, day 1.

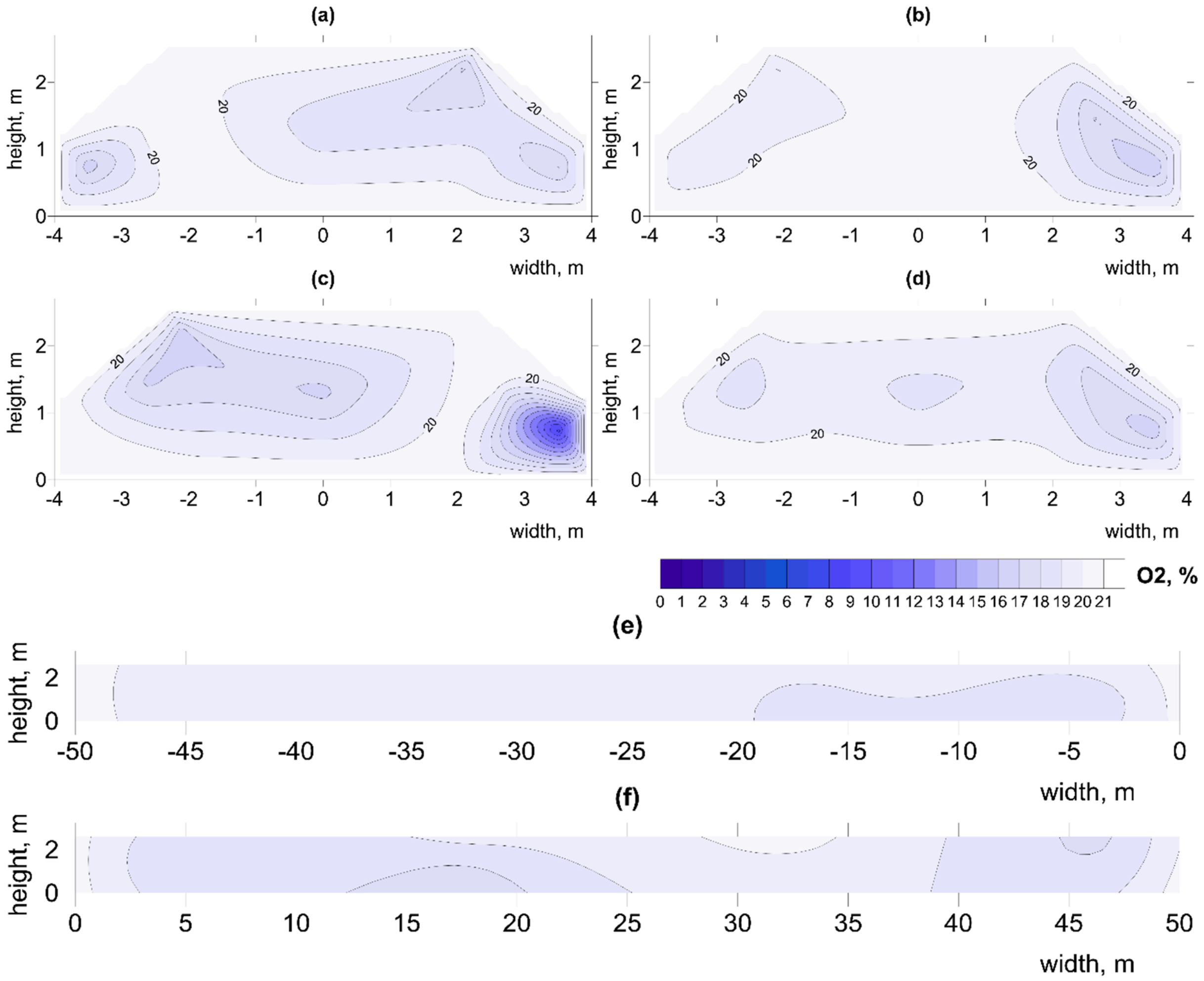

Figure S75. O2 distribution in the pile C1, day 9.

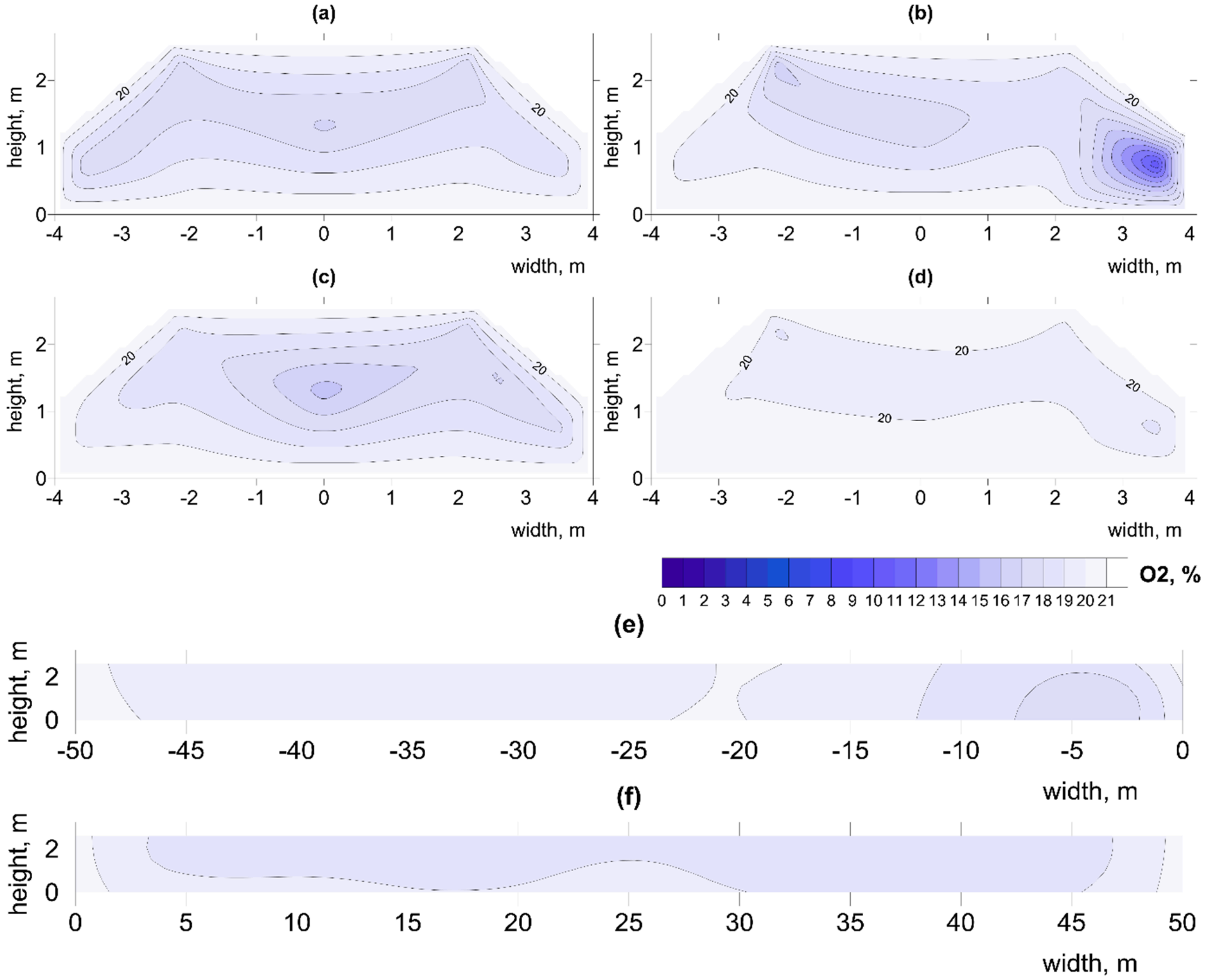

Figure S76. O2 distribution in the pile C1, day 16.

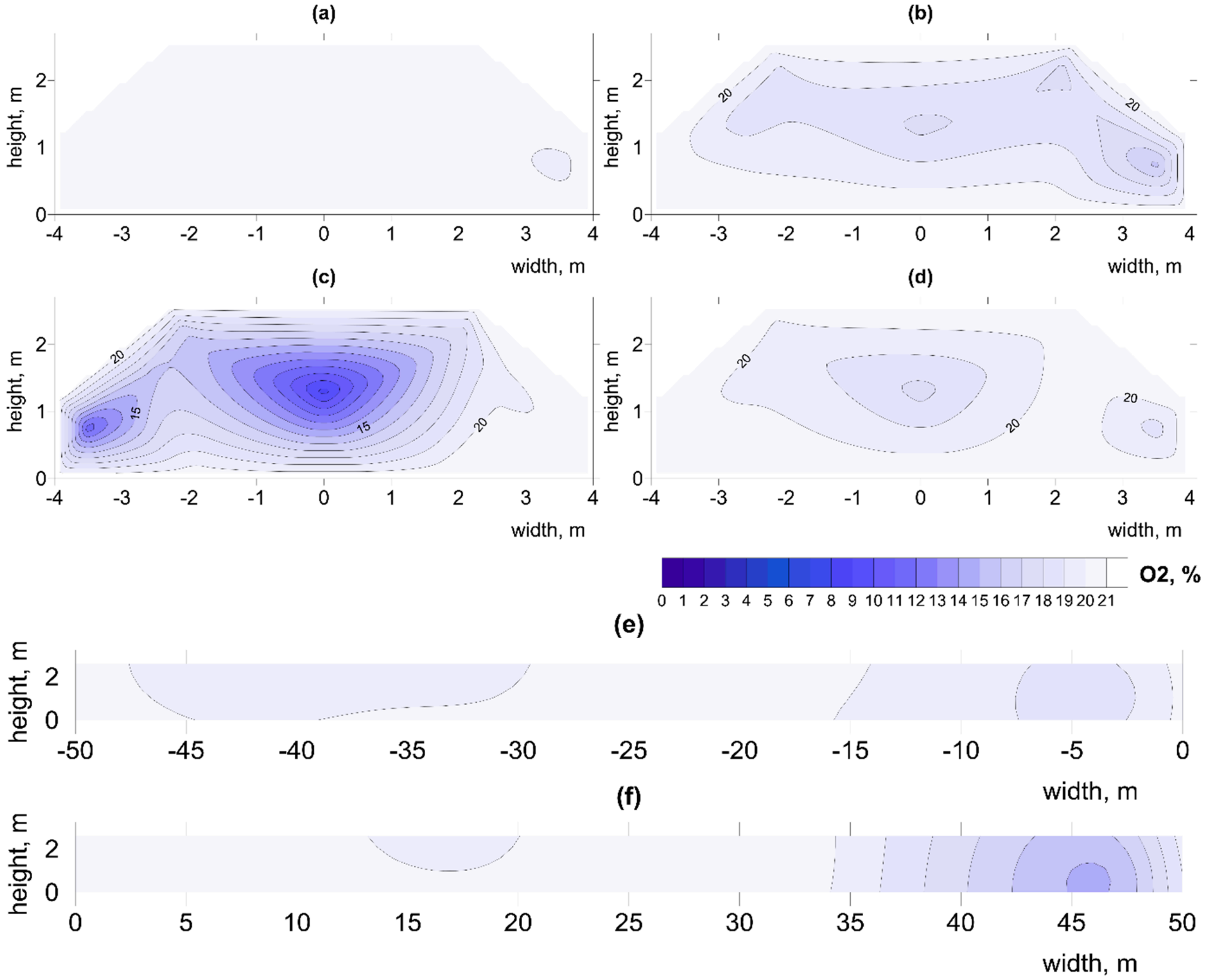

Figure S77. O2 distribution in the pile C1, day 28.

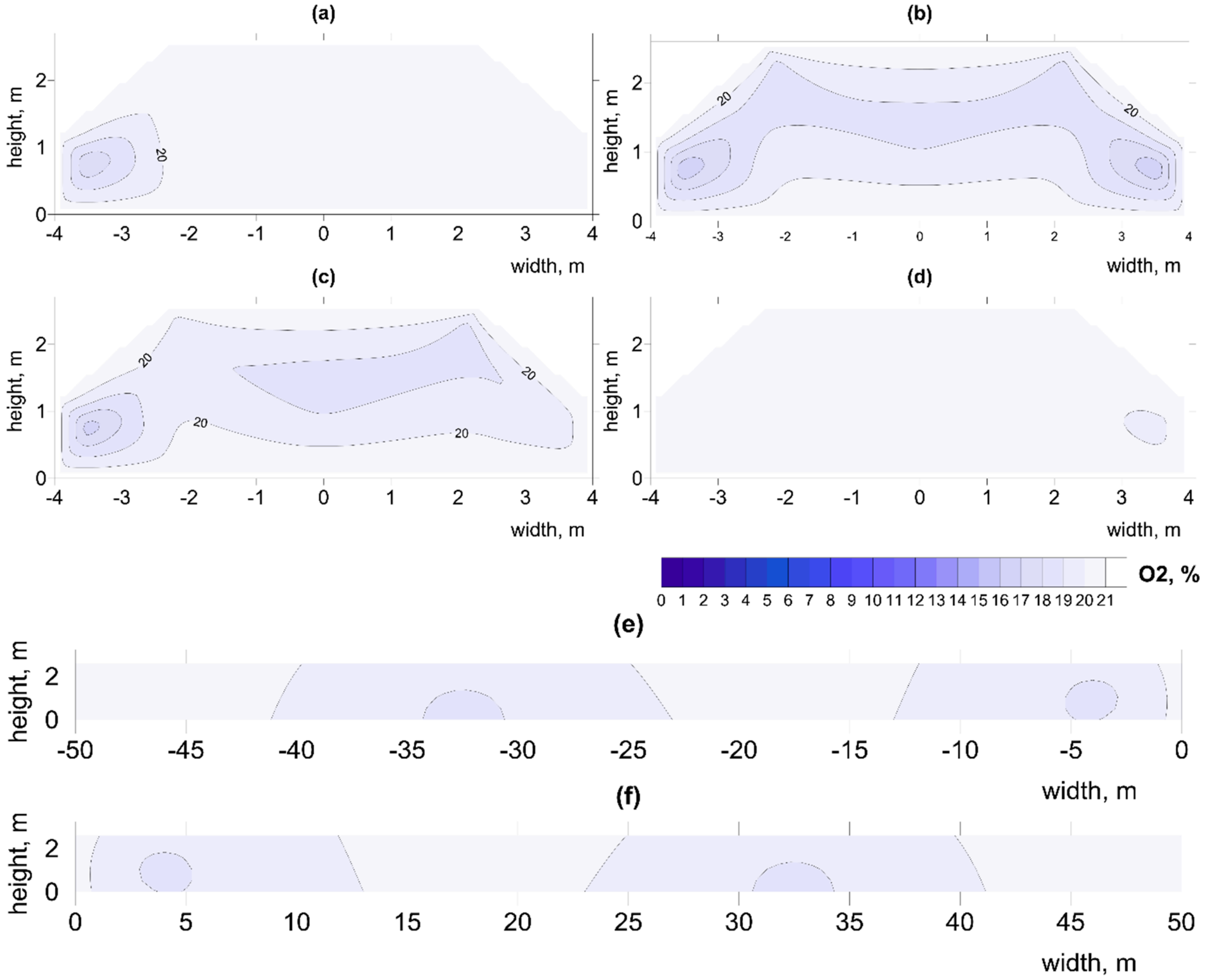

Figure S78. O2 distribution in the pile C1, day 35.

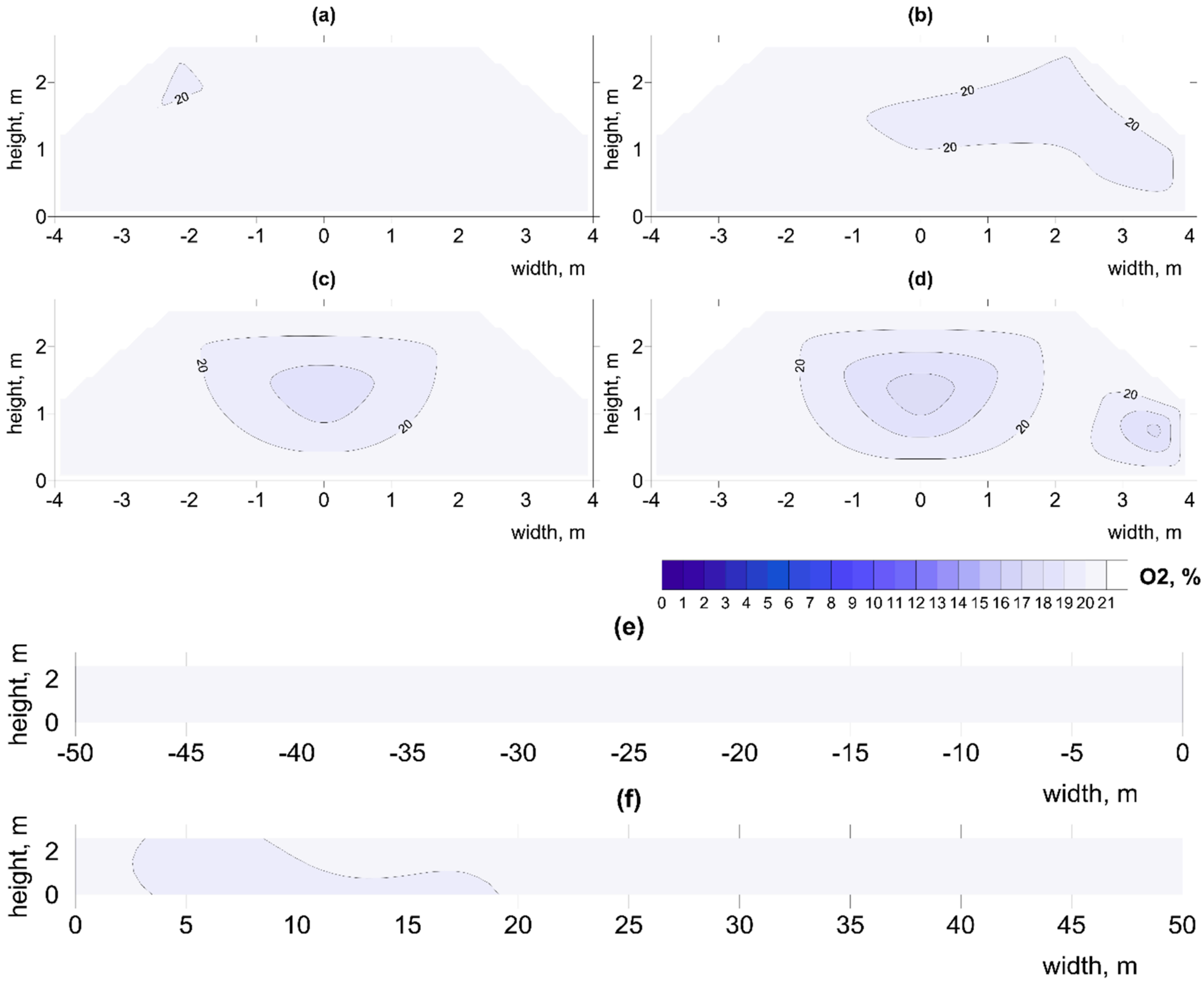

Figure S79. O2 distribution in the pile C1, day 42.

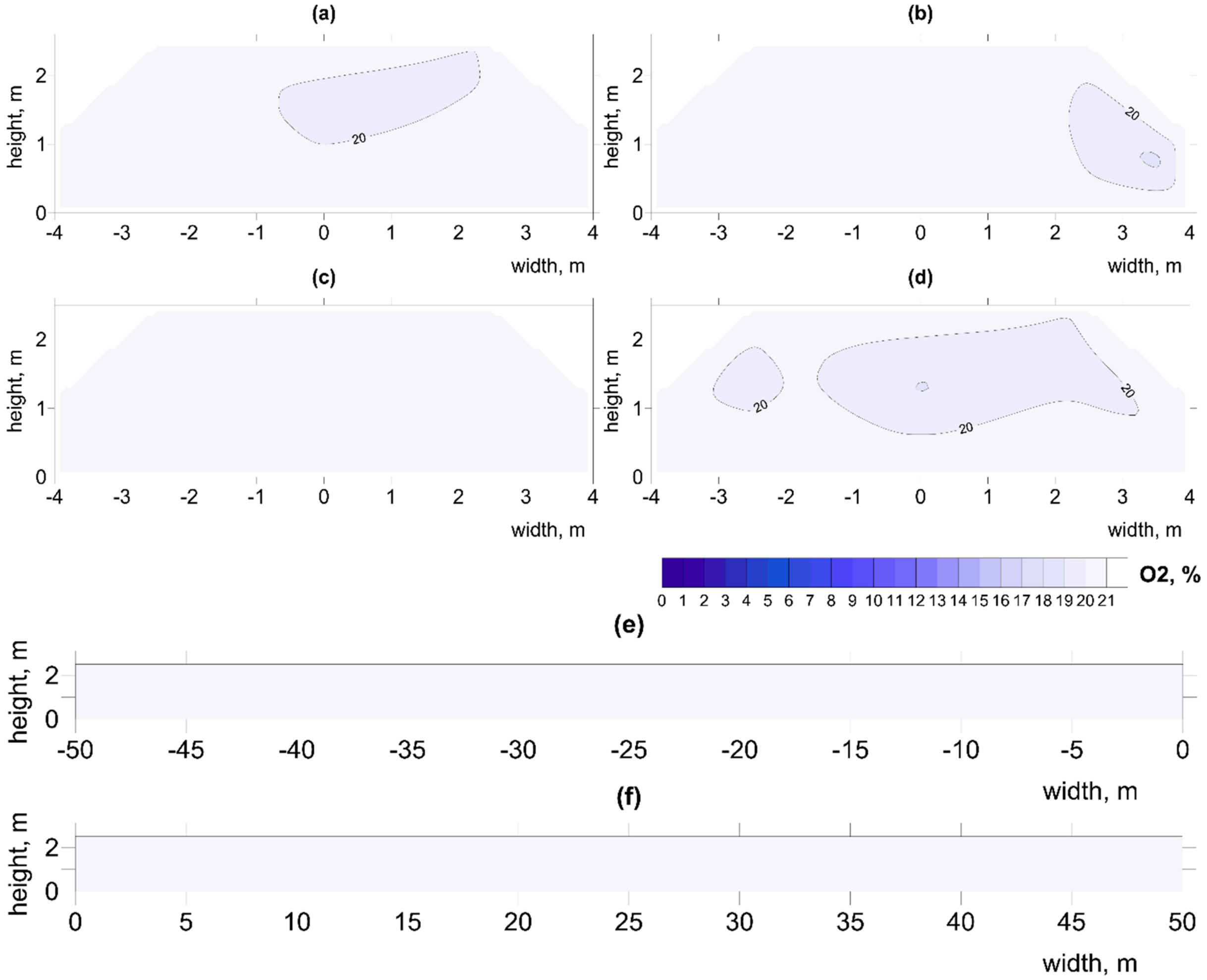

Figure S80. O2 distribution in the pile C2, day 1.

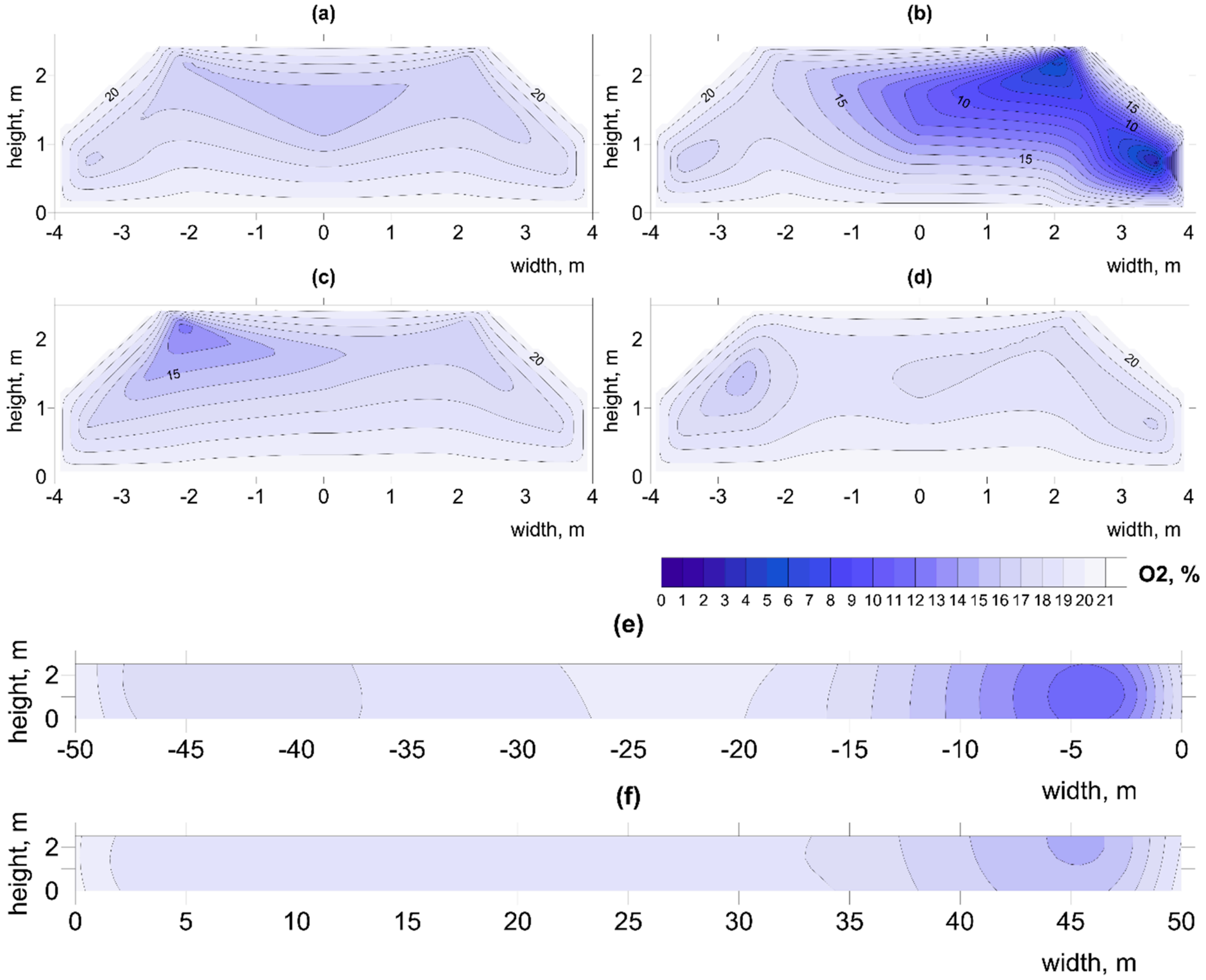

Figure S81. O2 distribution in the pile C2, day 9.

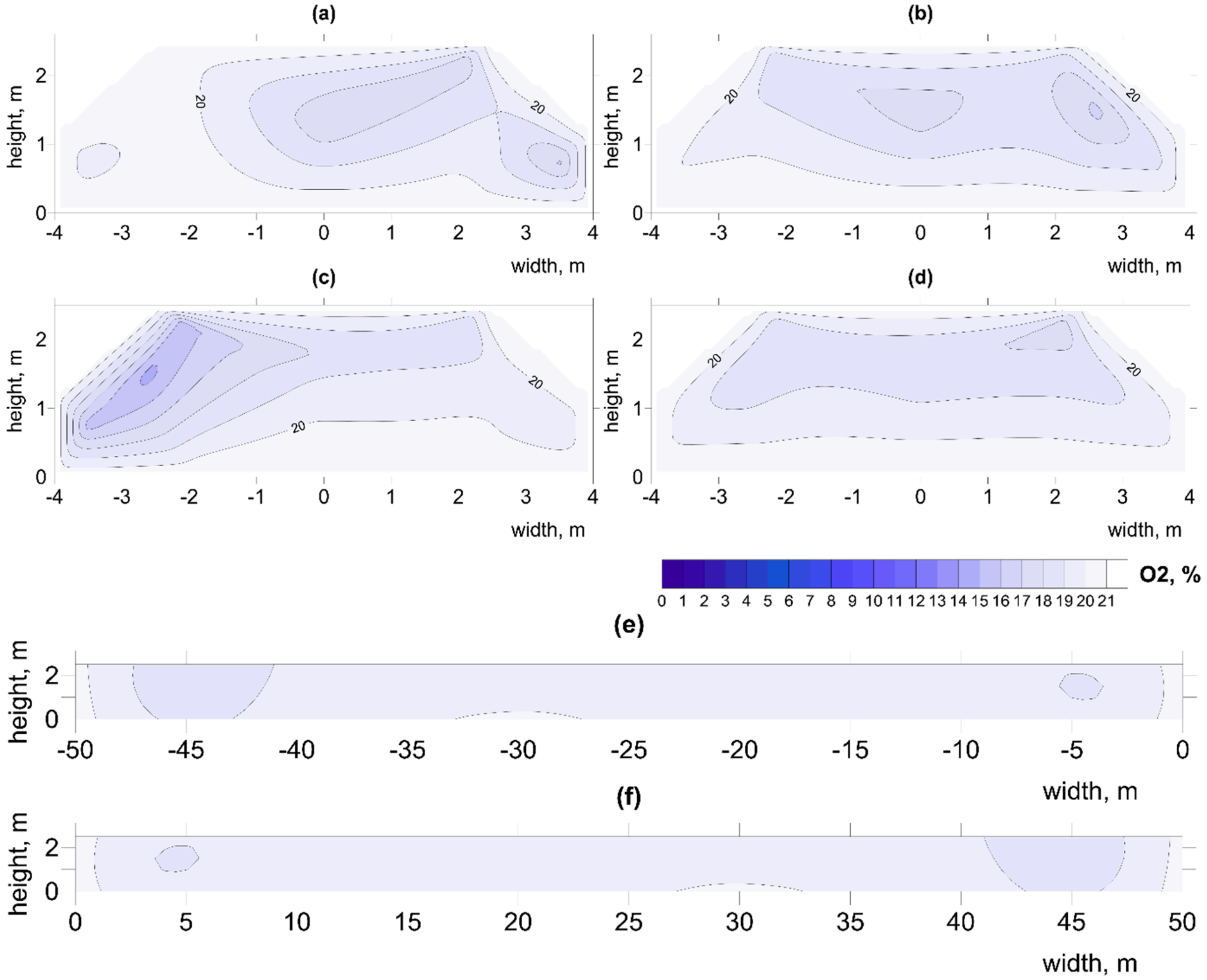

Figure S82. O2 distribution in the pile C2, day 21.

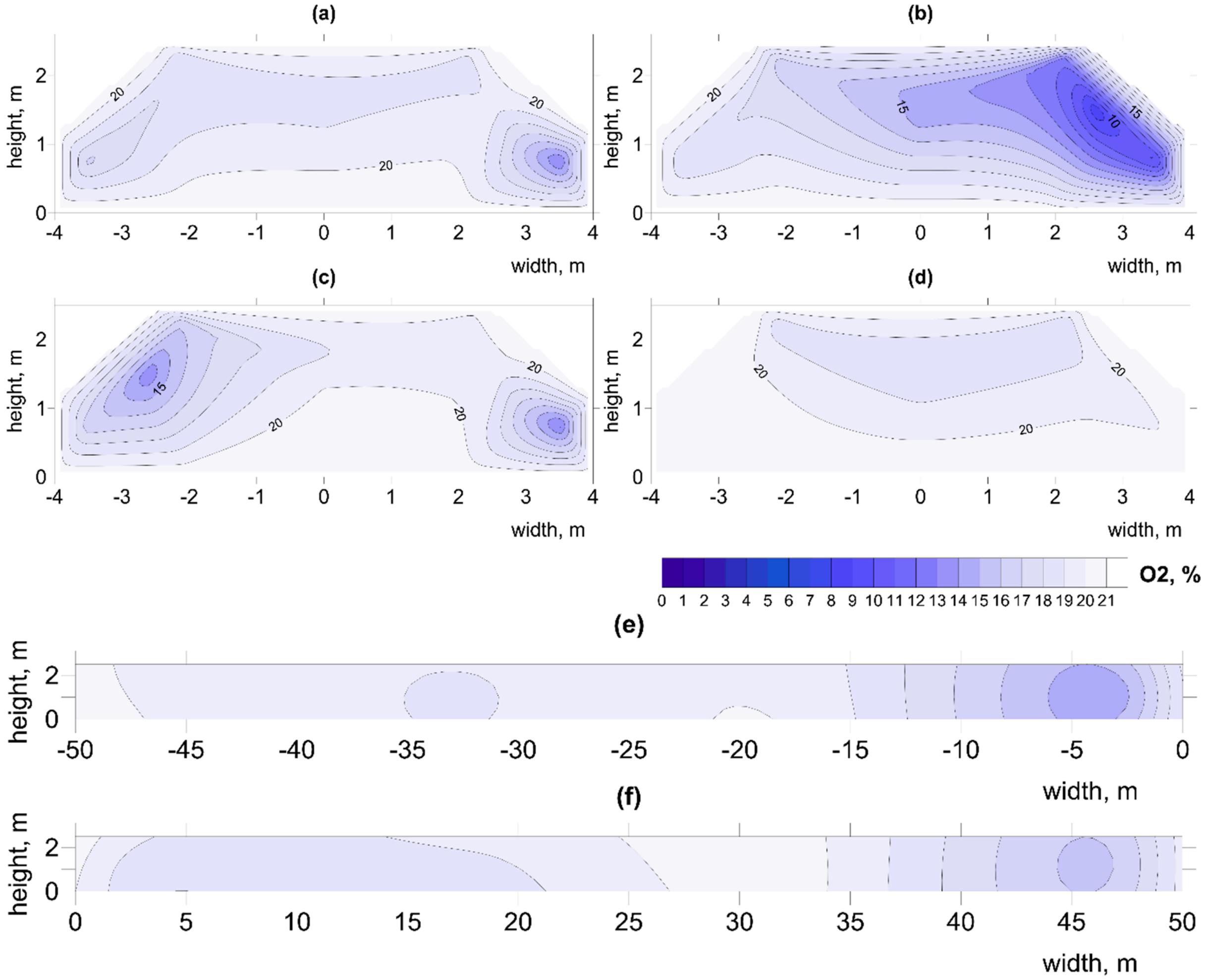

Figure S83. O2 distribution in the pile C2, day 28.

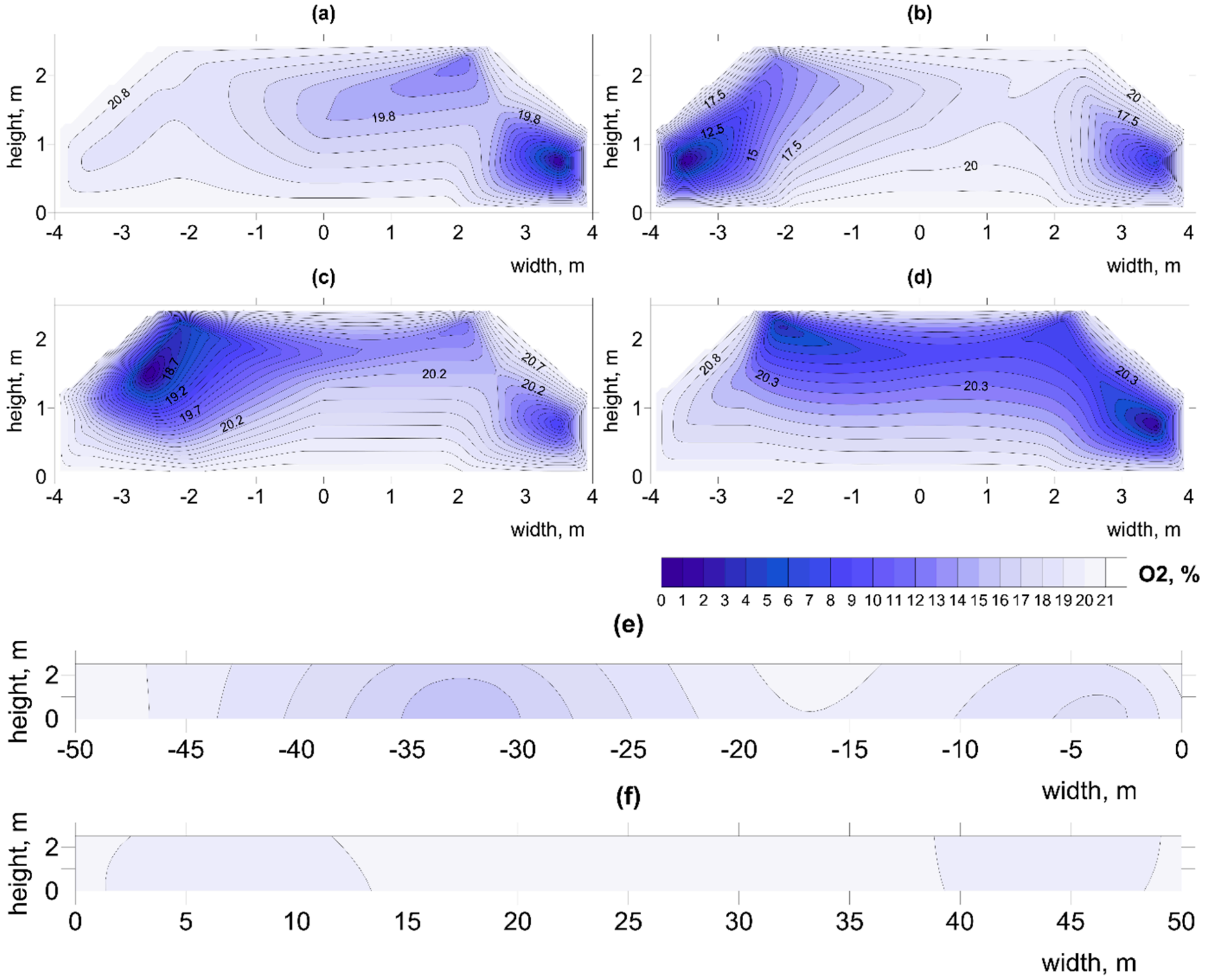

Figure S84. O2 distribution in the pile C2, day 35.

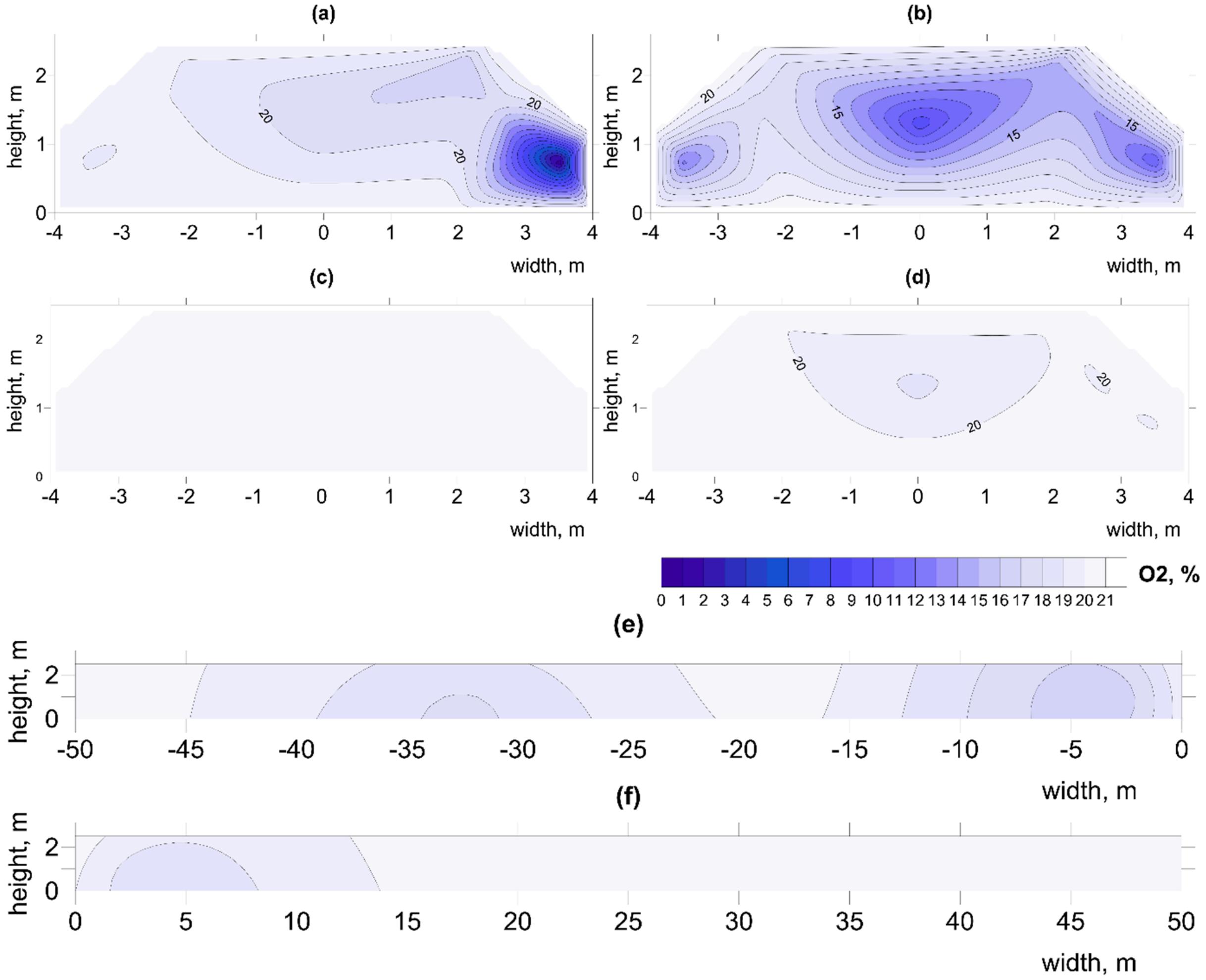

Figure S85. O2 distribution in the pile C2, day 42.

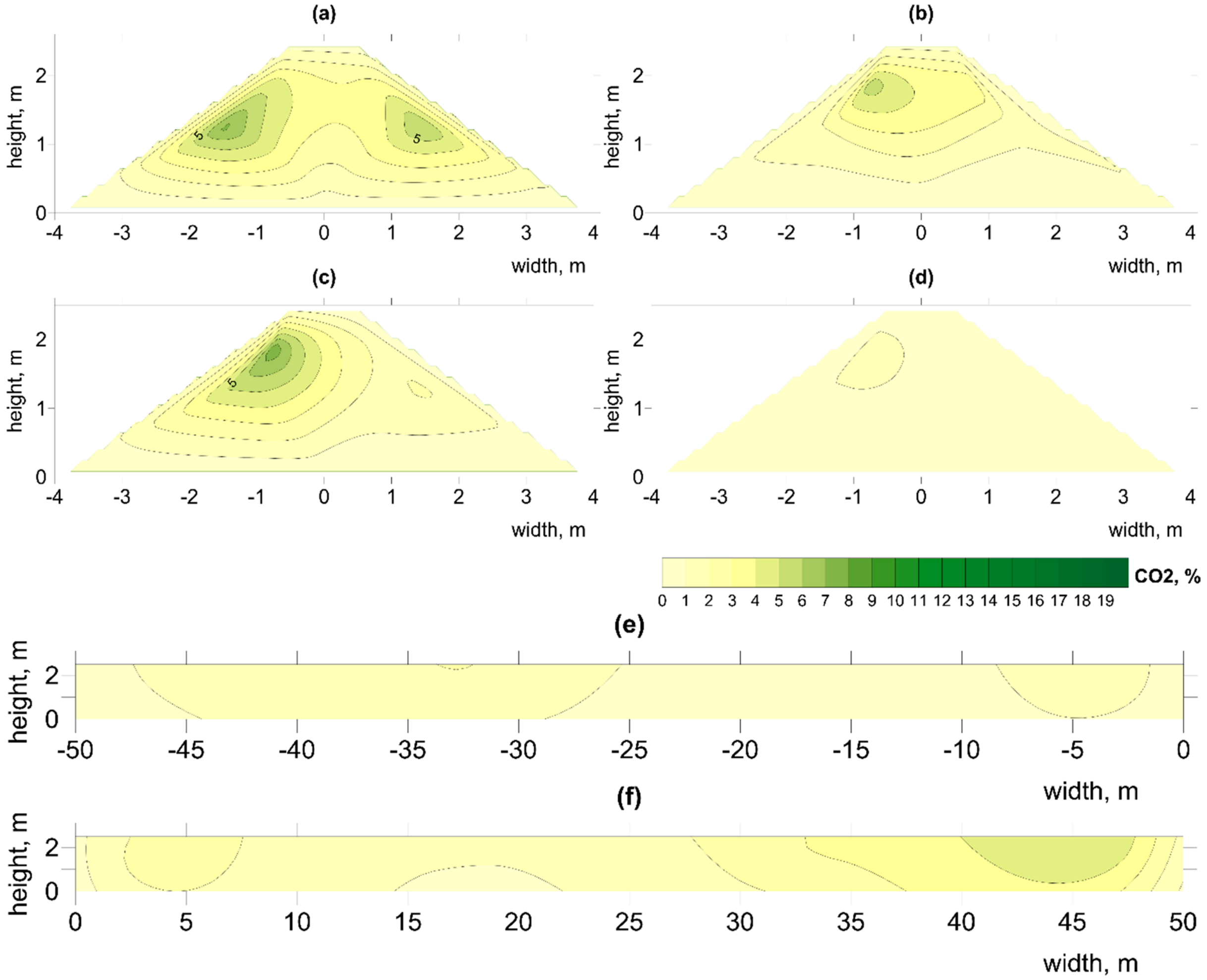

Figure S86. CO2 distribution in the pile A1, day 1.

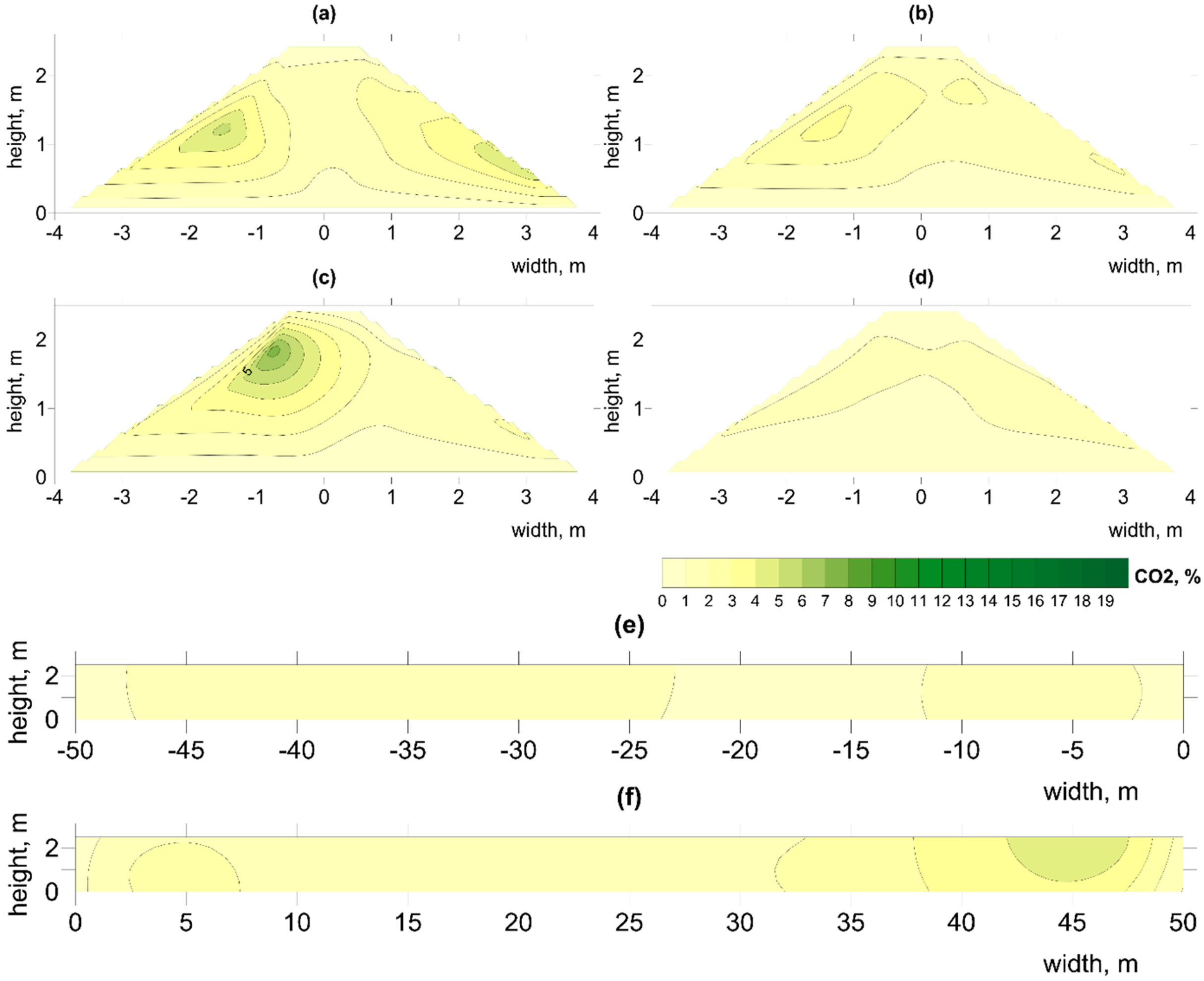

Figure S87. CO2 distribution in the pile A1, day 4.

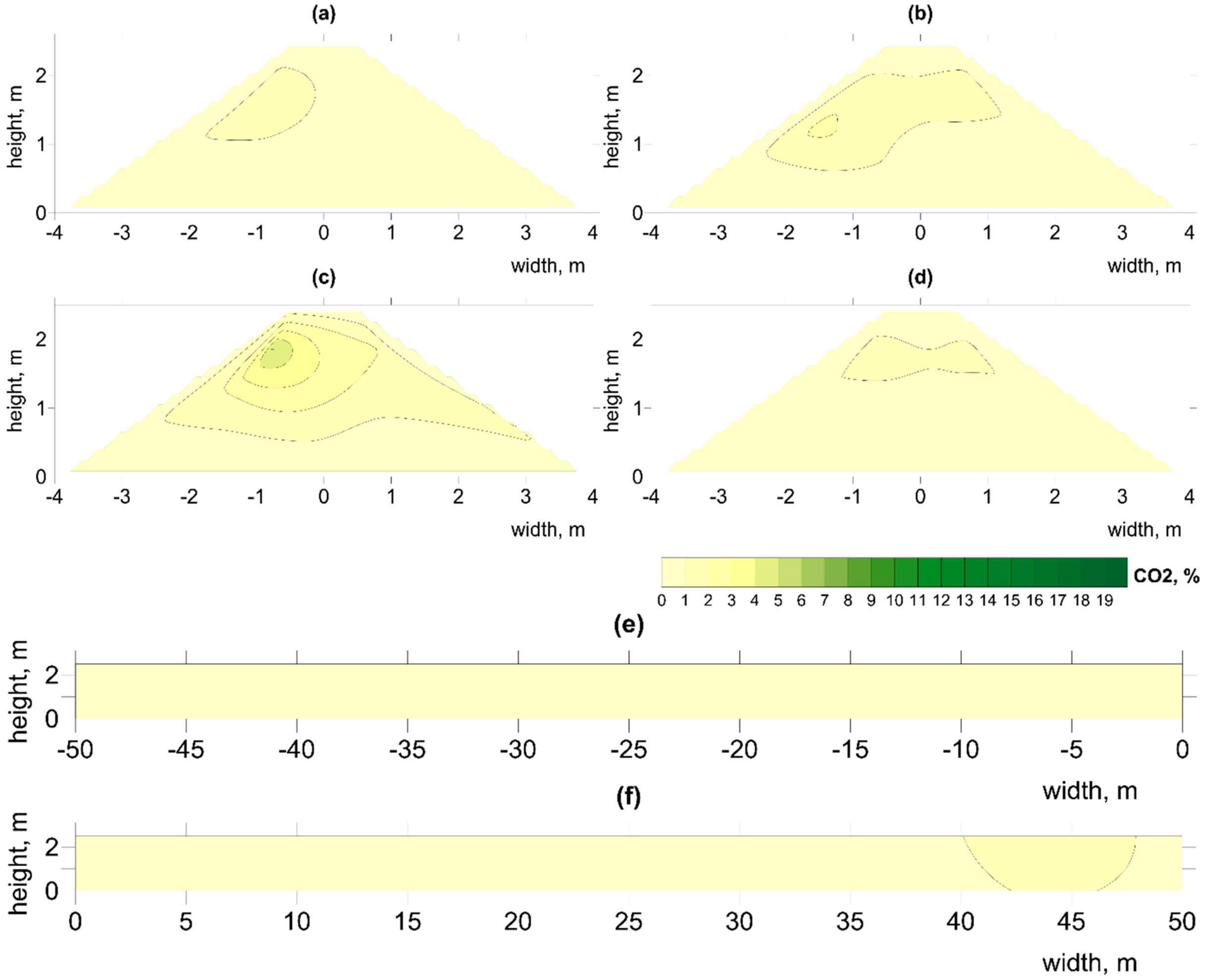

Figure S88. CO2 distribution in the pile A1, day 14.

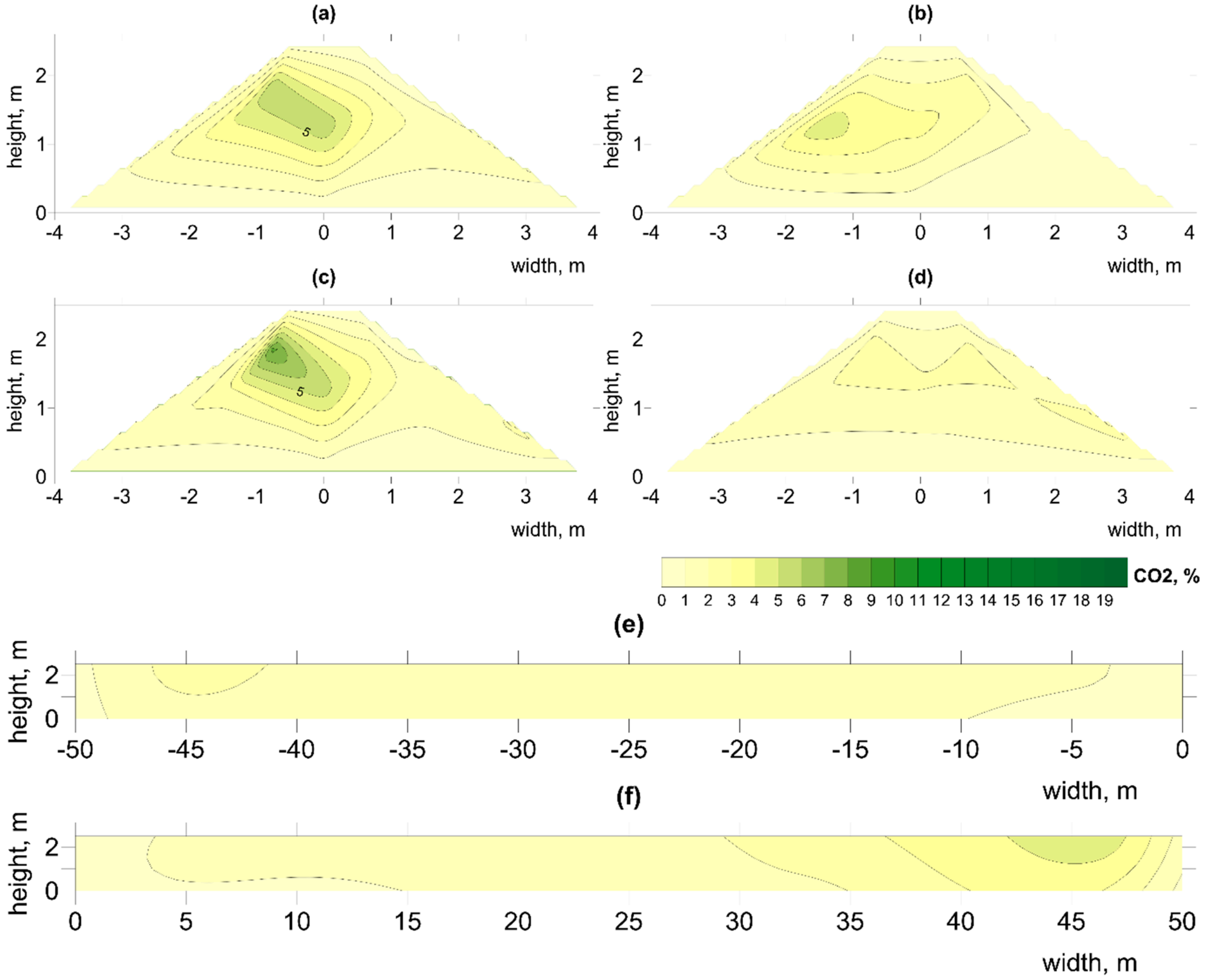

Figure S89. CO2 distribution in the pile A1, day 20.

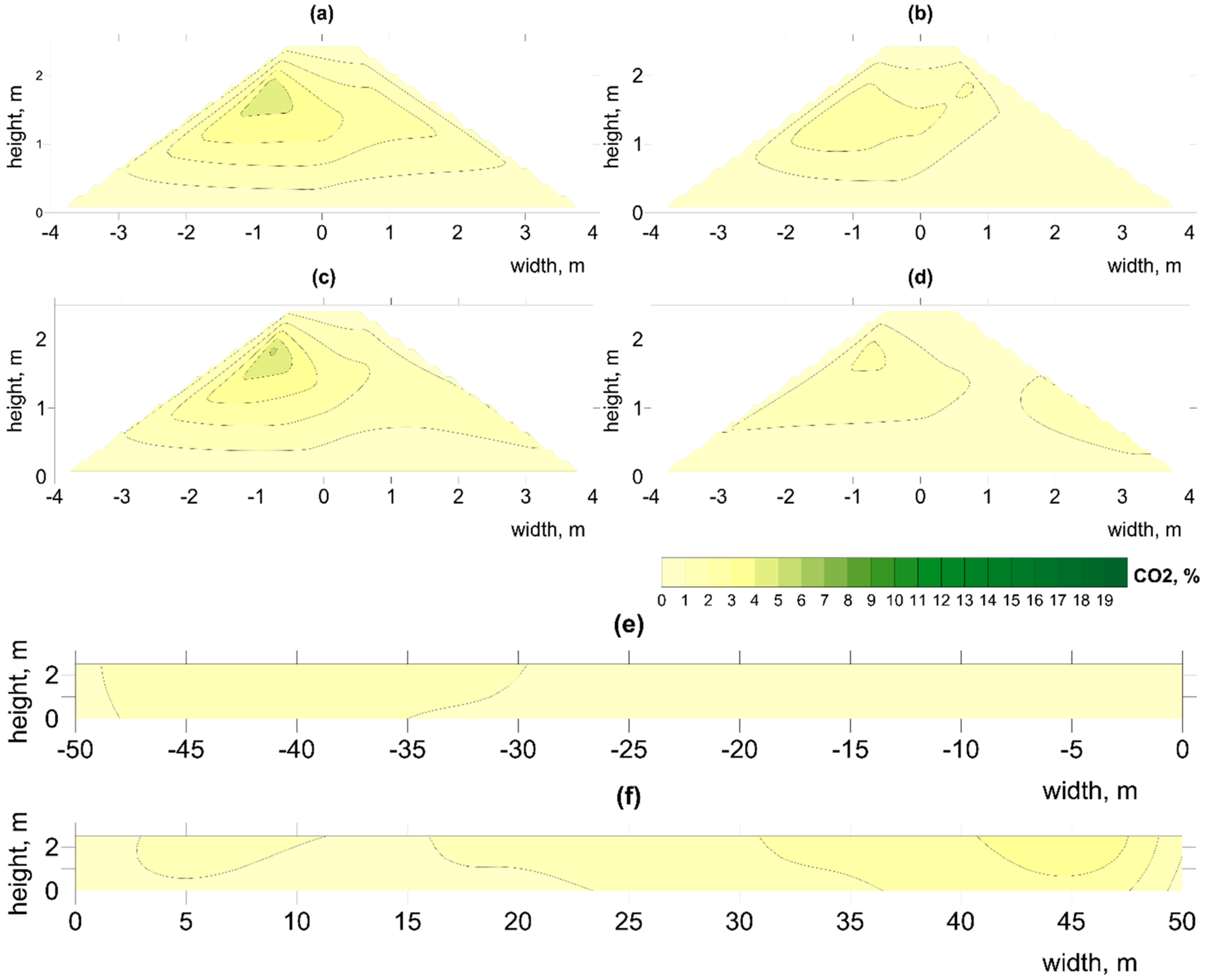

Figure S90. CO2 distribution in the pile A1, day 28.

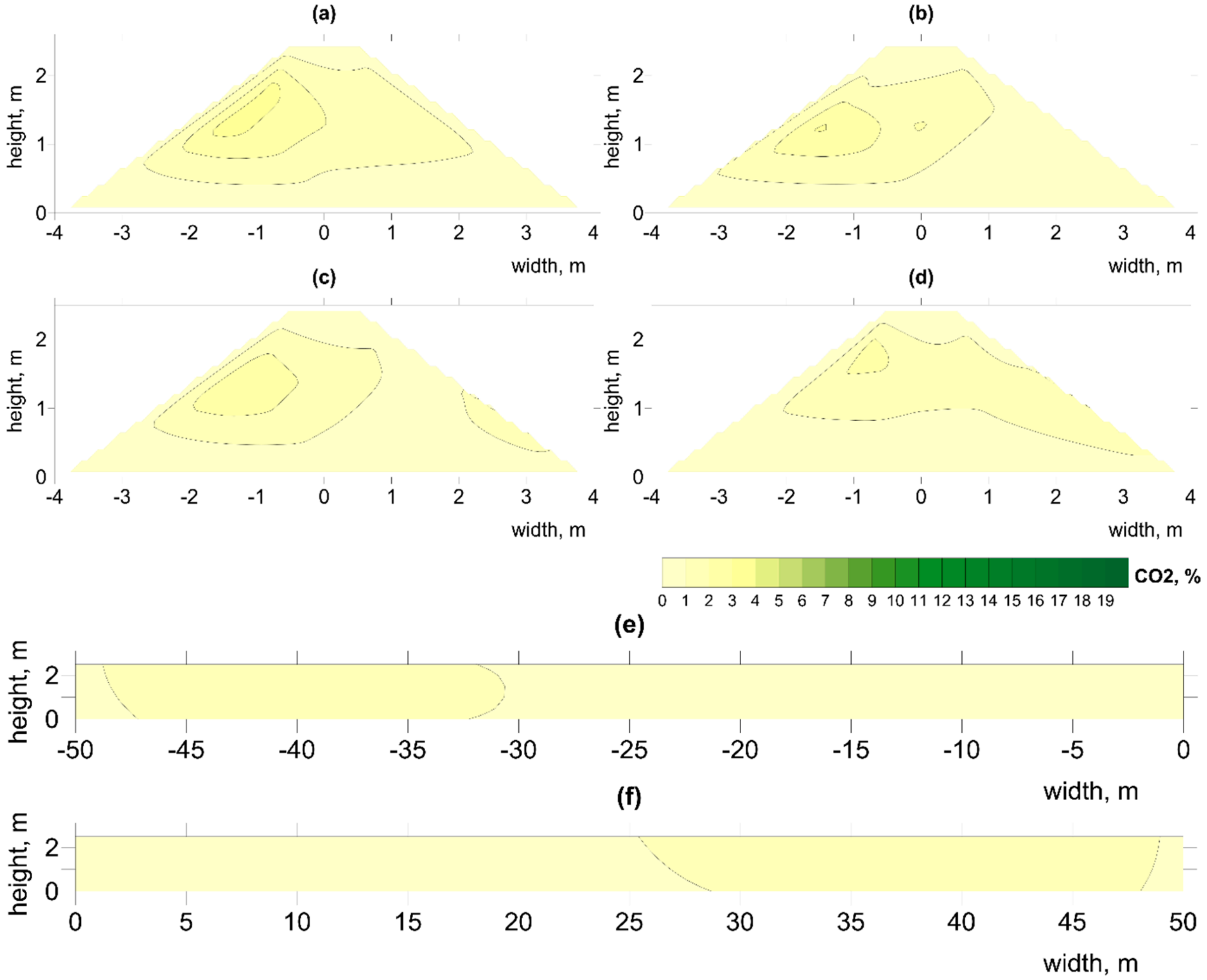

Figure S91. CO2 distribution in the pile A1, day 35.

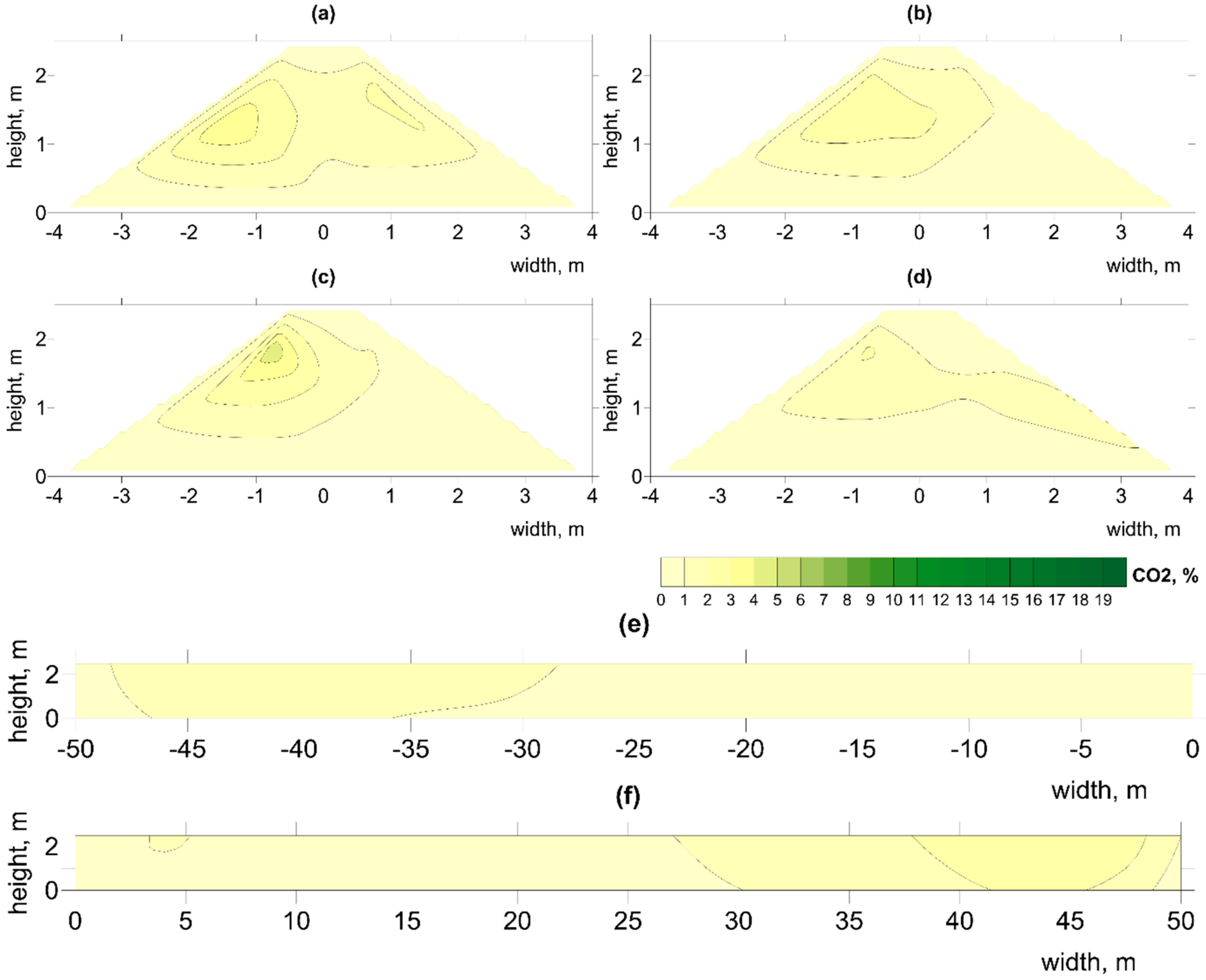

Figure S92. CO2 distribution in the pile A1, day 41.

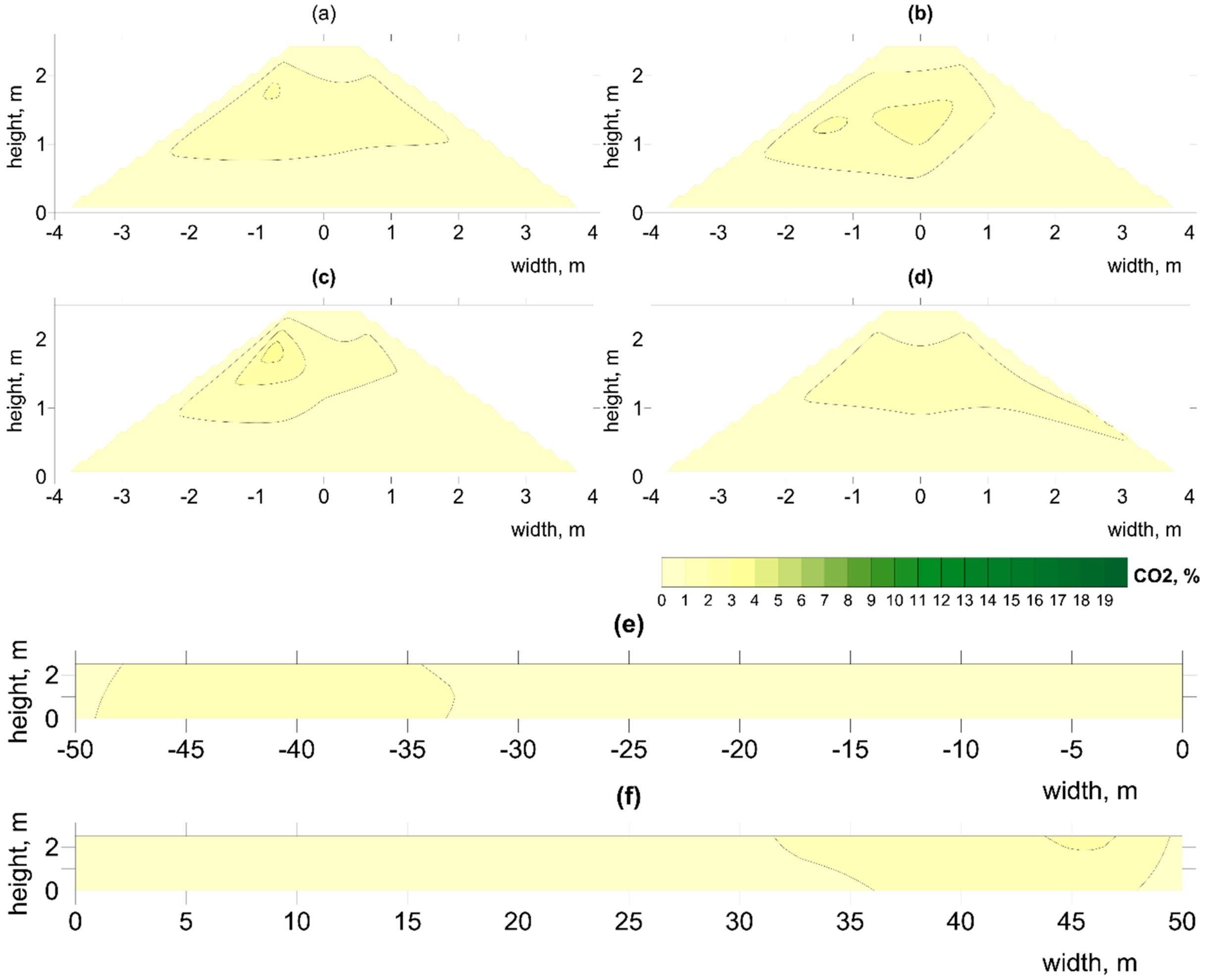

Figure S93. CO2 distribution in the pile A1, day 50.

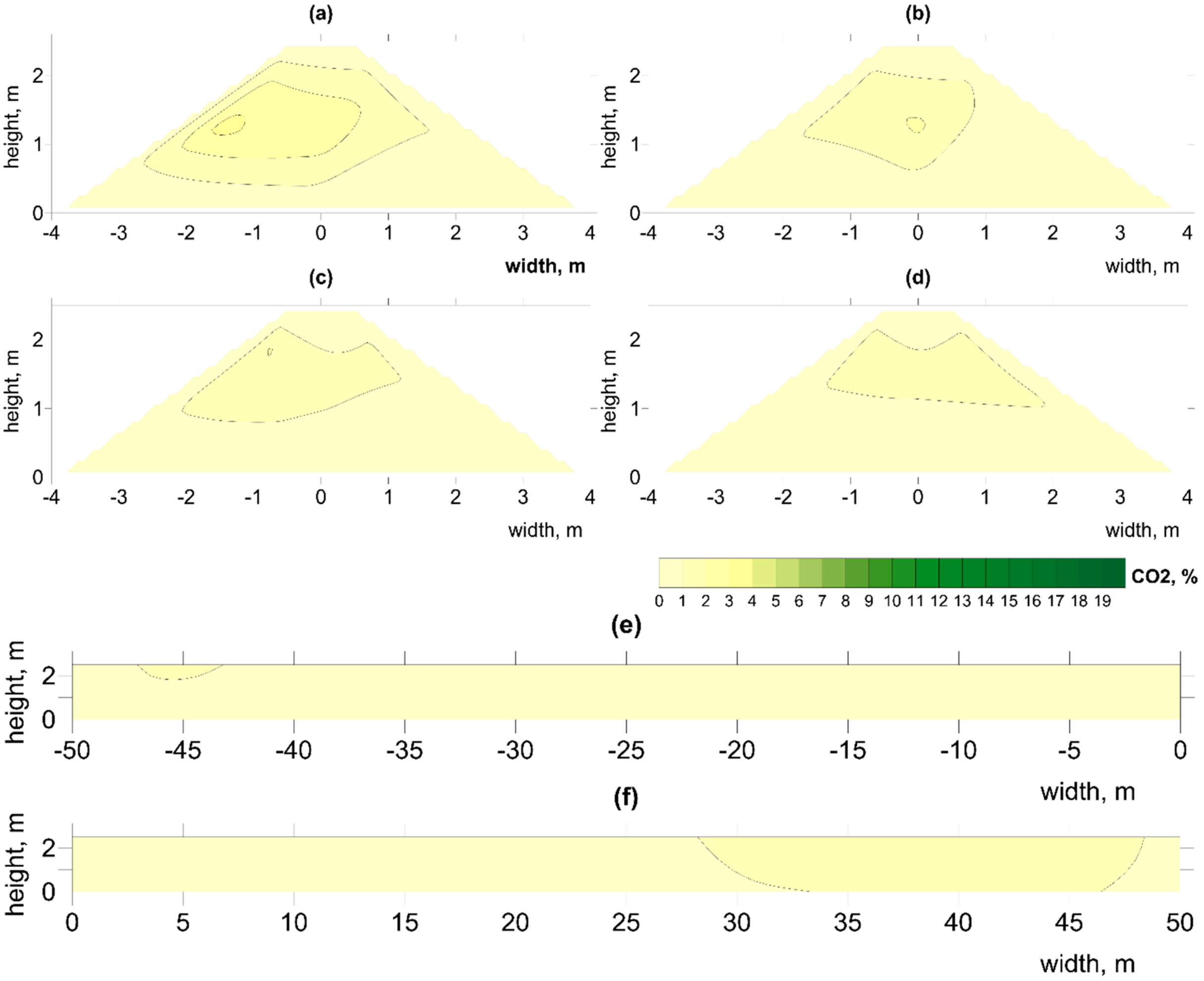

Figure S94. CO2 distribution in the pile A1, day 60.

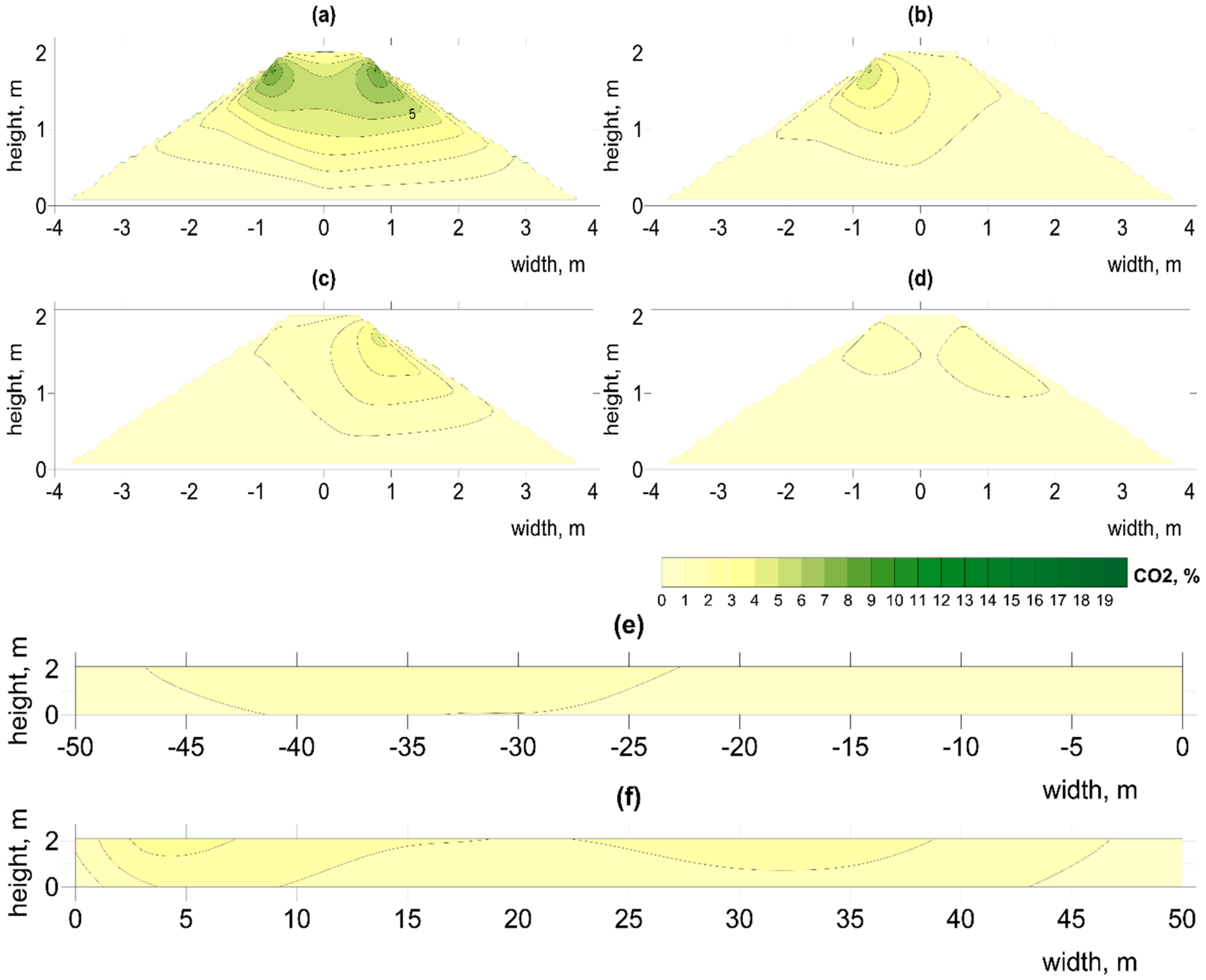

Figure S95. CO2 distribution in the pile A2, day 1.

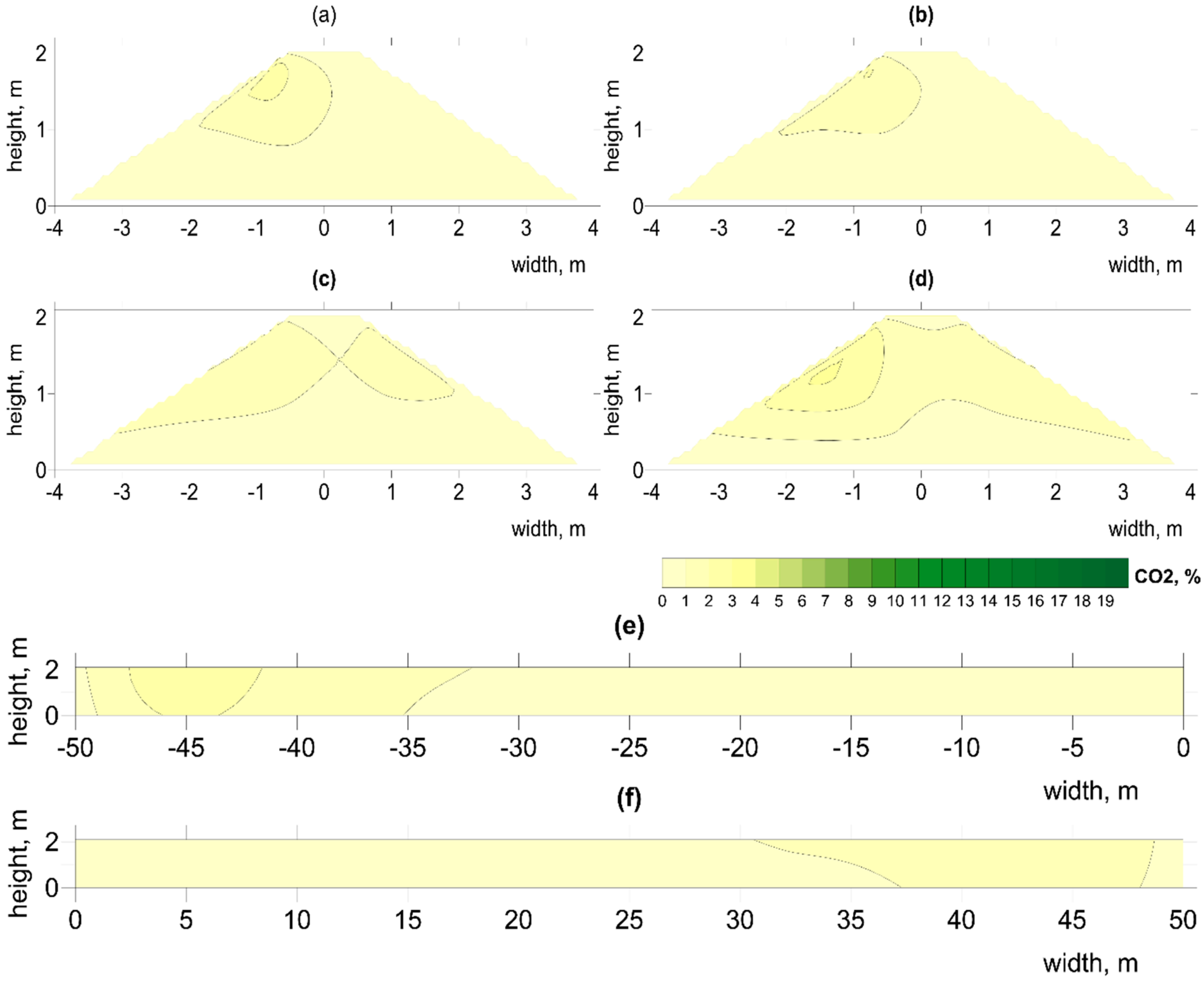

Figure S96. CO2 distribution in the pile A2, day 11.

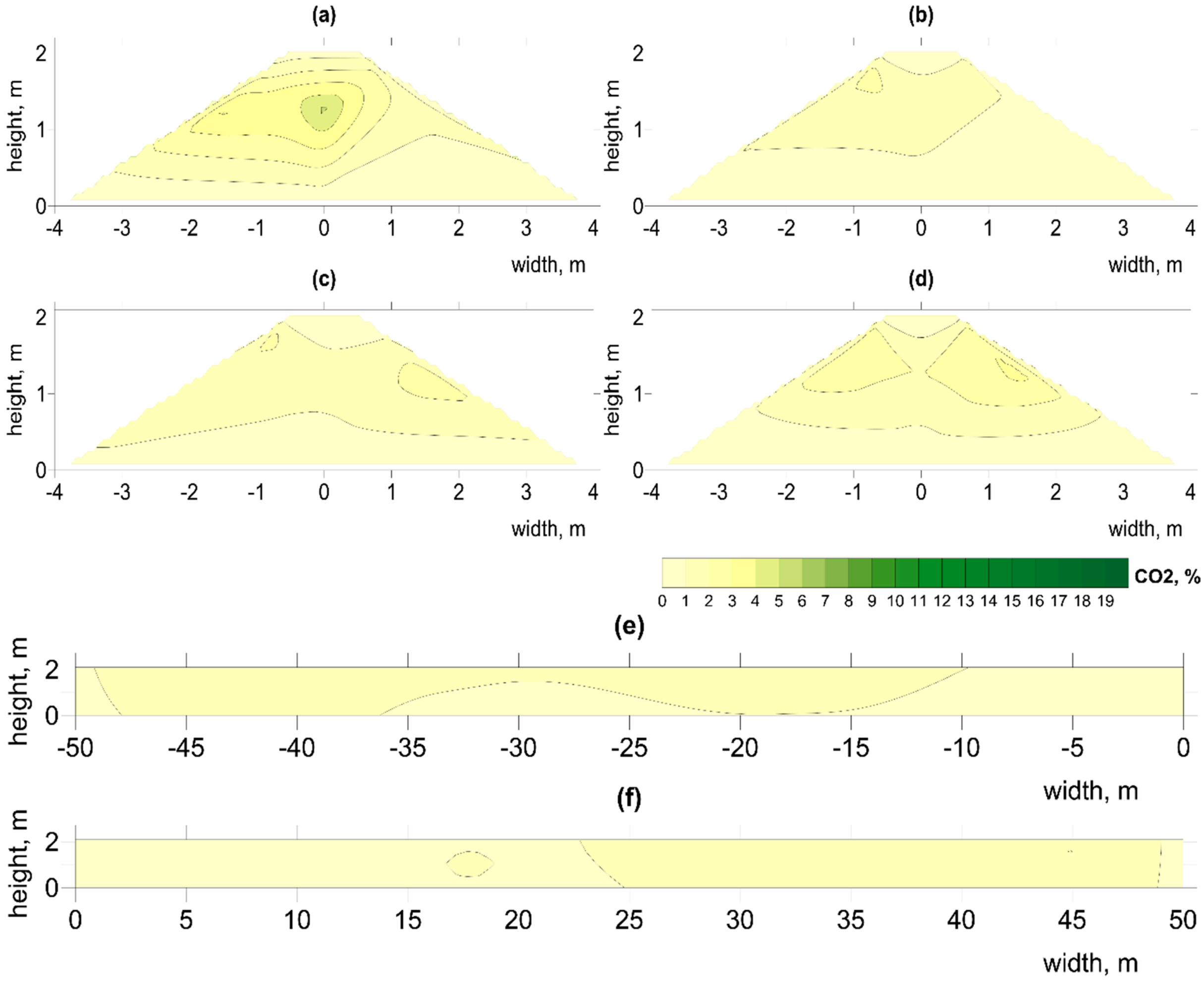

Figure S97. CO2 distribution in the pile A2, day 17.

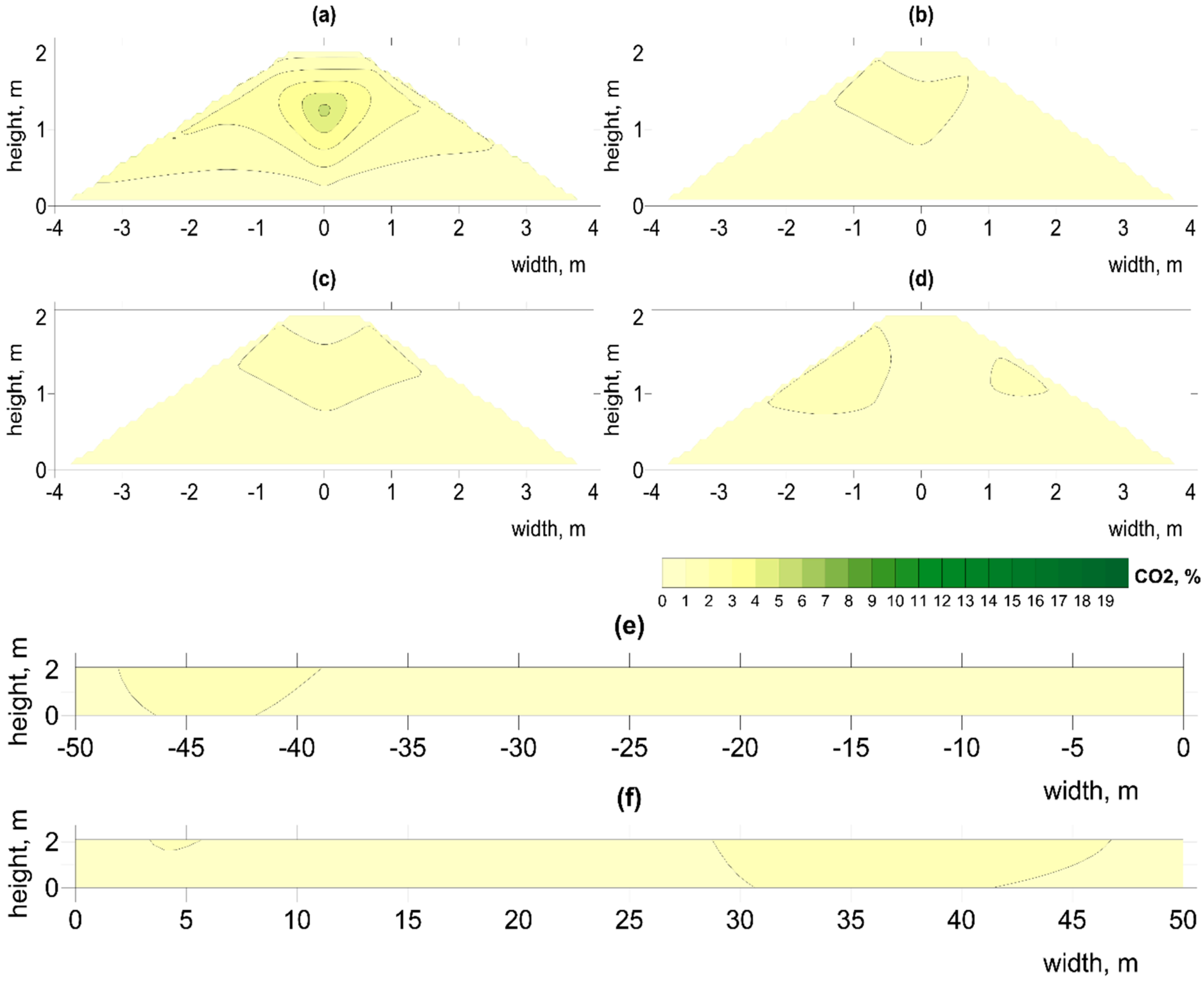

Figure S98. CO2 distribution in the pile A2, day 25.

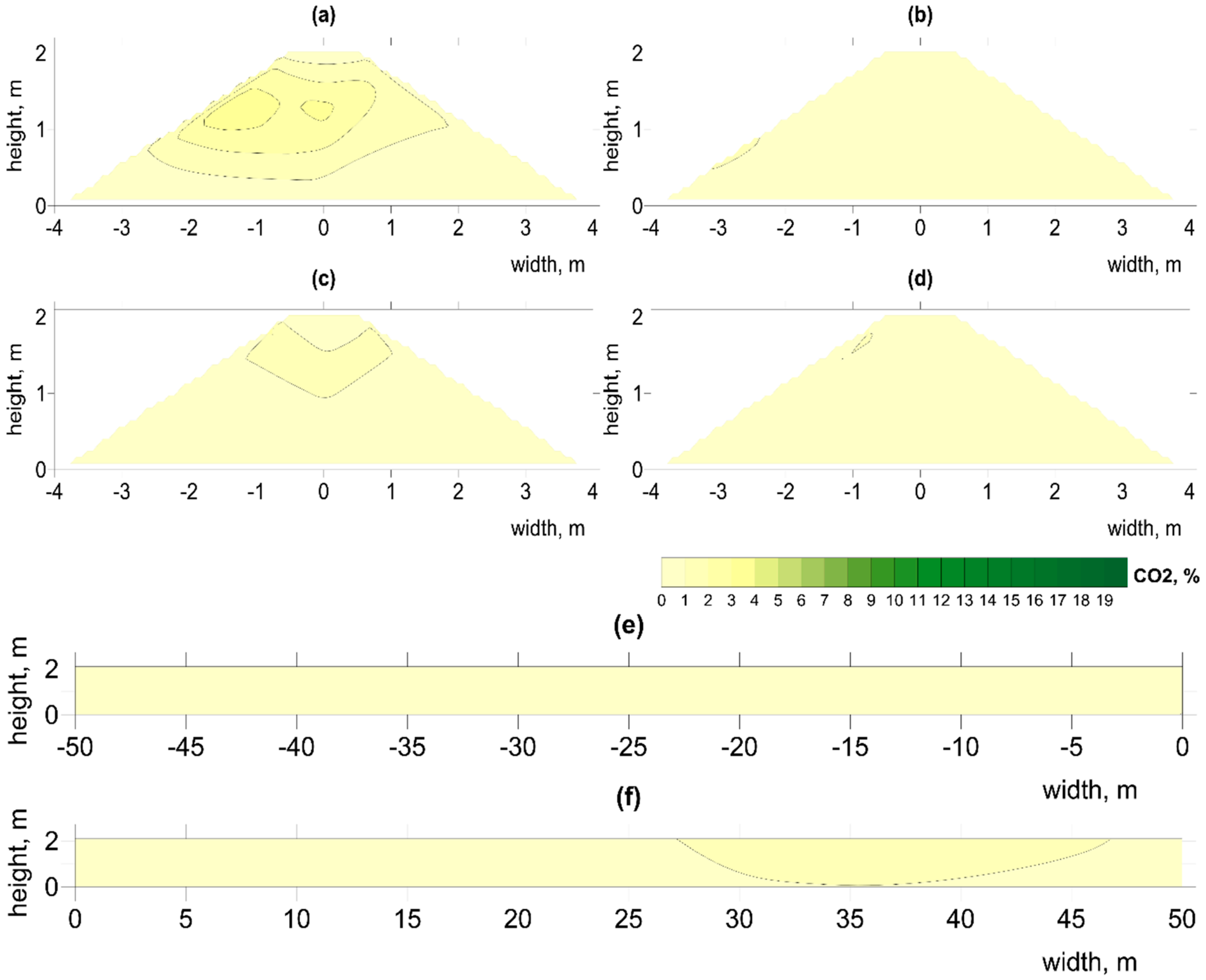

Figure S99. CO2 distribution in the pile A2, day 32.

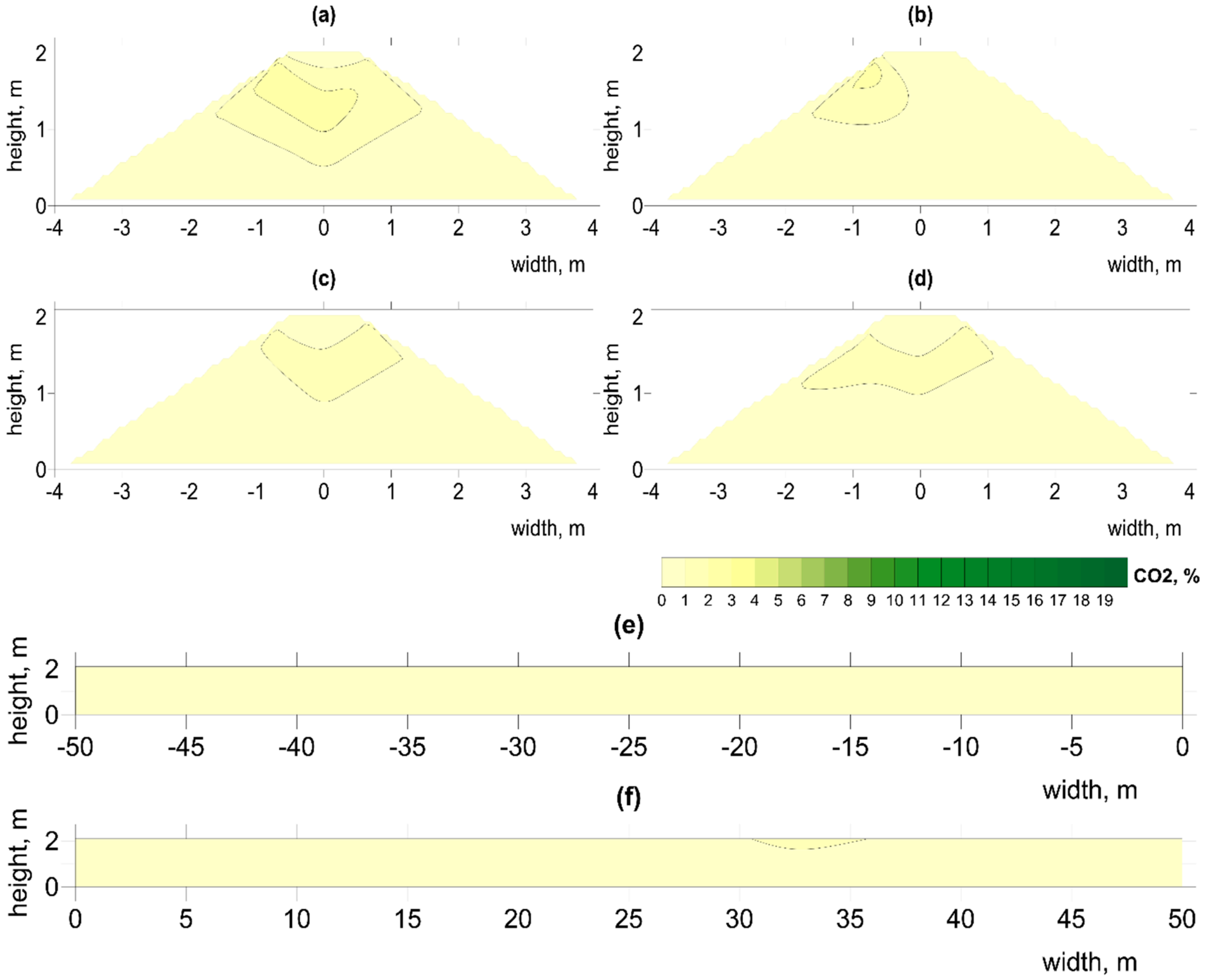

Figure S100. CO2 distribution in the pile A2, day 38.

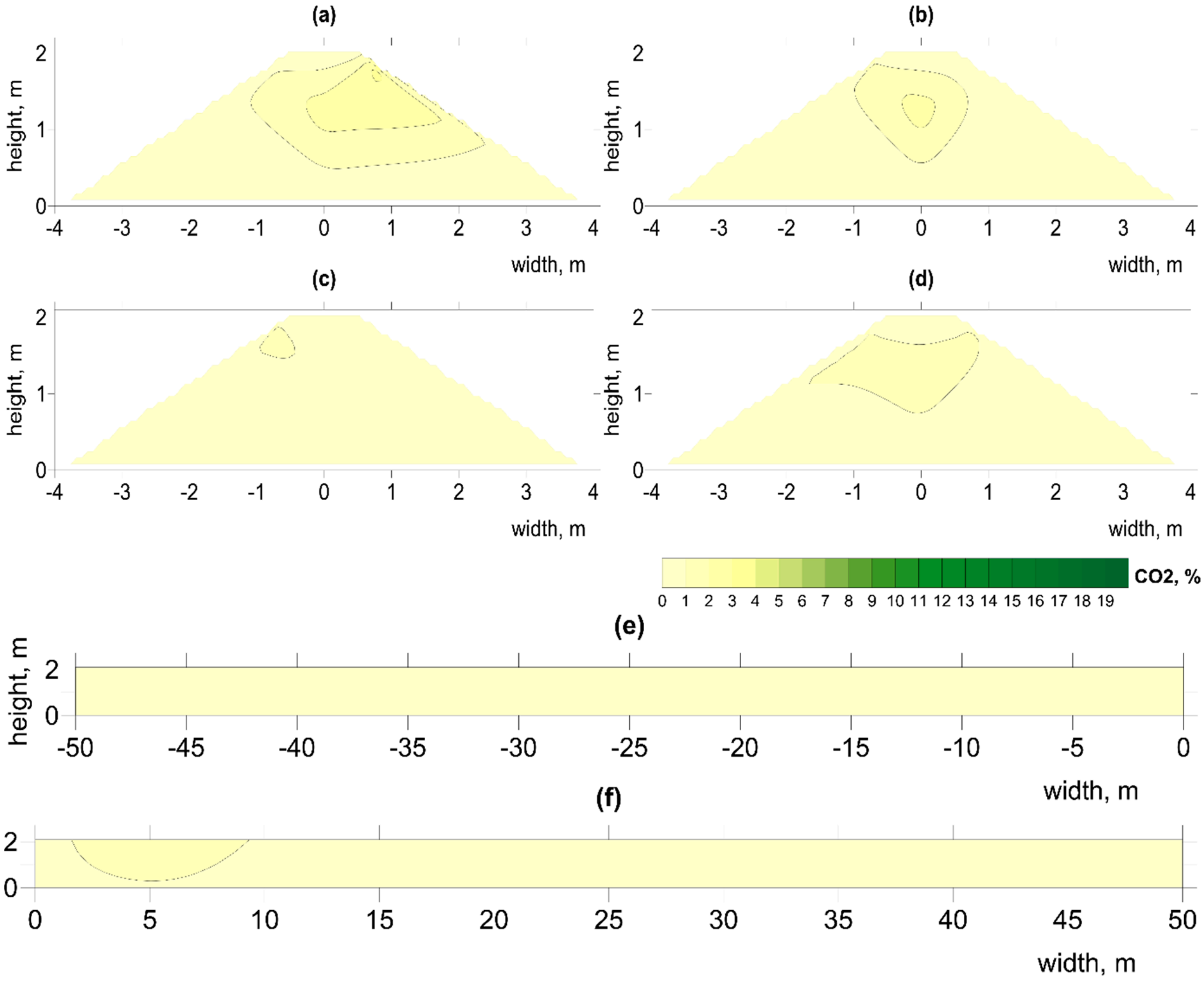

Figure S101. CO2 distribution in the pile A2, day 47.

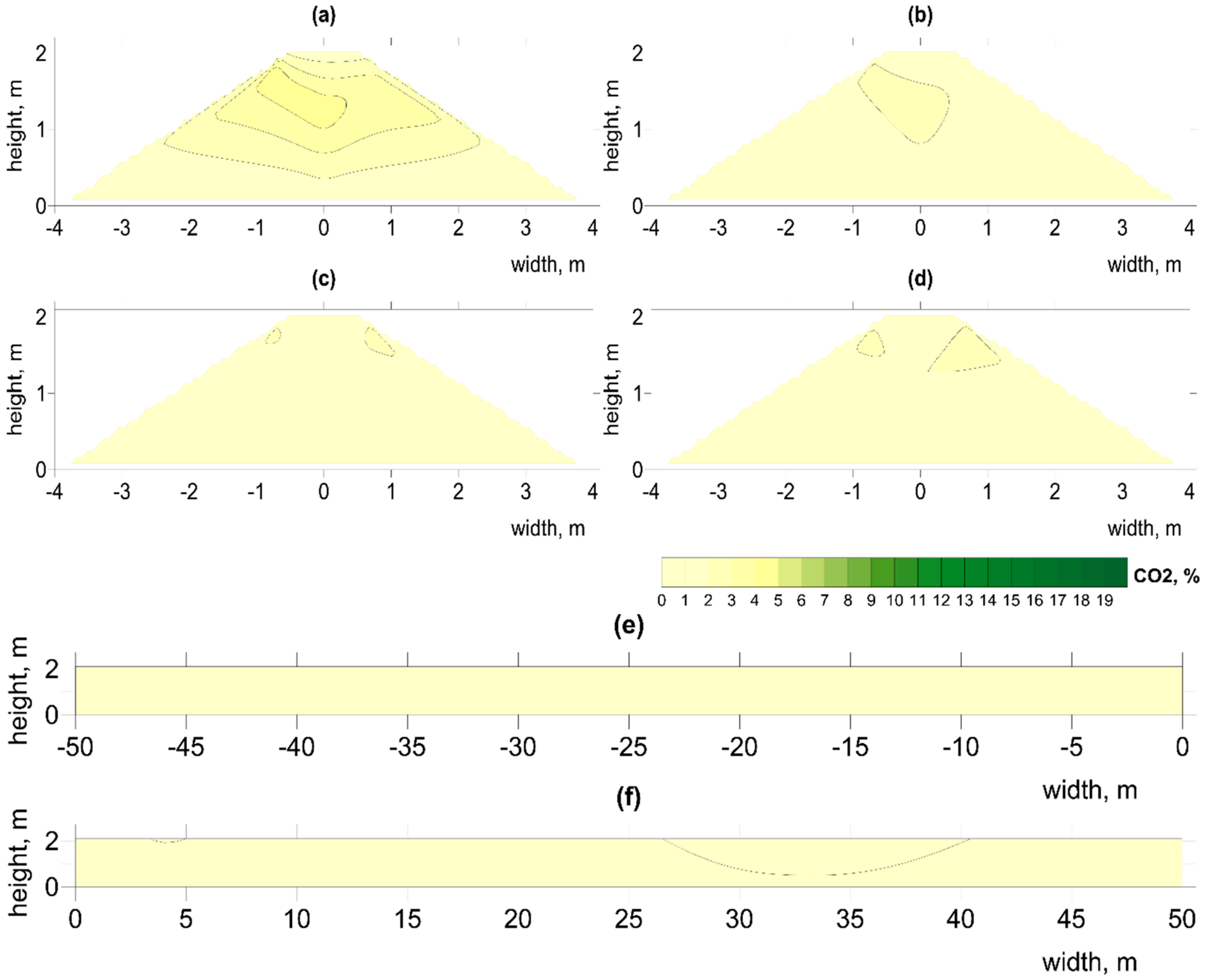

Figure S102. CO2 distribution in the pile A2, day 57.

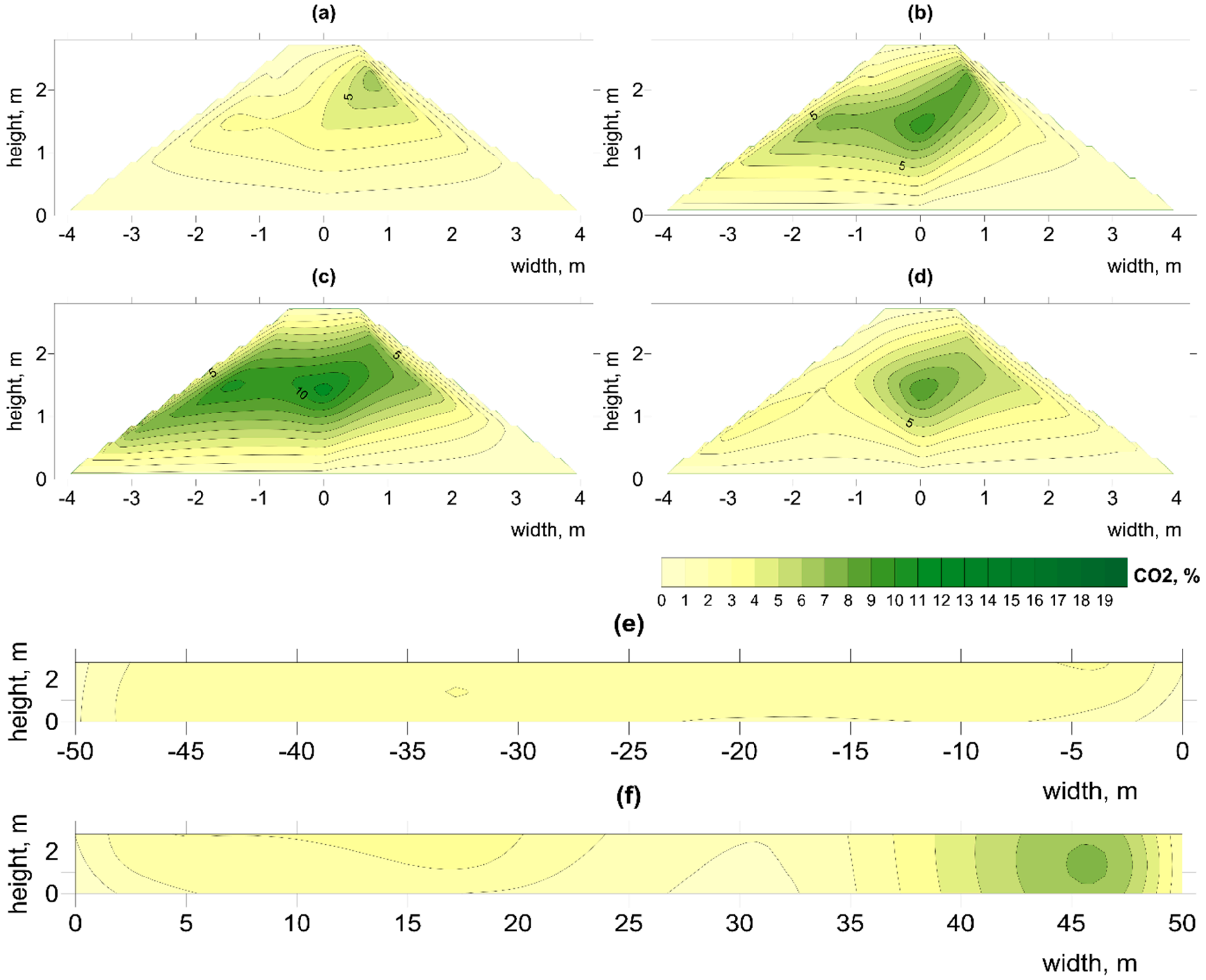

Figure S103. CO2 distribution in the pile B1, day 1.

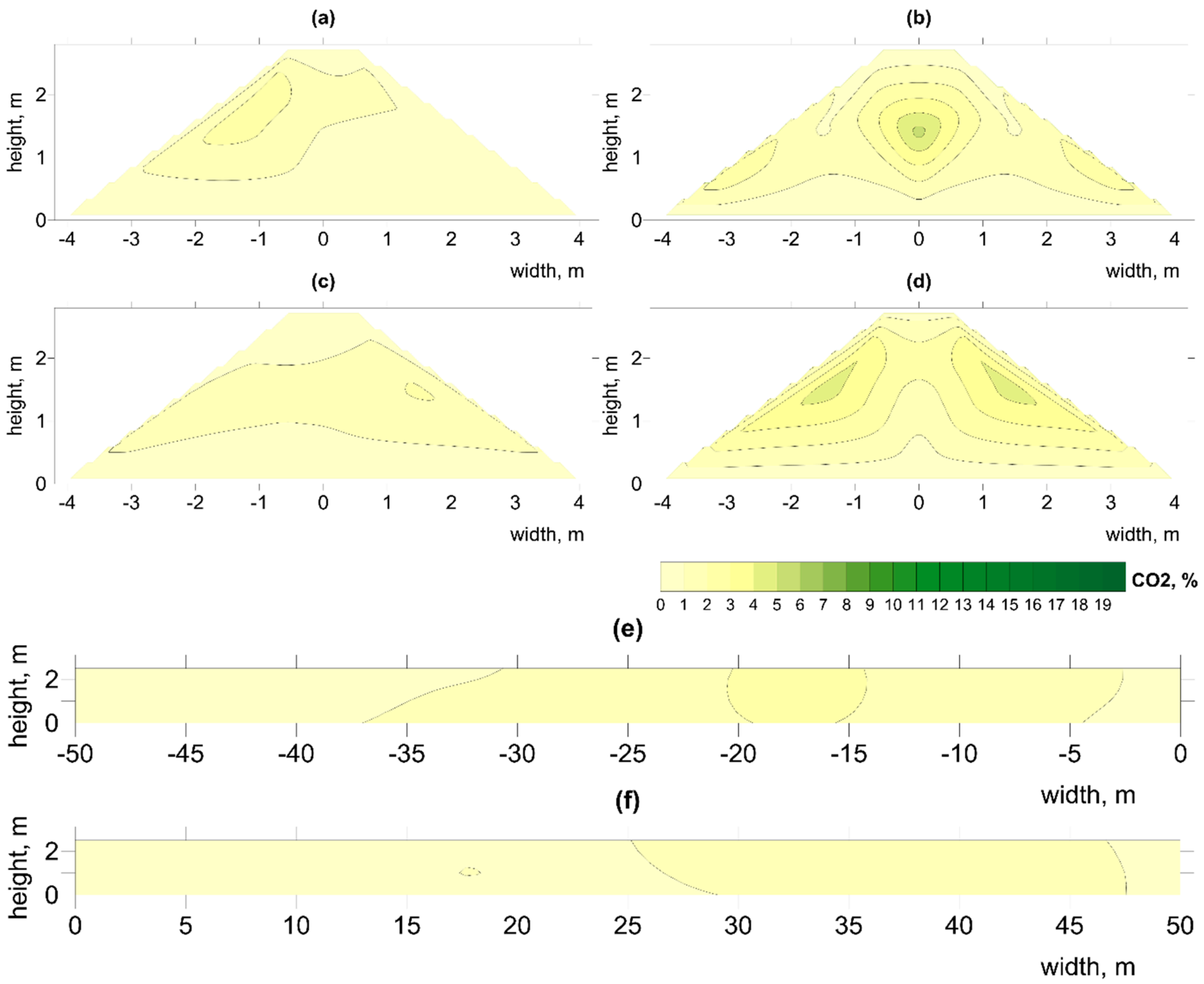

Figure S104. CO2 distribution in the pile B1, day 9.

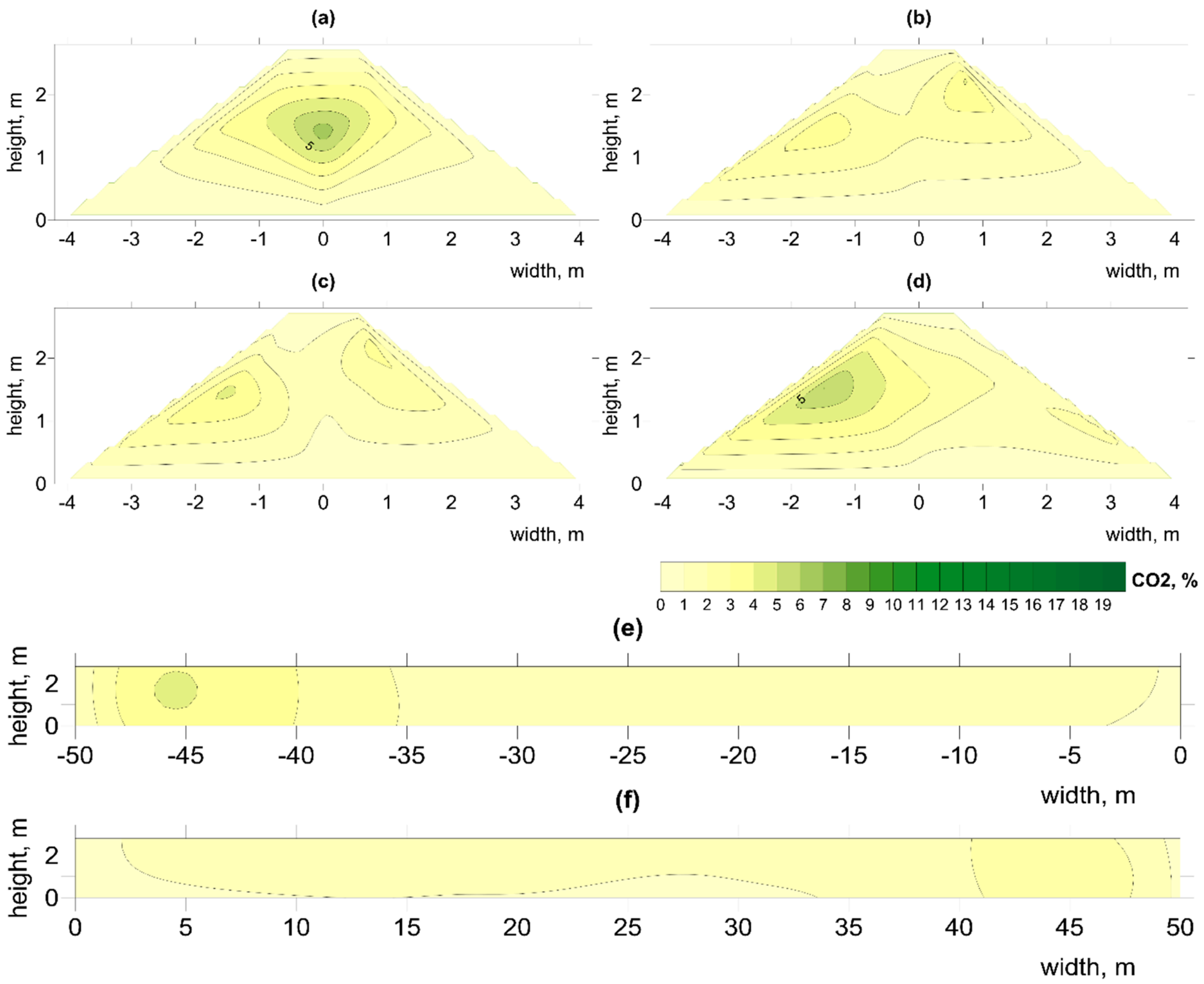

Figure S105. CO2 distribution in the pile B1, day 16.

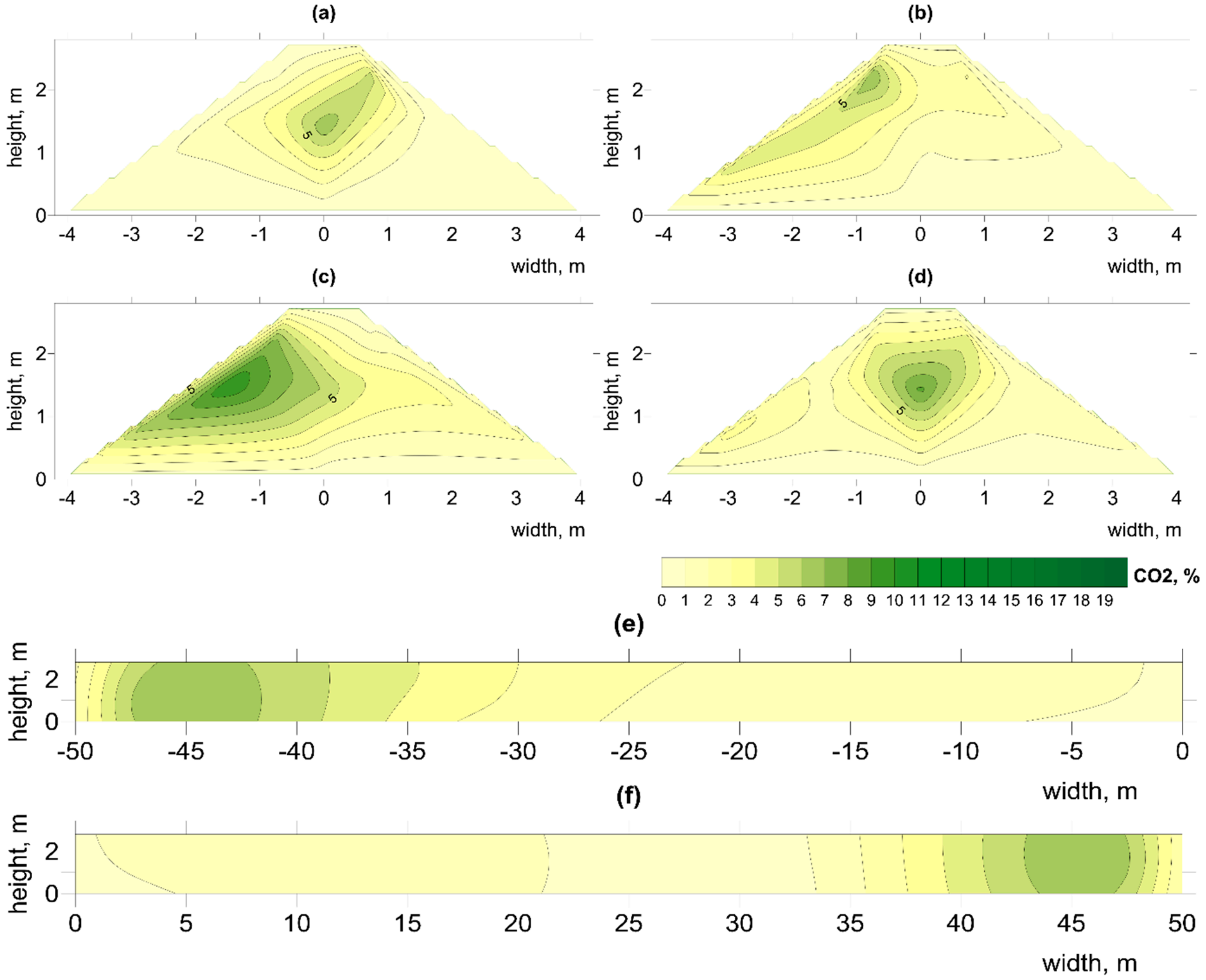

Figure S106. CO2 distribution in the pile B1, day 31.

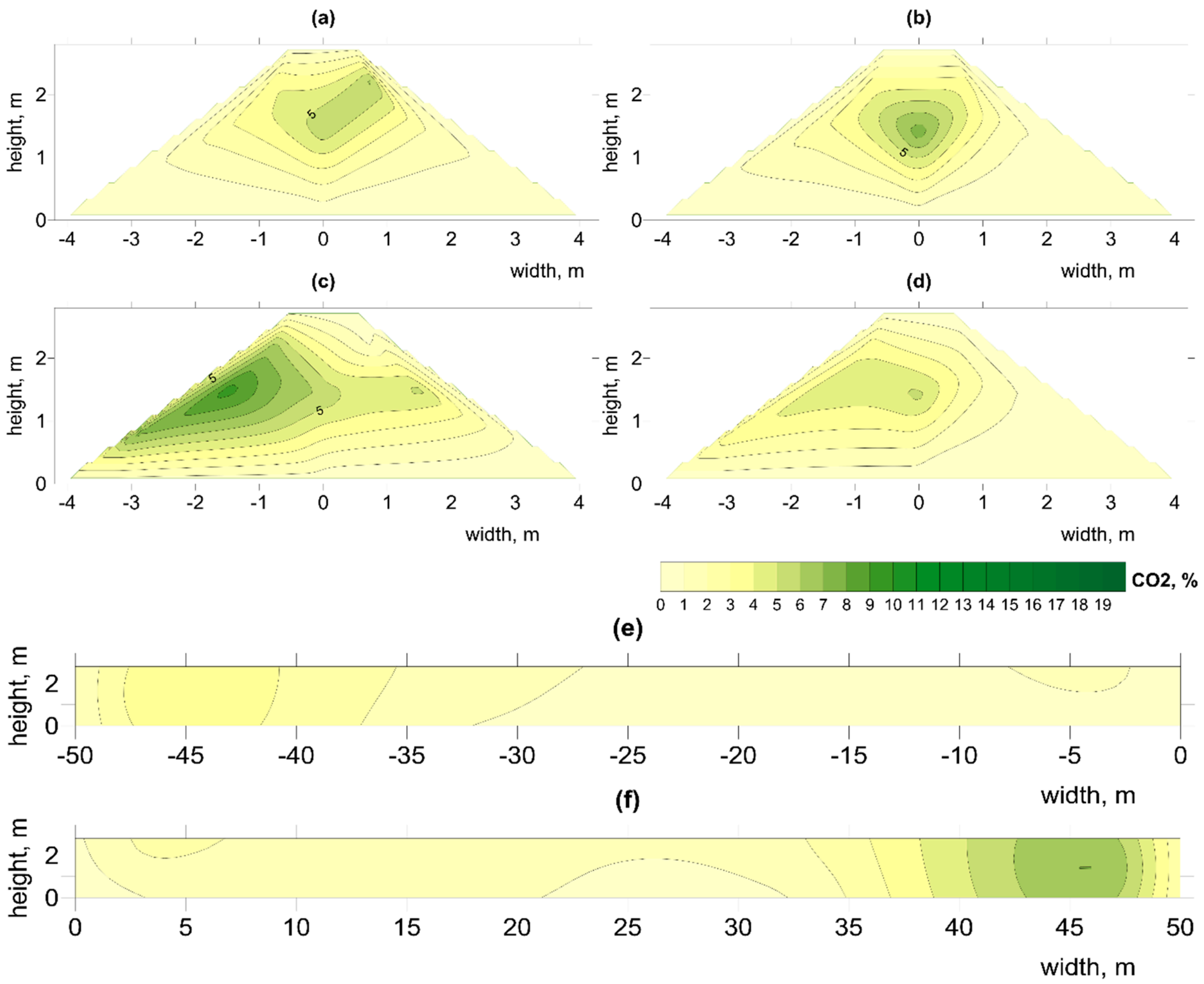

Figure S107. CO2 distribution in the pile B1, day 41.

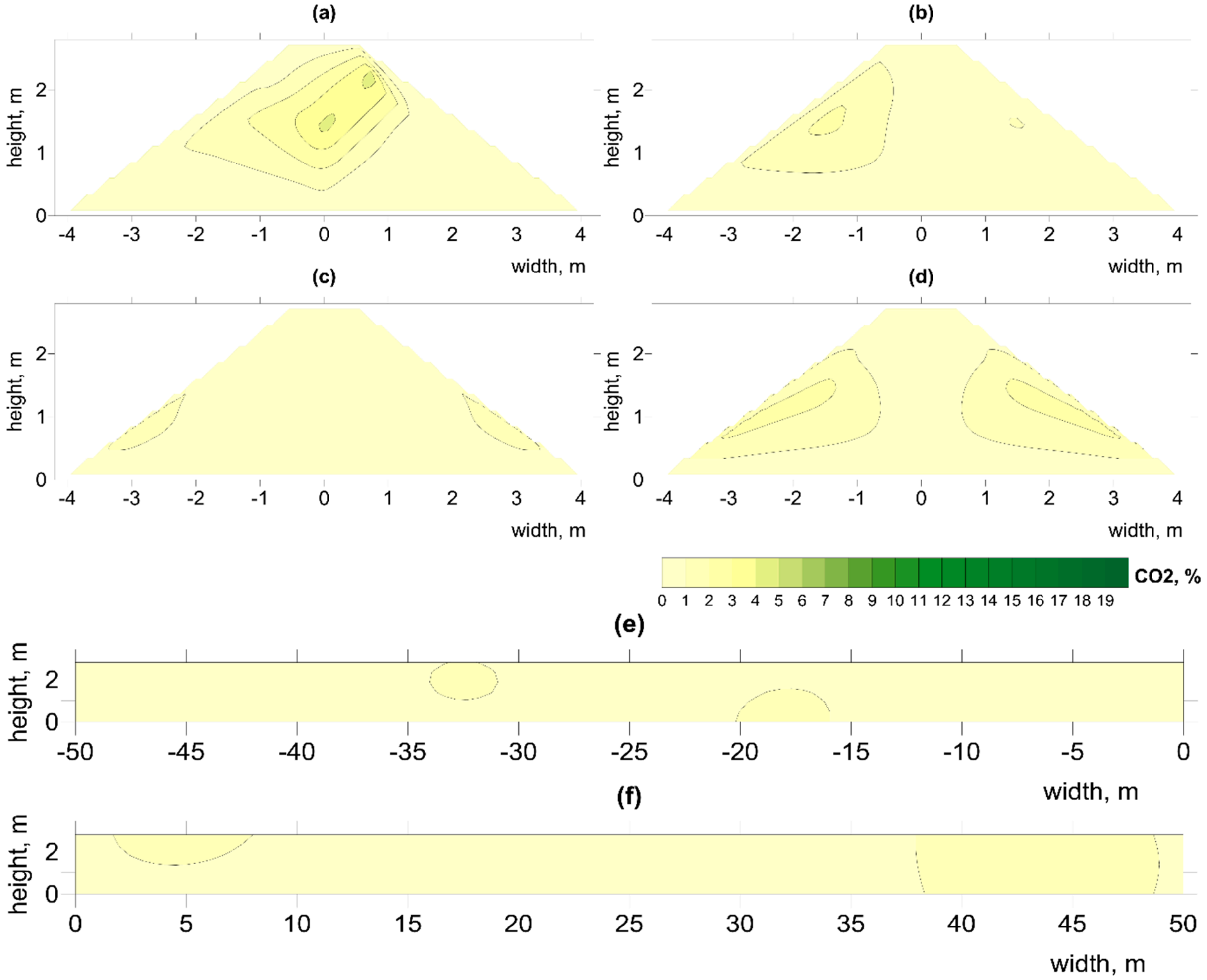

Figure S108. CO2 distribution in the pile B1, day 50.

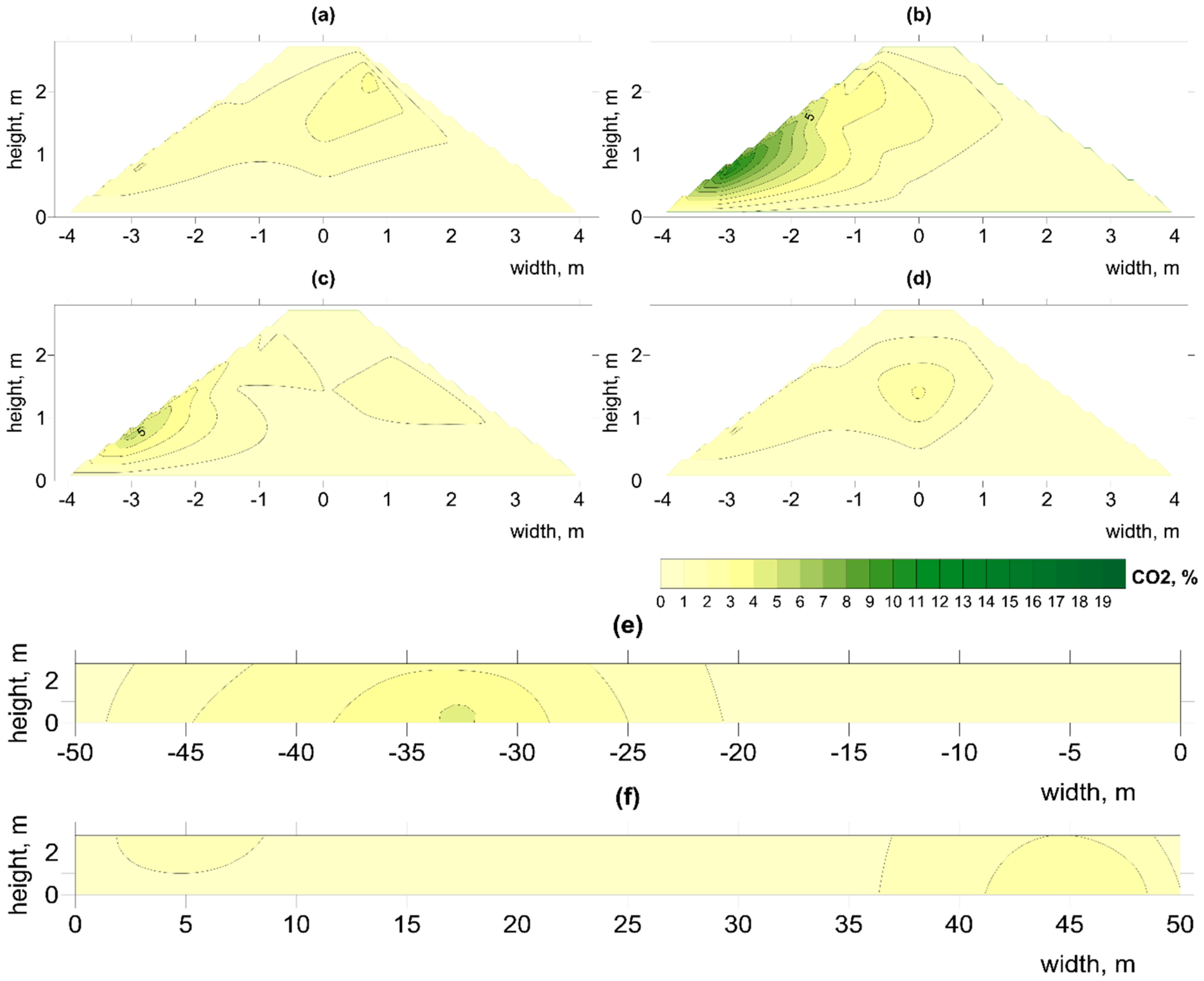

Figure S109. CO2 distribution in the pile B1, day 57.

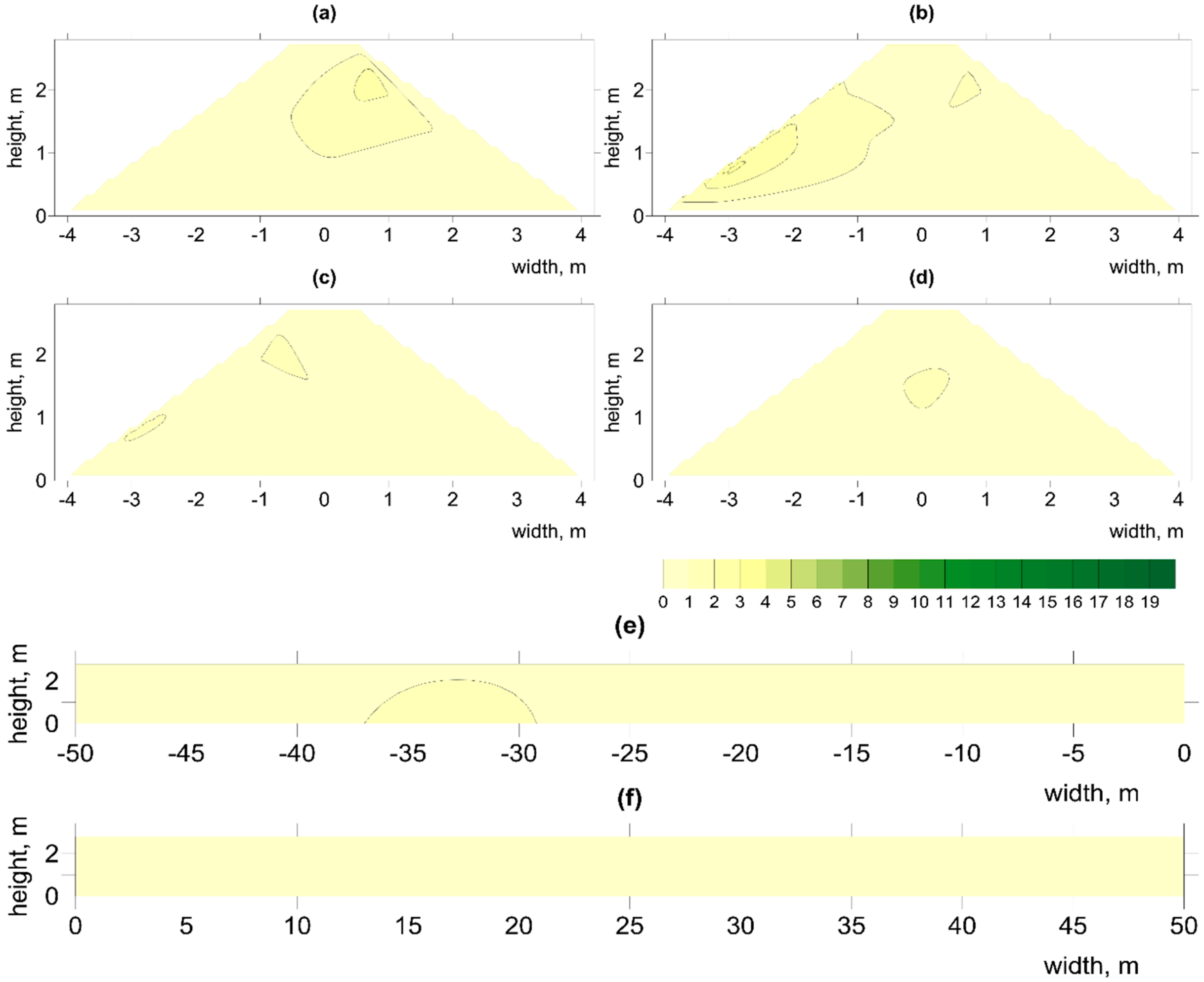

Figure S110. CO2 distribution in the pile B1, day 64.

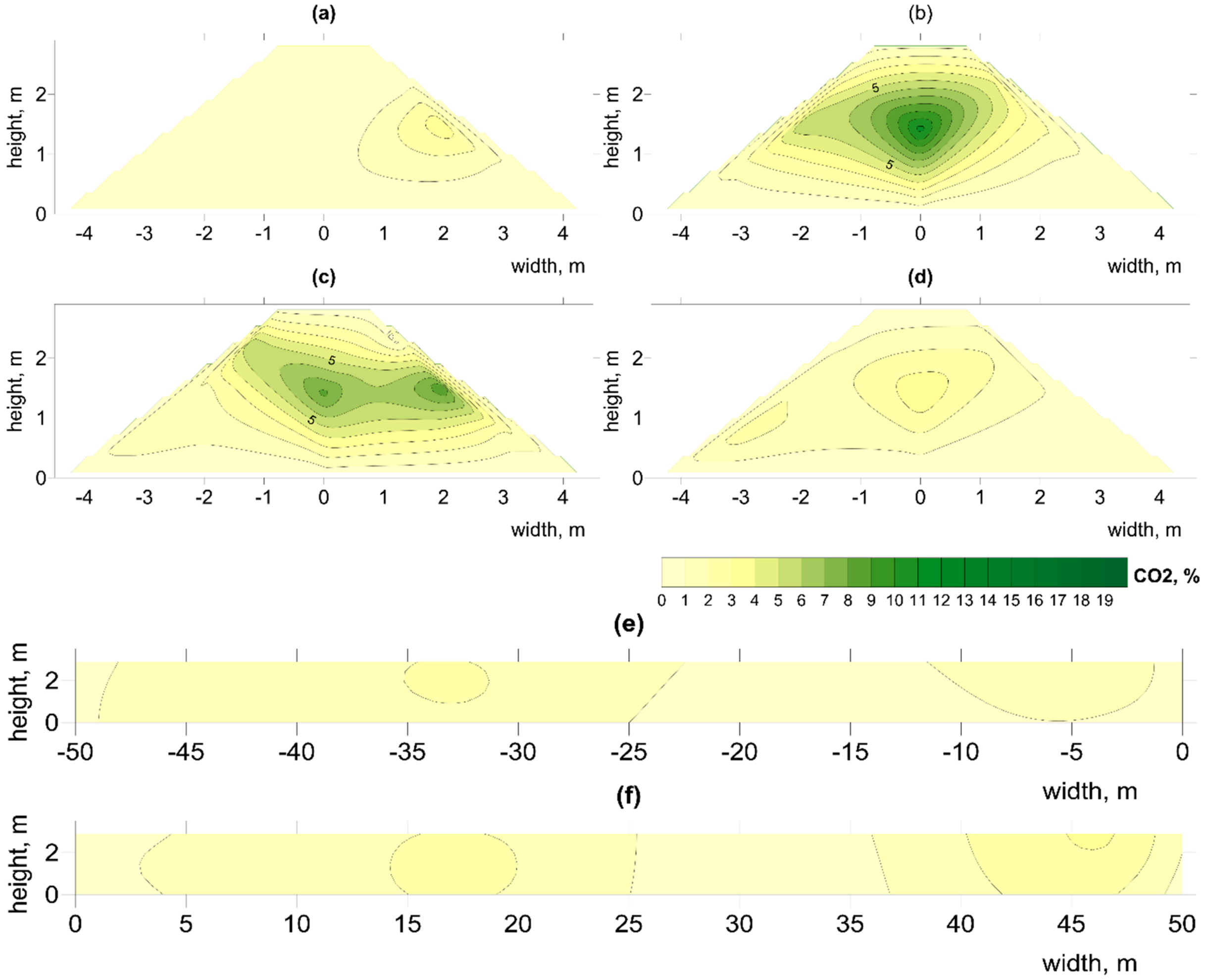

Figure S111. CO2 distribution in the pile B2, day 1.

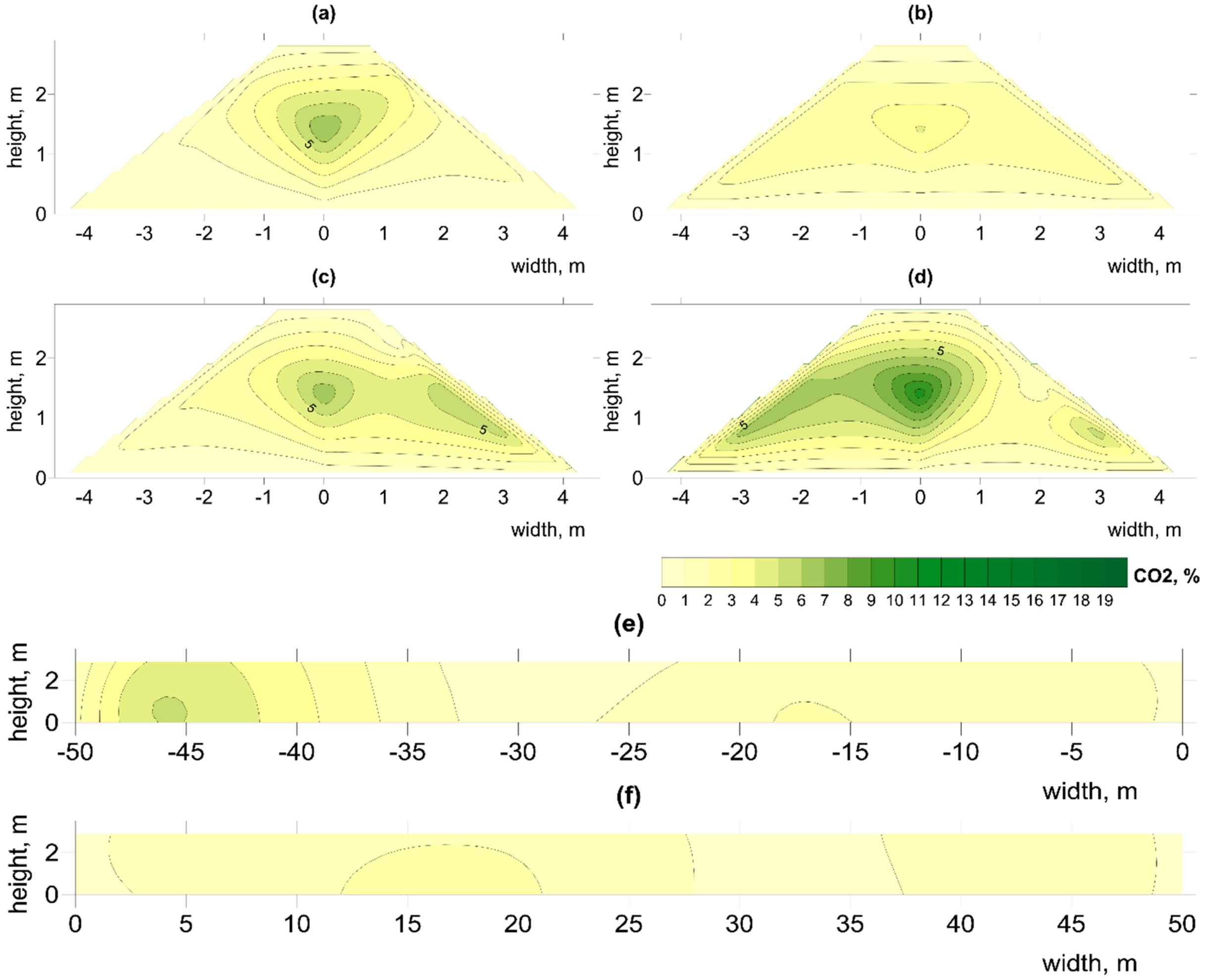

Figure S112. CO2 distribution in the pile B2, day 8.

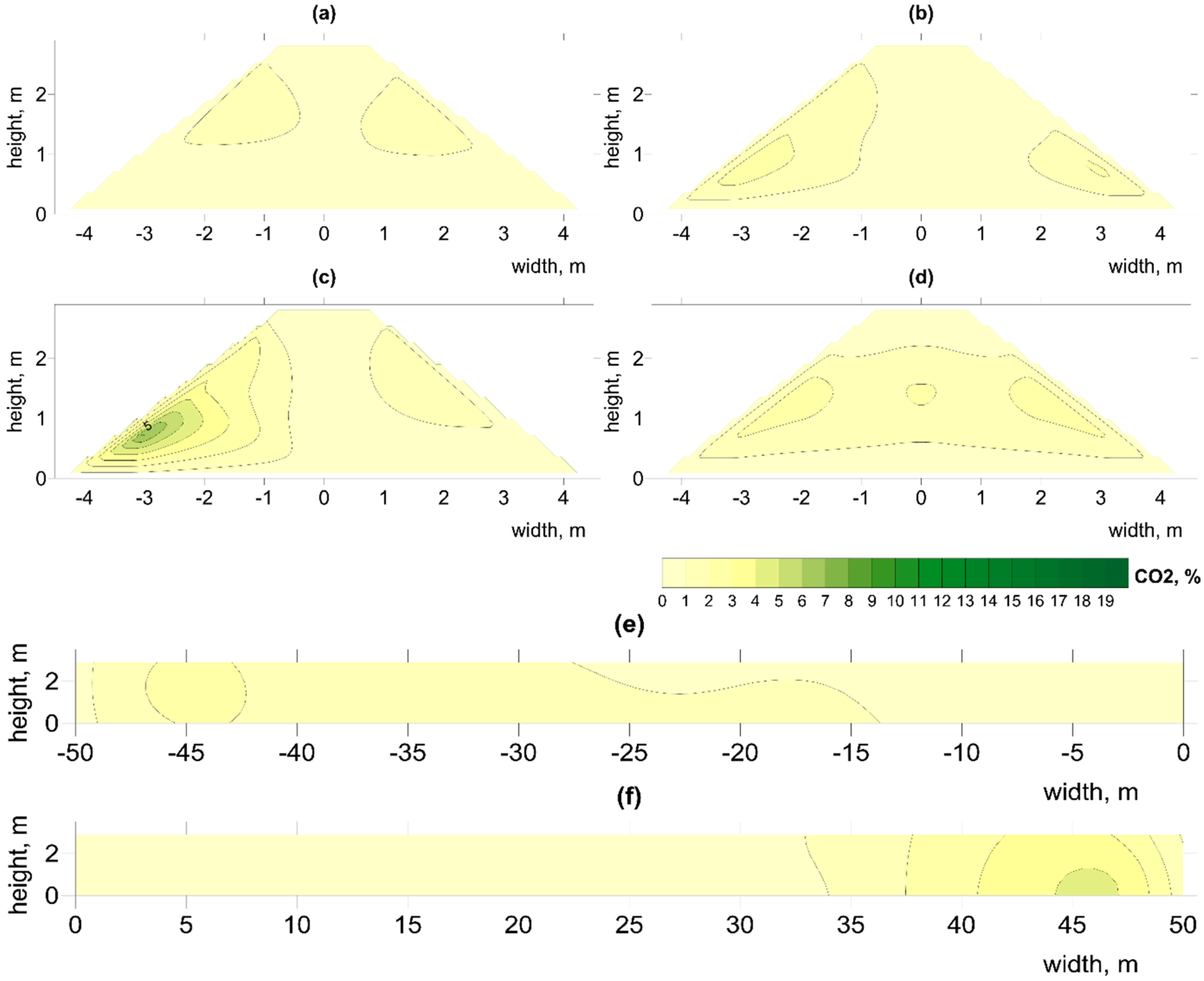

Figure S113. CO2 distribution in the pile B2, day 16.

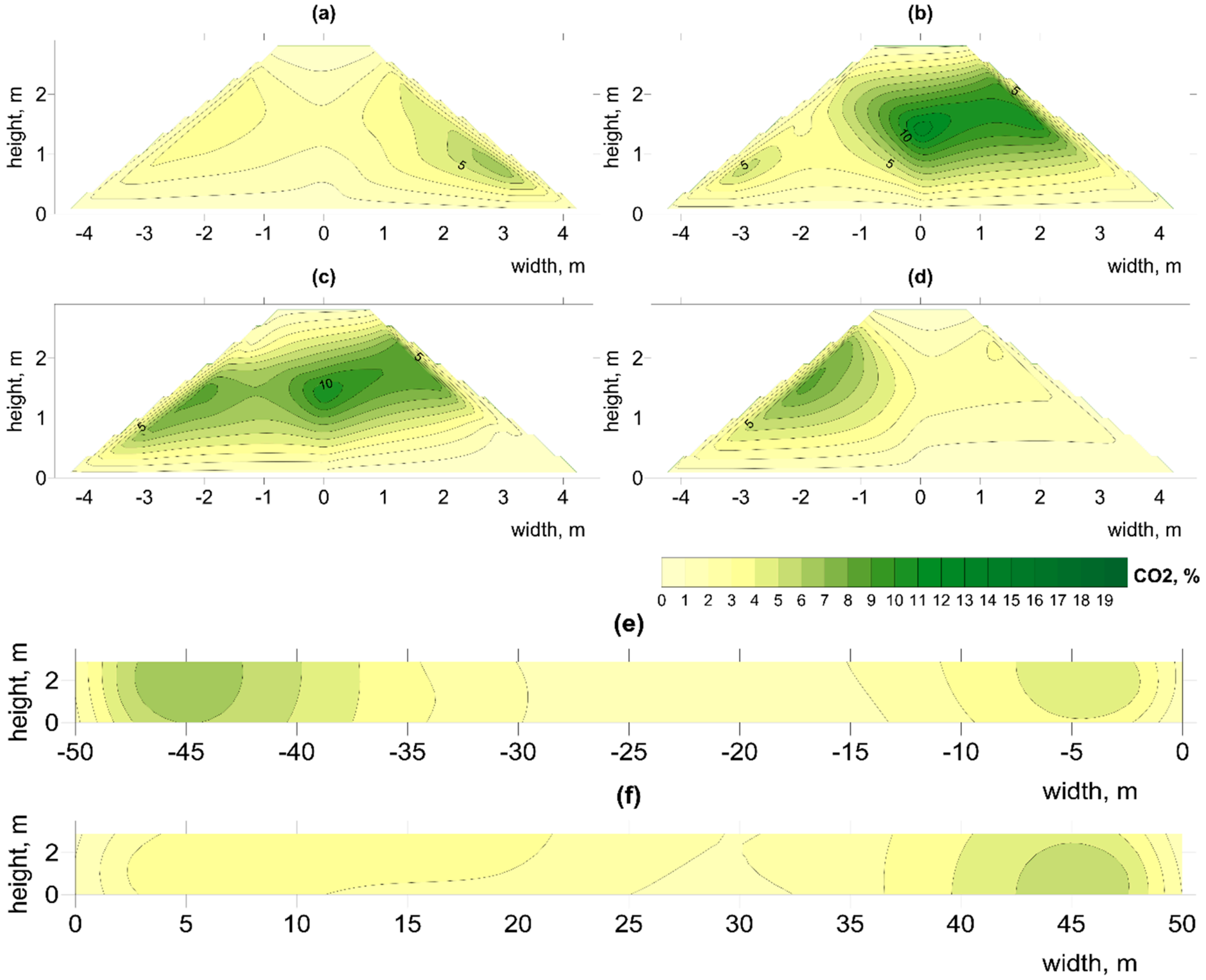

Figure S114. CO2 distribution in the pile B2, day 24.

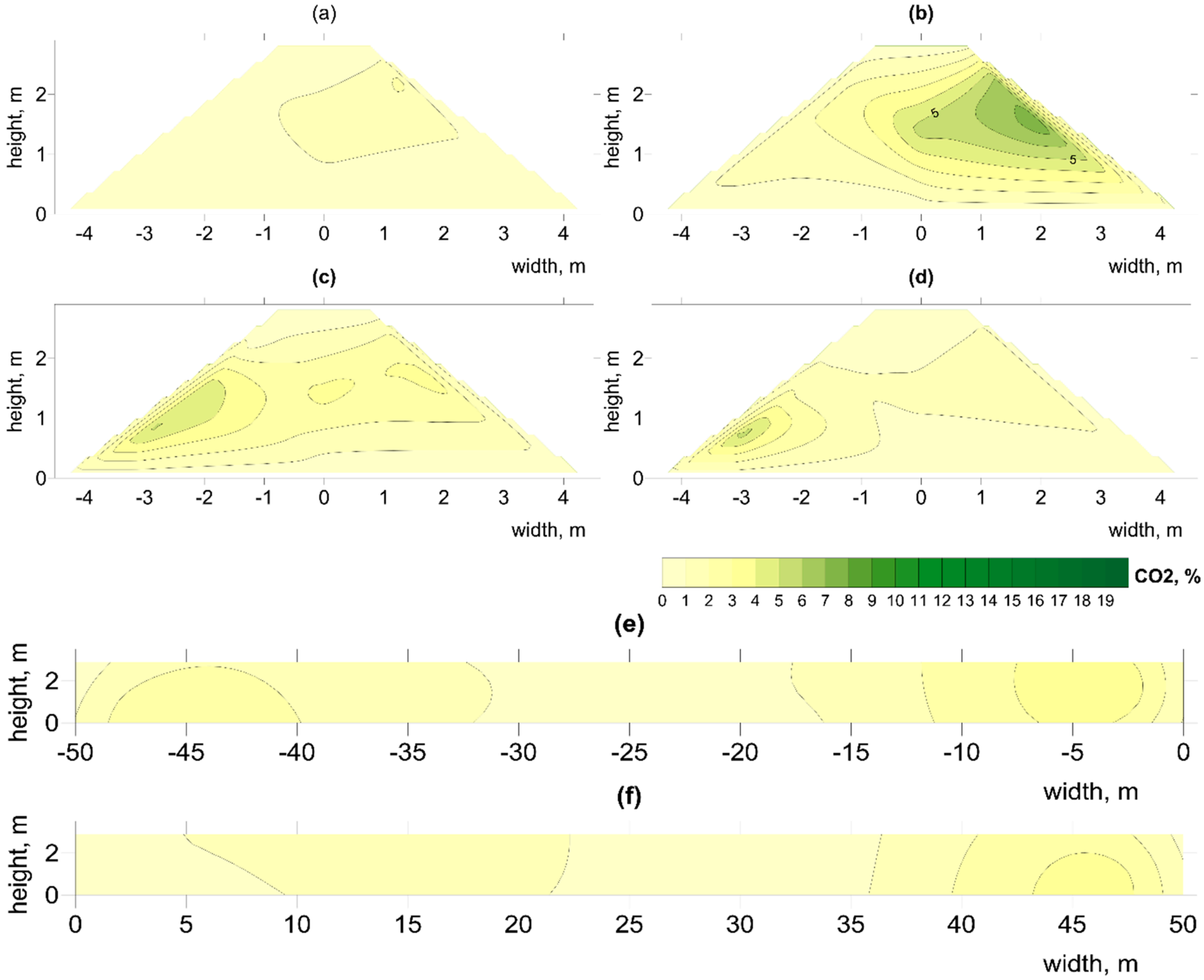

Figure S115. CO2 distribution in the pile B2, day 36.

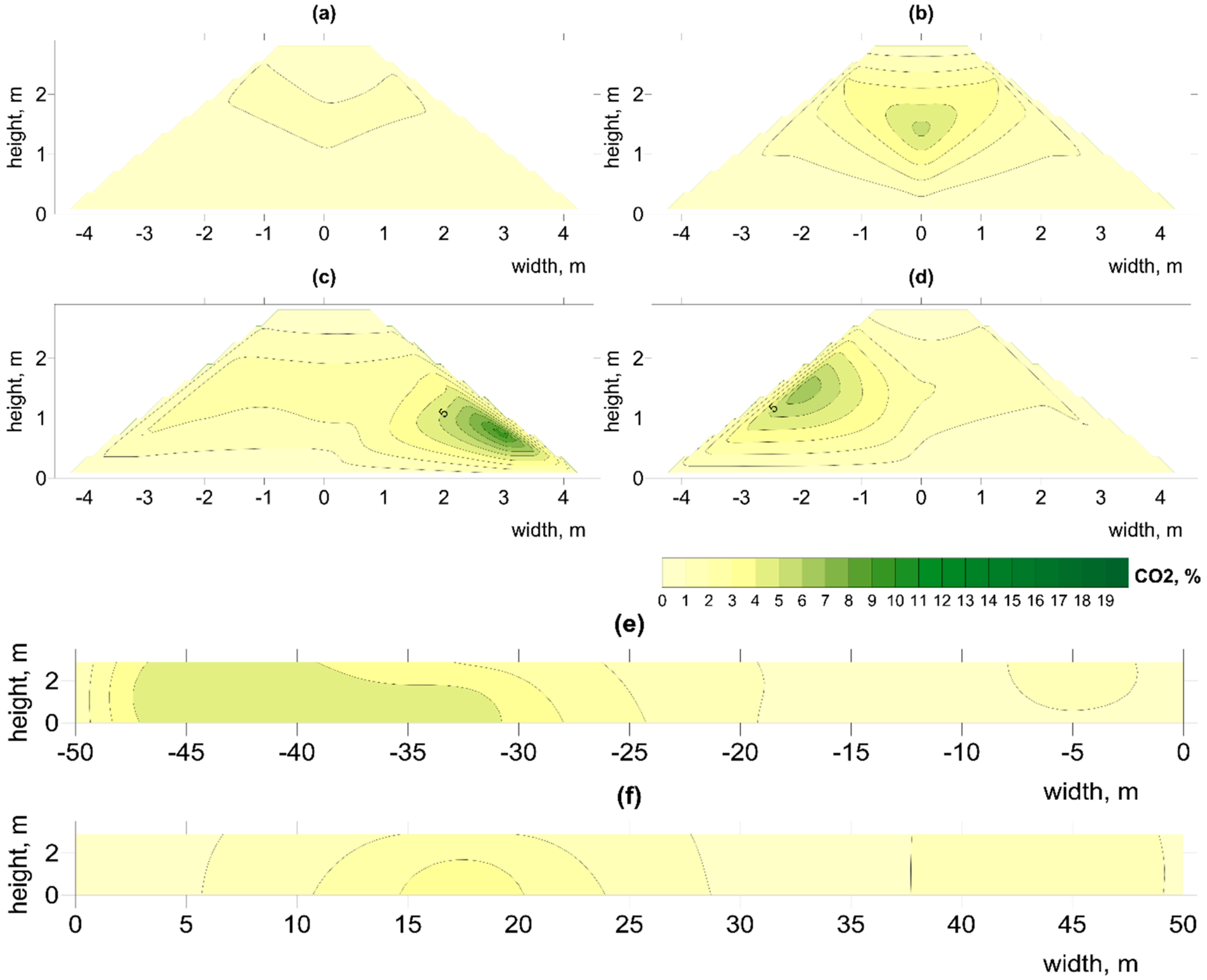

Figure S116. CO2 distribution in the pile B2, day 43.

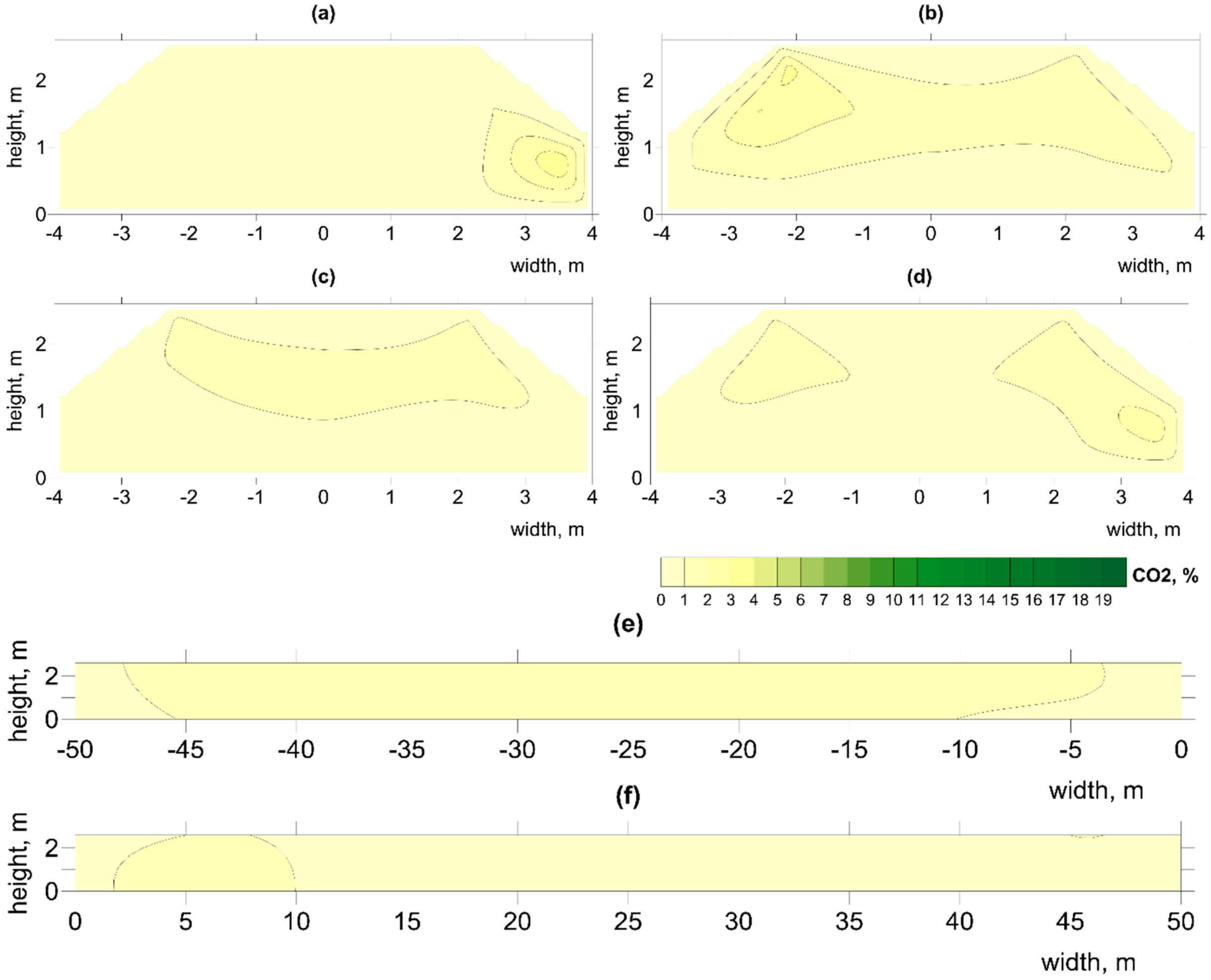

Figure S117. CO2 distribution in the pile C1, day 1.

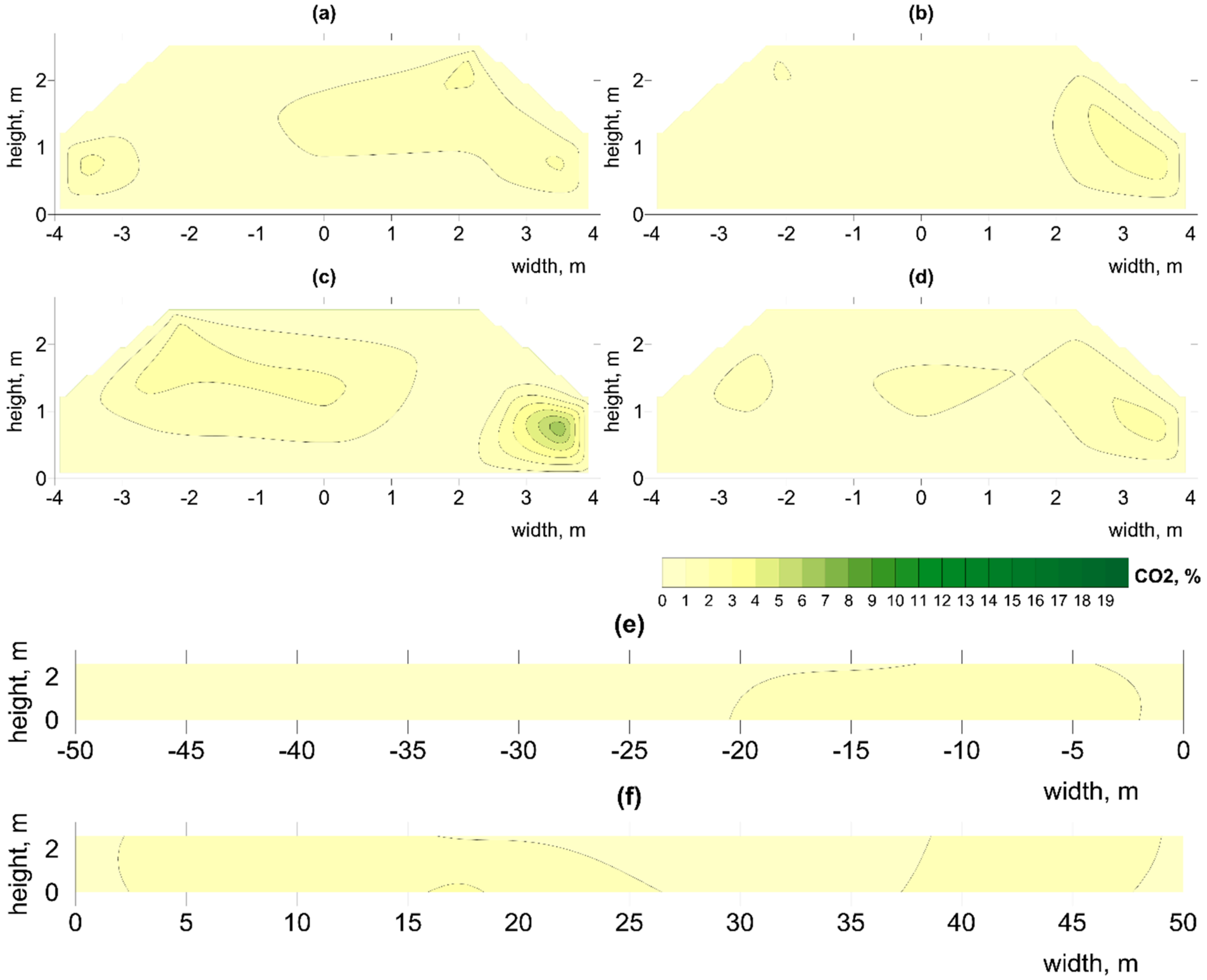

Figure S118. CO2 distribution in the pile C1, day 9.

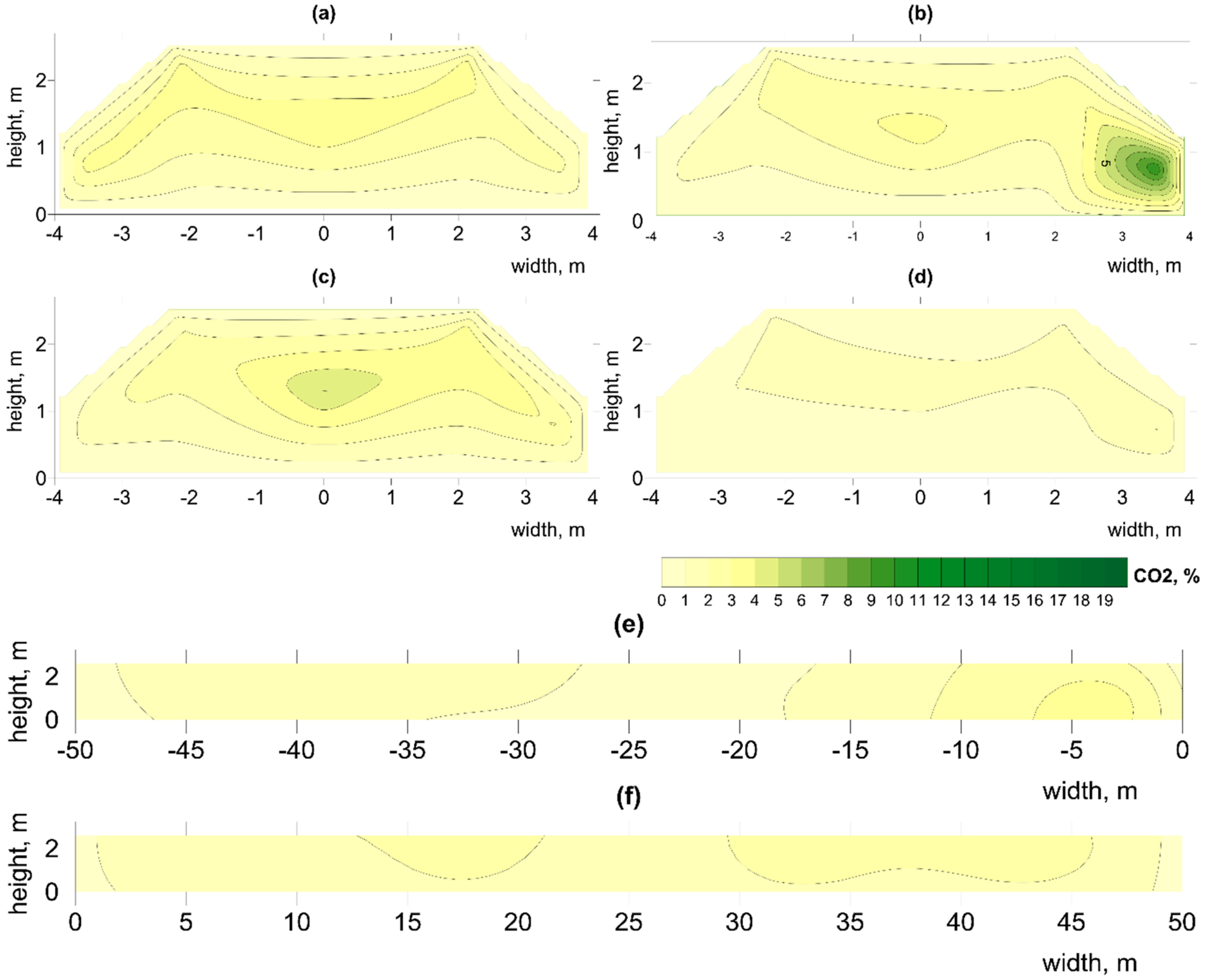

Figure S119. CO2 distribution in the pile C1, day 16.

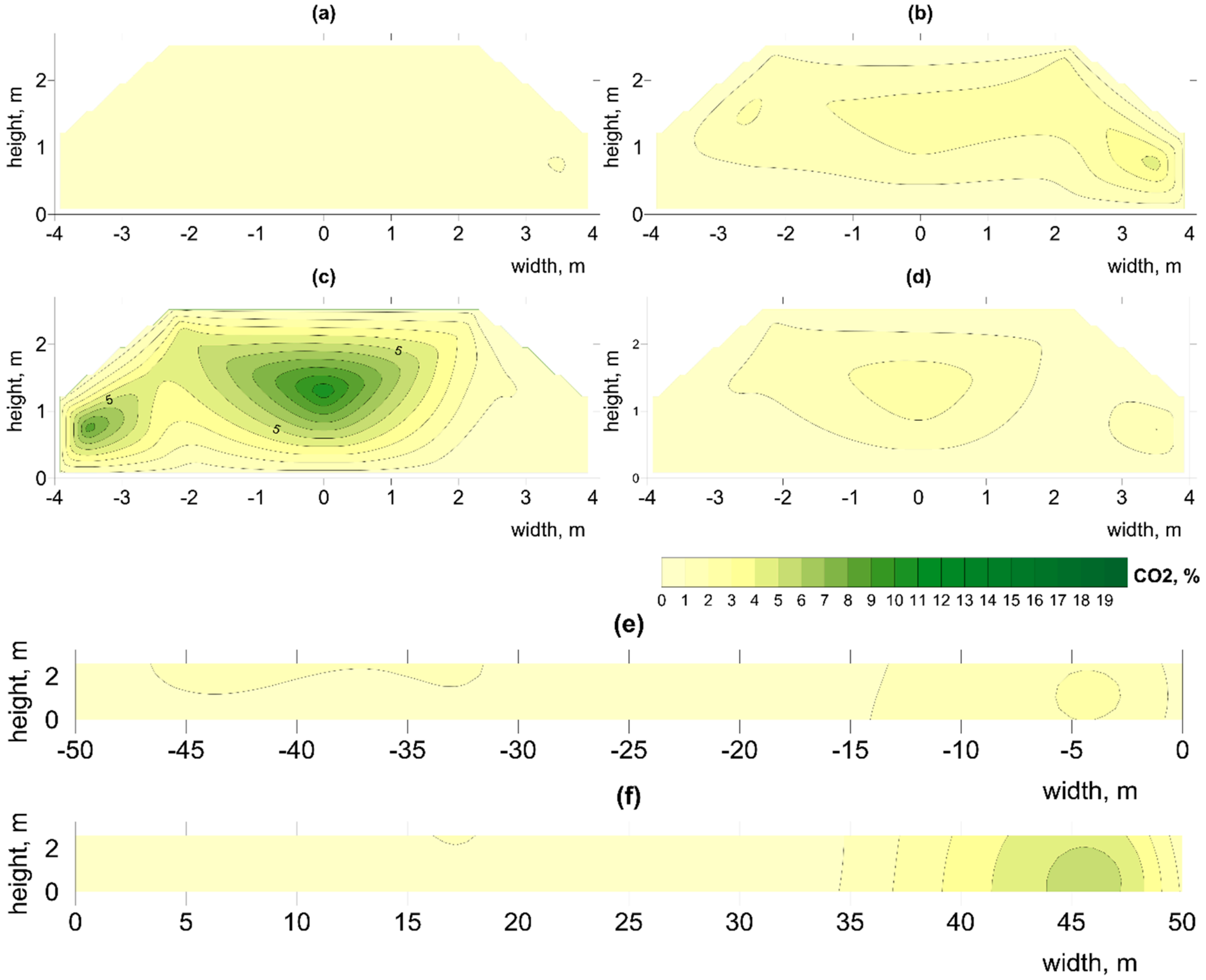

Figure S120. CO2 distribution in the pile C1, day 28.

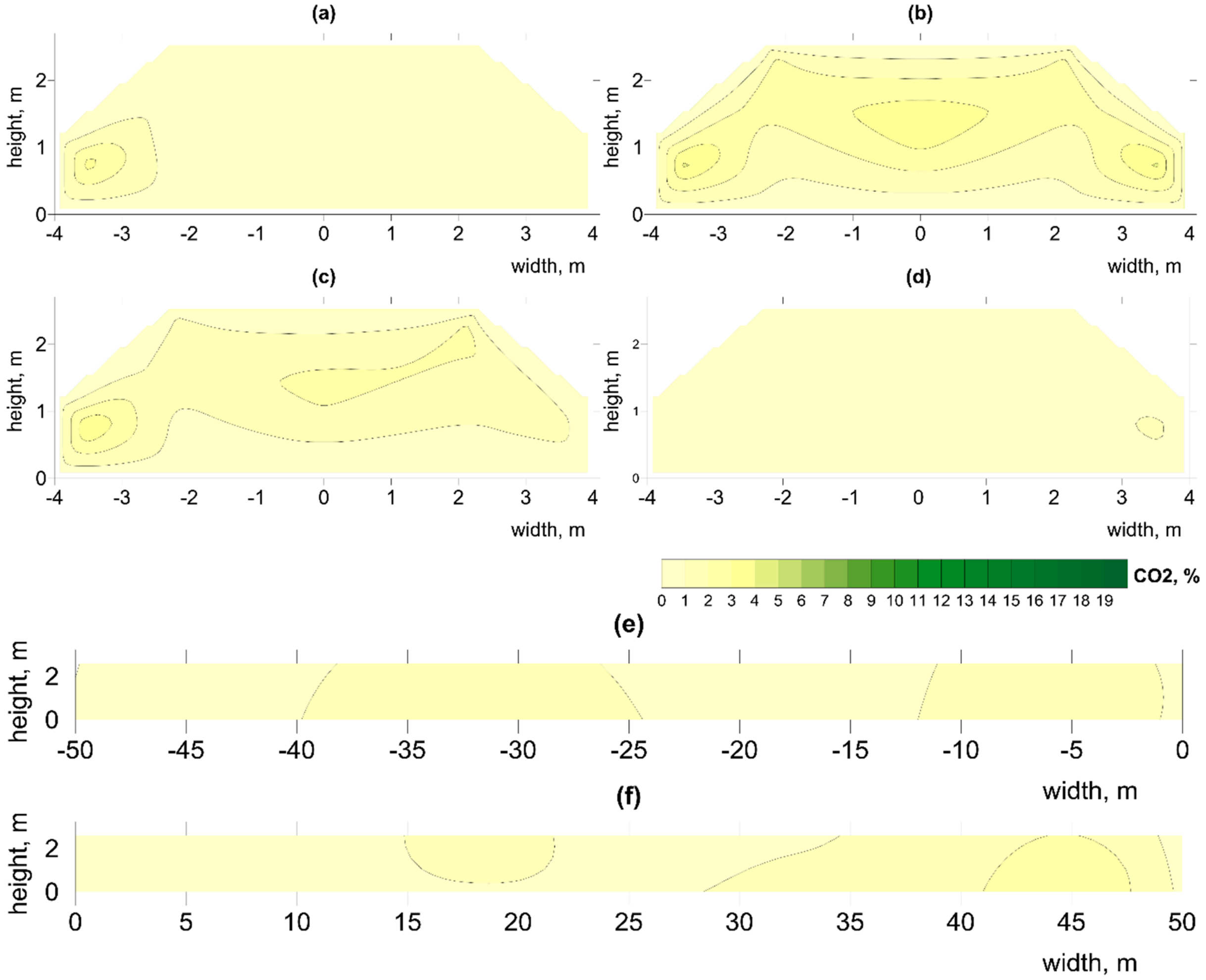

Figure S121. CO2 distribution in the pile C1, day 35.

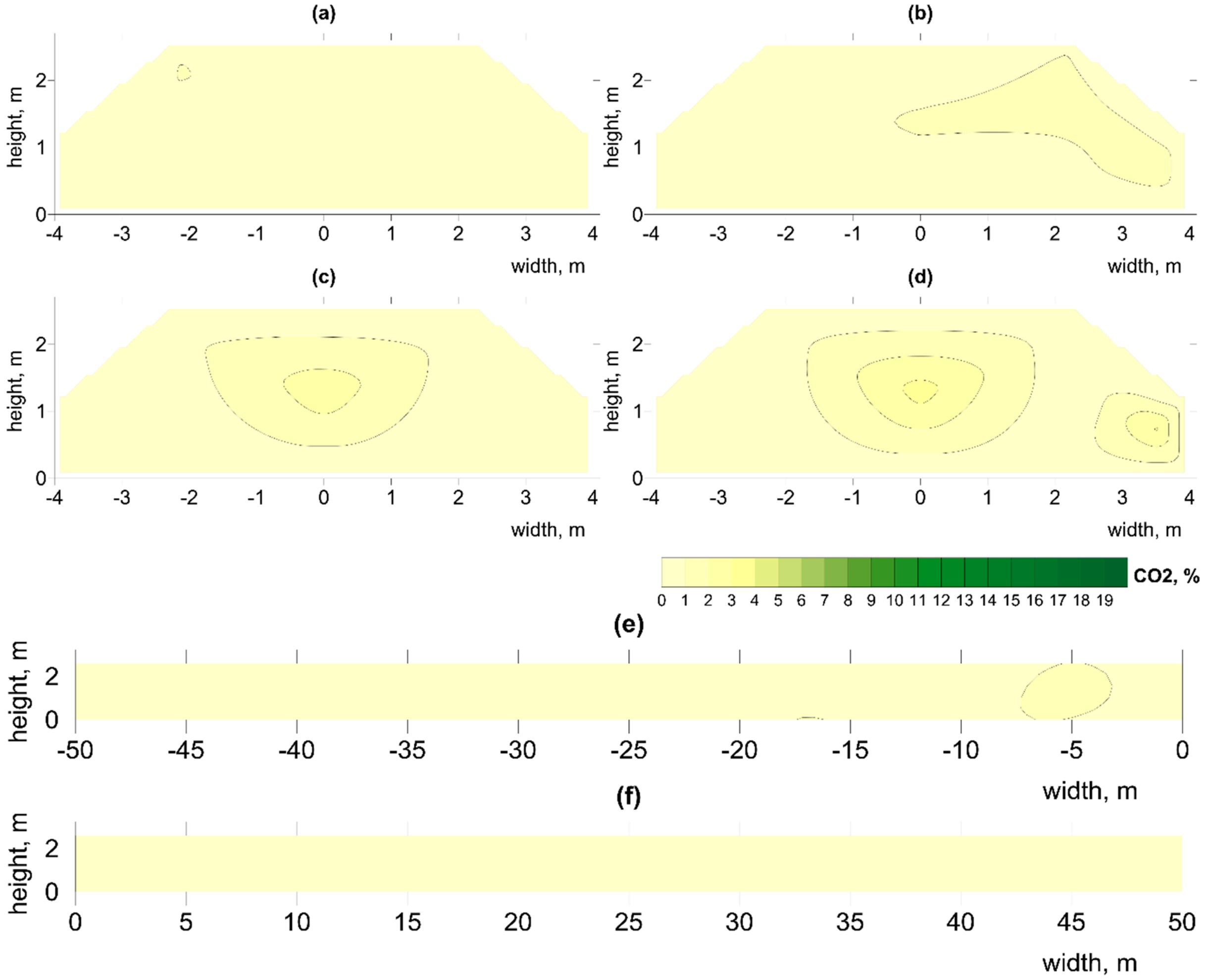

Figure S122. CO2 distribution in the pile C1, day 42.

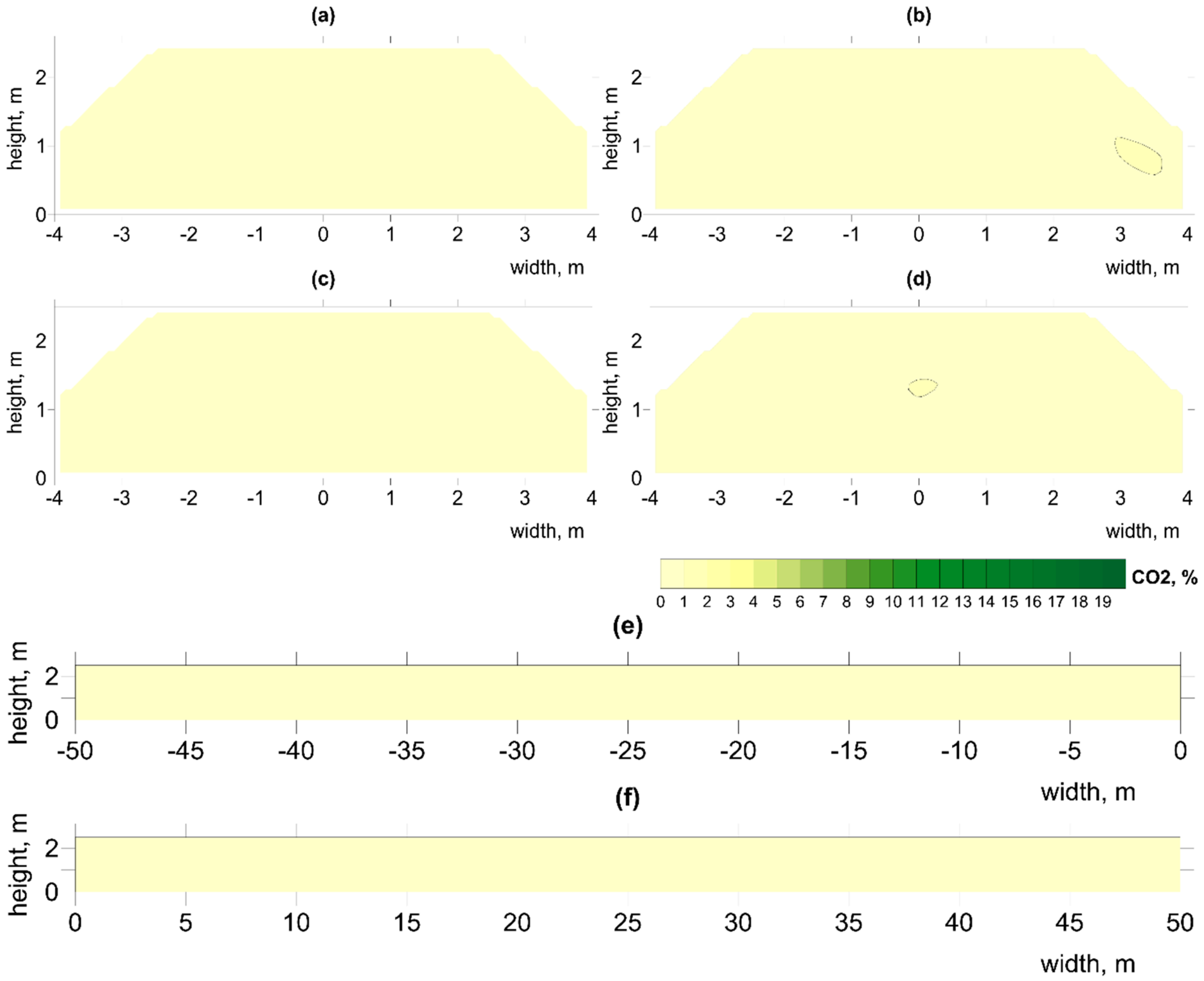

Figure S123. CO2 distribution in the pile C2, day 1.

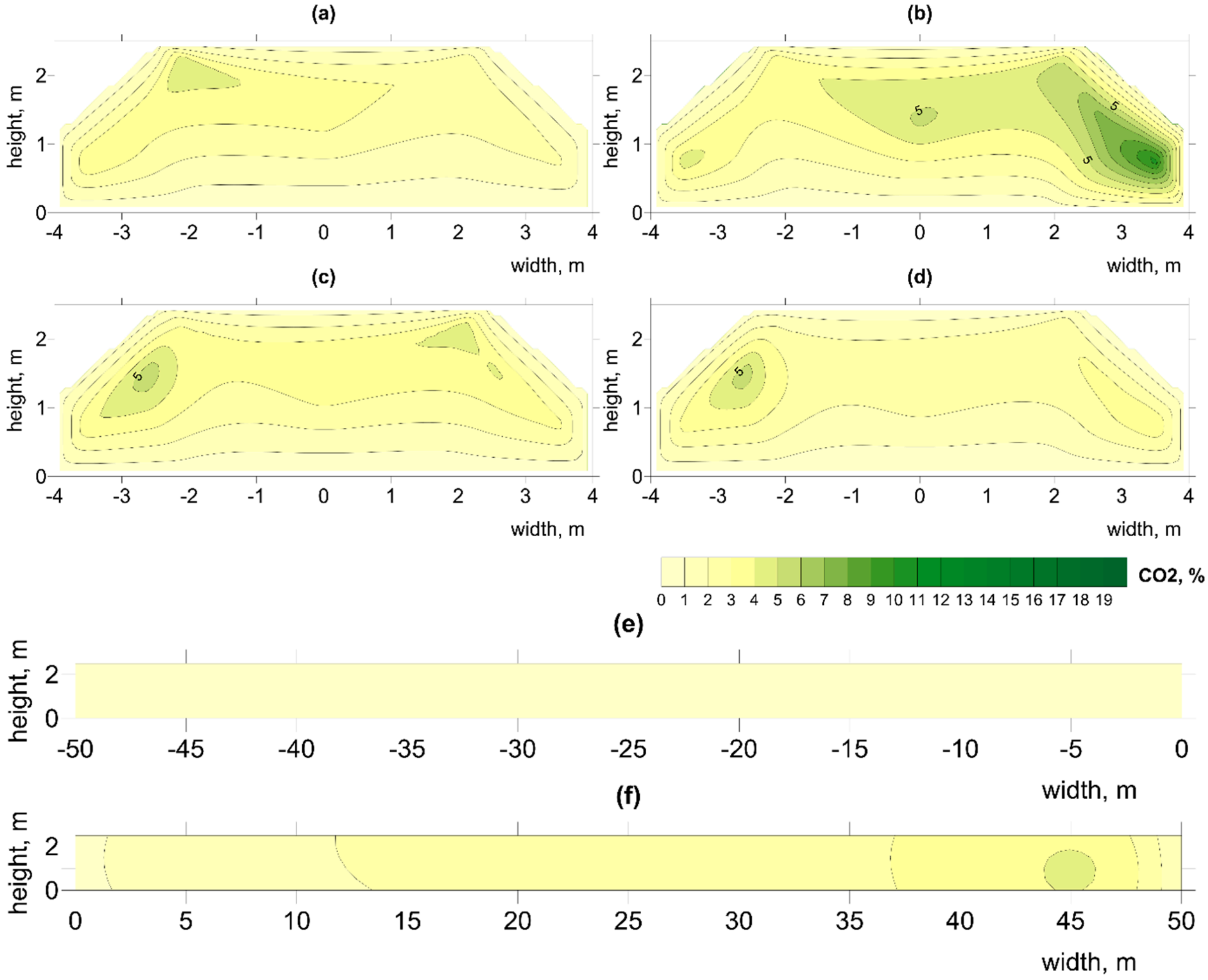

Figure S124. CO2 distribution in the pile C2, day 9.

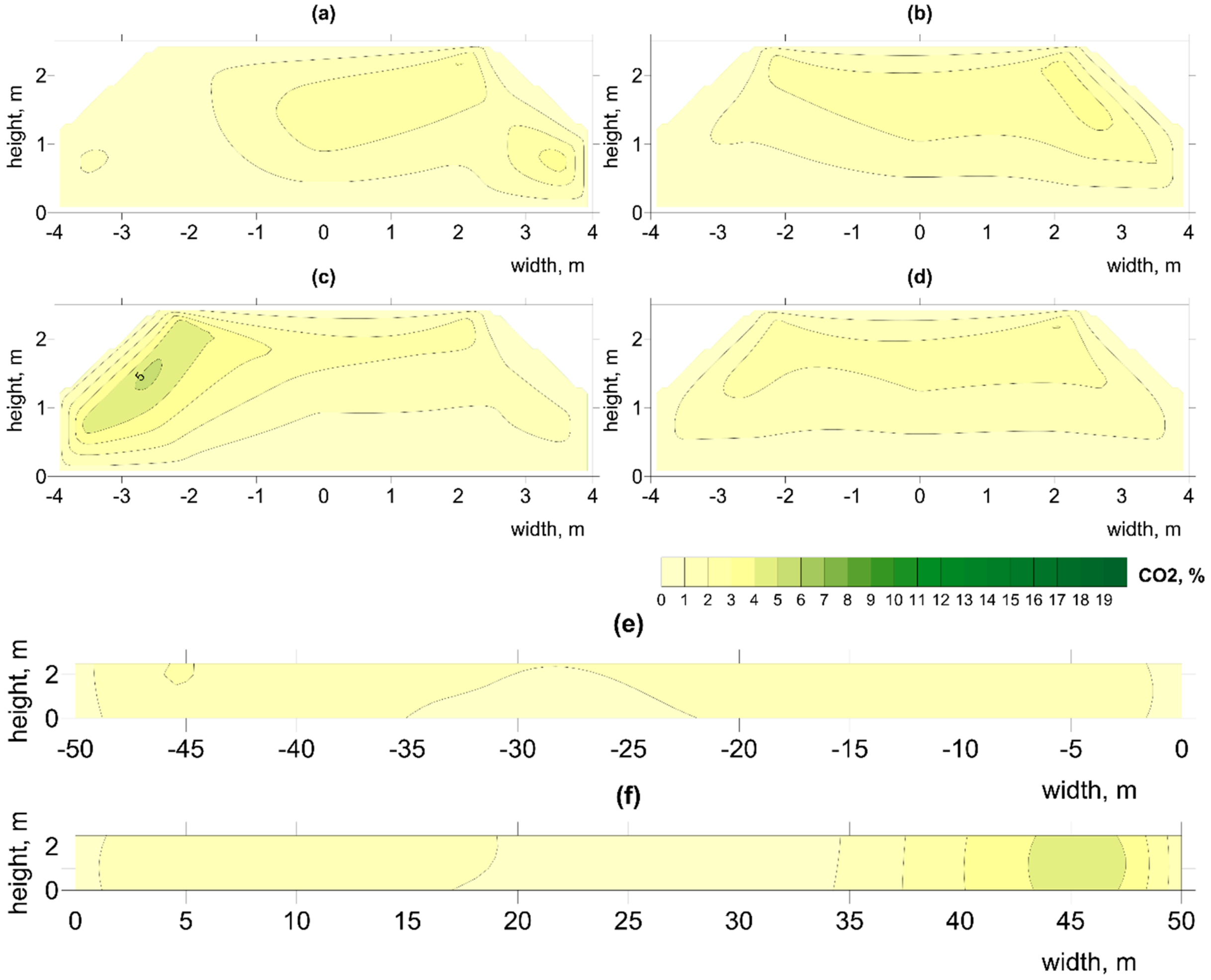

Figure S125. CO2 distribution in the pile C2, day 21.

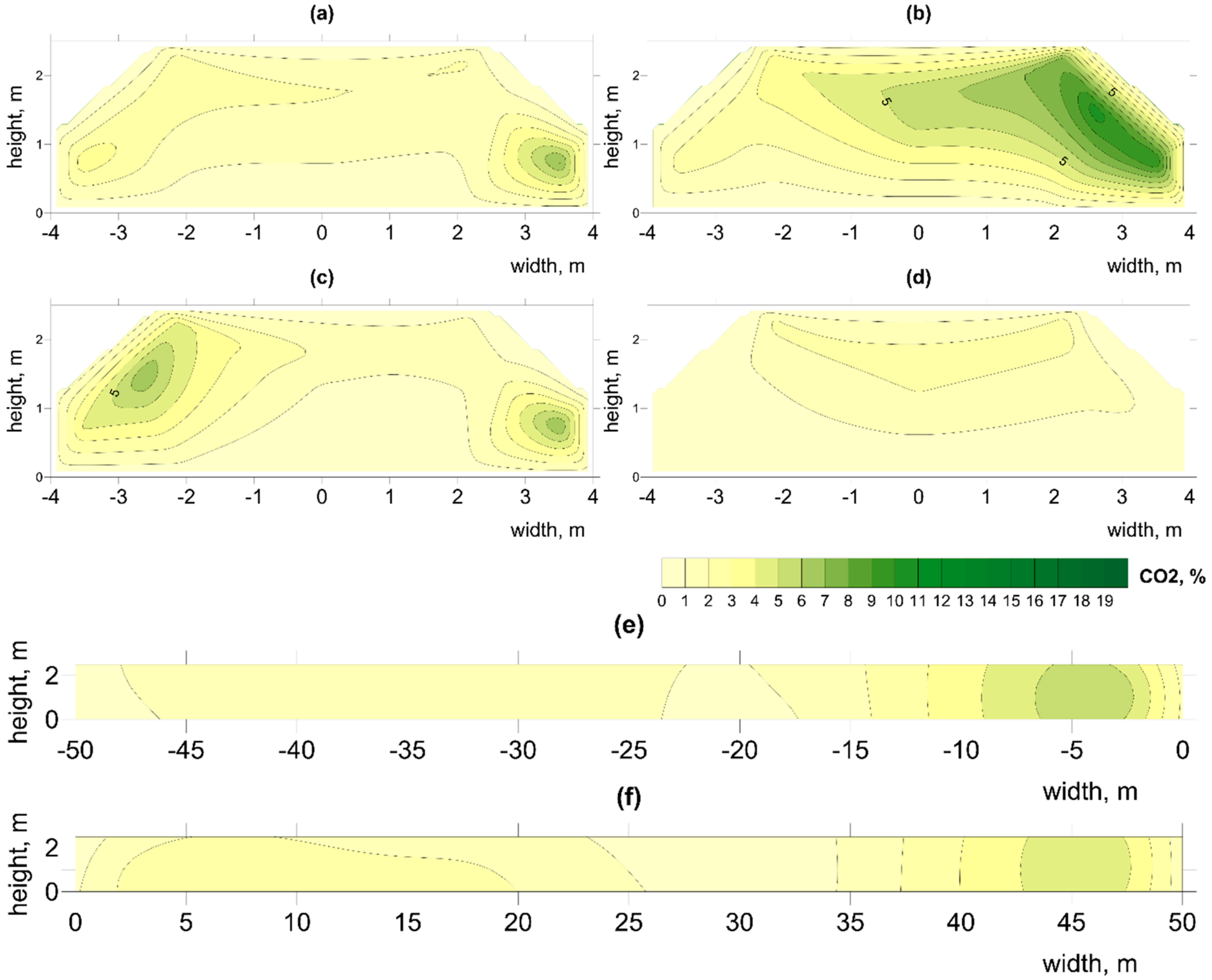

Figure S126. CO2 distribution in the pile C2, day 28.

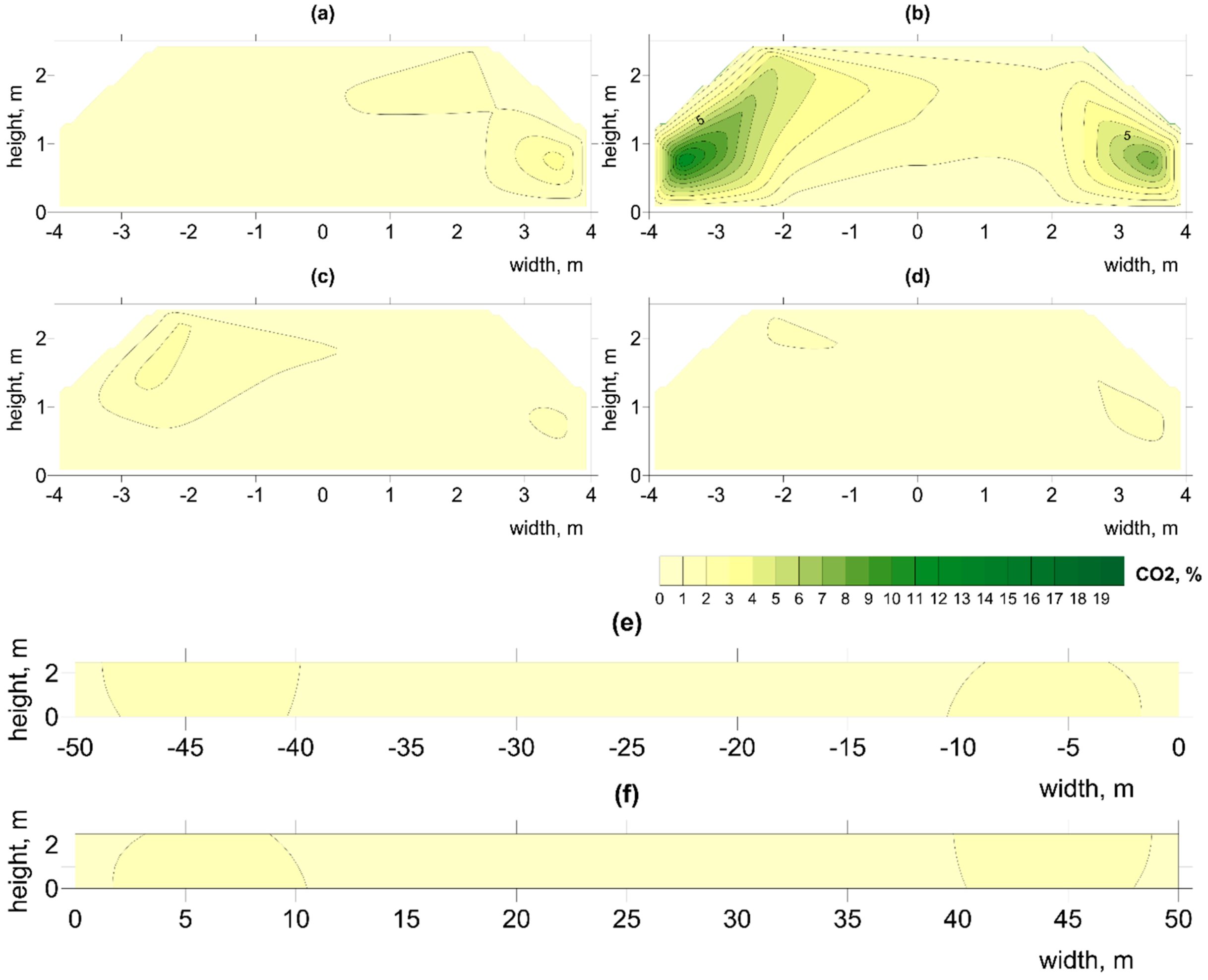

Figure S127. CO2 distribution in the pile C2, day 35.

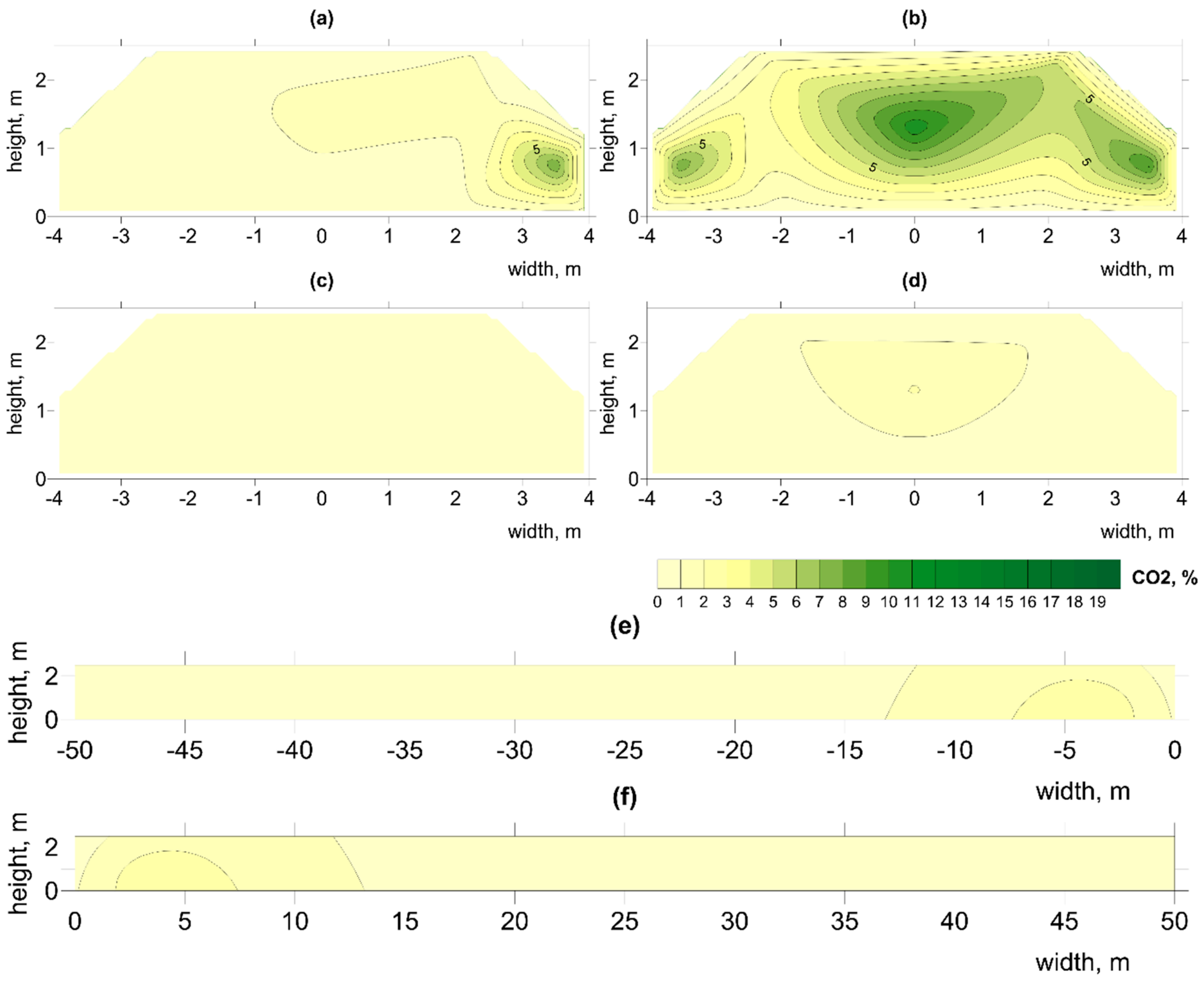

Figure S128. CO2 distribution in the pile C2, day 42.

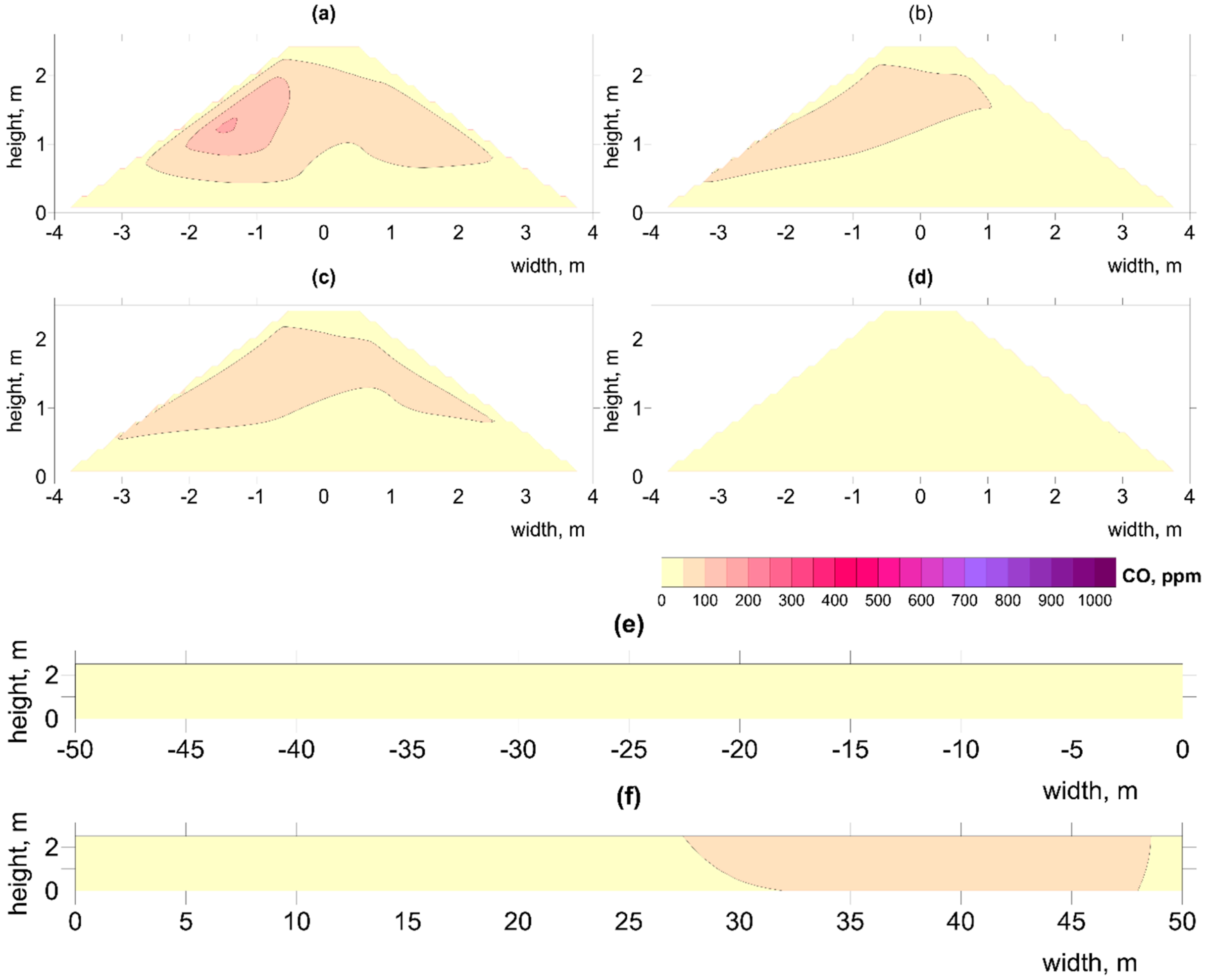

Figure S129. CO distribution in the pile A1, day 1.

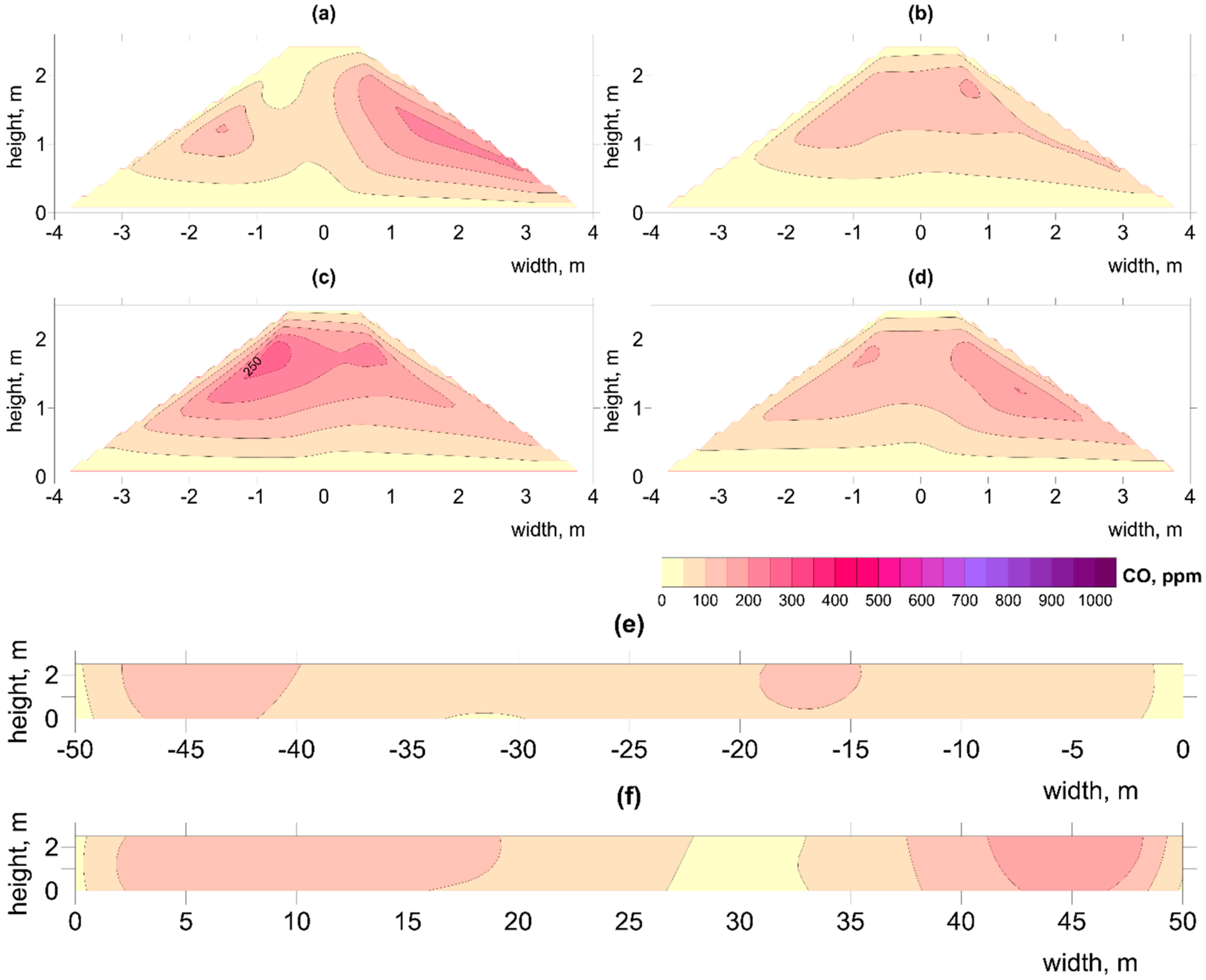

Figure S130. CO distribution in the pile A1, day 4.

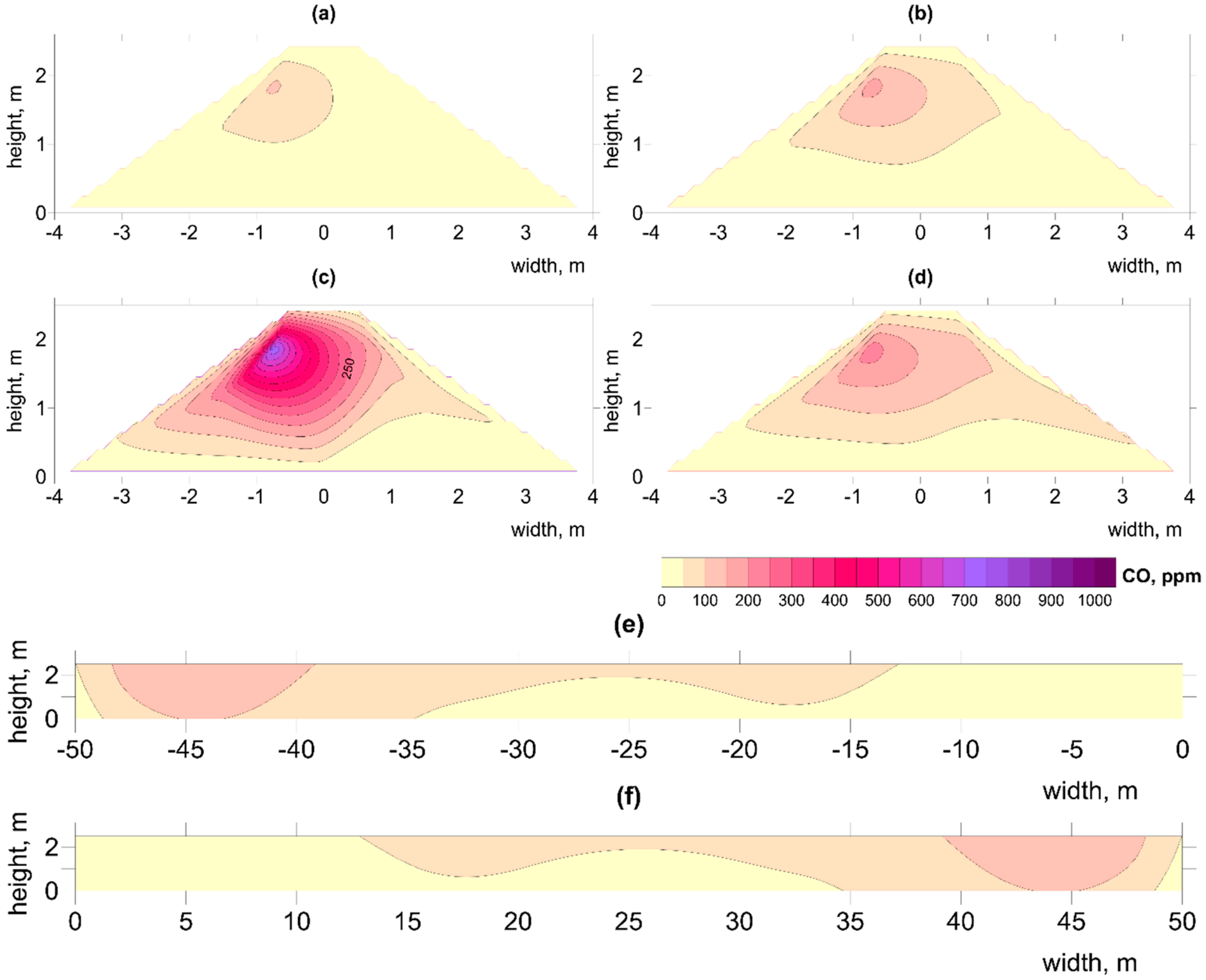

Figure S131. CO distribution in the pile A1, day 14.

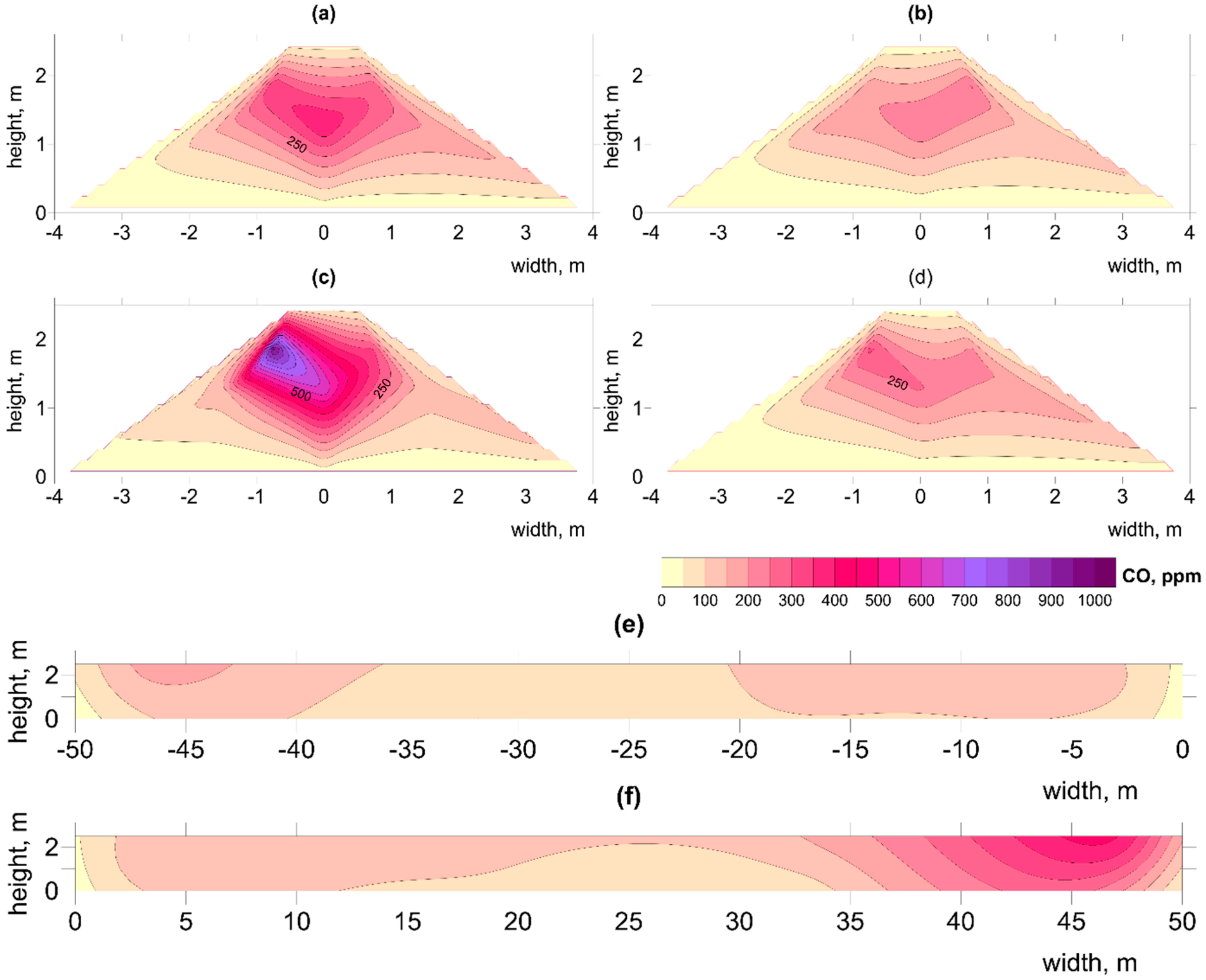

Figure S132. CO distribution in the pile A1, day 20.

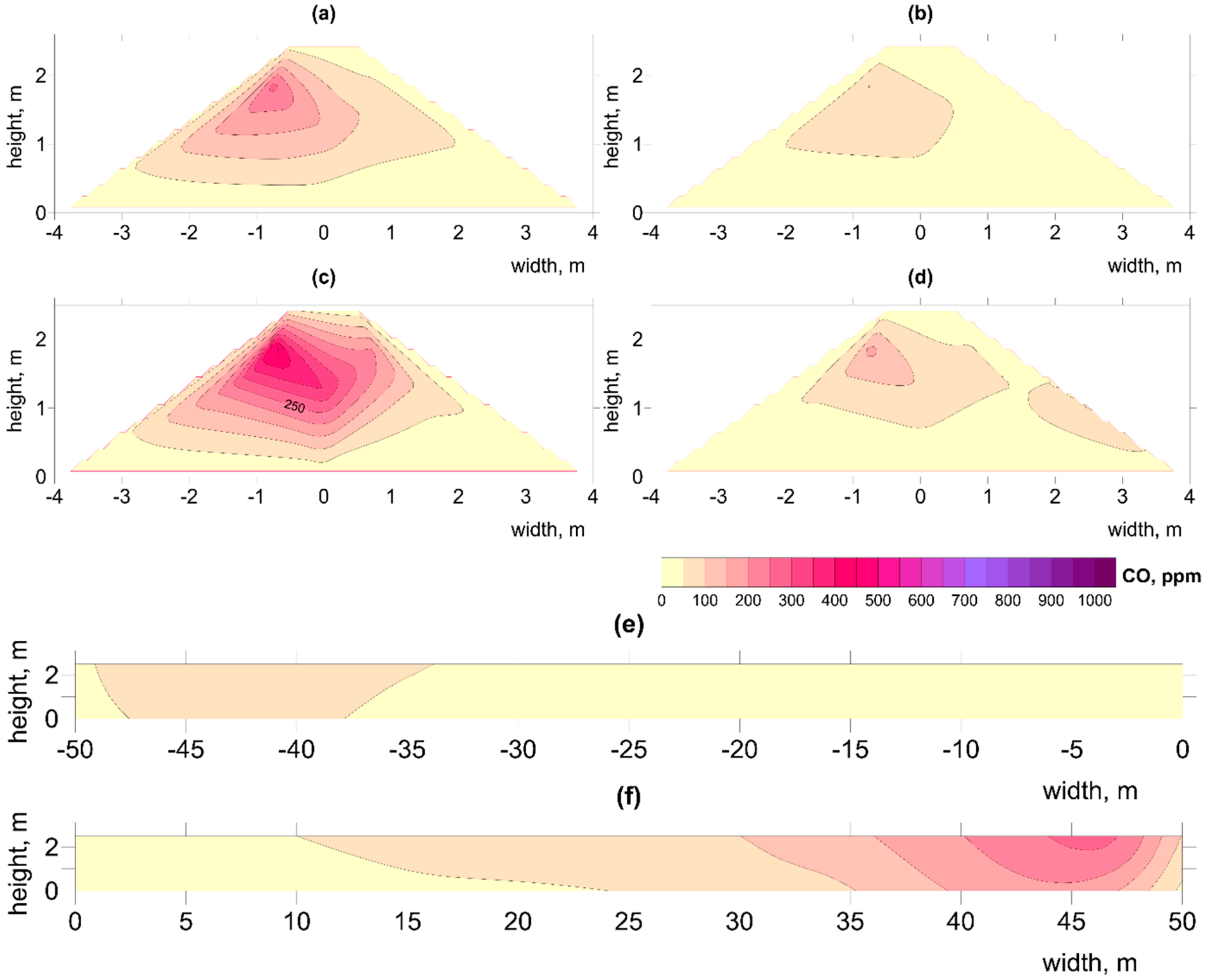

Figure S133. CO distribution in the pile A1, day 28.

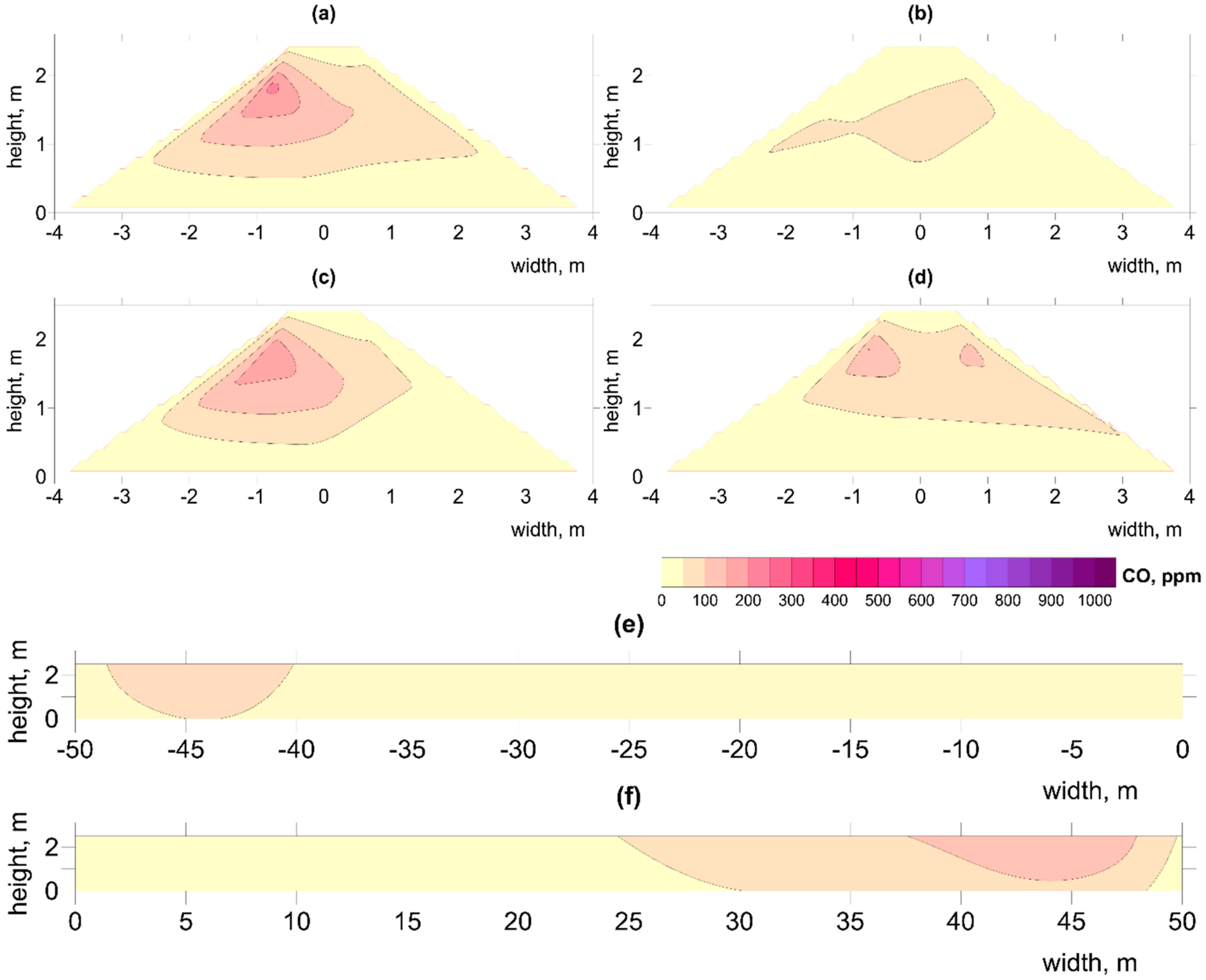

Figure S134. CO distribution in the pile A1, day 35.

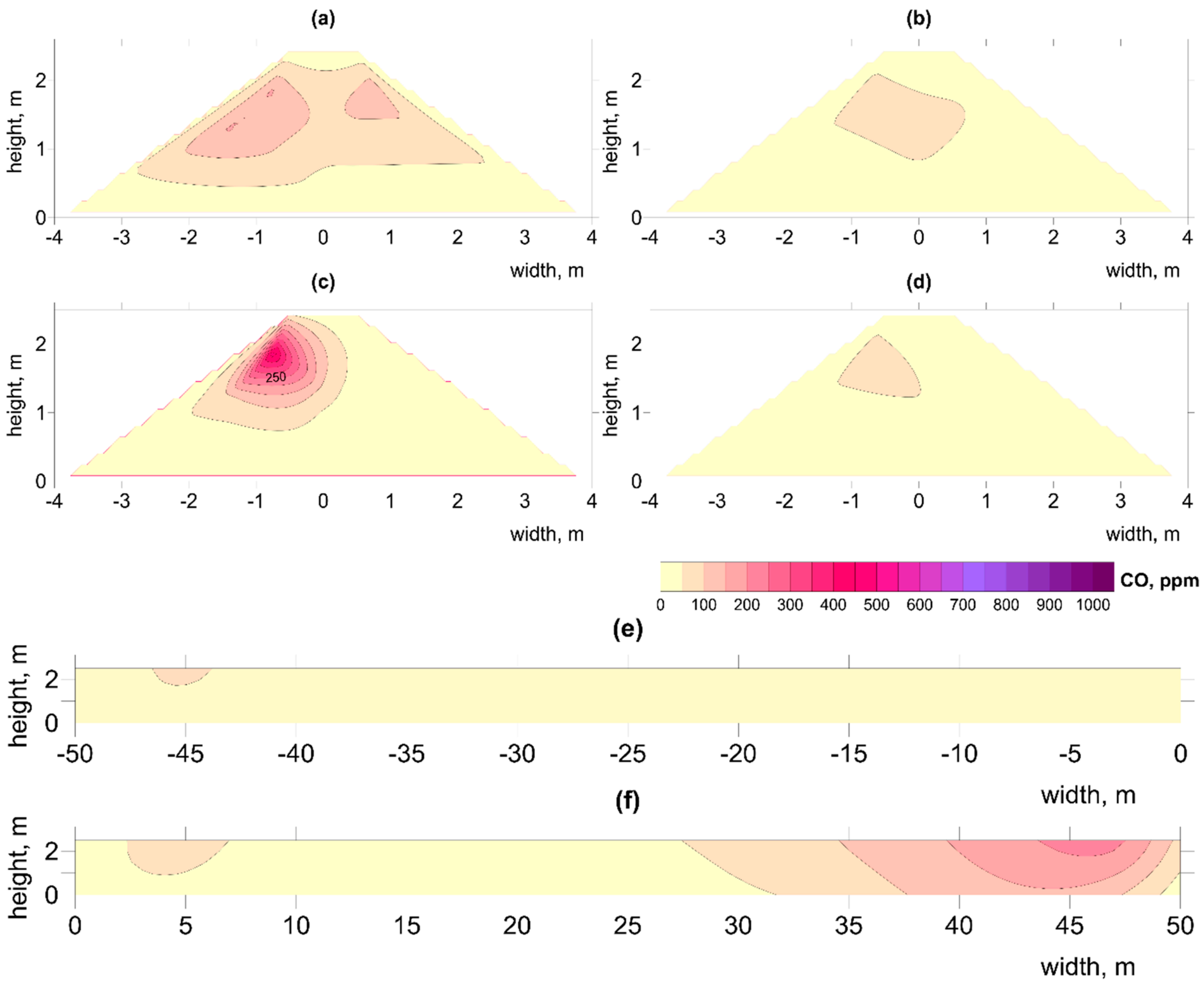

Figure S135. CO distribution in the pile A1, day 41.

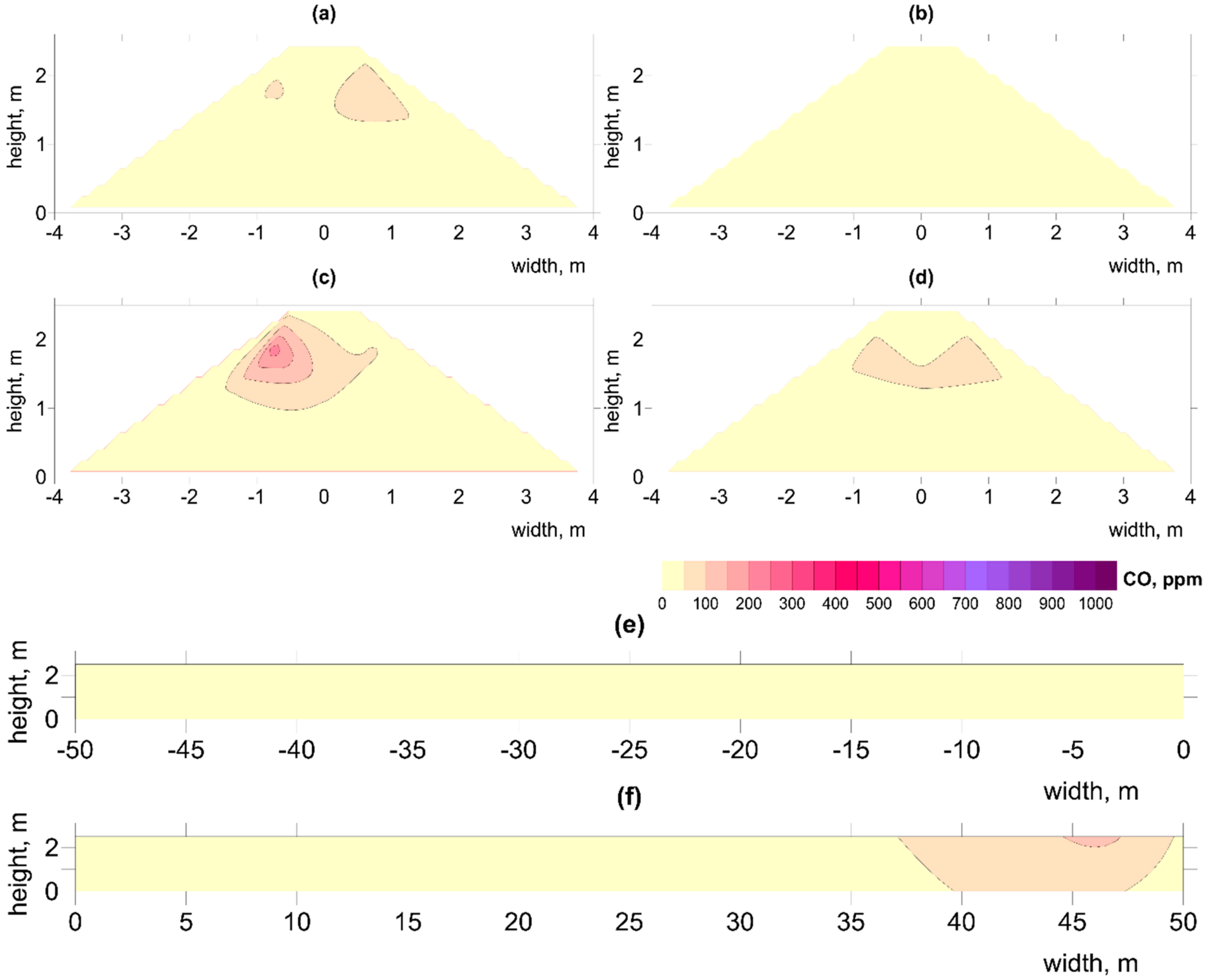

Figure S136. CO distribution in the pile A1, day 50.

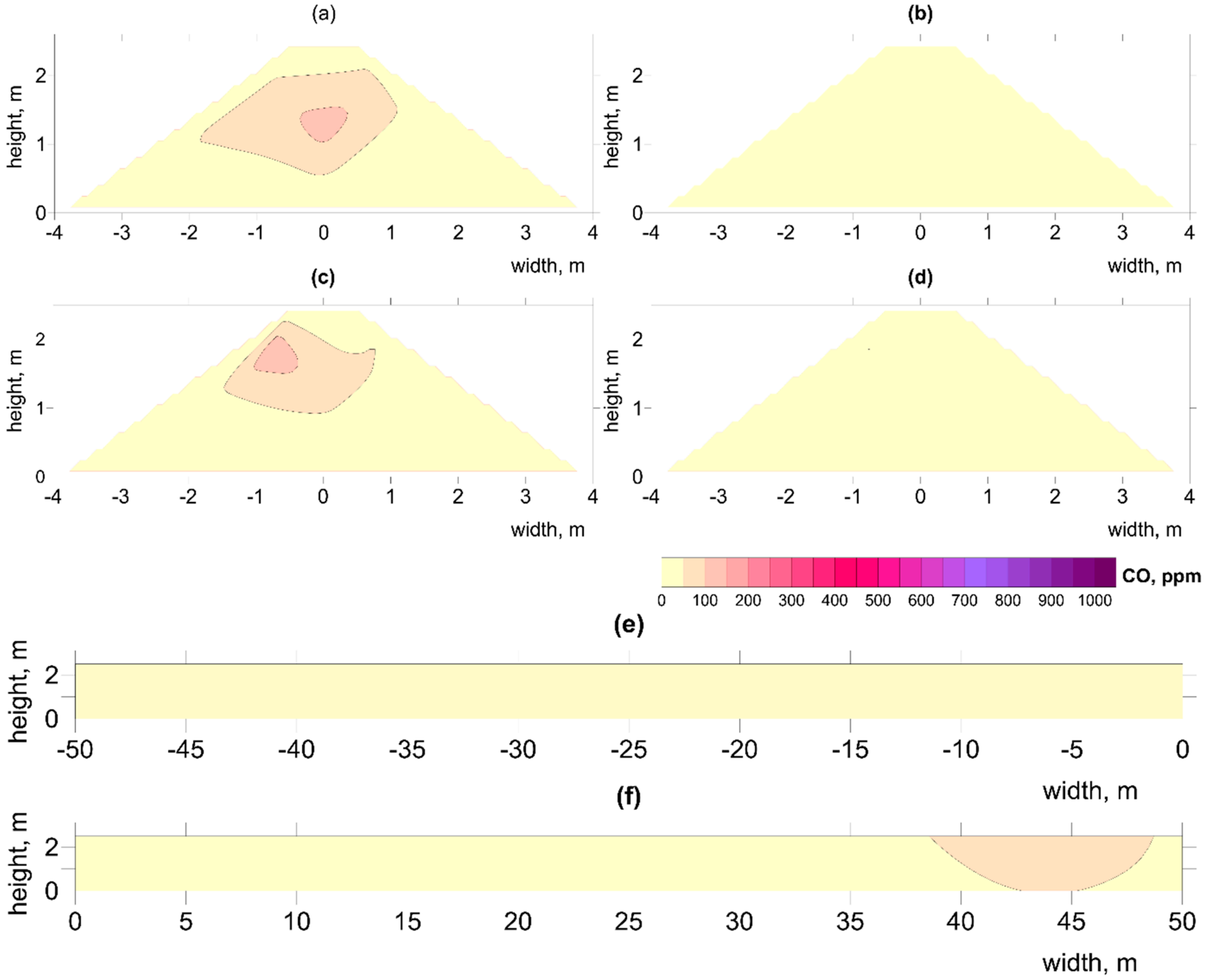

Figure S137. CO distribution in the pile A1, day 60.

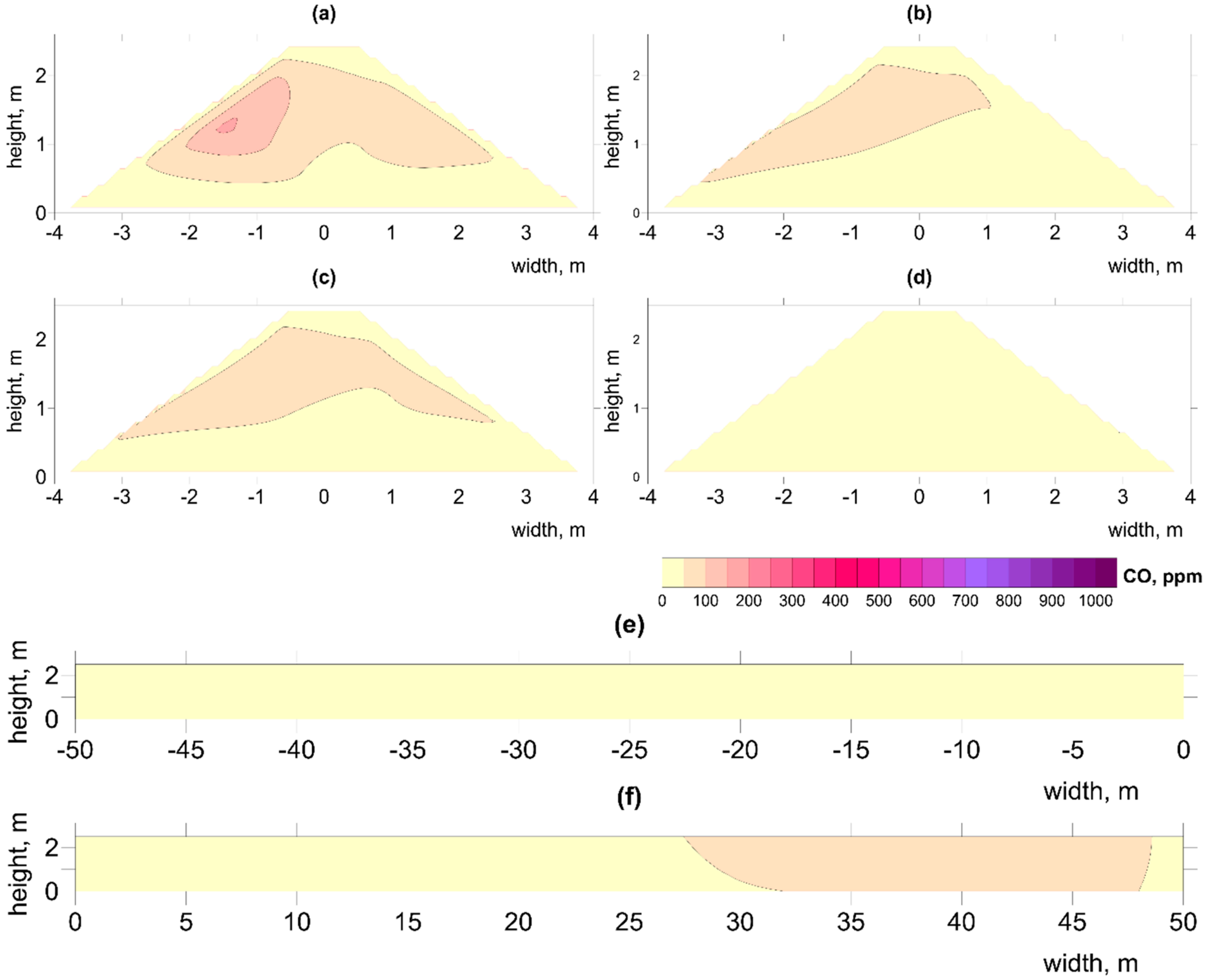

Figure S138. CO distribution in the pile A2, day 1.

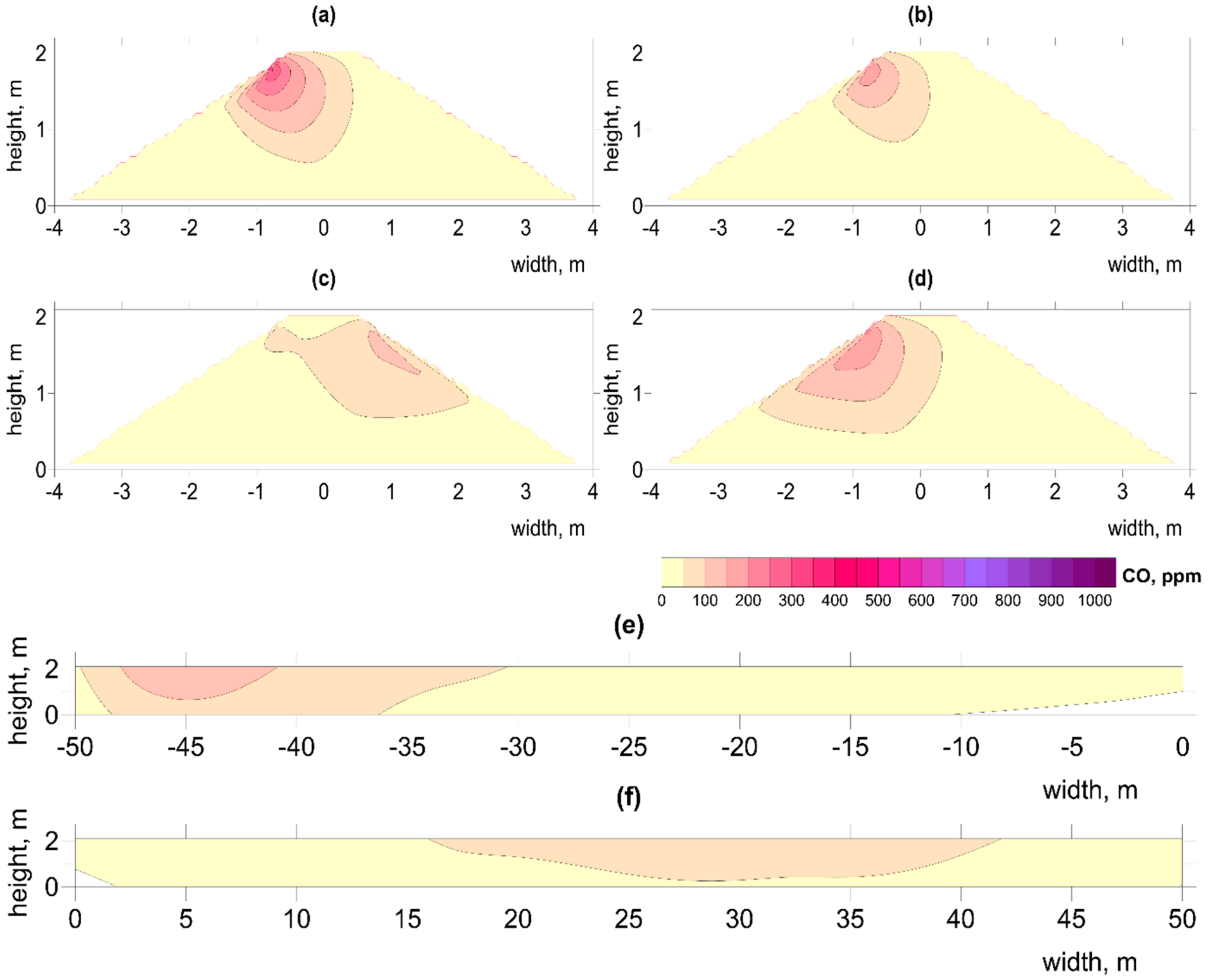

Figure S139. CO distribution in the pile A2, day 11.

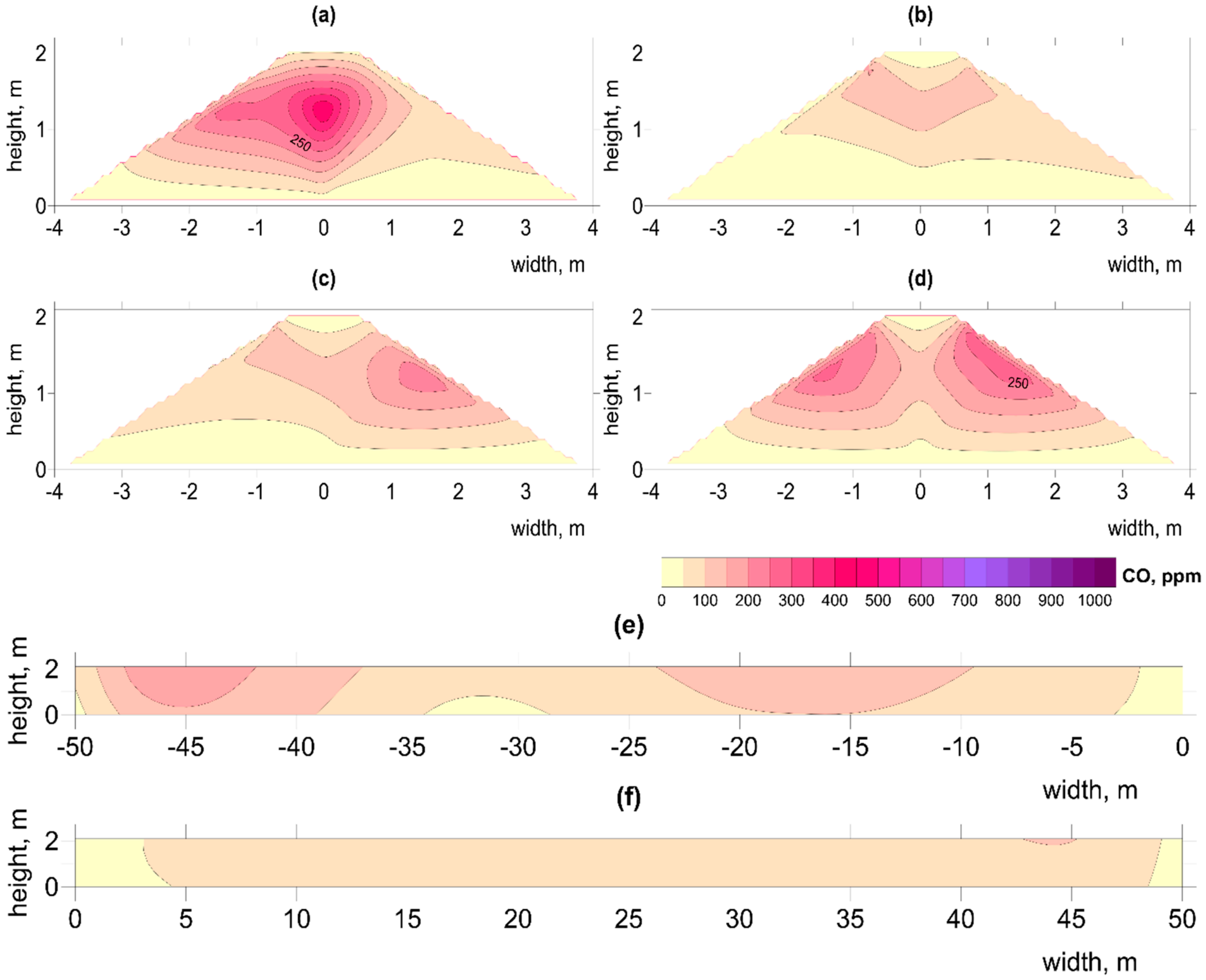

Figure S140. CO distribution in the pile A2, day 17.

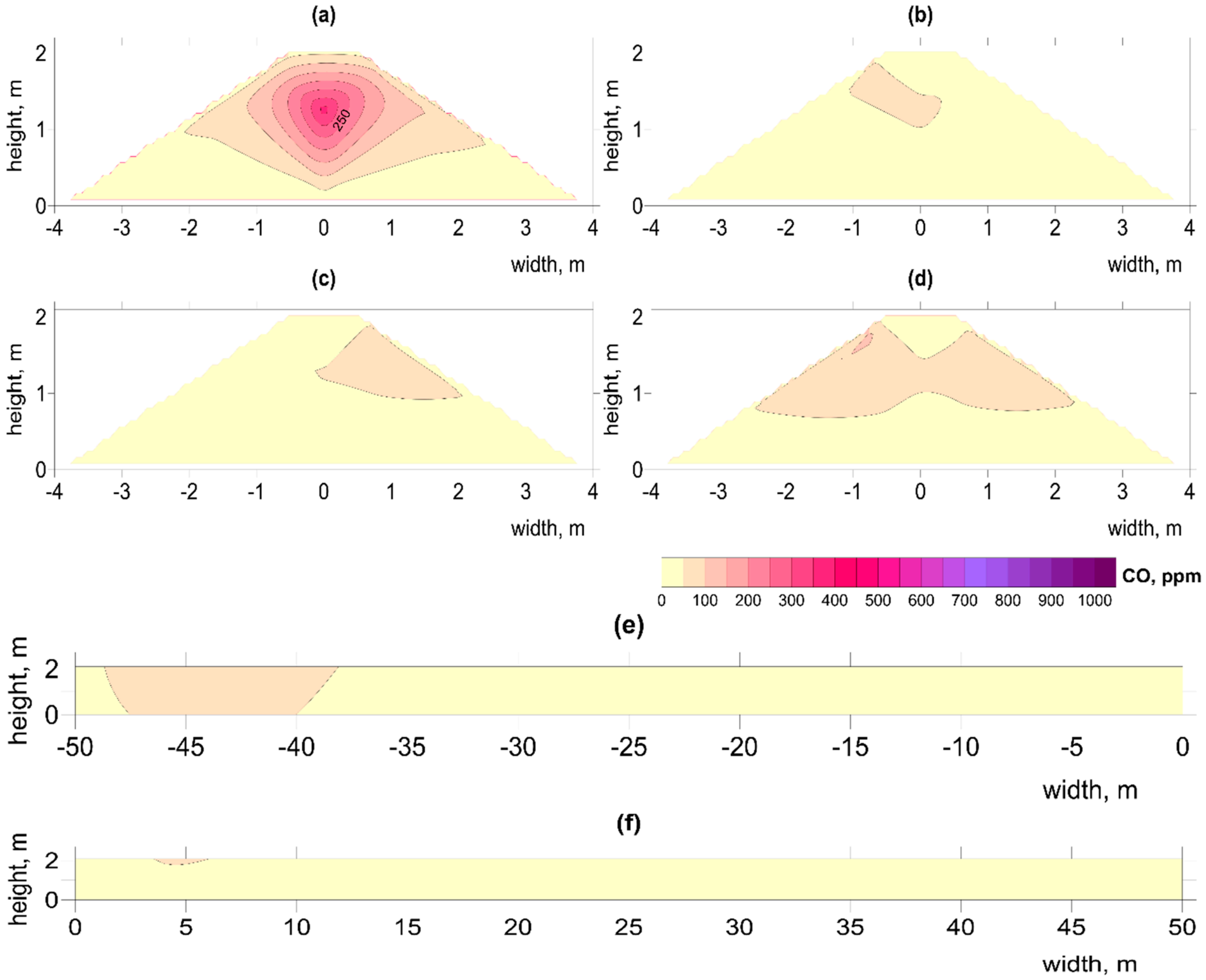

Figure S141. CO distribution in the pile A2, day 25.

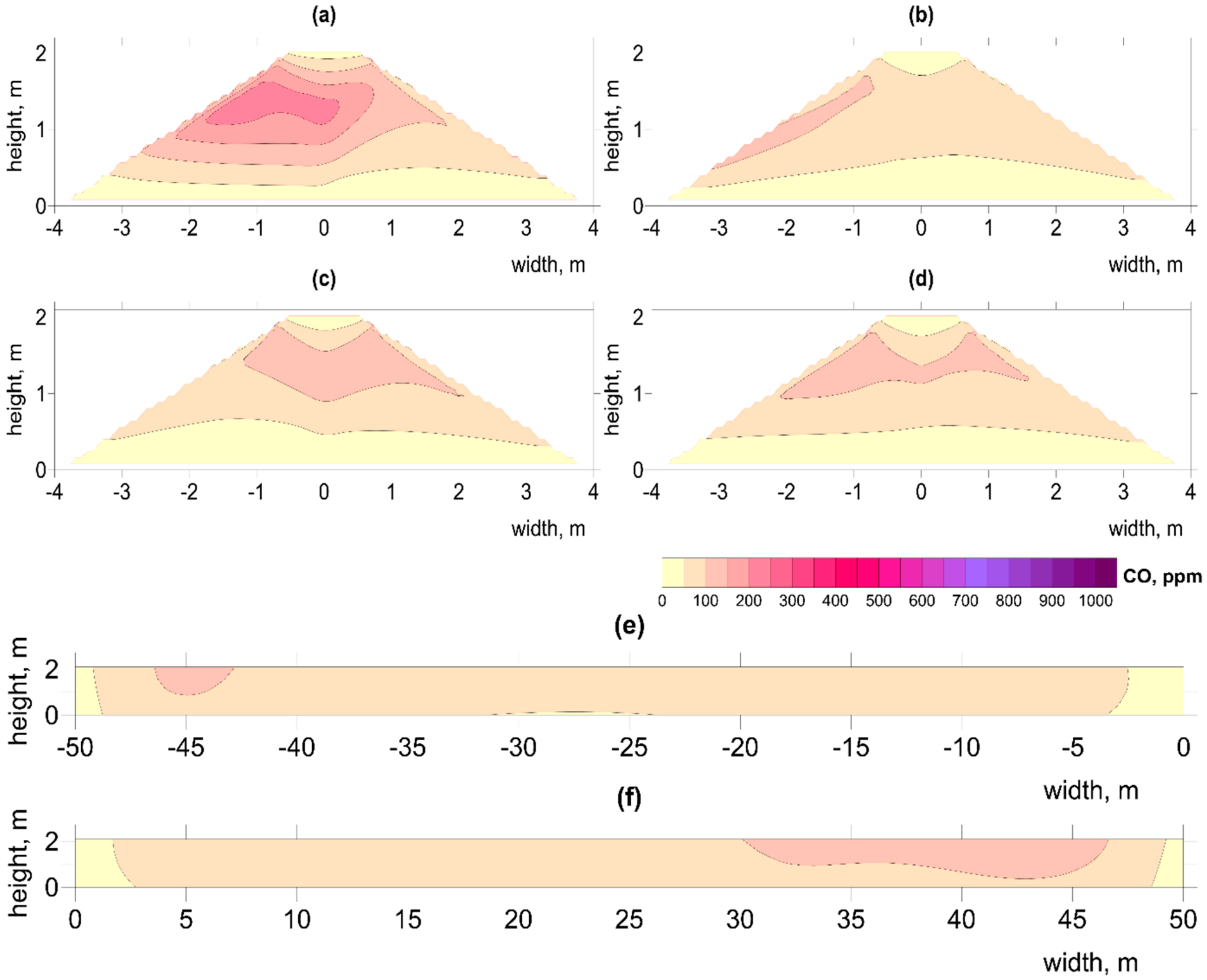

Figure S142. CO distribution in the pile A2, day 32.

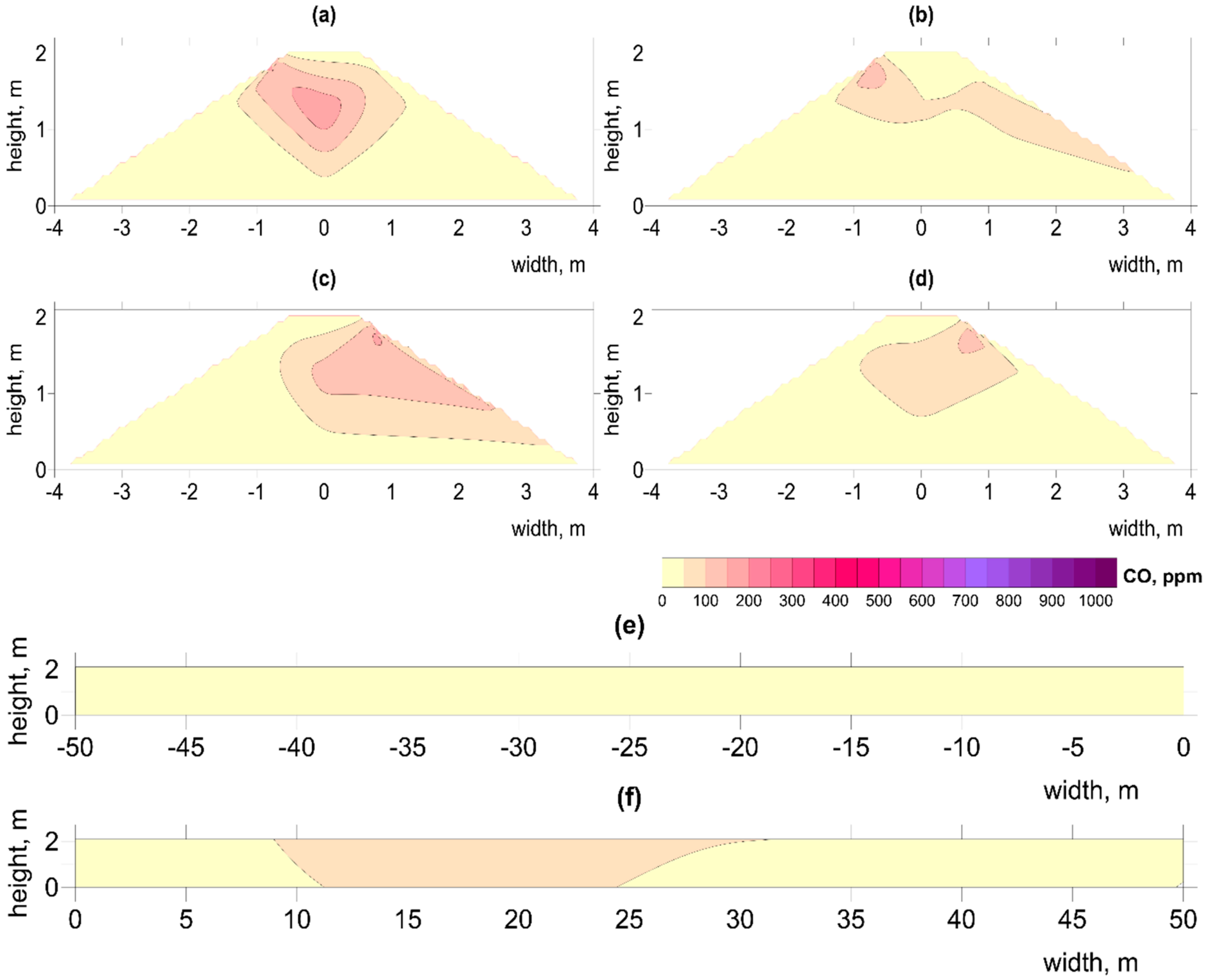

Figure S143. CO distribution in the pile A2, day 38.

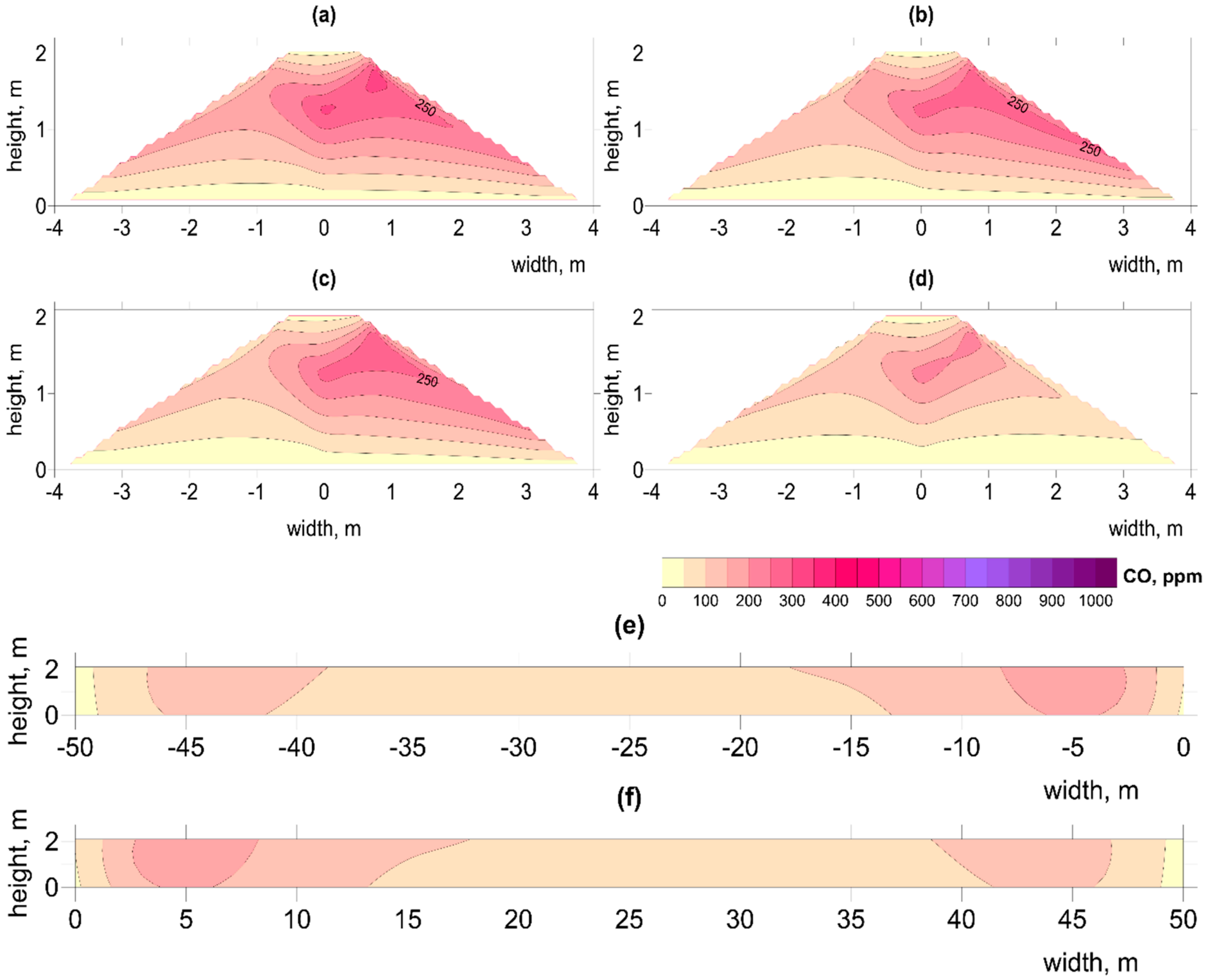

Figure S144. CO distribution in the pile A2, day 47.

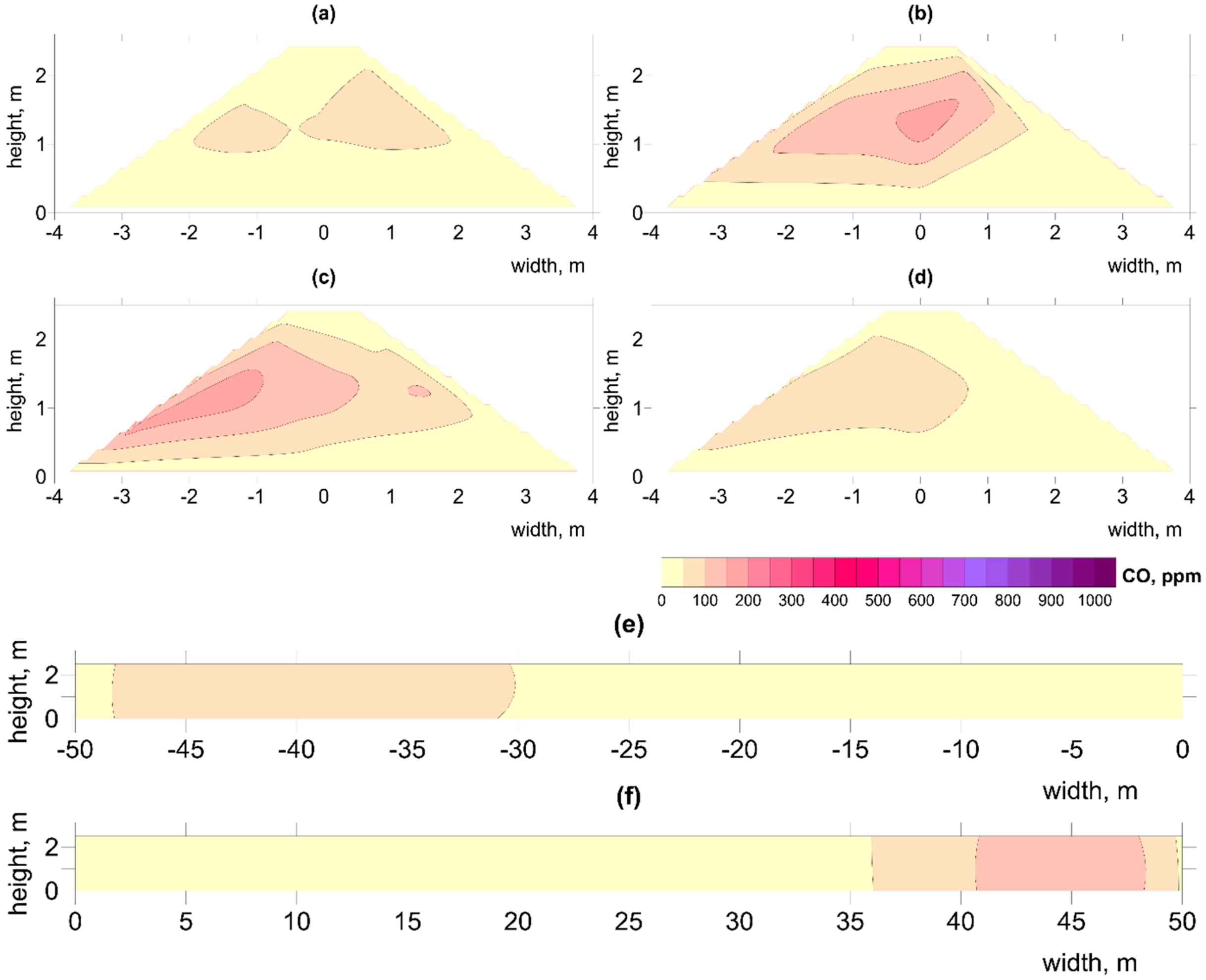

Figure S145. CO distribution in the pile B1, day 1.

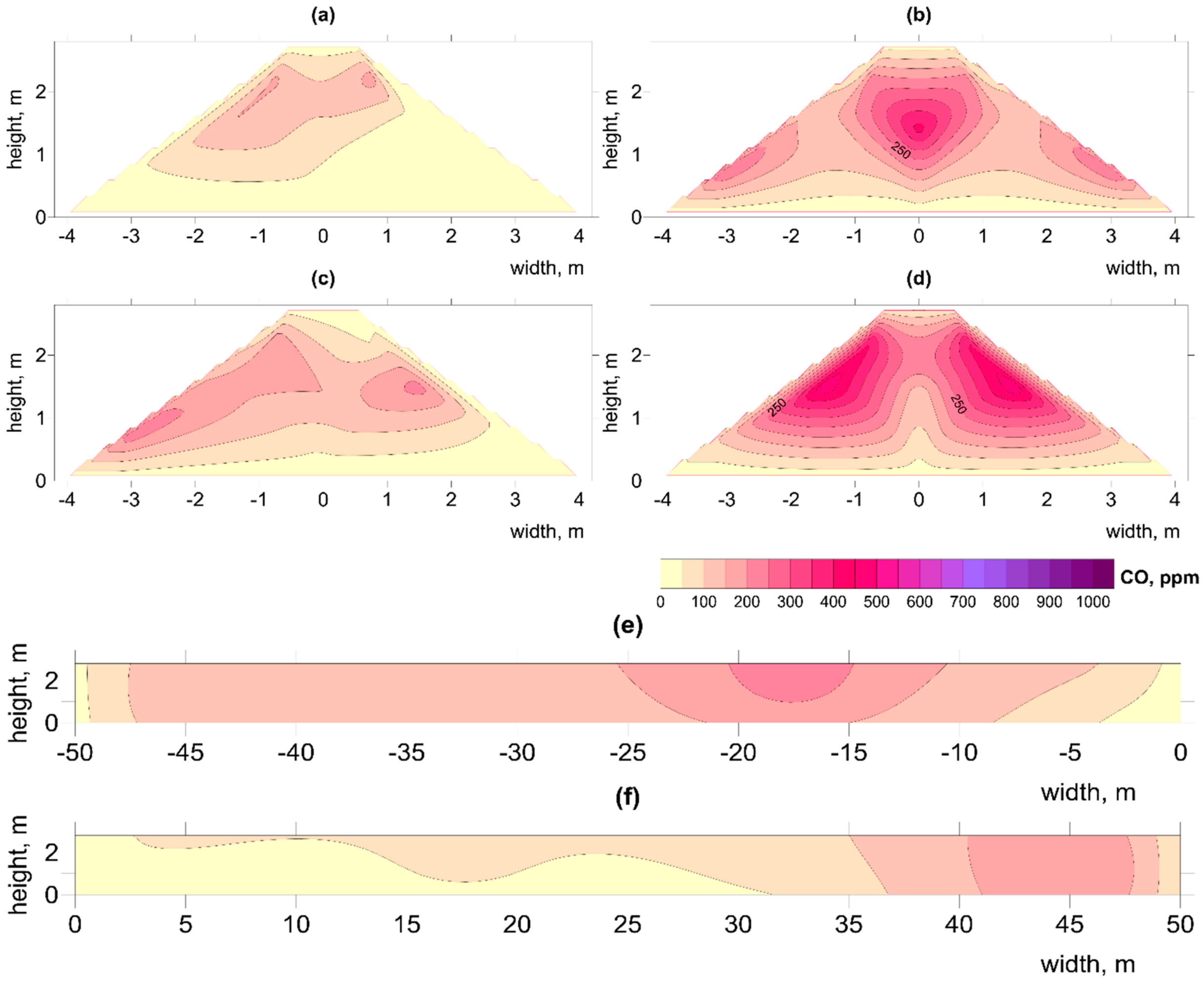

Figure S146. CO distribution in the pile B1, day 9.

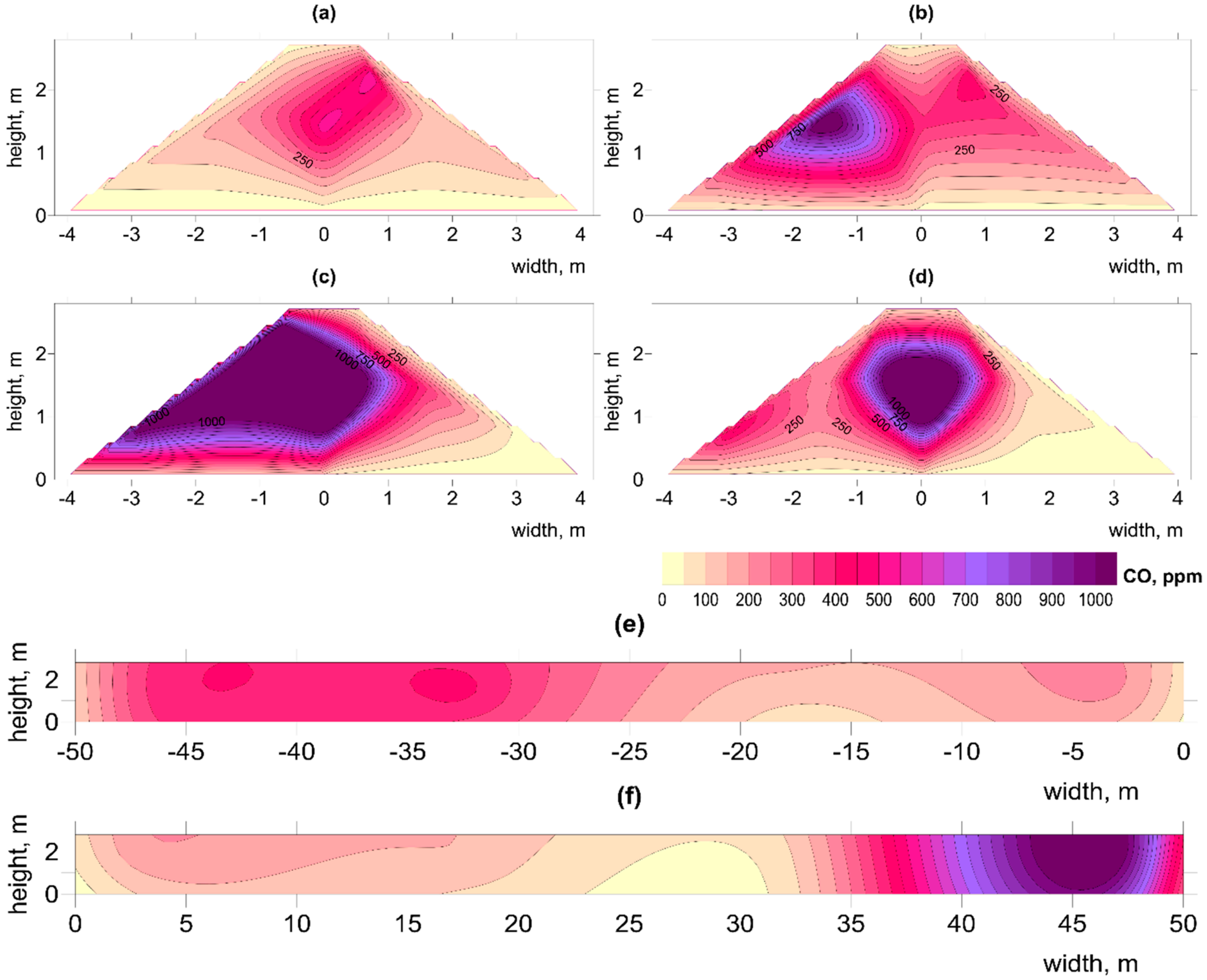

Figure S147. CO distribution in the pile B1, day 31.

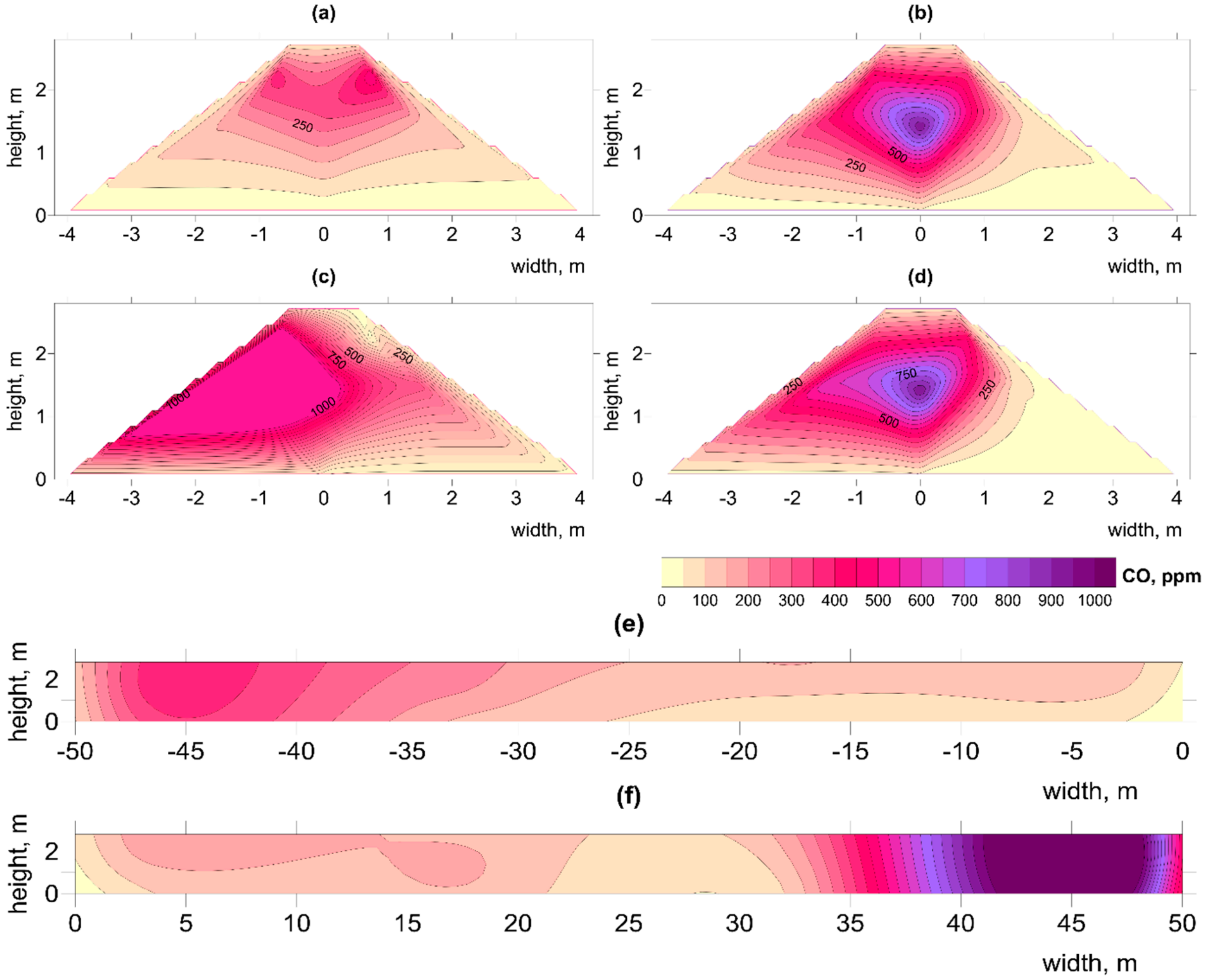

Figure S148. CO distribution in the pile B1, day 41.

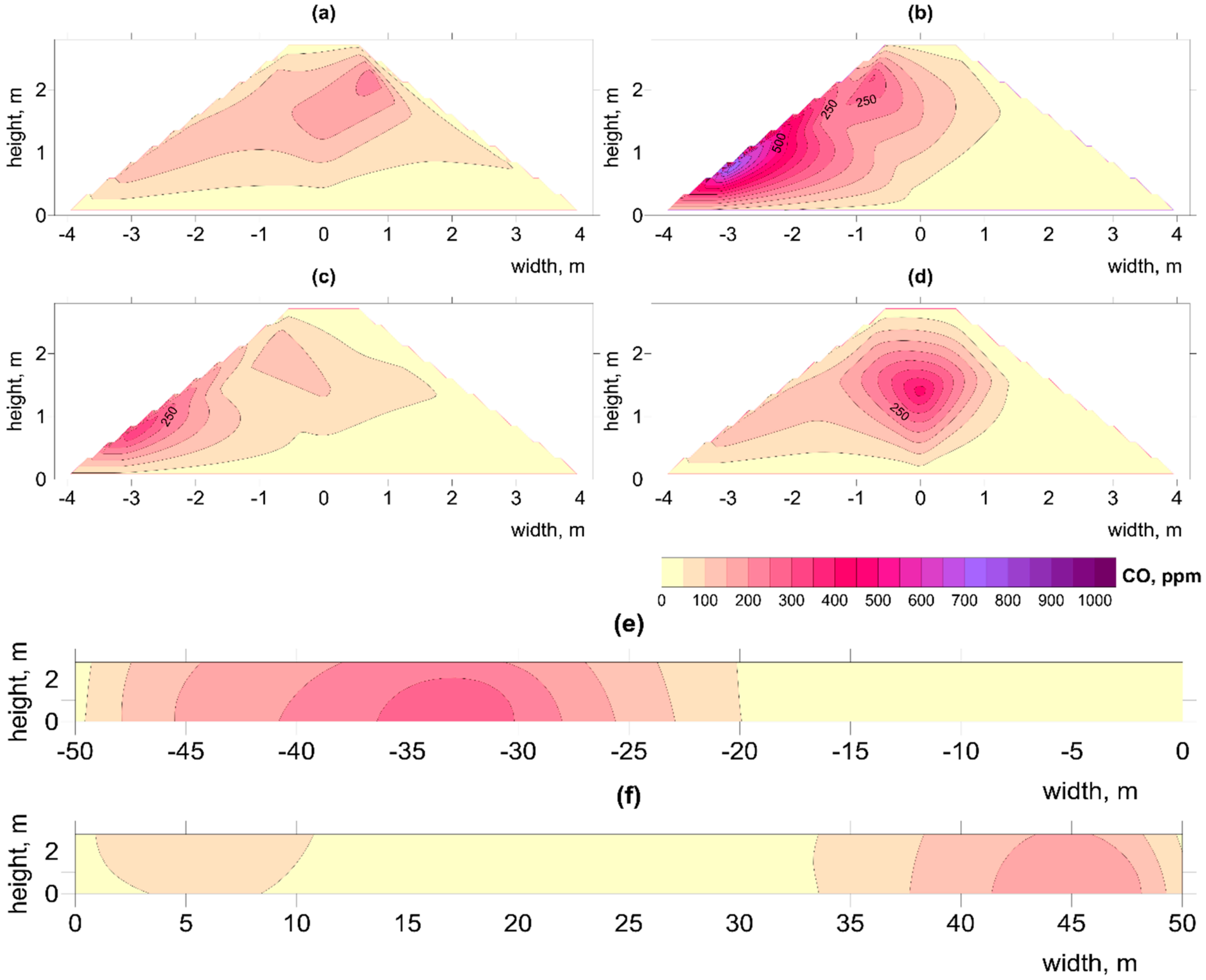

Figure S149. CO distribution in the pile B1, day 57.

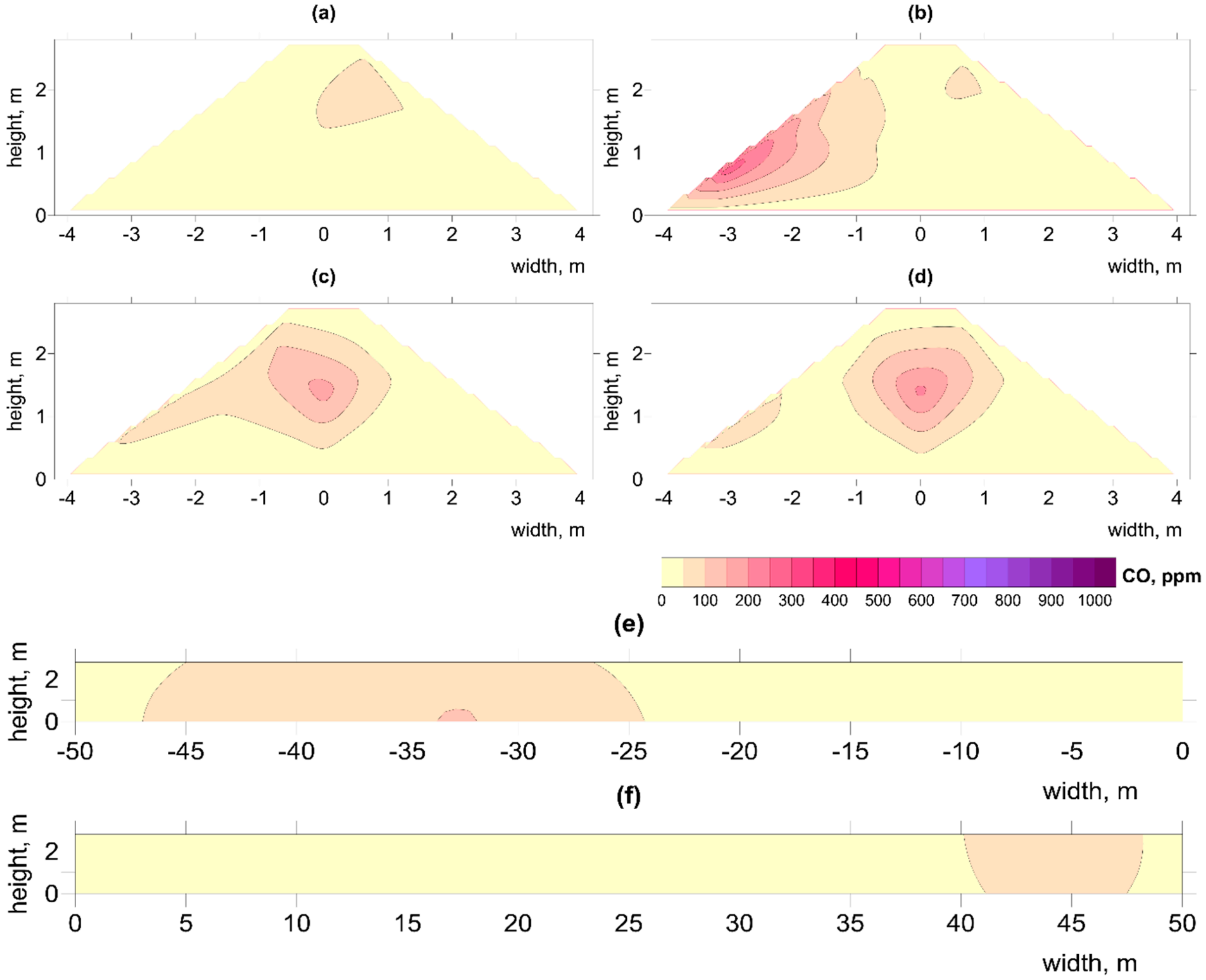

Figure S150. CO distribution in the pile B1, day 64.

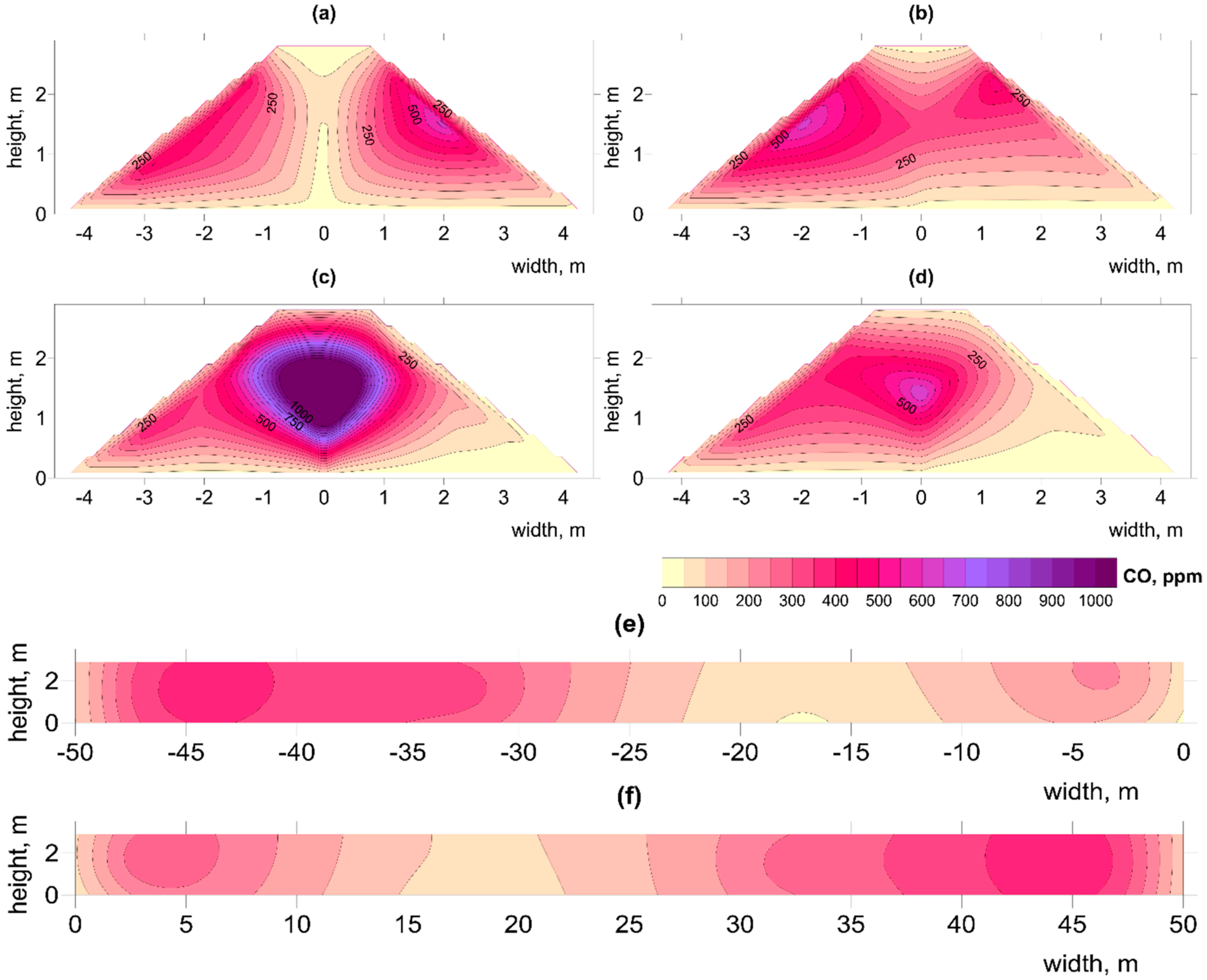

Figure S151. CO distribution in the pile B2, day 1.

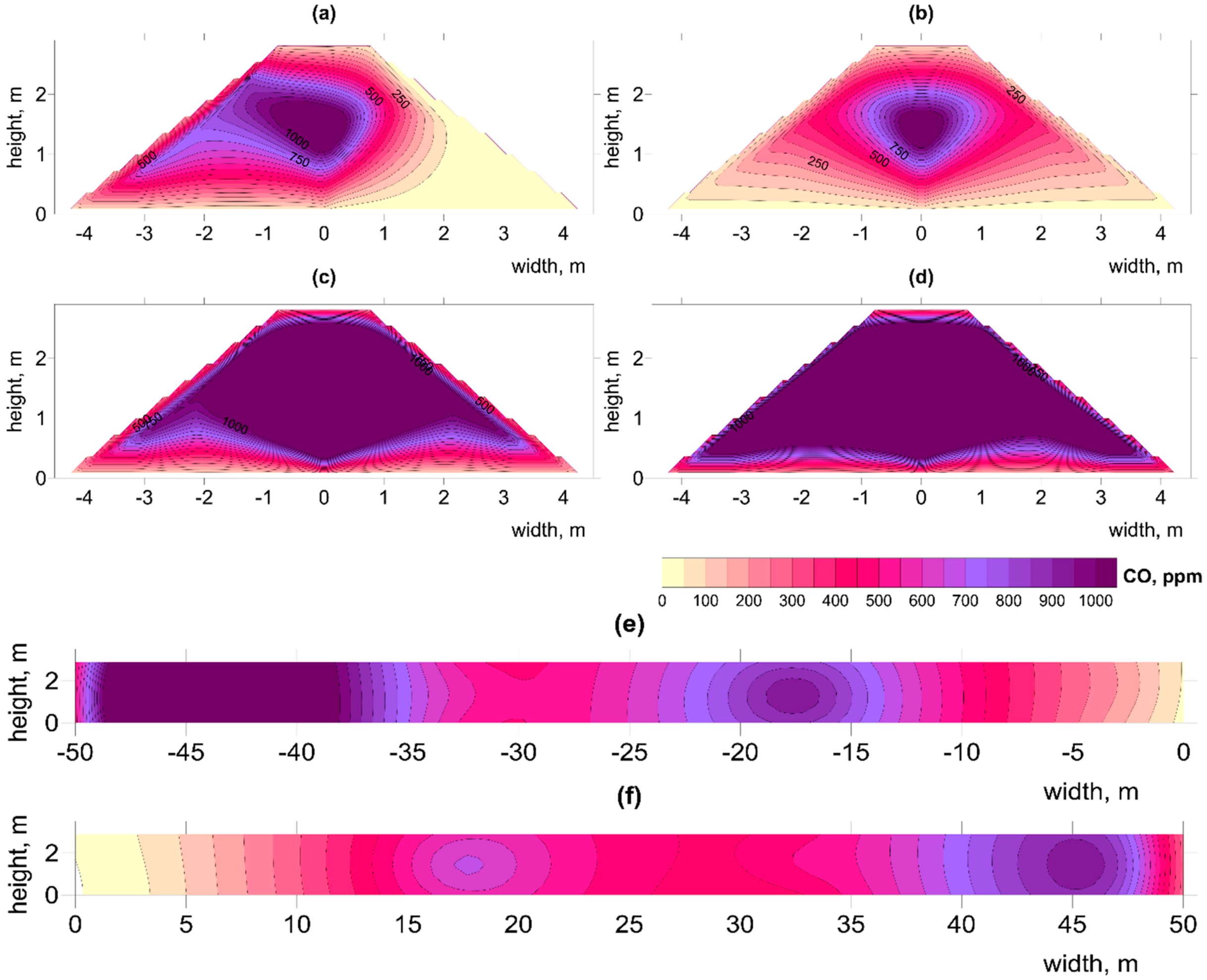

Figure S152. CO distribution in the pile B2, day 8.

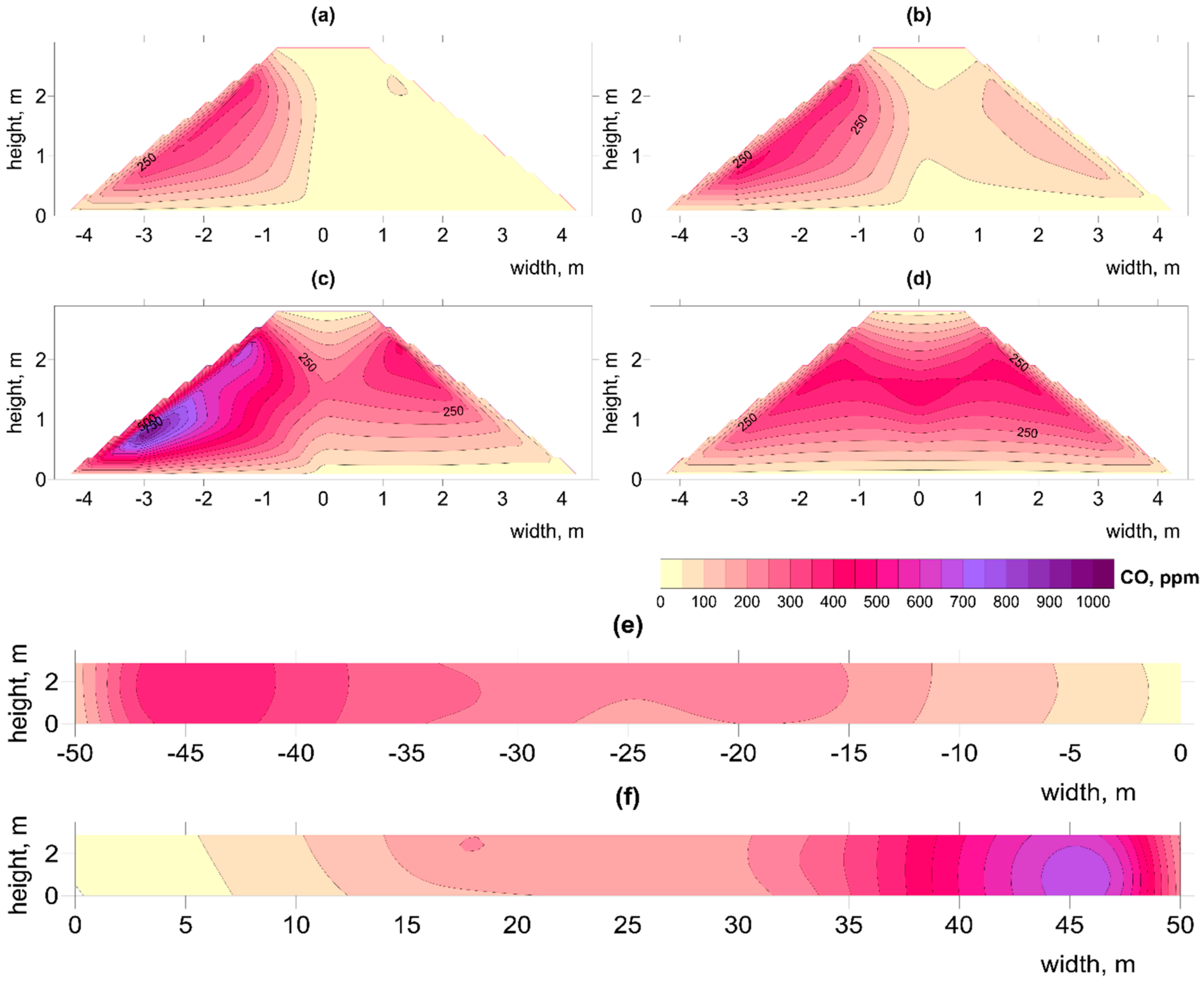

Figure S153. CO distribution in the pile B2, day 16.

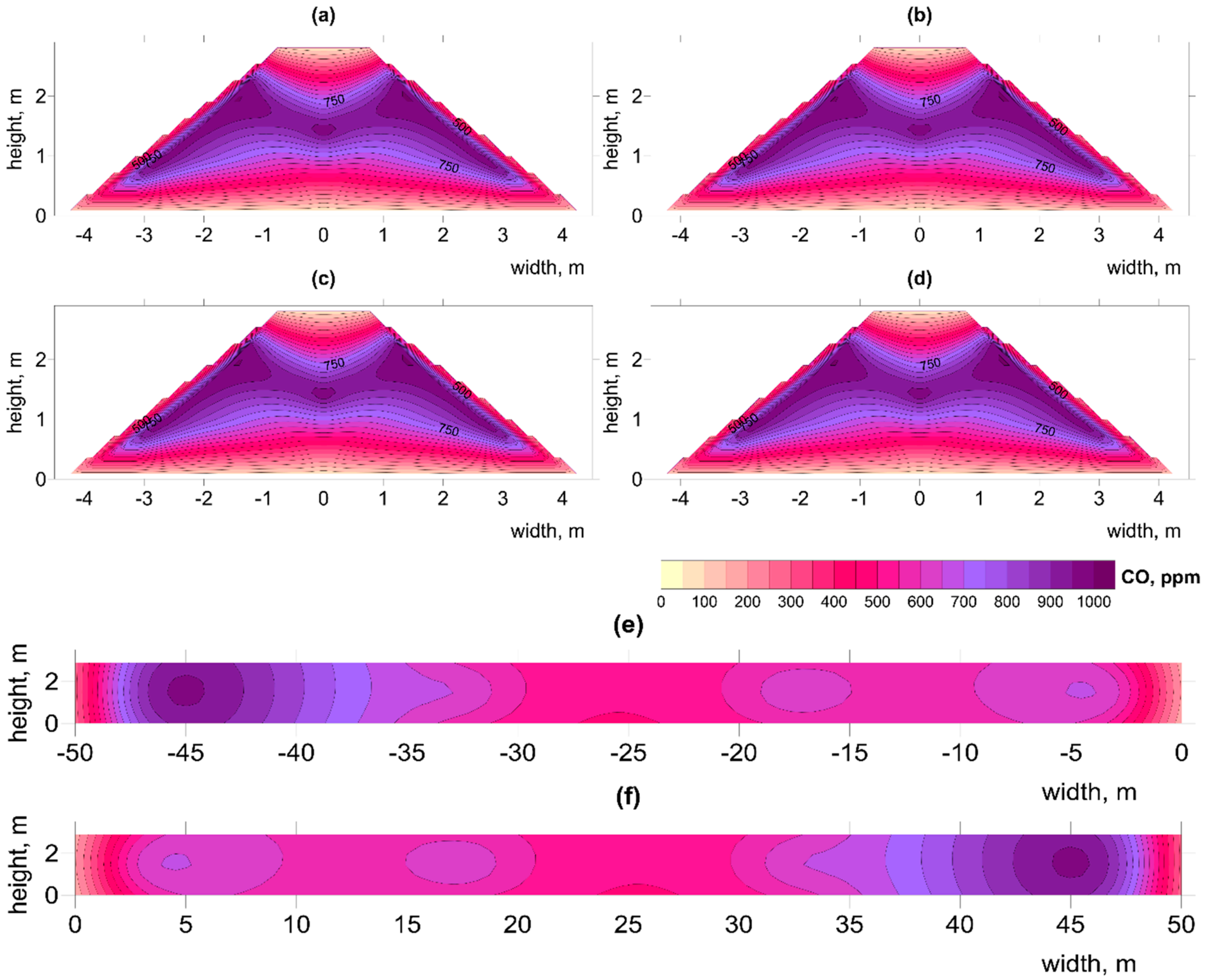

Figure S154. CO distribution in the pile B2, day 24.

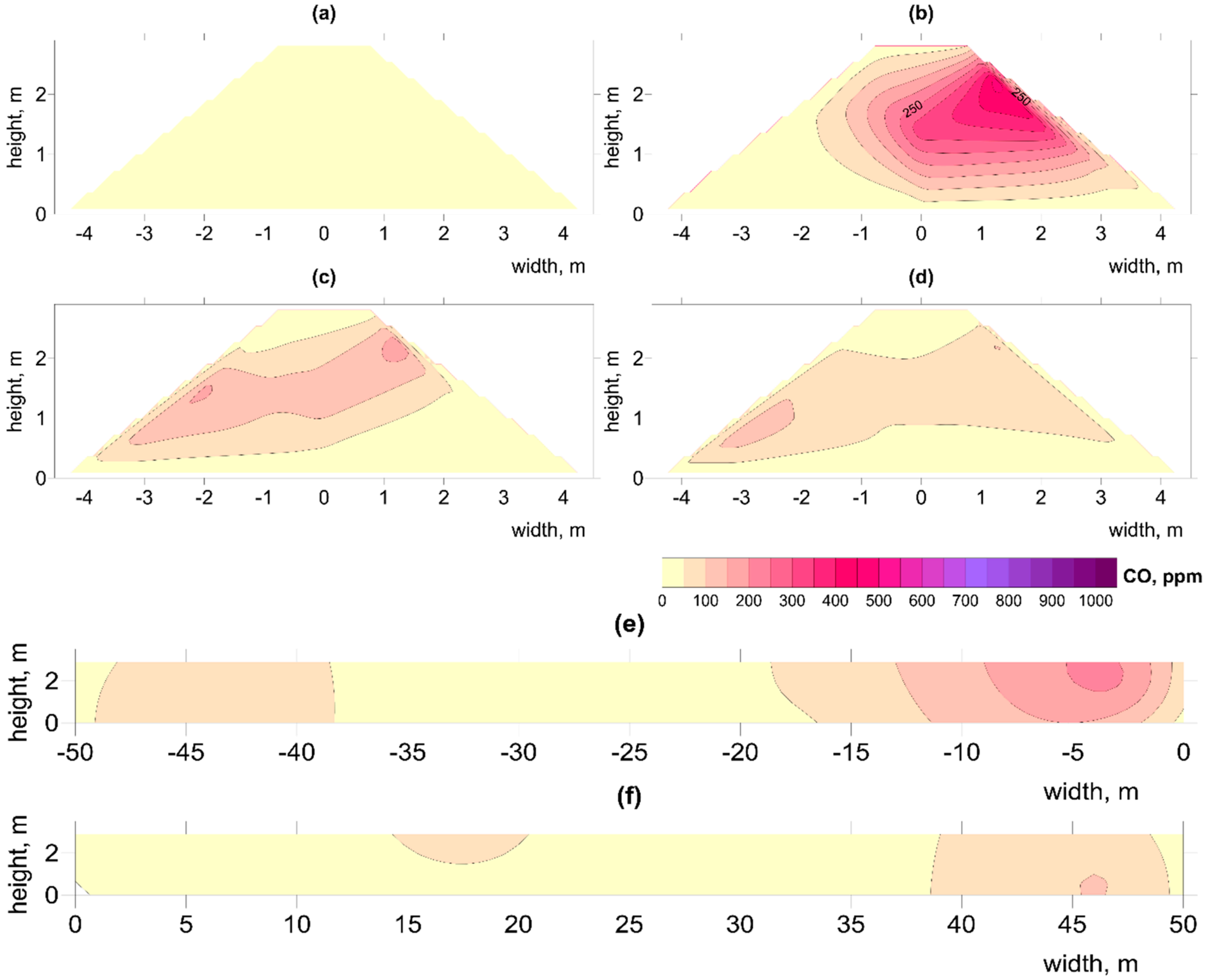

Figure S155. CO distribution in the pile B2, day 36.

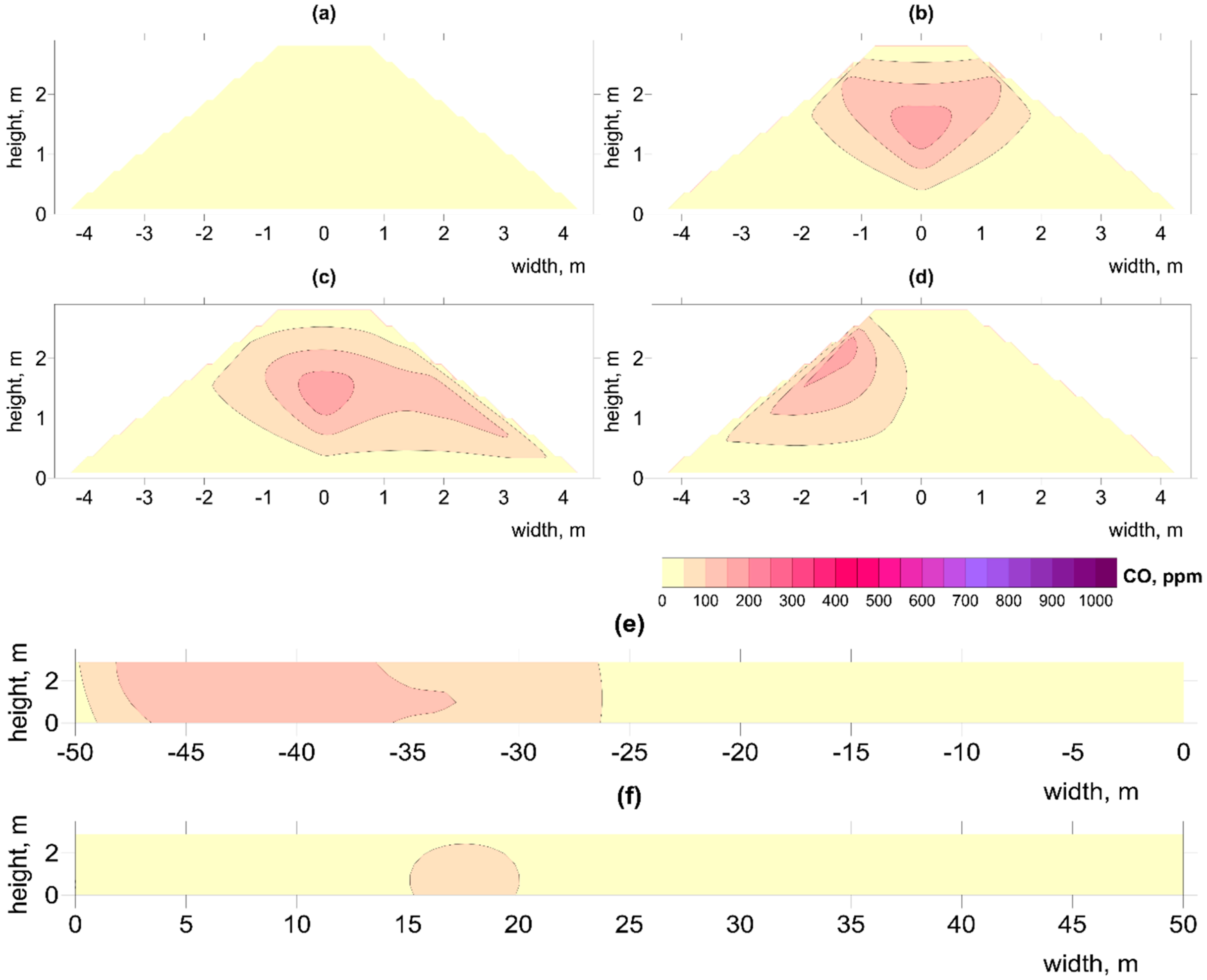

Figure S156. CO distribution in the pile B2, day 43.

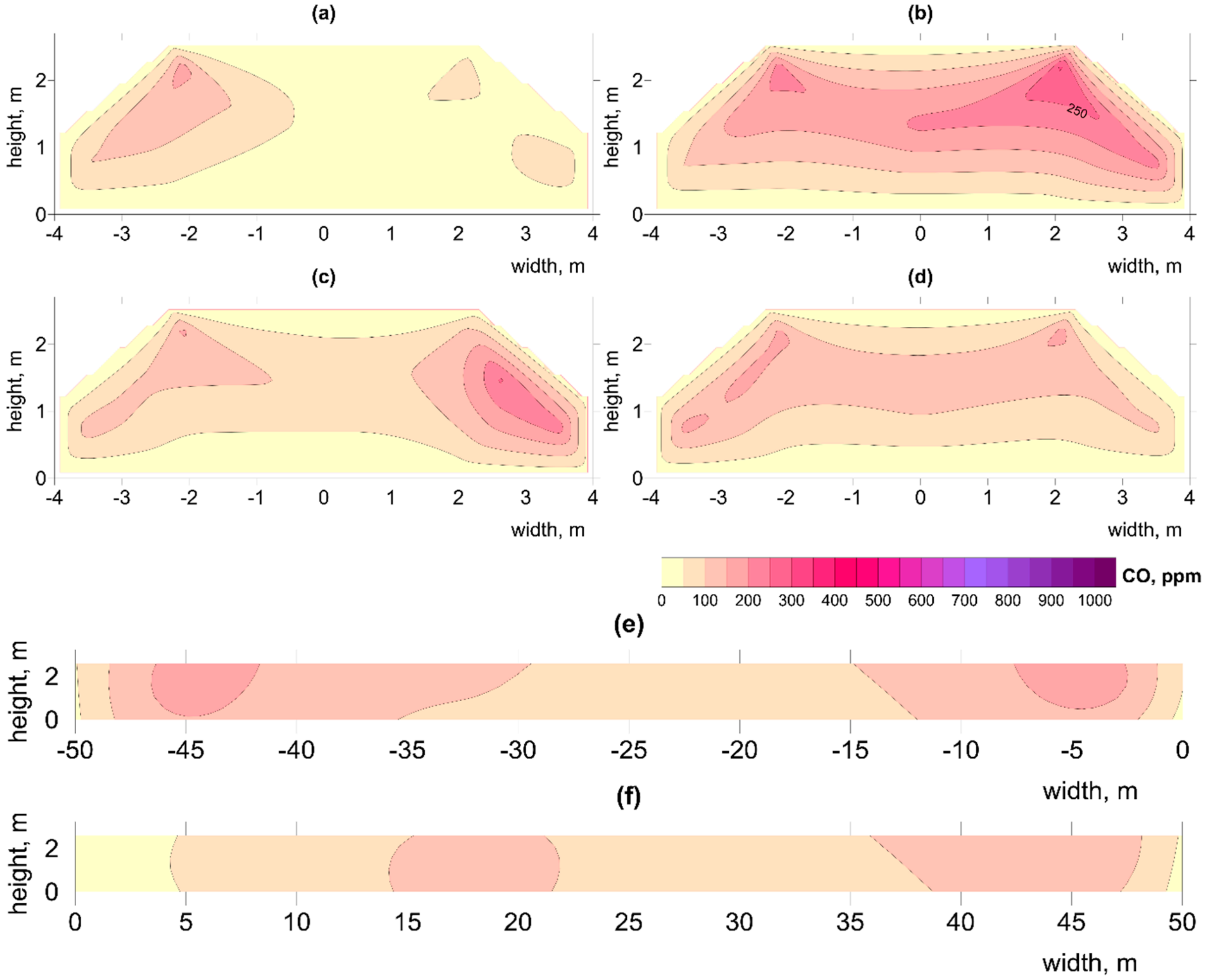

Figure S157. CO distribution in the pile C1, day 1.

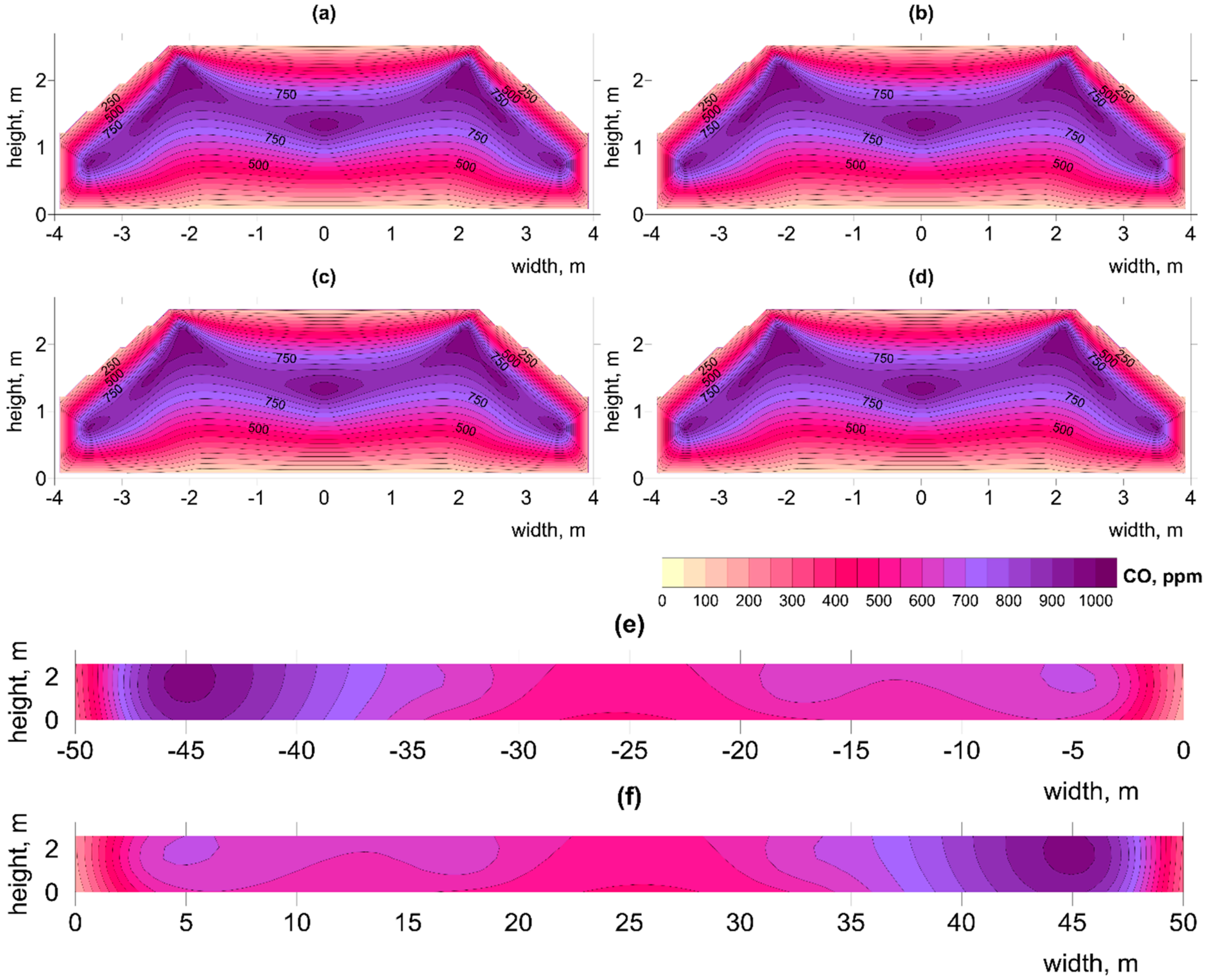

Figure S158. CO distribution in the pile C1, day 9.

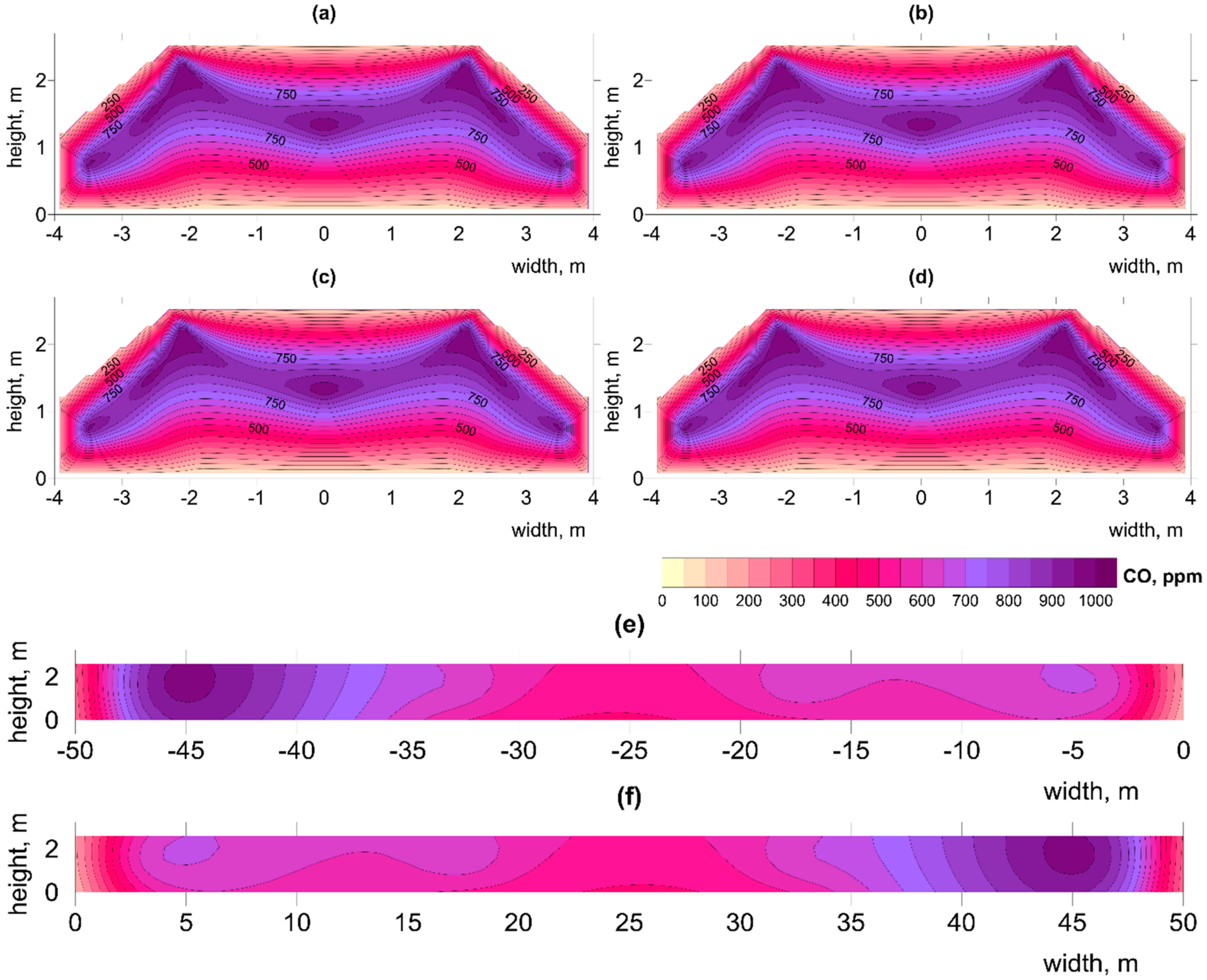

Figure S159. CO distribution in the pile C1, day 16.

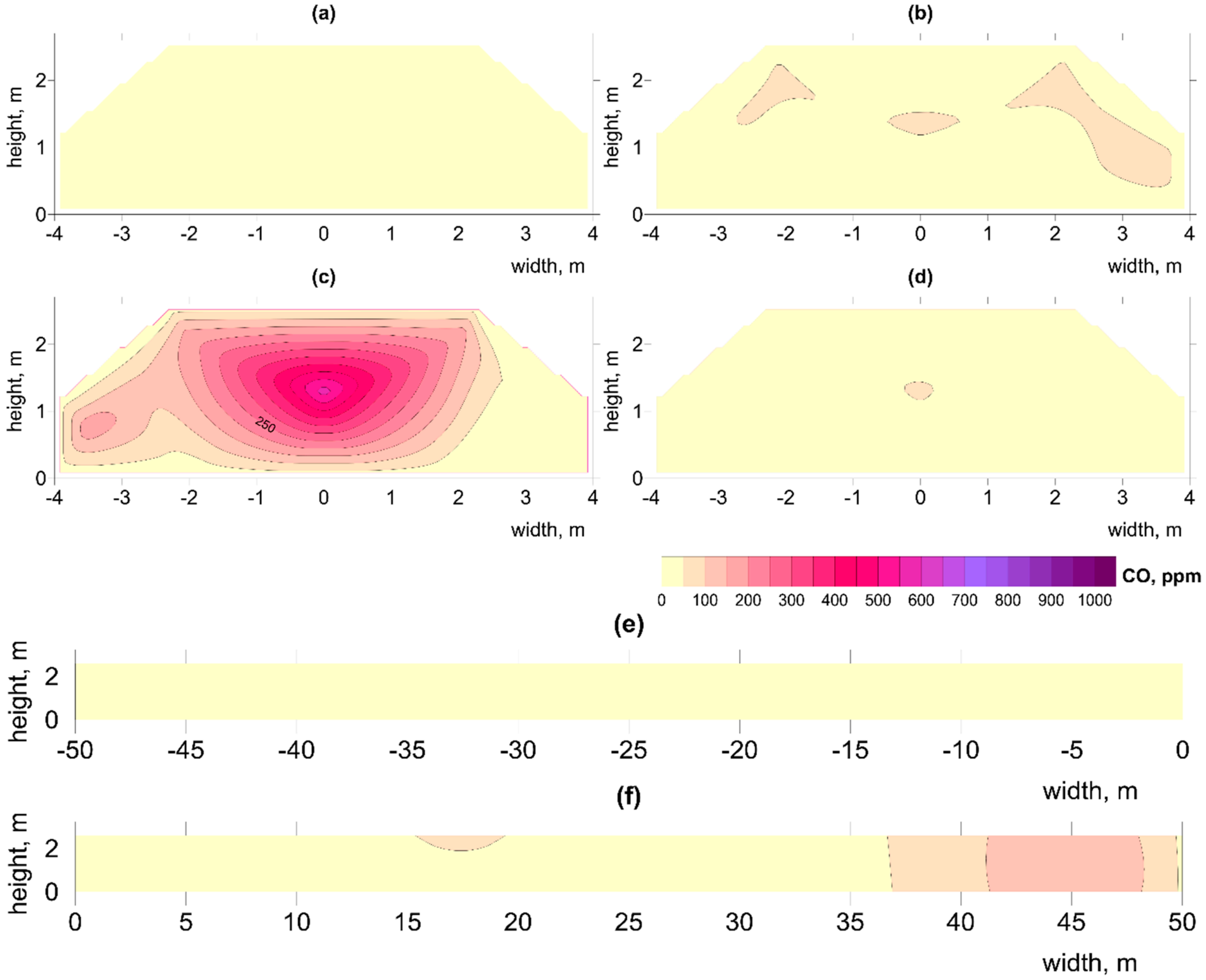

Figure S160. CO distribution in the pile C1, day 28.

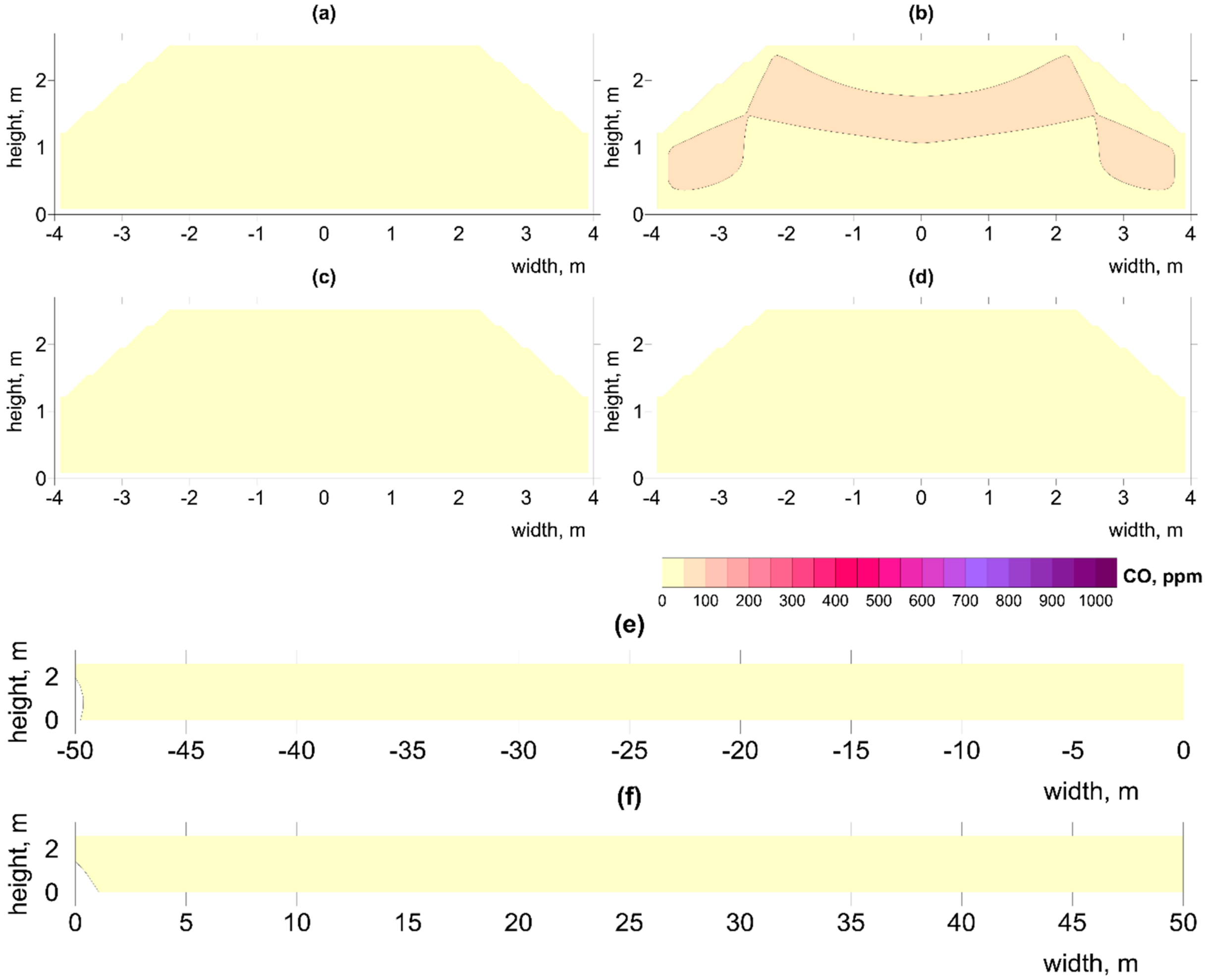

Figure S161. CO distribution in the pile C1, day 35.

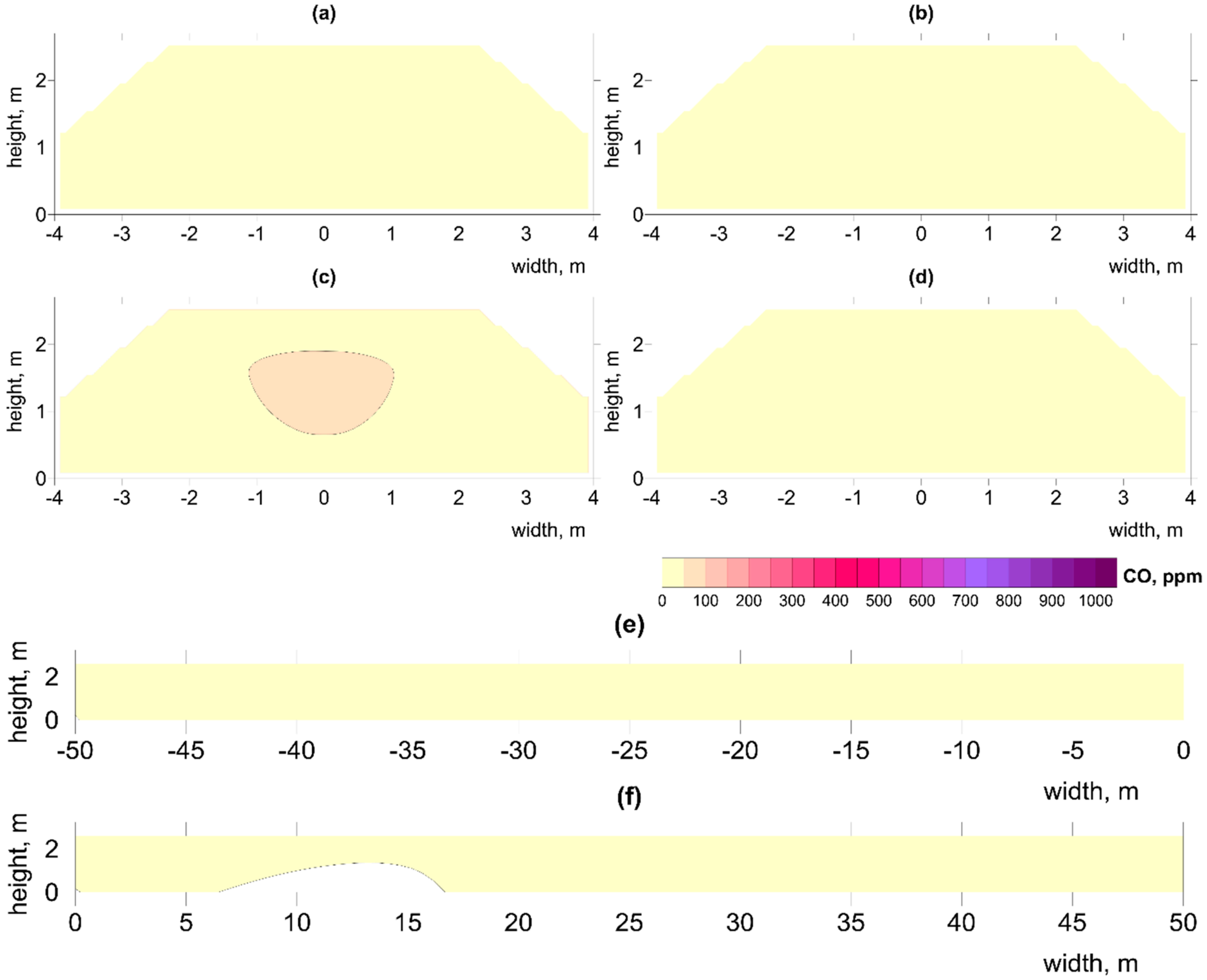

Figure S162. CO distribution in the pile C1, day 42.

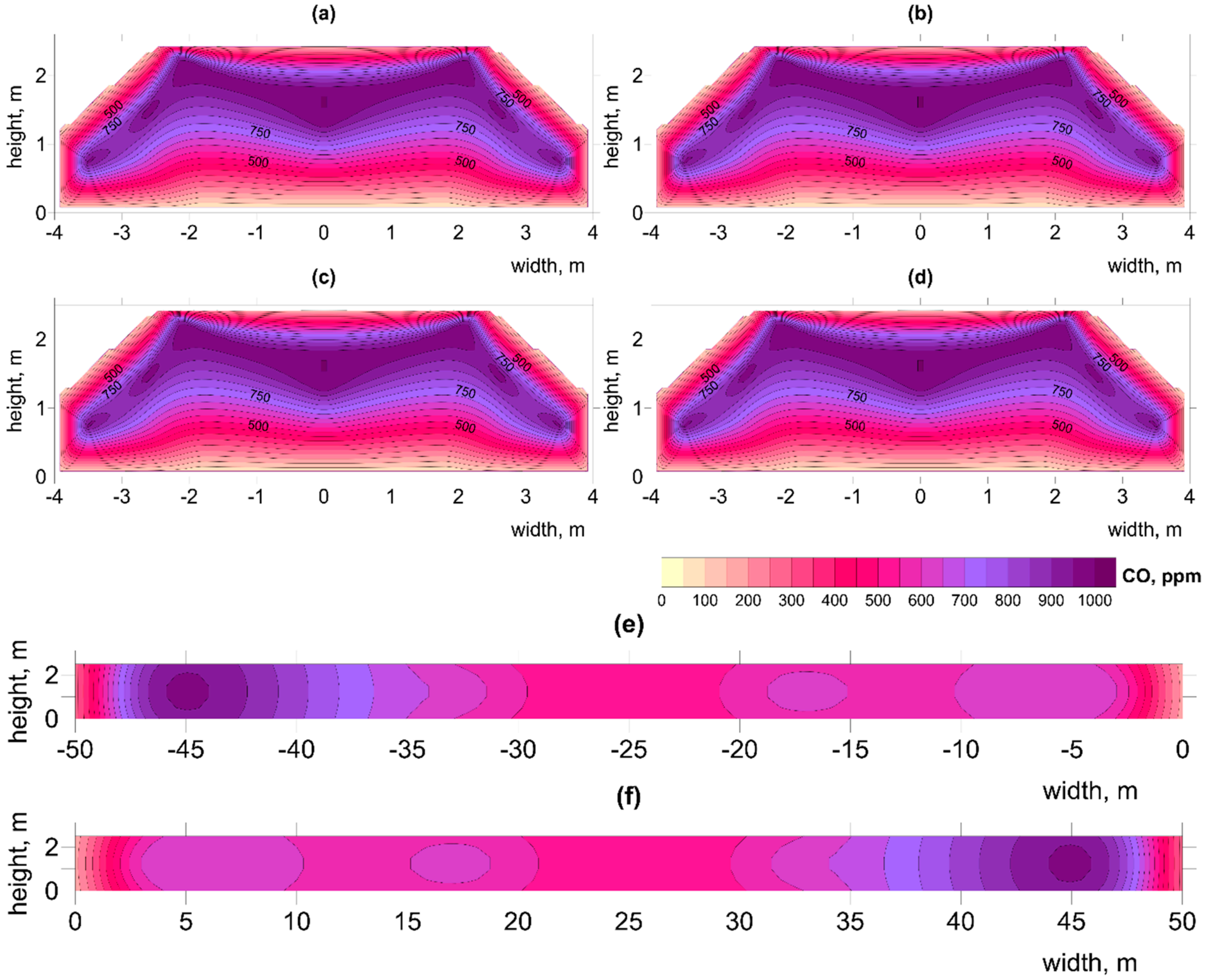

Figure S163. CO distribution in the pile C2, day 1.

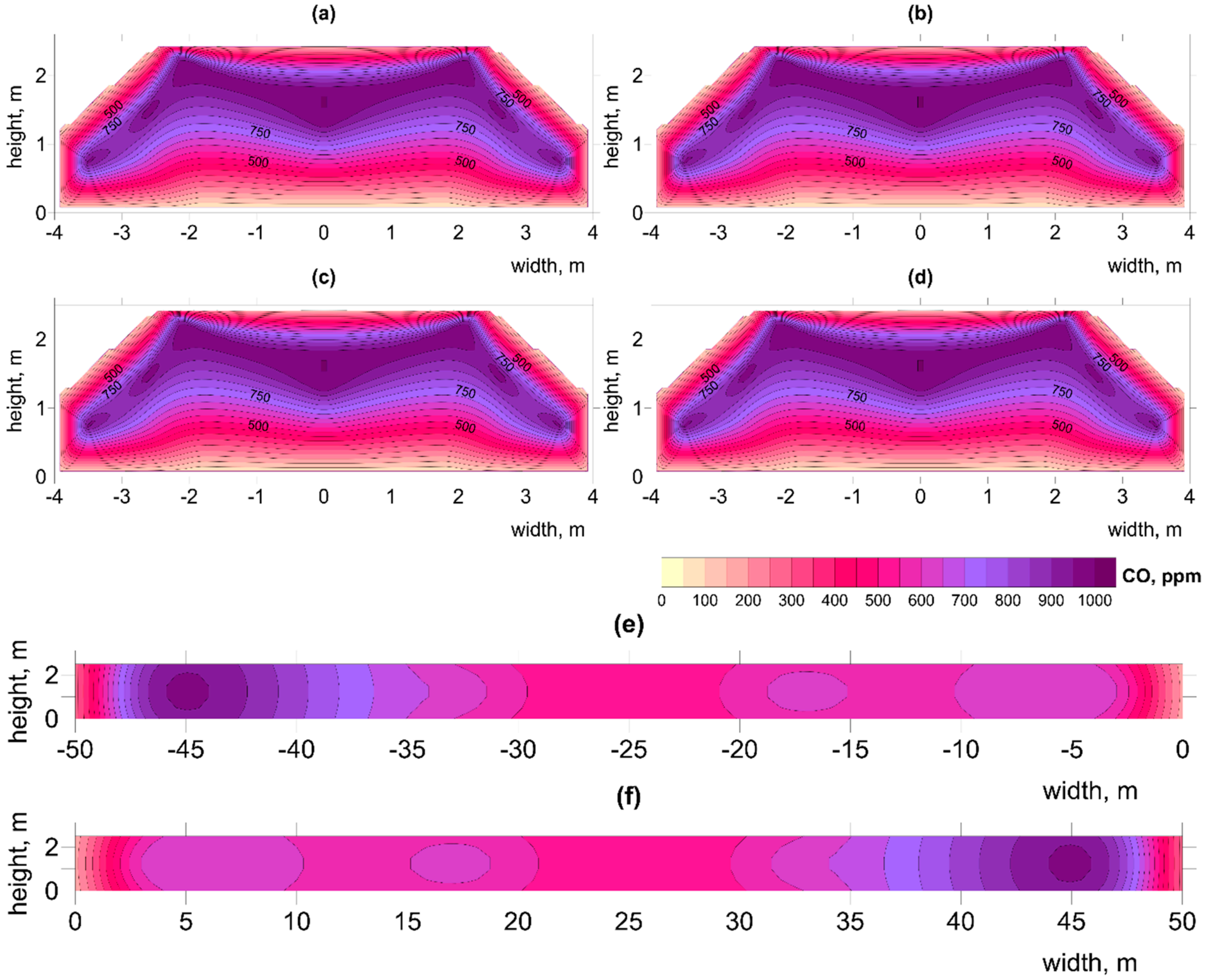

Figure S164. CO distribution in the pile C2, day 9.

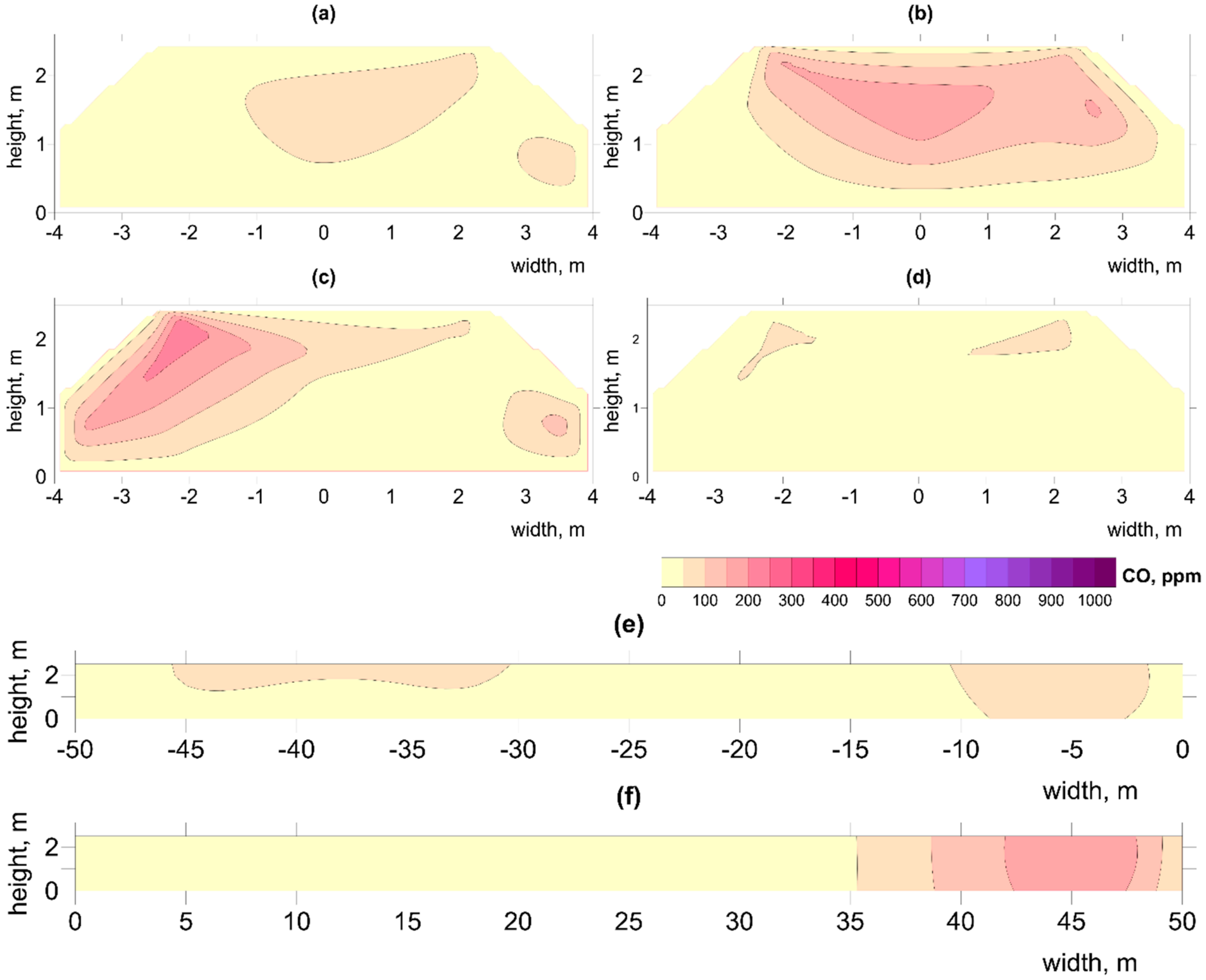

Figure S165. CO distribution in the pile C2, day 21.

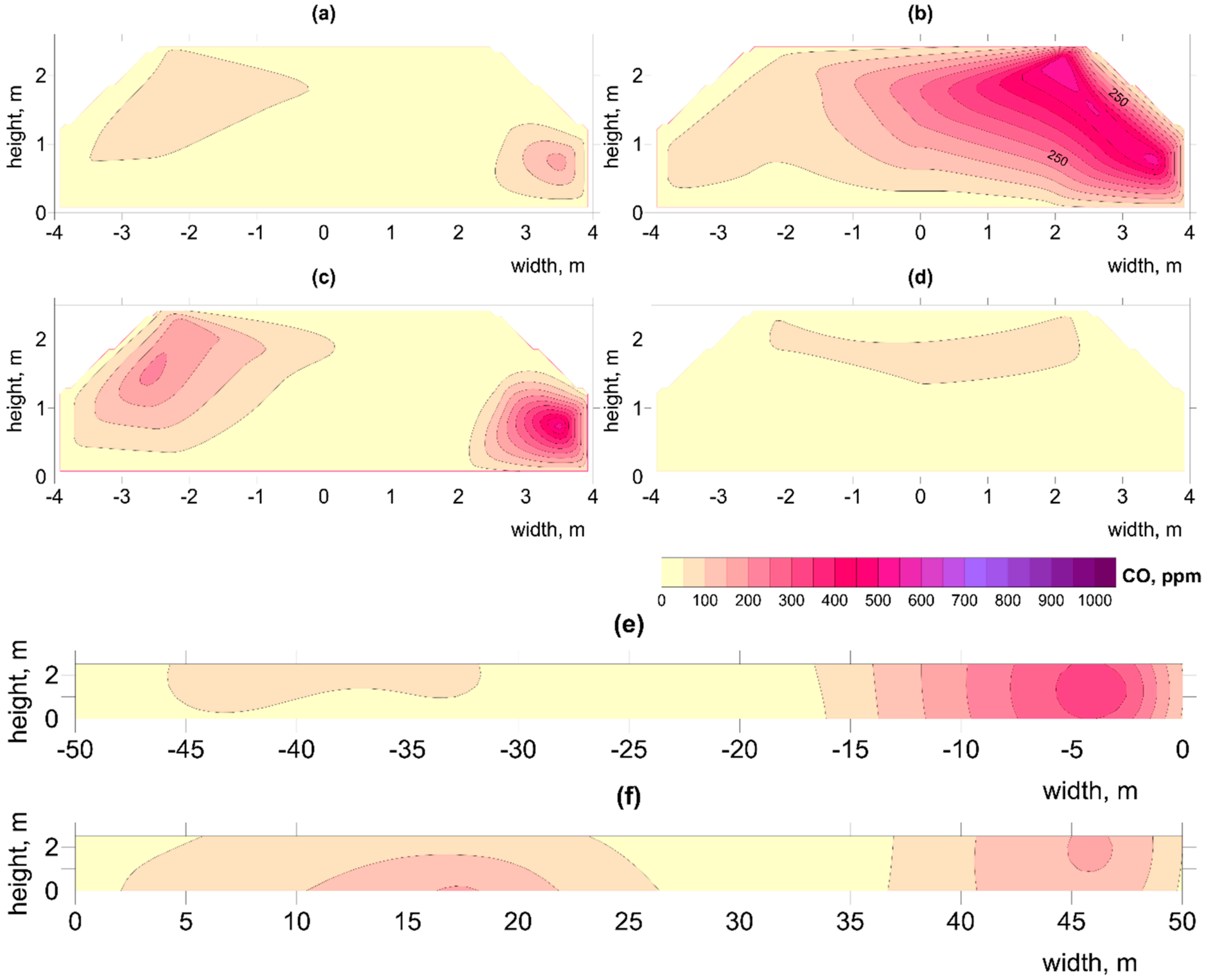

Figure S166. CO distribution in the pile C2, day 28.

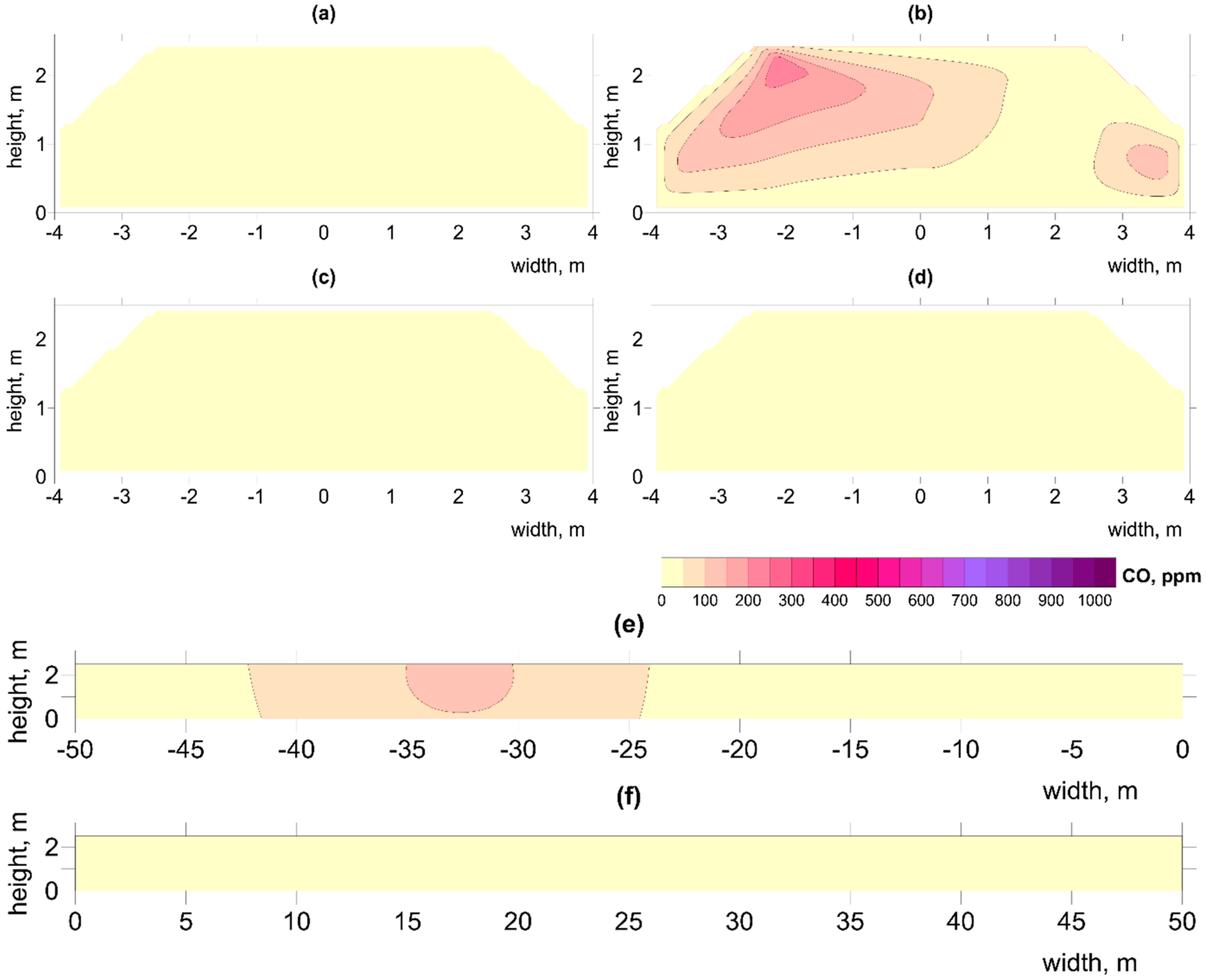

Figure S167. CO distribution in the pile C2, day 35.

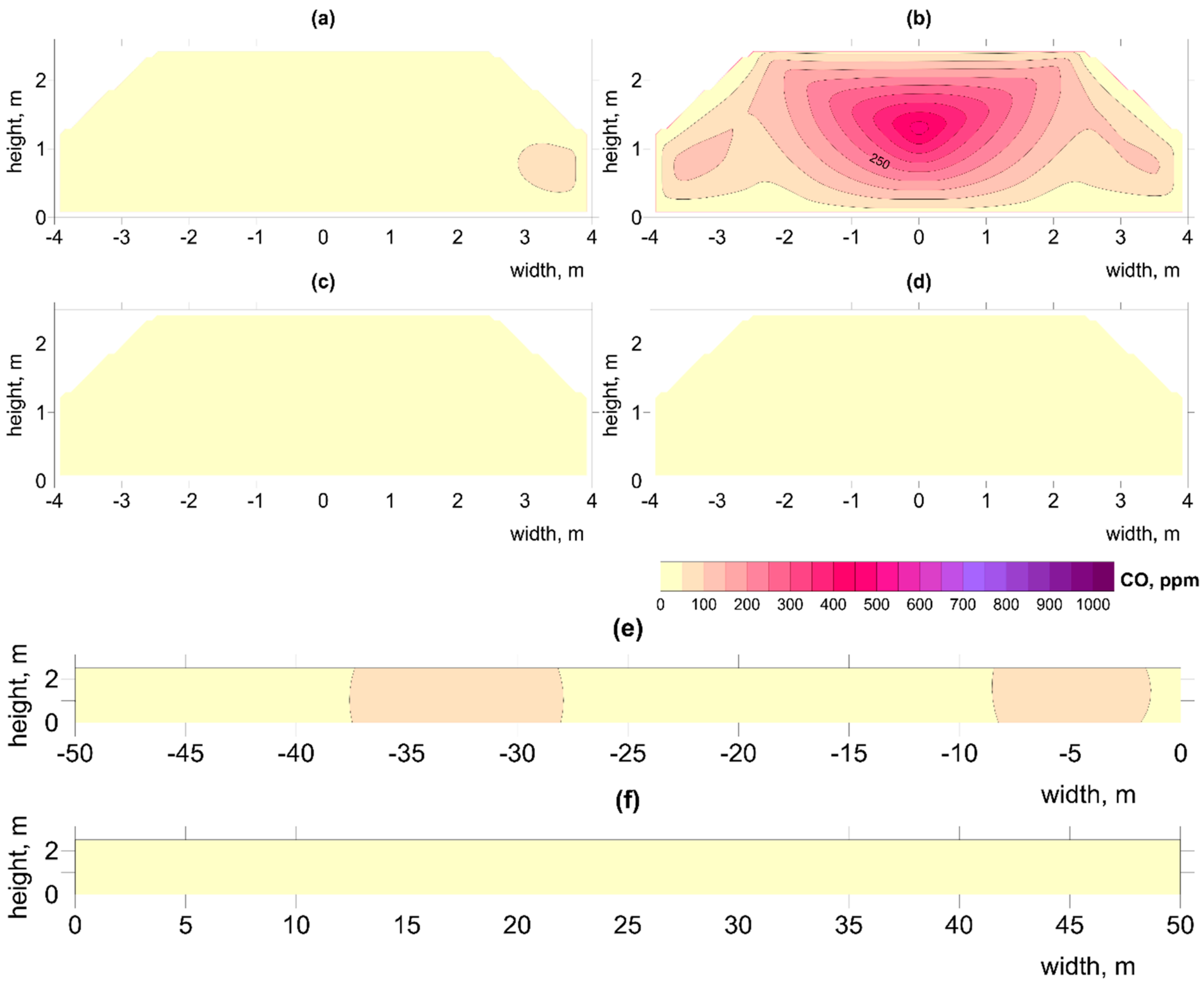

Figure S168. CO distribution in the pile C2, day 42.
